# Investigating causal relationships of blood and urine biomarkers with urological cancer risks: a mendelian randomization study and colocalization analyses

**DOI:** 10.7150/jca.103669

**Published:** 2025-01-01

**Authors:** Jian Li, Bing Yang, Lei Guo, Wenqi Huang, Qiong Hu, Hongting Yan, Rong Tan, Dongxin Tang

**Affiliations:** 1 The First College of Clinical Medicine, Guizhou University of Traditional Chinese Medicine, Guiyang, Guizhou, China.; 2 Student Management Office, The First Affiliated Hospital of Guizhou University of Traditional Chinese Medicine, Guiyang, Guizhou, China.; 3 Department of Geratology, The First Affiliated Hospital of Guizhou University of Traditional Chinese Medicine, Guiyang, Guizhou Province, China.; 4 Department of Oncology, The First Affiliated Hospital of Guizhou University of Traditional Chinese Medicine, Guiyang, Guizhou Province, China.; 5 Department of Pharmaceutics, The First Affiliated Hospital of Guizhou University of Traditional Chinese Medicine, Guiyang, Guizhou, China.

**Keywords:** blood biomarkers, urine biomarkers, urological cancers, causal association, Mendelian randomization

## Abstract

**Background:** Establishing the causal links between biomarkers and cancer enhances understanding of risk factors and facilitates the discovery of therapeutic targets. To this end, we used Mendelian randomization (MR) and colocalization analysis to explore the causal relationship of blood and urinary biomarkers (BUBs) with urological cancers (UCs).

**Methods:** First, we used a two-sample MR study to explore the causal relationship between 33 BUBs and 4 UCs, while we performed reverse Mendelian randomization. After Bonferroni correction, for BUB and UC with significant causality we confirmed the direct causality by multivariate MR adjusting for relevant risk factors. We also applied two-step MR analysis to further explore the possible mediators between BUB and UC with significant causality, while colocalization analysis was performed for BUB, UC and possible mediators. Sensitivity analysis were performed to assess the robustness of the results.

**Results:** A two-sample MR study found that there were 8 BUBs of CA, IGF-1, LPA, TP, CRE, BILD, TBIL and NAP with potential causality with some UCs (p<0.05), but after Bonferroni correction only IGF-1 had a significant causality with PCa (OR = 1.14, 95% CI: 1.06-1.23; p=0.0006<0.05/33). Moreover, the causal relationship between IGF-1 and PCa remained significant (P<0.05) after adjusting for relevant risk factors in the multivariate MR study. The two-step MR study found SHBG to be a mediator between IGF-1 and PCa, and the colocalization analysis found that there was a common causal variant (nearby gene TNS3) between IGF-1 and SHBG (PPH4=93.21%), which further confirmed the mediating effect of SHBG.

**Conclusion:** Strong evidence from our study suggests that IGF-1 increases the risk of PCa by decreasing SHBG levels, and in addition some BUBs were found to have a potential causal relationship with UCs.

## Introduction

Urological cancer (UC) encompasses a diverse range of tumors, including bladder cancer (BCa), prostate cancer (PCa), kidney cancer (KCa), and renal pelvis cancer (RPCa), among others. These cancers pose a significant threat to global health^1^. Since 1990, the incidence of UC has increased 2.5-fold and the mortality rate has increased 1.6-fold^2^, with this increase being particularly pronounced among men, accounting for approximately 33% of all reported malignancies in males^1^. This epidemiological reality underscores the urgent need to deepen our understanding of these cancers. However, each cancer type has distinct pathogenesis, necessitating different diagnostic and therapeutic approaches^3^. Biomarkers are substances that can be objectively measured and evaluated as indicators of normal biological processes, pathogenic processes, or pharmacological responses to a therapeutic intervention. Clinically, they are used in disease diagnosis, prognosis, monitoring therapeutic efficacy, and drug development. Therefore, elucidating the relationship between biomarkers and cancer not only aids in exploring the association between biomarkers and cancer risk but also can effectively improve cancer diagnosis rates and facilitate the exploration and development of better therapeutic targets.

Blood and urine biomarkers (BUB) are common laboratory tests, and recent observational studies have identified a close relationship between some biomarkers and UC^4^, ^5^. However, there are significant challenges in translating the findings of observational studies into effective cancer prevention and control strategies. This is because traditional observational designs are susceptible to various biases, such as residual confounding and reverse causality. Despite statistical and methodological efforts to address these issues, these biases often persist, making it difficult for observational studies to reliably establish causality between exposures and outcomes.

Mendelian Randomization (MR) is an epidemiological method that uses genetic variation to assess causal relationships. It utilizes genetic variants as instrumental variables (IVs) to investigate the causal effects of exposure factors (e.g., biomarkers) on diseases (e.g., cancer). MR studies can overcome many limitations of traditional observational studies, providing more reliable evidence of causal relationships^6^. To this end, we performed a Mendelian randomization (MR) analysis to explore the causal associations of 33 BUBs with 4 UCs (BCa, PCa, RPCa, KCa). We hope that this study will contribute to the research on the prevention and treatment of UC.

## Materials and methods

### Study design

**Figure 1:**
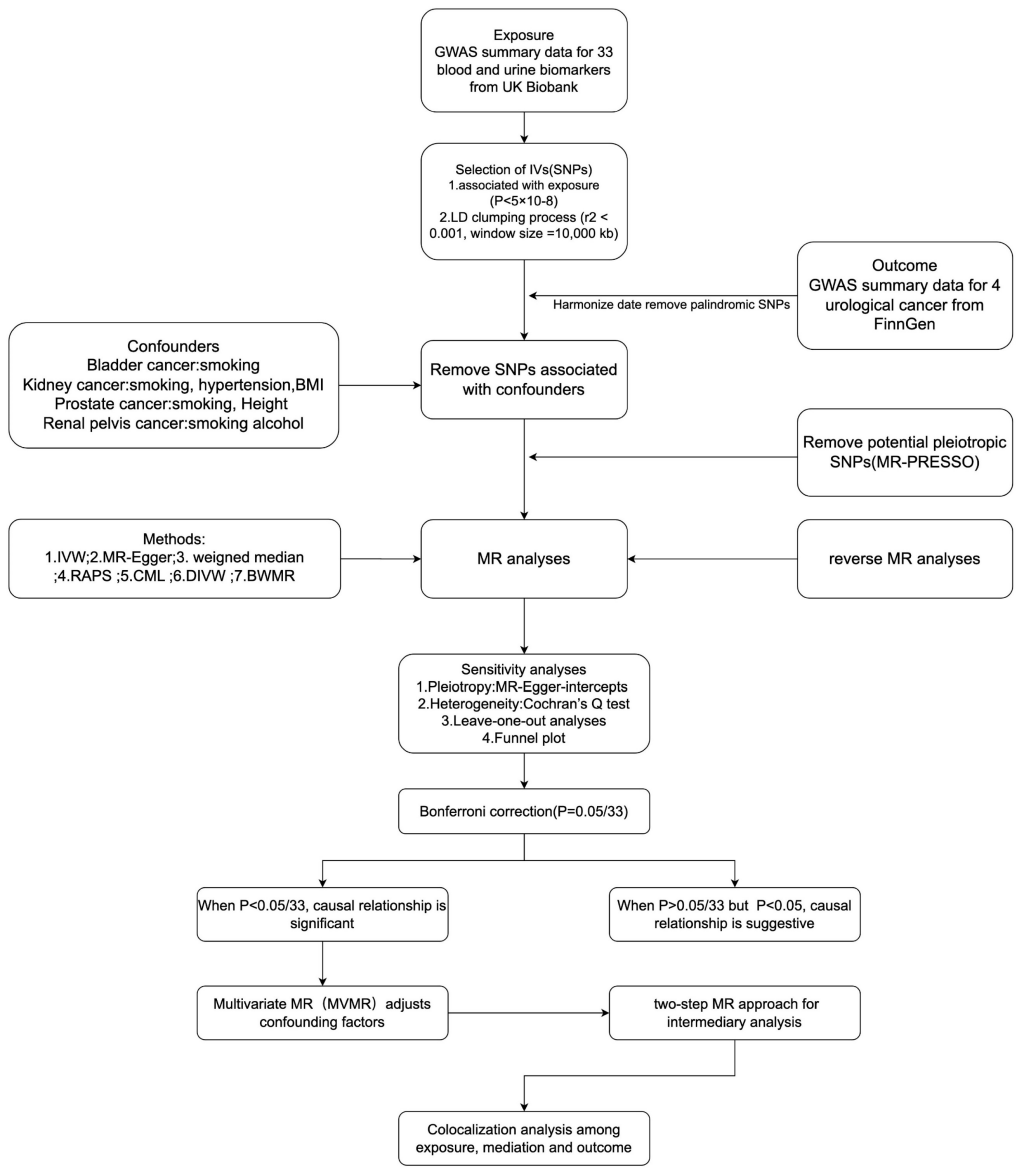
The overview of the study design and flowchart in this study (by Figdraw). SNPs, Single nucleotide polymorphisms; GWAS, Genome-wide association study; LD, Linkage disequilibrium; MR, Mendelian randomization; IVW, Inverse variance weighted; WM, weighted median; RAPS, Robust adjusted profile score; cML, Constrained maximum likelihood; DIVW, Debiased inverse-variance weighted method; BWMR, Bayesian weighted Mendelian randomization.

MR studies must satisfy three key core assumptions^7^:(1) The genetic variants used as IVs must be associated with the exposure of interest; (2) The genetic variants must be independent of confounders; (3) The genetic variants must affect the outcome only through the exposure and not via any other pathway. Our MR study design meets the three core assumptions and adheres to the STROBE-MR guidelines (Supplementary STROBE-MR-checklist). We used a two-sample MR study to explore the causal relationship between 33 BUBs and 4 UCs, while we performed reverse Mendelian randomization. After Bonferroni correction, for BUB and UC with significant causality we confirmed the direct causality by multivariate MR adjusting for relevant risk factors. We also applied two-step MR analysis to further explore the possible mediators between BUB and UC with significant causality, while colocalization analysis was performed for BUB, UC and possible mediators. Sensitivity analysis were performed to assess the robustness of the results. The entire study design is shown in Figure 1.

### Data sources

All data used in the MR analysis were obtained from publicly available genome-wide association studies (GWAS). Data for 33 BUBs were derived from a GWAS study of the UK Biobank (UKB), which included 363,228 participants of European ancestry^8^. We obtained the R10 version of the GWAS summary data for BCa, PCa, KCa, and RPCa from the FinnGen Consortium (https://finngen.gitbook.io/documentation/). Four UC were diagnosed according to ICD-O-3, controls excluding all cancers. As PCa risk factors smoking (N=4,772), height (N=360,388) and as mediators hormone binding globulin (SHBG) (N=180,094), total testosterone (TTES) (N=194,453), bioavailable testosterone (BTES) (N=178,782), estradiol (EST) (N=206,927), their genetic related data are all from the IEU Open GWAS database. (https://gwas.mrcieu.ac.uk/). For more information on the above data, please refer to Supplementary Table S1.

### Instrumental variable selection

We used single nucleotide polymorphisms (SNPs) as IVs for genetic variation. First, we extracted SNPs with genome-wide significance for exposure in GWAS (p < 5 × 10^-8^). Second, we excluded SNPs with linkage disequilibrium (LD) (r2< 0.001, clumping distance=10,000 kb) to eliminated highly associated SNPs. Third, we harmonize the effect sizes and alleles of SNPs in the exposure and outcome data. To prevent weak instrumental variable bias, SNPs with F-statistics < 10 were removed (F=R^2^ (n-k-1)/k(1-R^2^)). We looked for confounding factors associated with BCa^9^, PCa^10^, KCa^11^, and RPCa^12^. To minimize the bias caused by confounders we screened out SNPs strongly associated (p < 5 × 10^-8^) with confounders through the catalog website (https://www.ebi.ac.uk/gwas/). All removed SNPs associated with confounders were shown in Supplementary Table S3. In addition, we identified and excluded horizontal pleiotropic outliers using the MR-PRESSO test, and the excluded SNPs are shown in Supplementary Table S5. Finally, we used SNPs that met all the above criteria as IVs for the MR analysis. The characteristics of the SNPs used in this study are presented in Supplementary Table S2.

### Statistical analysis

We used the inverse-variance weighted (IVW) method as the primary MR analysis. IVW assumes that all genetic instruments are valid and provides a weighted average of the SNP-specific causal estimates. This method offers high statistical power under the assumption that there is no horizontal pleiotropy^13^. Cochrane's Q-tests were performed to scrutinize SNP-related heterogeneity for each exposure. In the presence of significant heterogeneity (p < 0.05), a fixed-effects IVW (FE-IVW) model was used; conversely, a random-effects IVW (RE-IVW) model was used. In addition, we performed a variety of other complementary MR Methods, including MR-Egger, weighted median (WM), Constrained maximum likelihood (cML), Debiased inverse-variance weighted method (dIVW), Robust adjusted profile score (RAPS), and Bayesian weighted Mendelian randomization (BWMR) to bolster the robustness and credibility of the MR outcomes. The MR-Egger method uses the regression intercept as an indicator to test potential multiple effects, and a P value less than 0.05 indicates pleiotropic effects. When more than 50% of the IVs are valid, the results of the weighted median method are reliable. cML is used to exclude bias caused by correlated and uncorrelated pleiotropy^14^. The dIVW method eliminates the weak instrumental bias of the IVW method and is more robust under many weak instruments^15^. RAPS allows the inclusion of weak instrumental variables and provides robust statistical estimates for MR through these weak instruments^16^. BWMR can not only take into account the uncertainty of estimated weak and weak level multinomial effects, but can also adaptively detect outliers due to a small number of large level multinomial effects, allowing causal inferences to be made despite the presence of multinomial effects^17^. The leave-one-out method was used in sensitivity analysis to assess the effect of individual SNPs on overall causal estimates. In addition, we performed sensitivity analysis on the MR results using scatter plots and funnel plots. We used the Steiger Test to detected the selected SNPs for potential reverse causality between BUB and UC. Due to the multiple testing of each type of UC with 33 different BUBs measures, we applied Bonferroni correction. A p-value of less than 0.0015(0.05/33=0.0015) was considered indicative of a significant causal relationship, while results with p-values between 0.05 and 0.0015 were considered indicative of a suggestive causal relationship. In addition, reverse MR analysis was performed to verify whether there was an inverse causal relationship between the 4 UCs and 33 BUBs. After Bonferroni correction, for BUB and UC with significant causality we confirmed the direct causality by multivariate MR adjusting for relevant risk factors.

To understand the potential causal mechanism between IGF-1 and PCa, mediation analysis was performed. Previous studies have found a strong relationship between sex hormone levels and PCa risk, so we selected SHBG, TTES, BTL, and EST as potential mediators. We first explored the causal effects of IGF-1 with potential mediators to identify important mediators. We then analyzed the causal relationships between possible mediators and PCa (among them, the SNPs included in the analysis of mediators to IGF-1 will remove the SNPs used in the IGF-1-PCa) (Table 1). In addition, we performed colocalization analysis on exposures, possible mediators and outcomes that showed a causal relationship with MR studies to determine whether they share the same causal variant^18^. Posterior probability of colocalization (PPH4) >80% indicates support for a common causal variant.

All statistical analysis and data visualizations were performed with the “TwoSampleMR”, “MRPRESSO”, “Forestplot” and “MRcML” R packages in R software version 4.3.3 (R Foundation for Statistical Computing, Vienna, Austria).

## Results

### Two-sample MR

**Figure 2:**
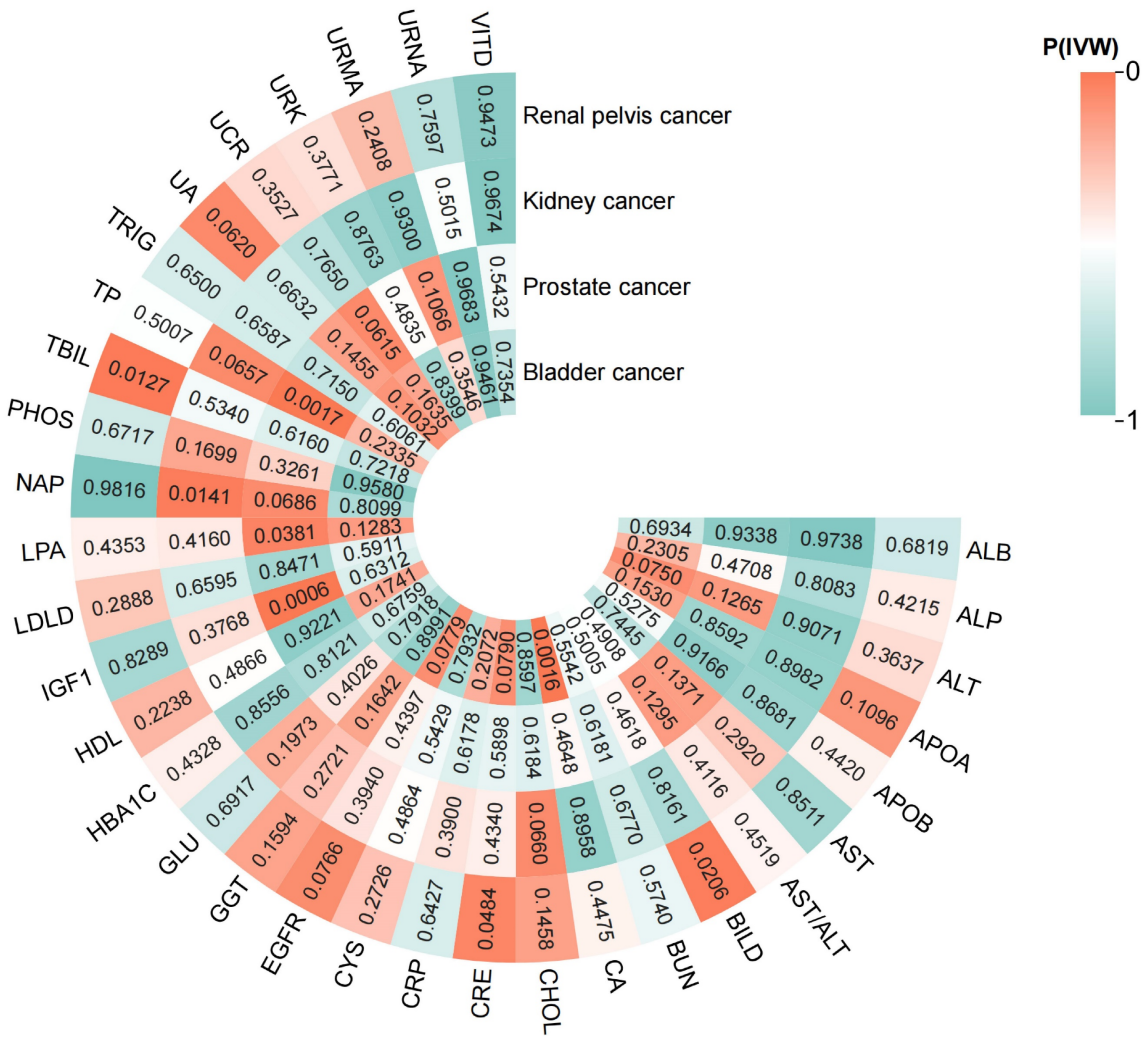
The heatmap of IVW analysis results for 33 blood and urine biomarkers (BUBs) with 4 urological cancers (UCs). All BUB abbreviations are shown in supplementary table S1. (Drawing is performed by site www.chiplot.online).

**Figure 3:**
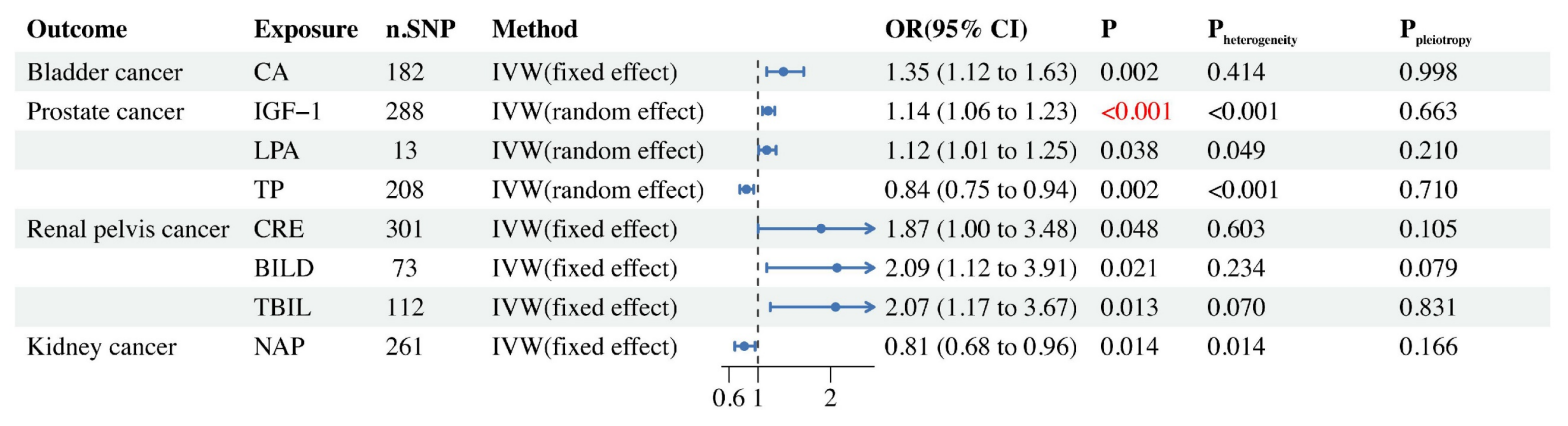
Two-sample MR forest plot of BUB causally related to UC.

The results of the two-sample MR analysis (IVW method) for 33 BUBs with 4UCs are presented in Figure 2. We found that 8 BUBs were associated with UC risk (Figure 3) and reverse Mendelian analysis does not find that there is no reverse causality (Supplementary Table S8). All results are shown to be robust through sensitivity analysis, details are provided in the Supplementary Material.

In BCa, we found a potential link between CA and its risk (Figure 3). Through Cochran's Q test (Supplementary Table S6), we did not find heterogeneity in gene IVS related to CA (P_heterogeneity_=0.414), so we selected the FE-IVW as the main MR analysis. FE-IVW results showed that CA increased the risk of BCa (OR = 1.35, 95% CI: 1.12-1.63; p=0.002). WM (OR = 1.60, 95% CI: 1.17-2.19; p=0.004), cML(OR = 1.36, 95% CI: 1.11-1.67; p=0.004), dIVW(OR = 1.36, 95% CI: 1.12-1.64; p=0.002), RAPS(OR = 1.37, 95% CI: 1.13-1.67; p=0.001), and BWMR(OR = 1.36, 95% CI: 1.12-1.65; p=0.002) methods also yielded results consistent with FE-IVW. However, MR-Egger (OR = 1.35, 95% CI: 0.95-1.93; p=0.096) results showed no statistical significance, but exhibited the same trend.

Our MR analysis showed that IGF-1 had a significant causal relationship with PCa risk, but LPA and TP may have a potential causal relationship with PCa risk (Figure 3). We selected RE-IVW as the primary MR analysis because its Cochran's Q test results showed heterogeneity (P_heterogeneity_<0.05), as shown in Supplementary Table S6. The results of RE-IVW analysis found that IGF-1 (OR = 1.14, 95% CI: 1.06-1.23; p<0.001) and LPA (OR = 1.12, 95% CI: 1.01-1.25; p=0.038) were associated with increased risk of PCa (Figure 3). The MR-Egger, WM, cML, dIVW, RAPS and BWMR methods for both of them also obtained results consistent with RE-IVW (Supplementary Table S4). The results of the RE-IVW method revealed that TP (OR = 0.84, 95% CI: 0.75-0.94; p=0.002) can reduce the risk of PCa, and the WM (OR = 0.80, 95% CI: 0.68-0.93; p=0.005), cML(OR = 0.84, 95% CI: 0.76-0.94; p=0.001), dIVW(OR = 0.84, 95% CI: 0.75-0.94; p=0.002), RAPS(OR = 0.85, 95% CI: 0.75-0.95; p=0.005), and BWMR (OR = 0.84, 95% CI: 0.76-0.94; p=0.002) methods also confirmed this result. MR Egger analysis failed to detect a statistically significant association but indicated a similar trend (OR = 0.88, 95% CI: 0.68-1.13; p=0.319).

Cochran's Q test for CRE (P_heterogeneity_=0.603), BILD (P_heterogeneity_=0.234) and TBIL (P_heterogeneity_=0.070) in RPCa did not reveal the presence of heterogeneity (Supplementary Table S6). The results of FE-IVW analysis revealed that CRE (OR = 1.87, 95% CI: 1.00-3.48; p=0.048), BILD (OR = 2.09, 95% CI: 1.12-3.91; p=0.021), and TBIL (OR = 2.07, 95% CI: 1.17-3.67; p=0.013) were potentially associated with the risk of RPCa. In CRE, no statistically significant association was found between the MR Egger (OR = 0.65, 95% CI: 0.16-2.68; p=0.552) and WM (OR = 1.19, 95% CI: 0.43-3.27; p=0.735) methods, but the WM method showed a similar trend to FE-IVW, while the MR Egger method indicated an opposite trend to FE-IVW. All other MR analysis methods for CRE, BILD and TBIL gave consistent results with their corresponding FE-IVW (Supplementary Table S4).

In KCa, we only found that NAP may reduce the risk of its (Figure 3), a result confirmed by six MR analysis, FE-IVW (OR = 0.81, 95% CI: 0.68-0.96; p=0.014), WM (OR = 0.73, 95% CI: 0.55-0.98; p=0.037), cML (OR = 0.78, 95% CI: 0.64-0.95; p=0.014), dIVW (OR = 0.80, 95% CI: 0.67-0.96; p=0.014), RAPS (OR = 0.77, 95% CI: 0.64-0.92; p=0.004) and BWMR (OR = 0.80, 95% CI: 0.67-0.95; p=0.013). However, the MR-Egger method obtained the opposite trend, but it was not statistically significant (OR = 1.02, 95% CI: 0.70-1.49; p=0.914).

### MVMR

**Figure 4:**
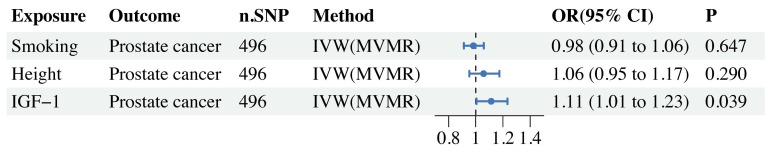
MVMR forest plot of IGF-1 and risk factors (smoking, height) with prostate cancer.

In the MVMR study, there was still a significant causal relationship between IGF-1 and PCa after adjusting for related risk factors such as smoking and height (Figure 4).

### Two-step MR

**Figure 5:**
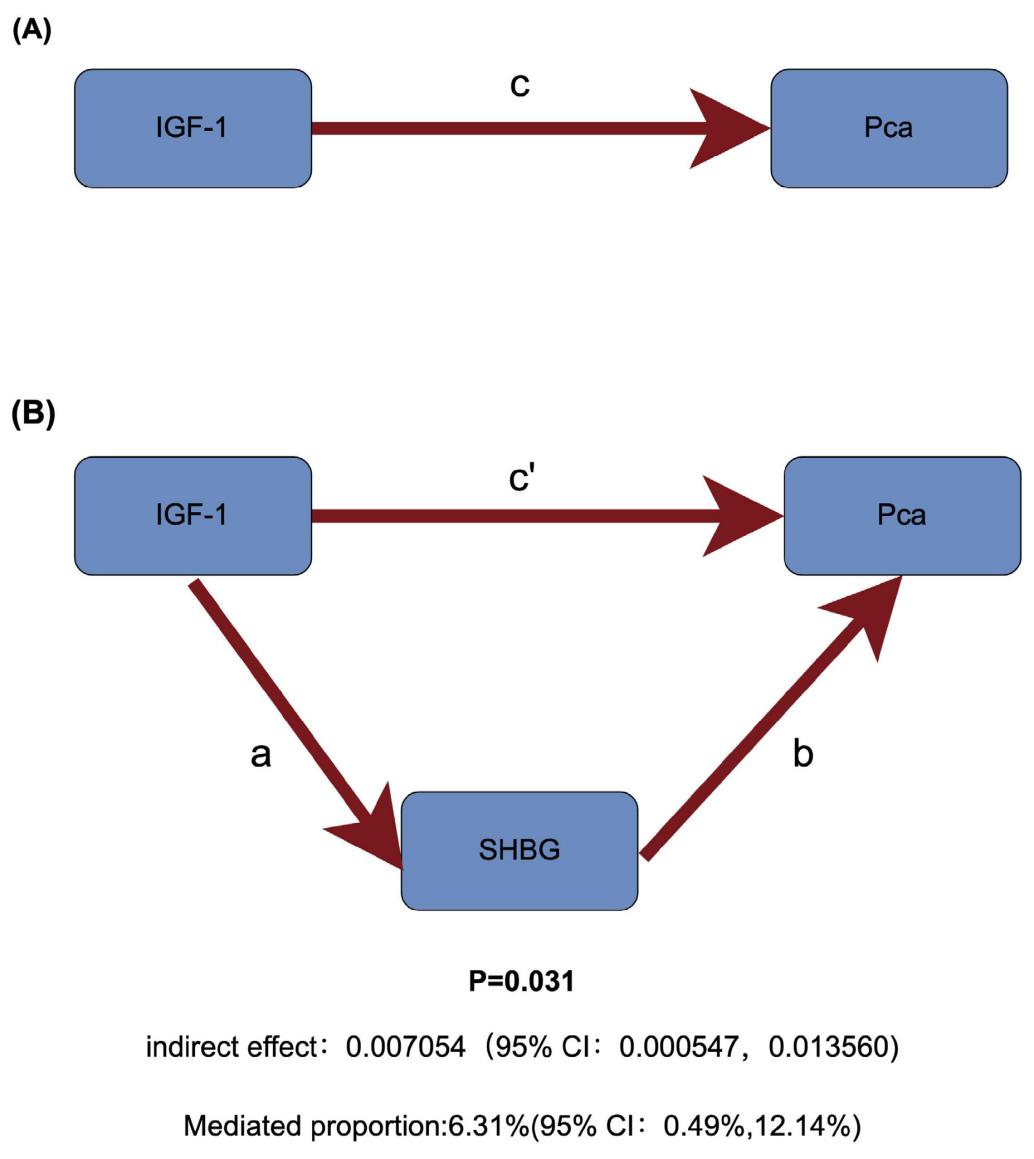
Schematic diagram of mediating effects of SHBG levels. (A) The total effect between insulin-like growth factor 1 (IGF-1) and prostate cancer (PCa). c is the total effect using genetically predicted IGF-1 as exposure and PCa as outcome. (B) The total effect was decomposed into: (i) indirect effect using a two-step approach (where a is the total effect of IGF-1 on sex hormone-binding globulin (SHBG), and b is the effect of IGF-1 on PCa) and the product method (a × b); (ii) direct effect (c′ = c - a × b). Proportion mediated was the indirect effect divided by the total effect.

Among sex hormones, a two-step MR study identified SHBG as a potential mediator of IGF-1 and PCa (Figure 5). Specifically, increased IGF-1 concentration can downregulate SHBG levels (Beta=-0.033, 95% CI: -0.044to-0.022), and increased SHBG levels can reduce the risk of PCa (OR = 0.807, 95% CI: 0.674-0.967). Therefore, IGF-1 increases prostate cancer risk by reducing SHBG levels (Table 1).

### Colocalization analysis

**Figure 6:**
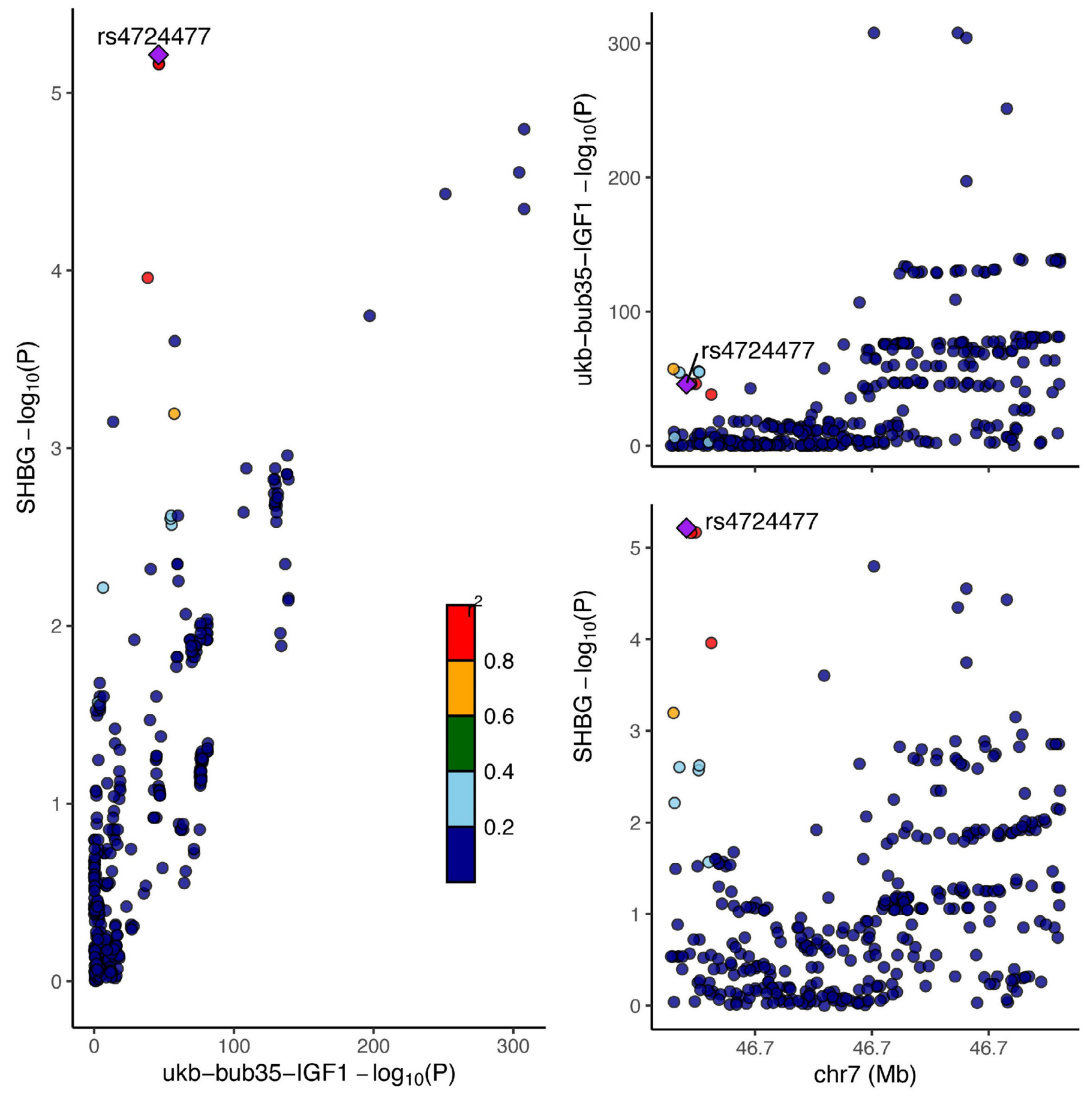
Association map of Lead SNPs on chromosomes for IGF-1 and SHBG sharing causal variants.

In the colocalization analysis of IGF-1, SHBG and PCa, we only found that IGF-1 shared a common causal variant with SHBG (PPH4 = 93.21%), which further confirmed the mediating effect of SHBG, as detailed in Table 2. In addition, colocalization analysis studies revealed that the Lead SNP for IGF-1 sharing a causal variant with SHBG is rs4724477 and it is close to the TNS3 gene (Figure 6).

## Discussion

The mortality and morbidity of UC are high worldwide and their diagnostic and therapeutic interventions are limited^1^, ^19^. Therefore, it is important to develop new strategies for the treatment of UC. This study systematically investigated the causal relationship between 33 common BUBs and 4 UCs using MR analysis, which provides direction for research on the prevention and treatment of UC.

BCa is a common form of UC for which smoking, gender and age are the main risk factors^9^. Some observational studies have found strong links between BUB and BCa, and they may help in diagnosis and treatment of the disease^20^, ^21^. Our MR study found that serum CA increased the risk of BCa. Serum CA is an important electrolyte in the body that is necessary for various physiologic processes, and some studies have found that the higher the serum CA, the worse the prognosis for cancer patients 22. In BCa, a previous retrospective study found that high serum CA increased the risk of bone metastasis in BCa^23^. However, we did not find other correlation studies between CA and BCa, and the relationship between the two remains unclear. Inflammation is widely recognized as a critical contributor to carcinogenesis^24^, including the development of bladder cancer^9^. Emerging evidence suggests that elevated serum CA levels may activate the NF-κB pathway, promoting the expression of pro-inflammatory mediators via calmodulin and protein kinase C^25^, ^26^. Additionally, CA can stimulate NLRP3 inflammasome activation, facilitating the maturation and release of key inflammatory cytokines, such as IL-1β and IL-18, through both direct and indirect mechanisms^27^. CA also activates specific ion channels, including TRPV4, further enhancing the secretion of pro-inflammatory factors^28^, ^29^. These pathways collectively suggest that elevated serum CA may contribute to bladder cancer risk by driving inflammatory responses. Moreover, calcium-dependent signaling pathways, including calmodulin and protein kinase C, play pivotal roles in regulating the cell cycle, cell proliferation, and apoptosis^30^. Elevated serum CA levels may dysregulate these processes, promoting abnormal proliferation of bladder epithelial cells through these pathways, which could further increase the risk of bladder cancer development.

PCa is a common malignancy among men, with more than 1.4 million new cases diagnosed annually^1^, and its major risk factors include age, family history , etc^10^. Our MR analysis identified associations between IGF-1, LPA, and TP with the risk of PCa. Specifically, we found that IGF-1 significantly increased the risk of PCa. IGF-1 is a peptide hormone produced in the human body that plays a crucial role in metabolism, tissue repair, and cell survival. Our findings are consistent with previous observational studies, further supporting the critical role of IGF-1 in the pathogenesis of PCa^31^-^33^. Experimental studies have found that IGF-1 can promote cancer cell proliferation and migration as well as inhibit apoptosis through AKT/FOXO3A/BIM^34^, PI3K-AKT-mTOR^35^ and RAS-MAPK^36^ pathways, and it has also been found that it promotes angiogenesis in prostate cancer^37^, which may be one of the reasons for the increase in the risk of PCa by IGF-1. However, we further found through a two-step Mendelian randomization study that IGF-1 increases the risk of PCa by reducing SHBG. In PCa, we were able to find a linear relationship between elevated IGF-1 and total SHBG from observational studies, but due to methodological issues in epidemiological studies we were unable to confirm that it was IGF-1 that downregulated SHBG synthesis^38^. Although the specific mechanism by which IGF-1 reduces SHBG is not clear, our co-localisation analysis study revealed that IGF-1 and SHBG share a common causal variant, and the leader SNP of the variant is rs4724477 (near TNS3 gene). These SNPs and genes may serve as important targets for future studies on the relationship between IGF-1 and SHBG. From our results, we speculate that IGF-1 may inhibit hepatic synthesis of SHBG by activating various downstream signalling pathways such as PI3K/AKT and MAPK through its receptor IGF1R^39^. In addition, IGF-1 is closely related to obesity, and it may also affect hepatic lipid metabolism and regulate SHBG through signalling pathways in adipocytes^40^. The fact that lower SHBG can increase the risk of PCa is perhaps better explained. It is known that the growth and development of prostate cancer usually depends on androgen stimulation such as testosterone, and androgen receptor inhibitors are one of the commonly used drugs for the treatment of prostate cancer^41^. The biological activity of testosterone is reduced when combined with SHBG^42^. Therefore, reduced SHBG levels may increase testosterone bioactivity (increased levels of bioavailable testosterone), thereby increasing the carcinogenic effects of testosterone on PCa. In addition, IGF-1 can enhance the expression and activity of androgen receptors^43^. These may account for the increased risk of PCa with IGF-1 downregulation of SHBG. Our study also found that LPA may be a risk factor for PCa. Two previous prospective cohort studies have indicated a positive association between LPA levels and the risk of PCa^44^. Additionally, another MR study has yielded similar results^45^. Although there are no direct studies to elucidate the mechanism by which LPA promotes PCa, LPA is an important immune/inflammatory regulator^46^, so we speculate that it contributes to the development of PCa through pro-inflammation and modulation of immunity. In contrast, this study found that TP levels may be inversely related to PCa risk. Consistent with our findings, a cohort study from the UKB also found that higher TP levels are associated with a reduced risk of PCa (OR = 0.88, 95% CI: 0.84-0.93)^47^. Serum TP includes various proteins with anti-inflammatory and antioxidant properties, particularly albumin. Albumin has the ability to scavenge free radicals and reduce oxidative stress^48^, which is a significant factor in the occurrence and progression of cancer^49^. Additionally, immunoglobulins within total protein can enhance tumor immune responses through various mechanisms, effectively improving the ability to recognize and eliminate potential cancer cells^50^. These beneficial effects of TP may help explain why it can reduce the risk of PCa.

RPCa is a very rare malignant tumor of the kidney, and little is known about the epidemiology of this disease^51^. Results from our MR study suggest that elevated BILD, TBIL, and CRE may increase its risk. Bilirubin is the end product of heme metabolism, primarily derived from the breakdown of aging red blood cells and the decomposition of other heme-rich tissues. TBIL includes both BILD and indirect bilirubin, with BILD being converted from indirect bilirubin. Contrary to our findings, some studies have shown that bilirubin can reduce the risk of cancer^52^, ^53^, likely due to the antioxidant properties of direct bilirubin^53^. However, other studies have found that bilirubin is not associated with cancer risk^54^, and some even suggest that bilirubin may increase the risk of cancer^55^. For RPCa, there is currently no direct evidence that bilirubin can directly damage the renal pelvis and cause cancer. BILD is excreted into the small intestine via bile. In the intestine, BILD is converted by gut microbiota into urobilinogen and stercobilinogen. Some urobilinogen is filtered through the kidneys and enters the urine, where it can be oxidized to urobilin. This process may produce free radicals and reactive oxygen species (ROS). These free radicals and ROS can cause oxidative stress, leading to cellular damage and DNA mutations, which can contribute to carcinogenesis^49^. This may explain why bilirubin increases the risk of RPCa. Our MR analysis showed that CRE increased the risk of RPCa. Previous observational studies have found that renal insufficiency increases the risk of RPCa^56^, and our findings further confirm this conclusion, but the specific mechanism is unclear. Elevated levels of CRE are often a sign of renal insufficiency, which triggers chronic inflammation and oxidative stress, leading to cellular damage and an increased risk of cancer^57^, and as renal function declines, urinary toxins (e.g., CREs) are not excreted efficiently, resulting in their accumulation in the body, where they may cause direct damage to DNA or induce cancerous changes by interfering with normal cellular metabolism^58^. These may be the reasons why CRE increase the risk of RPCa.

In our MR analysis, we found that NAP might reduce the risk of KCa. Proteinuria is composed of albumin and NAP, and urinary NAP is related to kidney diseases such as tubulointerstitial damage^59^. From previous studies, it is clear that proteinuria and albuminuria are a risk factor for cancer (including KCa) 60-63, but there are no studies that have found an association between NAP in urine and cancer (including KCa). The components of non-albumin proteinuria typically consist of various NAP (such as immunoglobulins) derived from different physiological and pathological processes^64^. Among these, antioxidant-related proteins (such as superoxide dismutase)[65]and metal ion-binding proteins (such as ferritin and transferrin)^66^ can reduce oxidative stress levels. Additionally, some proteins, such as adiponectin and lectins, can inhibit the production of pro-inflammatory cytokines, including interleukin-6 and tumor necrosis factor-α, thereby reducing the inflammatory response^67^. It is possible that these anti-inflammatory and antioxidative effects of NAP contribute to the reduced risk of KCa.

The strength of our study is the comprehensive and systematic assessment of 33 BUBs and 4 UCs risks, as well as the exploration of their possible mechanisms. However, there are some limitations to this study. Firstly, the study population was limited to individuals of European ancestry, potentially restricting the applicability of the findings to other populations. Secondly, despite the use of various sensitivity tests to examine our results, it was not possible to test the independence and exclusion hypotheses in the MR analysis, so the possibility of multiple effects cannot be completely ruled out. Thirdly, since the GWAS data used are summary data and without specific information on individuals, we were unable to perform subgroup analysis. Fourth, our analysis was limited to cancer risk rather than progression and therefore may not provide information on the utility of targeted biomarkers in the context of cancer treatment. Because of these limitations, these findings need to be validated by studies in other ethnic populations, as well as further experimental and clinical studies to determine their potential value in clinical practice.

## Conclusions

In conclusion, strong evidence from our study suggests that IGF-1 increases the risk of PCa by decreasing SHBG levels, and our MR study identified potential associations between CA, LPA, TP, BILD, CRE, TBIL, and NAP with certain UCs risks. These findings warrant further research to understand these biomarkers' roles in urological cancers, explore their potential as therapeutic targets, and improve prevention and treatment strategies.

## Supplementary Material

Supplementary figures and information.

Supplementary tables.

## Figures and Tables

**Table 1:** Two-step Mendelian randomization study. Beta is used for continuous variables and OR is used for categorical variables.

Exposure	Outcome	n.SNP	Method	Beta/OR (95%cl)	Pvalue
IGF1	SHBG (mediator)	262	IVW	-0.033(-0.044to-0.022)	1.38E-08
IGF1	TTES (mediator)	282	IVW	-0.025 (-0.051to0.001)	0.056
IGF1	BTES (mediator)	319	IVW	0.033(0.011to0.055)	0.003
IGF1	EST (mediator)	325	IVW	0.001(0.003to0.006)	0.655
SHBG (mediator)	PCa	227	IVW	0.807 (0.674to0.967)	0.020
TTES (mediator)	PCa	149	IVW	1.014(0.927to1.110)	0.756
BTES (mediator)	PCa	76	IVW	1.067(0.922to1.235)	0.385
EST (mediator)	PCa	12	IVW	0.864 (0.208to3.581)	0.840

hormone binding globulin (SHBG), total testosterone (TTES), bioavailable testosterone (BTES), estradiol (EST)

**Table 2:** Colocalization analysis between IGF-1, SHBG and PCa. PP.H0 =neither exposure nor outcome has a genetic association in the region, PP.H1 = only exposure has a genetic association in the region, PP.H2 = only outcome has a genetic association in the region, PP.H3 = both exposure and outcome are associated but have different causal variants, PP.H4 = both exposure and outcome are associated and share a single causal variant.

Exposure	Outcome	n.SNPs	PP.H0	PP.H1	PP.H2	PP.H3	PP.H4
IGF-1	SHBG	359	1.50E-302	5.44%	3.97E-303	1.35%	93.21%
SHBG	PCa	382	2.72E-301	82.63%	4.99E-302	15.15%	2.21%
IGF-1	PCa	337	5.50E-301	96.50%	1.57E-302	2.75%	00.76%

## Abbreviations

BCa: Bladder cancer
BILD: direct bilirubin
BUB: blood and urine biomarkers
BWMR: Bayesian weighted Mendelian randomization
BTES: bioavailable testosterone
CA: calcium
cML: Constrained maximum likelihood
CRE: Creatinine
dIVW: Debiased inverse-variance weighted method
EST: estradiol
FE-IVW: fixed-effects Inverse variance weighted
GWAS: Genome-wide association study
IGF-1: insulin-like growth factor 1
IVs: Instrumental variables
IVW: Inverse variance weighted
KCa: Kidney cancer
LD: Linkage disequilibrium
LPA: lipoprotein A
MR: Mendelian randomization
MVMR: Multivariate Mendelian randomization
NAP: non-albumin protein
PCa: Prostate cancer
RAPS: Robust adjusted profile score
RE-IVW: random-effects Inverse variance weighted
ROS: reactive oxygen species
RPCa: renal pelvis cancer
SHBG: sex hormone-binding globulin
SNP: Single nucleotide polymorphism
TBIL: total bilirubin
TP: total protein
TTES: total testosterone
UC: urological cancers
UKB: UK Biobank
WM: weighted median
