# Supplementary material for: Investigating causal relationships of blood and urine biomarkers with urological cancer risks: a mendelian randomization study and colocalization analyses
Source: J Cancer. 2025 Jan 1;16(3):1020–31. doi: 10.7150/jca.103669 (PMC11705052; doi:10.7150/jca.103669)
Supplement: Supplementary file 1 — Supplementary figures and information. [file jcav16p1020s1.pdf]

Scatter plot of Bca

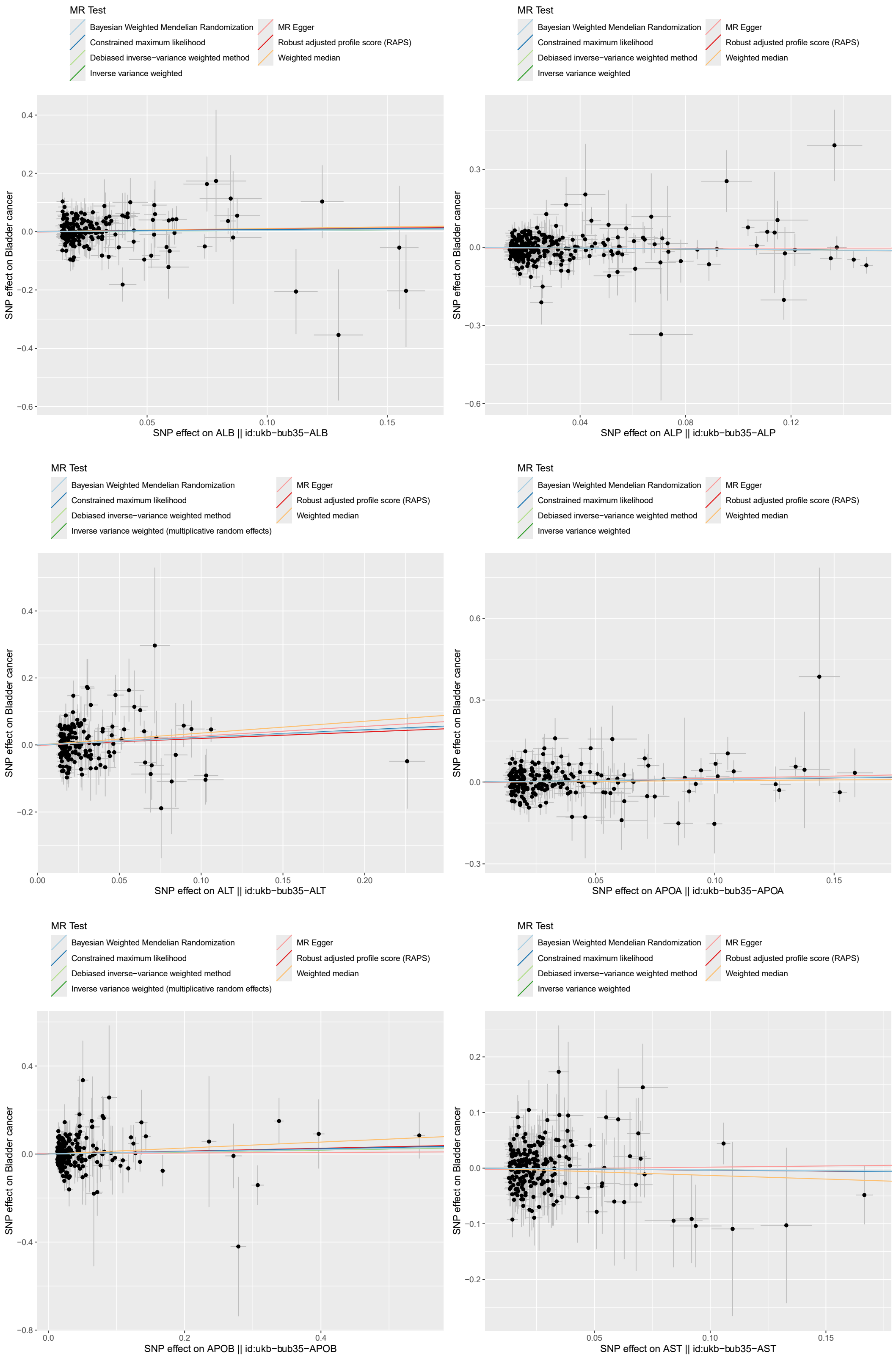

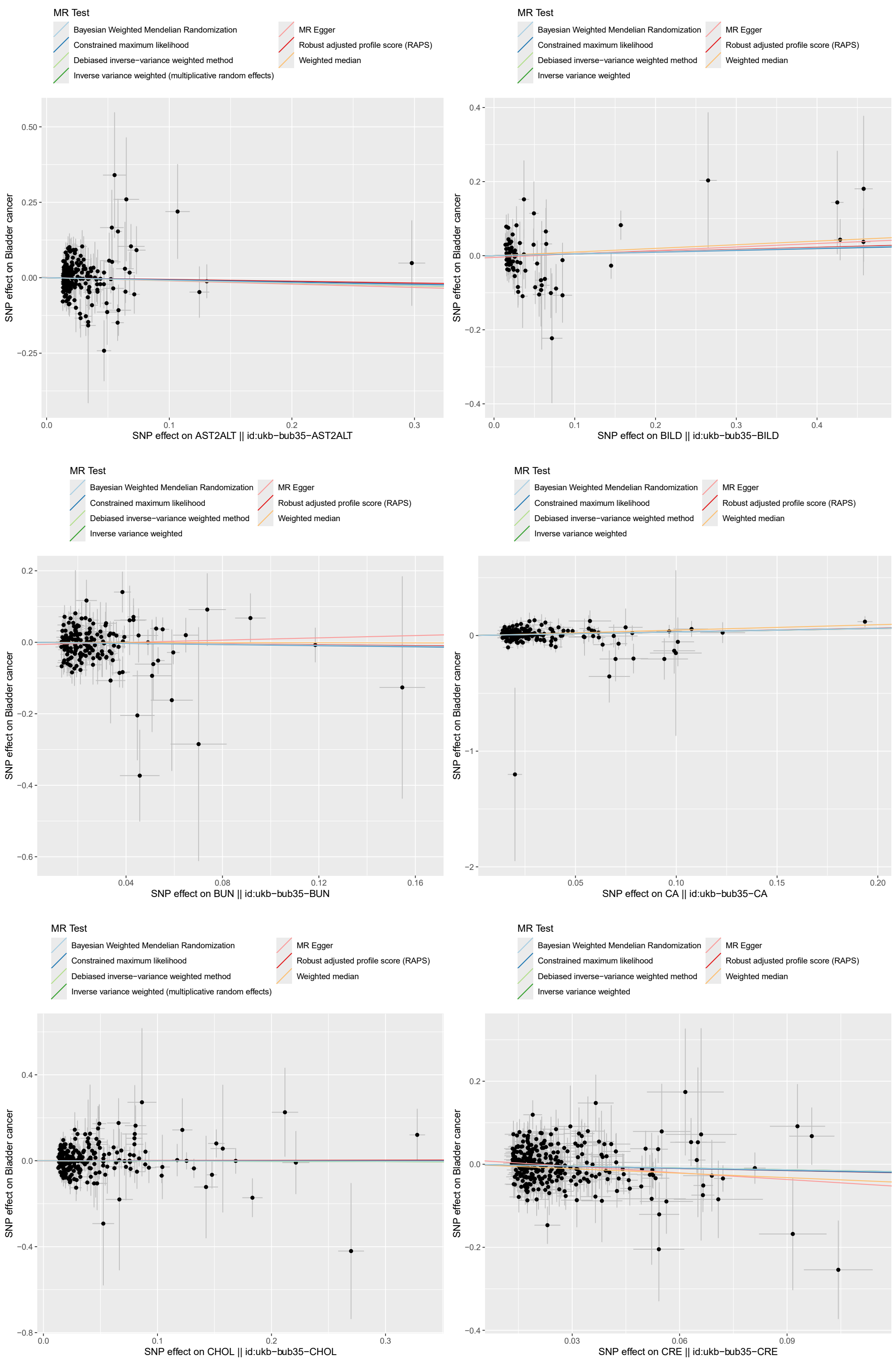

MR Test
Bayesian Weighted Mendelian Randomization
Constrained maximum likelihood
Debiased inverse−variance weighted method
Inverse variance weighted (multiplicative random effects)
MR Egger
Robust adjusted profile score (RAPS)
Weighted median
SNP effect on Bladder cancer
SNP effect on AST2ALT || id:ukb−bub35−AST2ALT
MR Test
Bayesian Weighted Mendelian Randomization
Constrained maximum likelihood
Debiased inverse−variance weighted method
Inverse variance weighted
MR Egger
Robust adjusted profile score (RAPS)
Weighted median
SNP effect on Bladder cancer
SNP effect on BILD || id:ukb−bub35−BILD
MR Test
Bayesian Weighted Mendelian Randomization
Constrained maximum likelihood
Debiased inverse−variance weighted method
Inverse variance weighted
MR Egger
Robust adjusted profile score (RAPS)
Weighted median
SNP effect on Bladder cancer
SNP effect on BUN || id:ukb−bub35−BUN
MR Test
Bayesian Weighted Mendelian Randomization
Constrained maximum likelihood
Debiased inverse−variance weighted method
Inverse variance weighted
MR Egger
Robust adjusted profile score (RAPS)
Weighted median
SNP effect on Bladder cancer
SNP effect on CA || id:ukb−bub35−CA
MR Test
Bayesian Weighted Mendelian Randomization
Constrained maximum likelihood
Debiased inverse−variance weighted method
Inverse variance weighted (multiplicative random effects)
MR Egger
Robust adjusted profile score (RAPS)
Weighted median
SNP effect on Bladder cancer
SNP effect on CHOL || id:ukb−bub35−CHOL
MR Test
Bayesian Weighted Mendelian Randomization
Constrained maximum likelihood
Debiased inverse−variance weighted method
Inverse variance weighted
MR Egger
Robust adjusted profile score (RAPS)
Weighted median
SNP effect on Bladder cancer
SNP effect on CRE || id:ukb−bub35−CRE

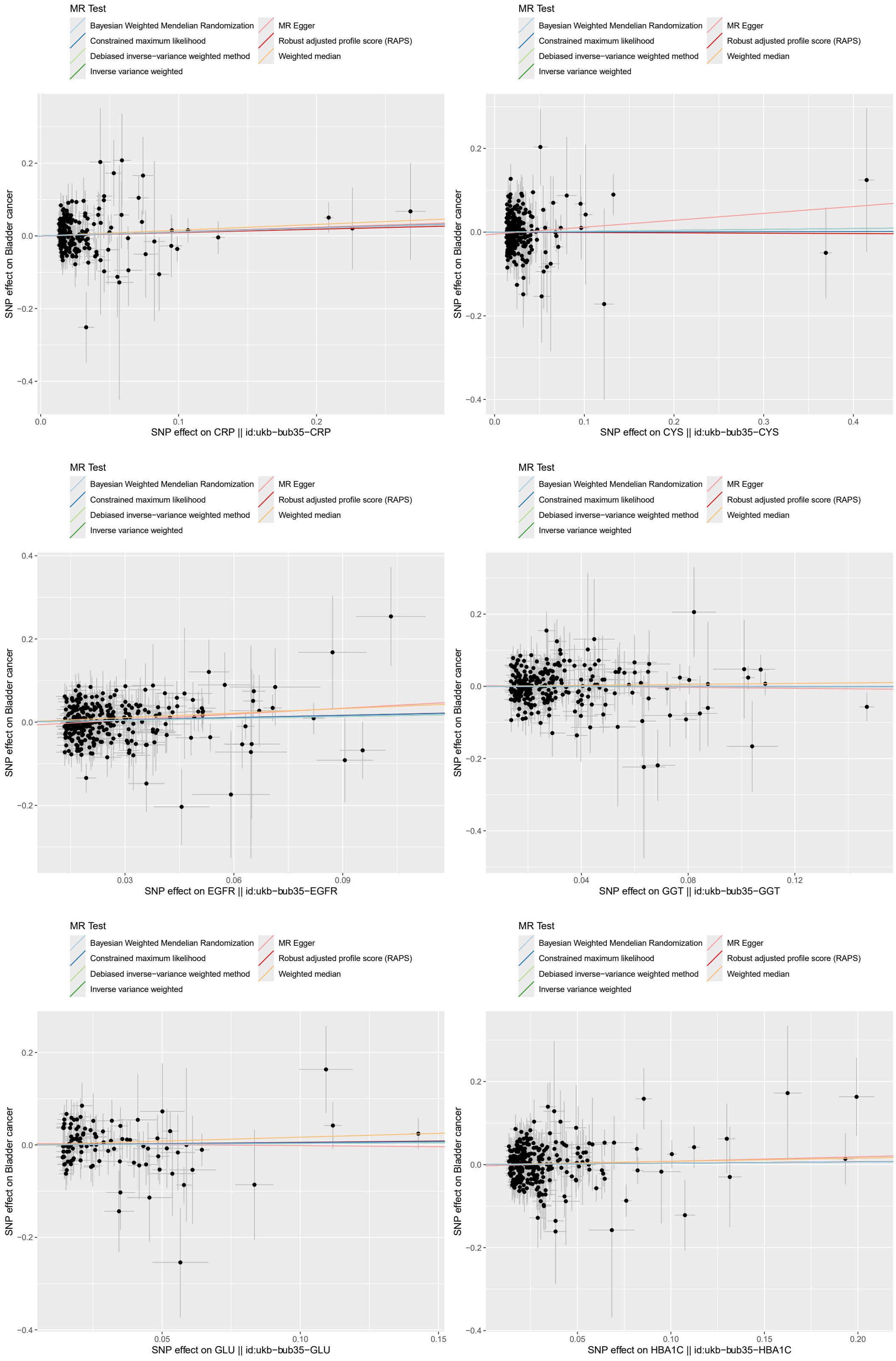
MR Test
Bayesian Weighted Mendelian Randomization
Constrained maximum likelihood
Debiased inverse−variance weighted method
Inverse variance weighted
MR Egger
Robust adjusted profile score (RAPS)
Weighted median
SNP effect on Bladder cancer
SNP effect on CRP || id:ukb−bub35−CRP
SNP effect on CYS || id:ukb−bub35−CYS
SNP effect on EGFR || id:ukb−bub35−EGFR
SNP effect on GGT || id:ukb−bub35−GGT
SNP effect on GLU || id:ukb−bub35−GLU
SNP effect on HBA1C || id:ukb−bub35−HBA1C

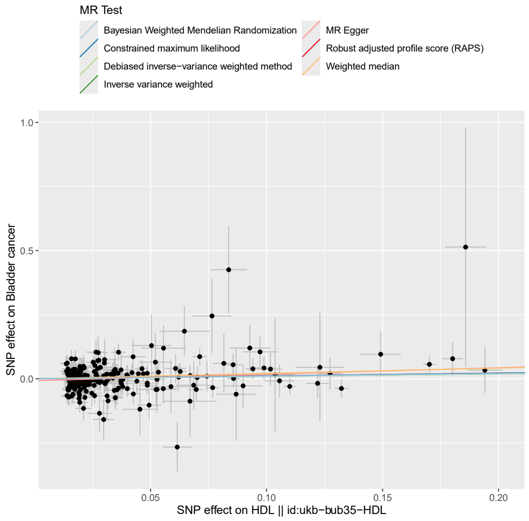
MR Test
Bayesian Weighted Mendelian Randomization
Constrained maximum likelihood
Debiased inverse−variance weighted method
Inverse variance weighted
MR Egger
Robust adjusted profile score (RAPS)
Weighted median
SNP effect on Bladder cancer
SNP effect on HDL || id:ukb-bub35-HDL

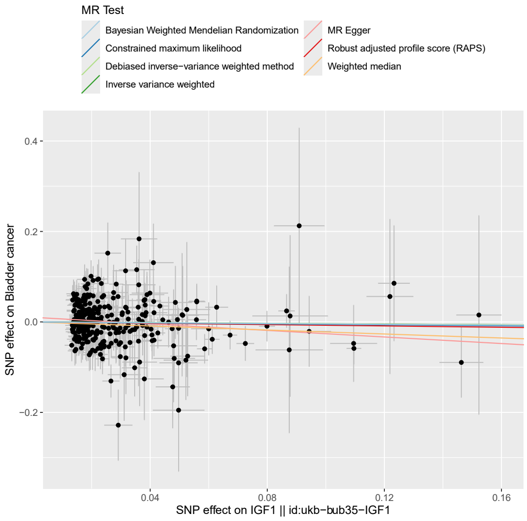
MR Test
Bayesian Weighted Mendelian Randomization
Constrained maximum likelihood
Debiased inverse−variance weighted method
Inverse variance weighted
MR Egger
Robust adjusted profile score (RAPS)
Weighted median
SNP effect on Bladder cancer
SNP effect on IGF1 || id:ukb-bub35-IGF1

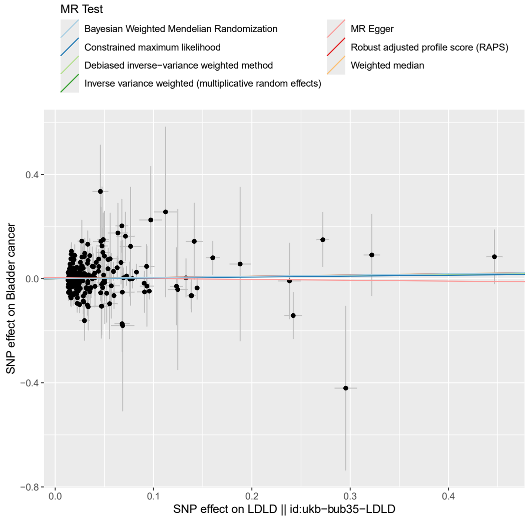
MR Test
Bayesian Weighted Mendelian Randomization
Constrained maximum likelihood
Debiased inverse−variance weighted method
Inverse variance weighted (multiplicative random effects)
MR Egger
Robust adjusted profile score (RAPS)
Weighted median
SNP effect on Bladder cancer
SNP effect on LDLD || id:ukb-bub35-LDLD

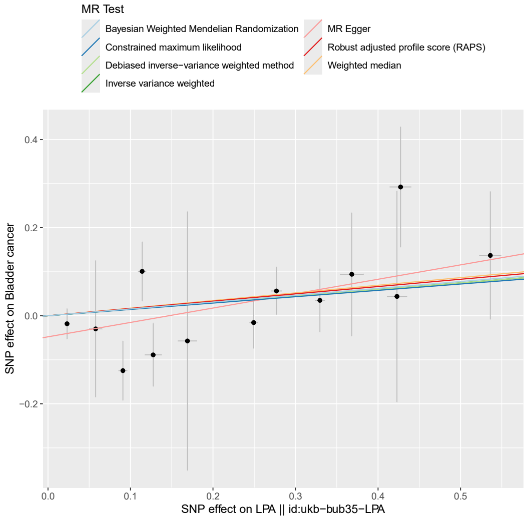
MR Test
Bayesian Weighted Mendelian Randomization
Constrained maximum likelihood
Debiased inverse−variance weighted method
Inverse variance weighted
MR Egger
Robust adjusted profile score (RAPS)
Weighted median
SNP effect on Bladder cancer
SNP effect on LPA || id:ukb-bub35-LPA

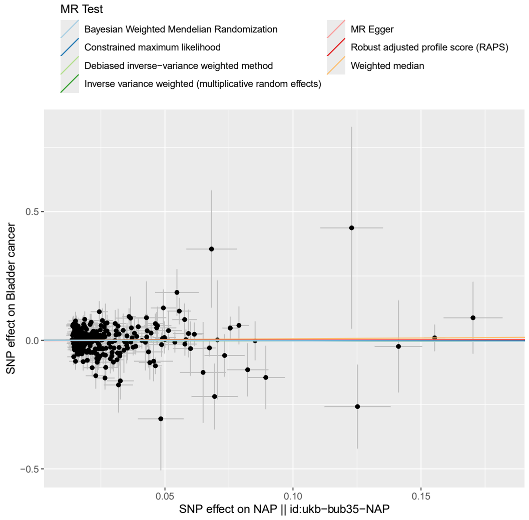
MR Test
Bayesian Weighted Mendelian Randomization
Constrained maximum likelihood
Debiased inverse−variance weighted method
Inverse variance weighted (multiplicative random effects)
MR Egger
Robust adjusted profile score (RAPS)
Weighted median
SNP effect on Bladder cancer
SNP effect on NAP || id:ukb-bub35-NAP

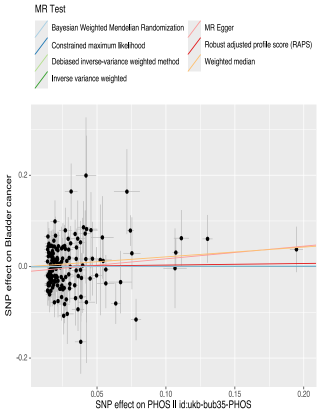
MR Test
Bayesian Weighted Mendelian Randomization
Constrained maximum likelihood
Debiased inverse-variance weighted method
Inverse variance weighted
MR Egger
Robust adjusted profile score (RAPS)
Weighted median
SNP effect on Bladder cancer
SNP effect on PHOS || id:ukb-bub35-PHOS

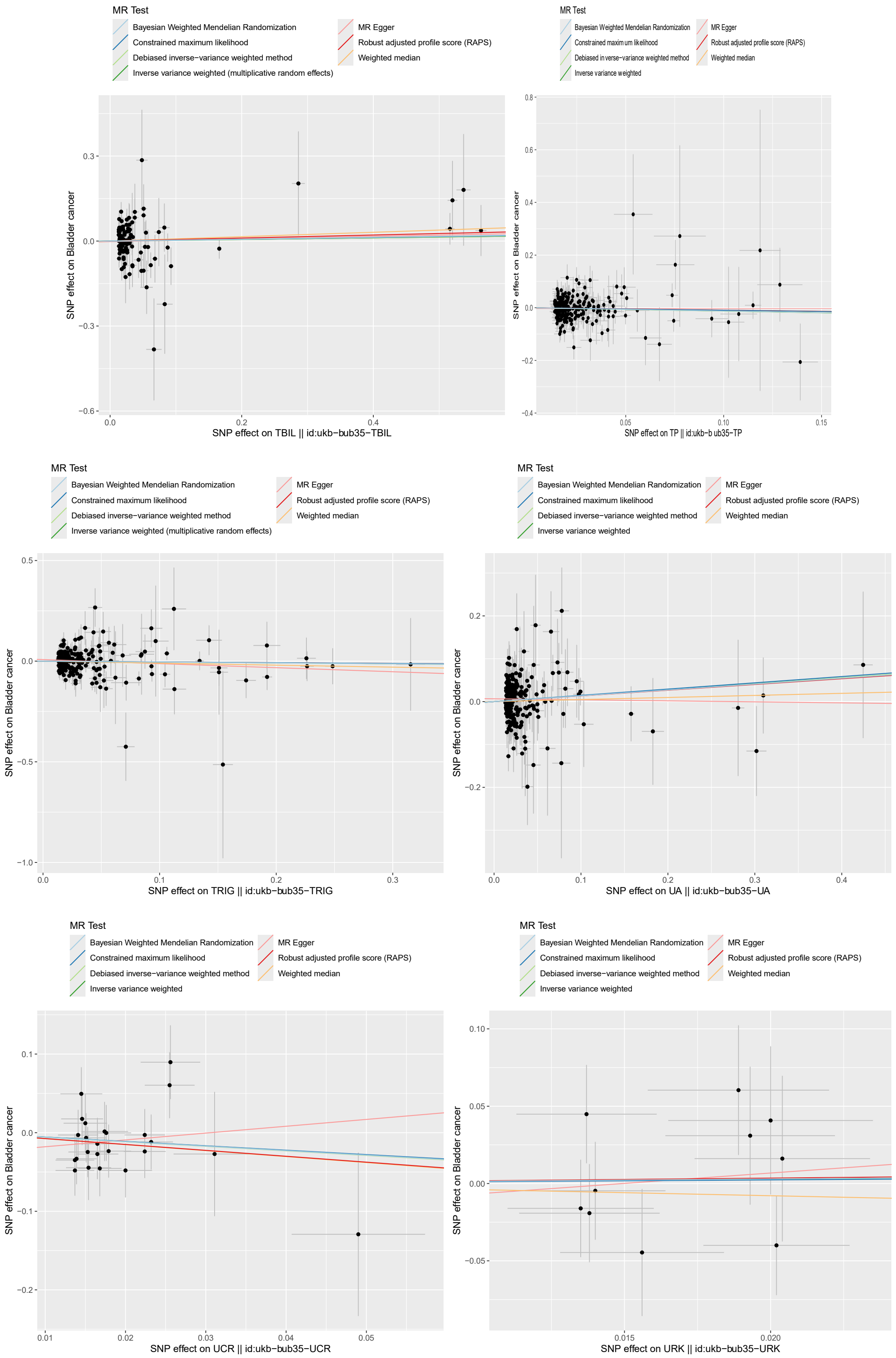
MR Test
Bayesian Weighted Mendelian Randomization
Constrained maximum likelihood
Debiased inverse−variance weighted method
Inverse variance weighted (multiplicative random effects)
MR Egger
Robust adjusted profile score (RAPS)
Weighted median
SNP effect on Bladder cancer
SNP effect on TBIL || id:ukb−bub35−TBIL
MR Test
Bayesian Weighted Mendelian Randomization
Constrained maximum likelihood
Debiased inverse−variance weighted method
Inverse variance weighted
MR Egger
Robust adjusted profile score (RAPS)
Weighted median
SNP effect on Bladder cancer
SNP effect on TP || id:ukb−bub35−TP
MR Test
Bayesian Weighted Mendelian Randomization
Constrained maximum likelihood
Debiased inverse−variance weighted method
Inverse variance weighted (multiplicative random effects)
MR Egger
Robust adjusted profile score (RAPS)
Weighted median
SNP effect on Bladder cancer
SNP effect on TRIG || id:ukb−bub35−TRIG
MR Test
Bayesian Weighted Mendelian Randomization
Constrained maximum likelihood
Debiased inverse−variance weighted method
Inverse variance weighted
MR Egger
Robust adjusted profile score (RAPS)
Weighted median
SNP effect on Bladder cancer
SNP effect on UA || id:ukb−bub35−UA
MR Test
Bayesian Weighted Mendelian Randomization
Constrained maximum likelihood
Debiased inverse−variance weighted method
Inverse variance weighted
MR Egger
Robust adjusted profile score (RAPS)
Weighted median
SNP effect on Bladder cancer
SNP effect on UCR || id:ukb−bub35−UCR
MR Test
Bayesian Weighted Mendelian Randomization
Constrained maximum likelihood
Debiased inverse−variance weighted method
Inverse variance weighted
MR Egger
Robust adjusted profile score (RAPS)
Weighted median
SNP effect on Bladder cancer
SNP effect on URK || id:ukb−bub35−URK

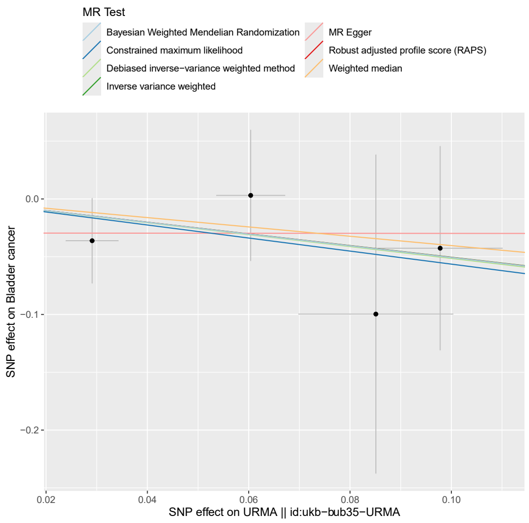
MR Test
Bayesian Weighted Mendelian Randomization
Constrained maximum likelihood
Debiased inverse−variance weighted method
Inverse variance weighted
MR Egger
Robust adjusted profile score (RAPS)
Weighted median
SNP effect on Bladder cancer
SNP effect on URMA || id:ukb−bub35−URMA
0.0
−0.1
−0.2
0.02
0.04
0.06
0.08
0.10

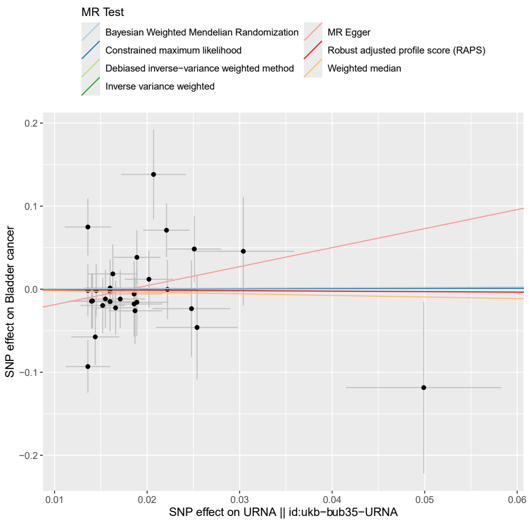
MR Test
Bayesian Weighted Mendelian Randomization
Constrained maximum likelihood
Debiased inverse−variance weighted method
Inverse variance weighted
MR Egger
Robust adjusted profile score (RAPS)
Weighted median
SNP effect on Bladder cancer
SNP effect on URNA || id:ukb−bub35−URNA
0.2
0.1
0.0
−0.1
−0.2
0.01
0.02
0.03
0.04
0.05
0.06

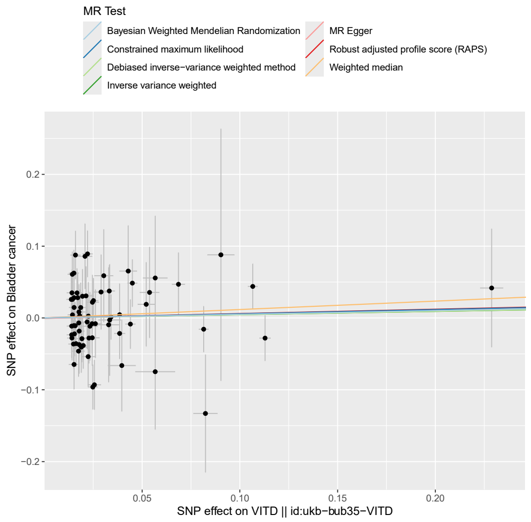
MR Test
Bayesian Weighted Mendelian Randomization
Constrained maximum likelihood
Debiased inverse−variance weighted method
Inverse variance weighted
MR Egger
Robust adjusted profile score (RAPS)
Weighted median
SNP effect on Bladder cancer
SNP effect on VITD || id:ukb−bub35−VITD
0.2
0.1
0.0
−0.1
−0.2
0.05
0.10
0.15
0.20

Scatter plot of PCa

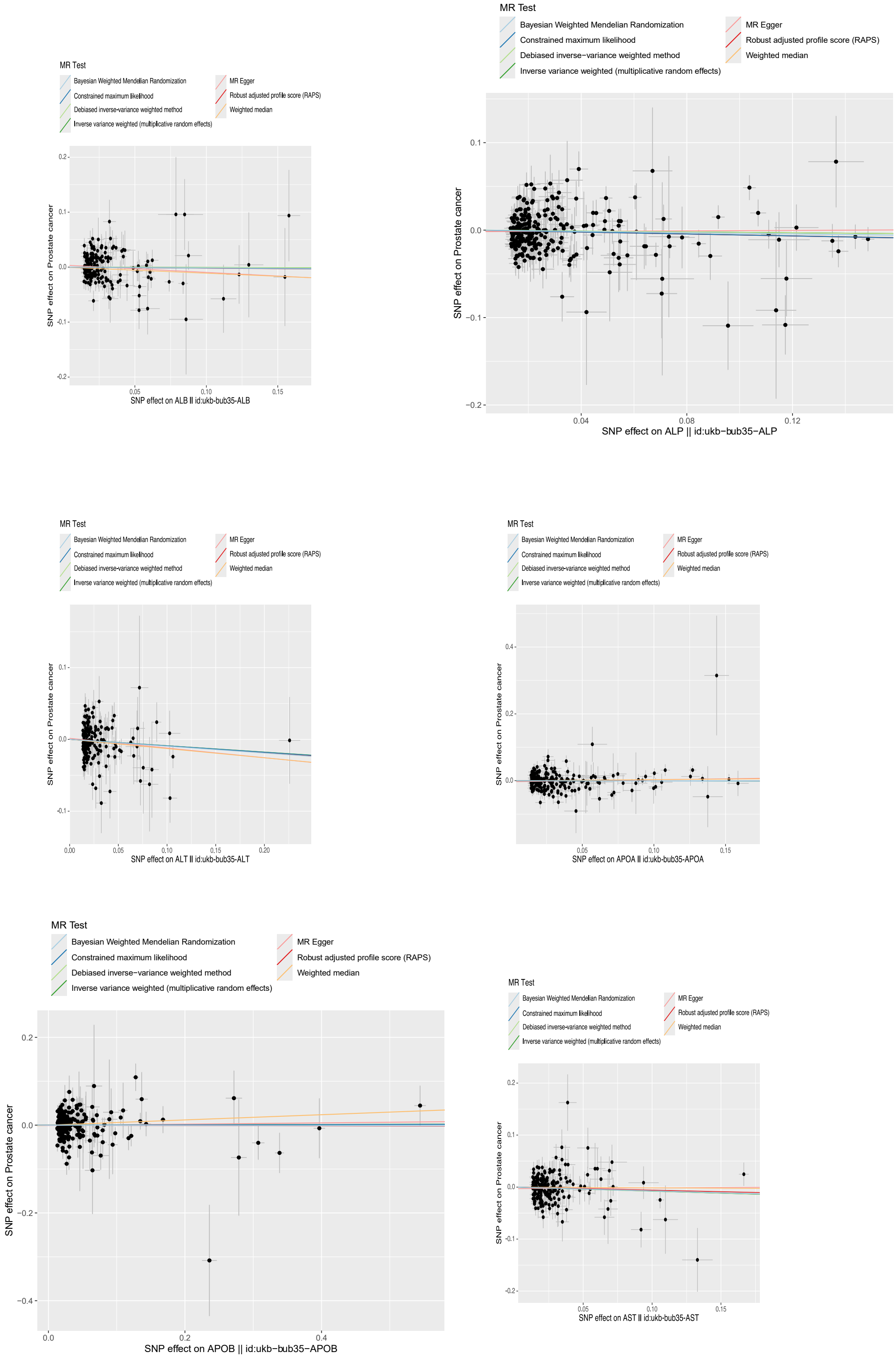

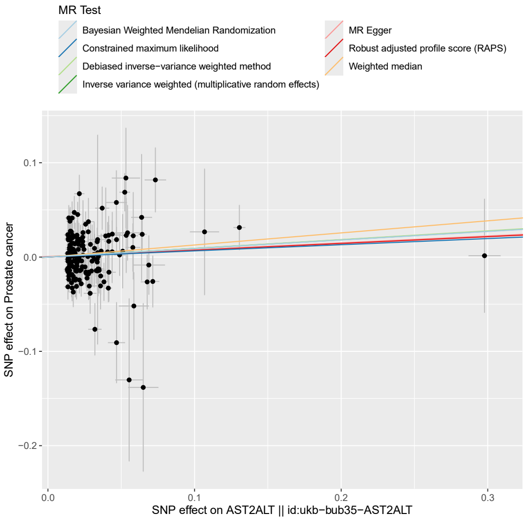

MR Test
Bayesian Weighted Mendelian Randomization
Constrained maximum likelihood
Debiased inverse−variance weighted method
Inverse variance weighted (multiplicative random effects)
MR Egger
Robust adjusted profile score (RAPS)
Weighted median
SNP effect on Prostate cancer
0.1
0.0
−0.1
−0.2
0.0
0.1
0.2
0.3
SNP effect on AST2ALT || id:ukb−bub35−AST2ALT

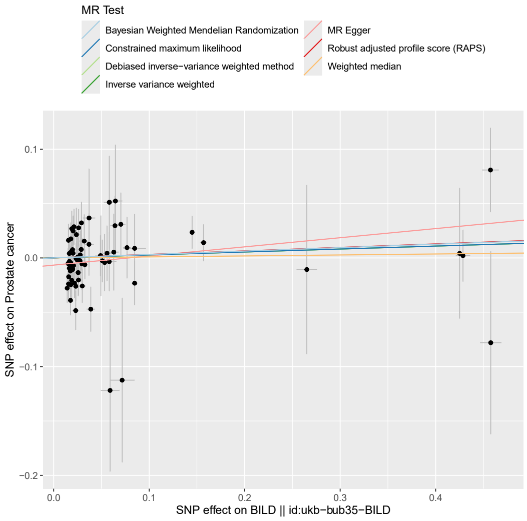

MR Test
Bayesian Weighted Mendelian Randomization
Constrained maximum likelihood
Debiased inverse−variance weighted method
Inverse variance weighted
MR Egger
Robust adjusted profile score (RAPS)
Weighted median
SNP effect on Prostate cancer
0.1
0.0
−0.1
−0.2
0.0
0.1
0.2
0.3
0.4
SNP effect on BILD || id:ukb−bub35−BILD

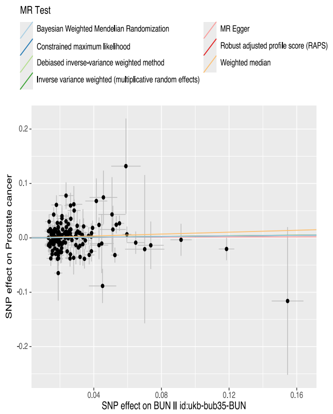

MR Test
Bayesian Weighted Mendelian Randomization
Constrained maximum likelihood
Debiased inverse-variance weighted method
Inverse variance weighted (multiplicative random effects)
MR Egger
Robust adjusted profile score (RAPS)
Weighted median
SNP effect on Prostate cancer
0.2
0.1
0.0
-0.1
-0.2
0.04
0.08
0.12
0.16
SNP effect on BUN || id:ukb-bub35-BUN

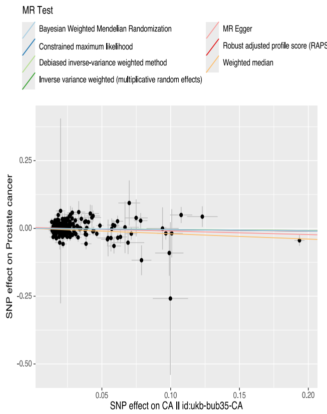

MR Test
Bayesian Weighted Mendelian Randomization
Constrained maximum likelihood
Debiased inverse-variance weighted method
Inverse variance weighted (multiplicative random effects)
MR Egger
Robust adjusted profile score (RAPS)
Weighted median
SNP effect on Prostate cancer
0.25
0.00
-0.25
-0.50
0.05
0.10
0.15
0.20
SNP effect on CA || id:ukb-bub35-CA

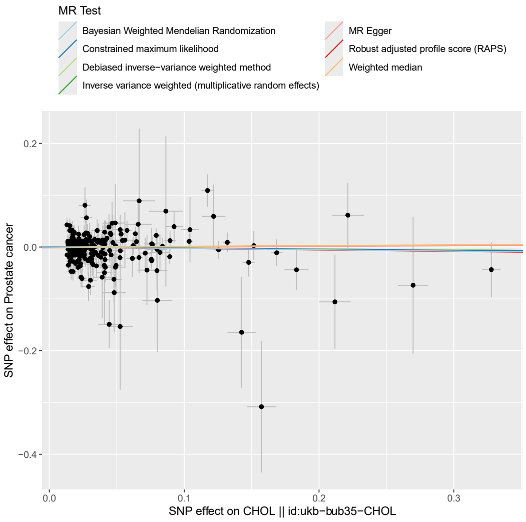

MR Test
Bayesian Weighted Mendelian Randomization
Constrained maximum likelihood
Debiased inverse−variance weighted method
Inverse variance weighted (multiplicative random effects)
MR Egger
Robust adjusted profile score (RAPS)
Weighted median
SNP effect on Prostate cancer
0.2
0.0
−0.2
−0.4
0.0
0.1
0.2
0.3
SNP effect on CHOL || id:ukb−bub35−CHOL

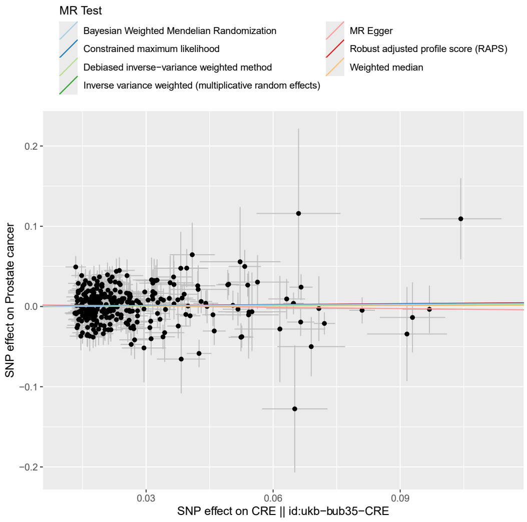

MR Test
Bayesian Weighted Mendelian Randomization
Constrained maximum likelihood
Debiased inverse−variance weighted method
Inverse variance weighted (multiplicative random effects)
MR Egger
Robust adjusted profile score (RAPS)
Weighted median
SNP effect on Prostate cancer
0.2
0.1
0.0
−0.1
−0.2
0.03
0.06
0.09
SNP effect on CRE || id:ukb−bub35−CRE

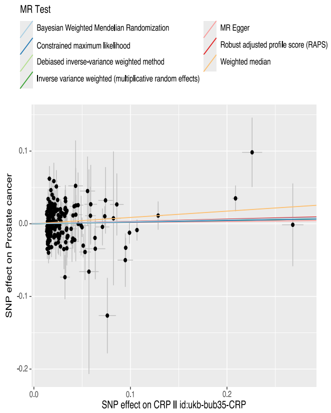
MR Test
Bayesian Weighted Mendelian Randomization
Constrained maximum likelihood
Debiased inverse-variance weighted method
Inverse variance weighted (multiplicative random effects)
MR Egger
Robust adjusted profile score (RAPS)
Weighted median
SNP effect on Prostate cancer
SNP effect on CRP || id:ukb-bub35-CRP

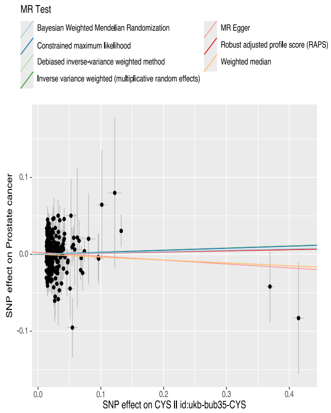
MR Test
Bayesian Weighted Mendelian Randomization
Constrained maximum likelihood
Debiased inverse-variance weighted method
Inverse variance weighted (multiplicative random effects)
MR Egger
Robust adjusted profile score (RAPS)
Weighted median
SNP effect on Prostate cancer
SNP effect on CYS || id:ukb-bub35-CYS

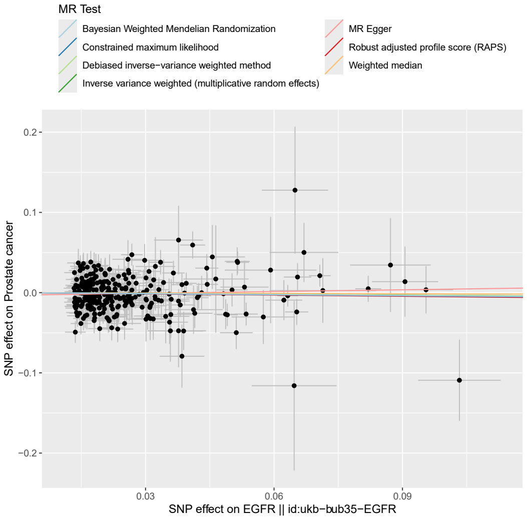
MR Test
Bayesian Weighted Mendelian Randomization
Constrained maximum likelihood
Debiased inverse−variance weighted method
Inverse variance weighted (multiplicative random effects)
MR Egger
Robust adjusted profile score (RAPS)
Weighted median
SNP effect on Prostate cancer
SNP effect on EGFR || id:ukb−bub35−EGFR

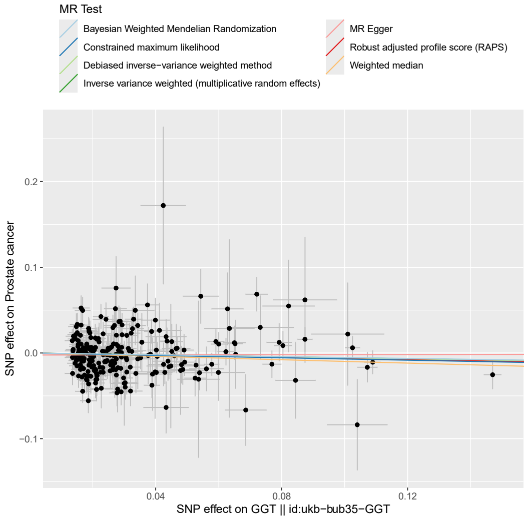
MR Test
Bayesian Weighted Mendelian Randomization
Constrained maximum likelihood
Debiased inverse−variance weighted method
Inverse variance weighted (multiplicative random effects)
MR Egger
Robust adjusted profile score (RAPS)
Weighted median
SNP effect on Prostate cancer
SNP effect on GGT || id:ukb−bub35−GGT

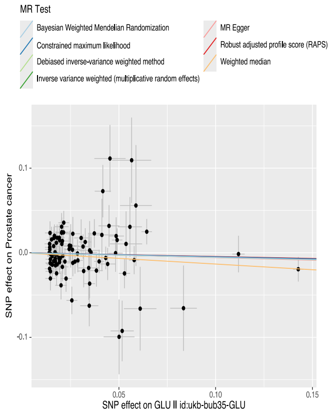
MR Test
Bayesian Weighted Mendelian Randomization
Constrained maximum likelihood
Debiased inverse-variance weighted method
Inverse variance weighted (multiplicative random effects)
MR Egger
Robust adjusted profile score (RAPS)
Weighted median
SNP effect on Prostate cancer
SNP effect on GLU || id:ukb-bub35-GLU

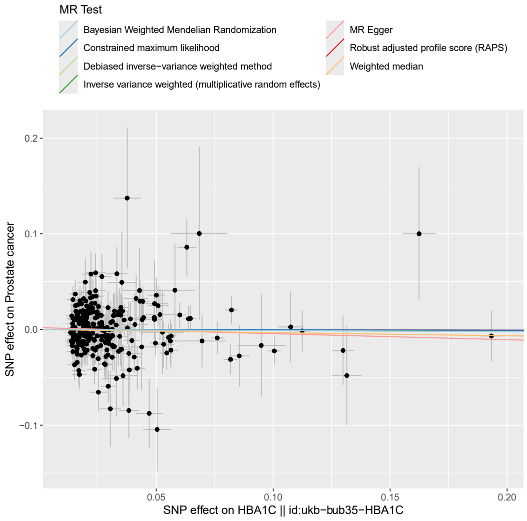
MR Test
Bayesian Weighted Mendelian Randomization
Constrained maximum likelihood
Debiased inverse−variance weighted method
Inverse variance weighted (multiplicative random effects)
MR Egger
Robust adjusted profile score (RAPS)
Weighted median
SNP effect on Prostate cancer
SNP effect on HBA1C || id:ukb−bub35−HBA1C

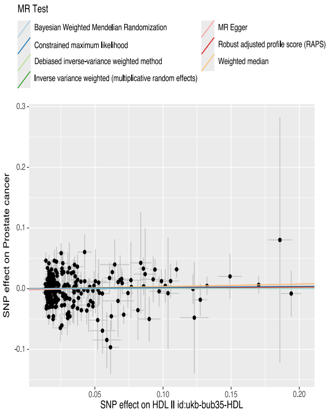
MR Test
Bayesian Weighted Mendelian Randomization
Constrained maximum likelihood
Debiased inverse-variance weighted method
Inverse variance weighted (multiplicative random effects)
MR Egger
Robust adjusted profile score (RAPS)
Weighted median
SNP effect on Prostate cancer
SNP effect on HDL || id:ukb-bub35-HDL

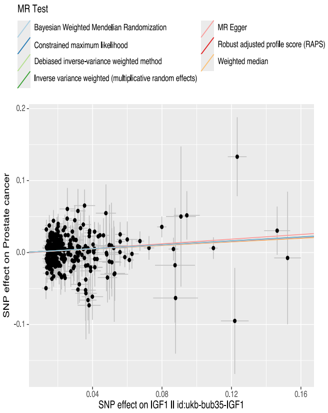
MR Test
Bayesian Weighted Mendelian Randomization
Constrained maximum likelihood
Debiased inverse-variance weighted method
Inverse variance weighted (multiplicative random effects)
MR Egger
Robust adjusted profile score (RAPS)
Weighted median
SNP effect on Prostate cancer
SNP effect on IGF1 || id:ukb-bub35-IGF1

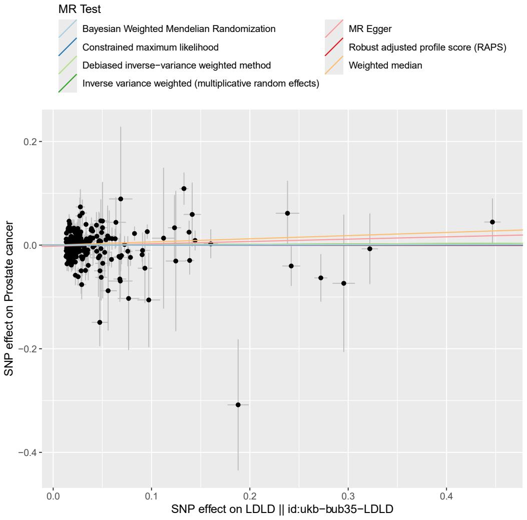
MR Test
Bayesian Weighted Mendelian Randomization
Constrained maximum likelihood
Debiased inverse−variance weighted method
Inverse variance weighted (multiplicative random effects)
MR Egger
Robust adjusted profile score (RAPS)
Weighted median
SNP effect on Prostate cancer
SNP effect on LDLD || id:ukb−bub35−LDLD

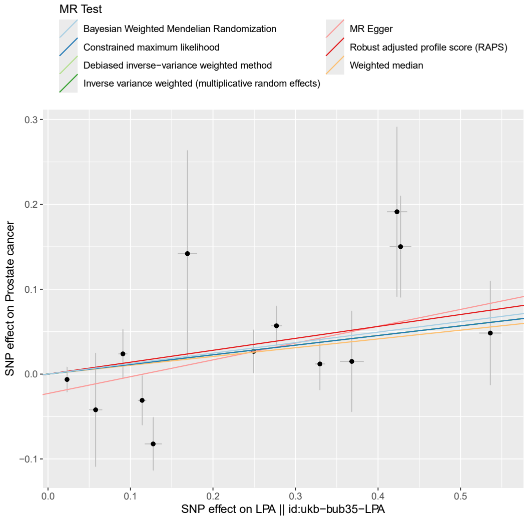
MR Test
Bayesian Weighted Mendelian Randomization
Constrained maximum likelihood
Debiased inverse−variance weighted method
Inverse variance weighted (multiplicative random effects)
MR Egger
Robust adjusted profile score (RAPS)
Weighted median
SNP effect on Prostate cancer
SNP effect on LPA || id:ukb−bub35−LPA

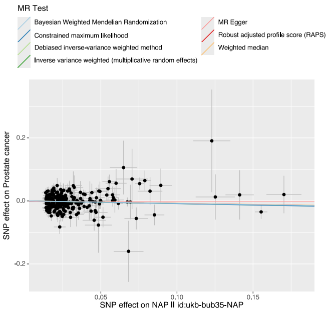
MR Test
Bayesian Weighted Mendelian Randomization
Constrained maximum likelihood
Debiased inverse-variance weighted method
Inverse variance weighted (multiplicative random effects)
MR Egger
Robust adjusted profile score (RAPS)
Weighted median
SNP effect on Prostate cancer
SNP effect on NAP || id:ukb-bub35-NAP

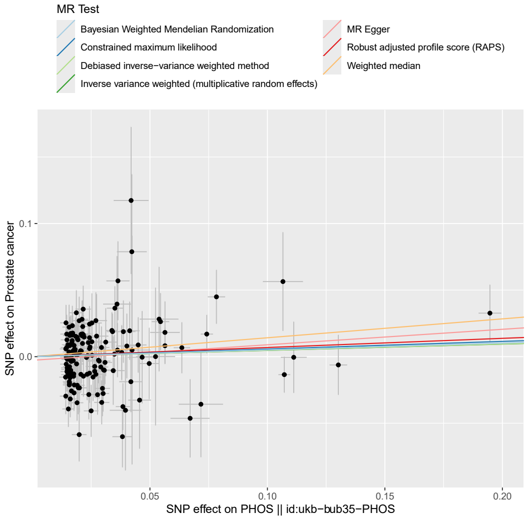
MR Test
Bayesian Weighted Mendelian Randomization
Constrained maximum likelihood
Debiased inverse−variance weighted method
Inverse variance weighted (multiplicative random effects)
MR Egger
Robust adjusted profile score (RAPS)
Weighted median
SNP effect on Prostate cancer
SNP effect on PHOS || id:ukb−bub35−PHOS

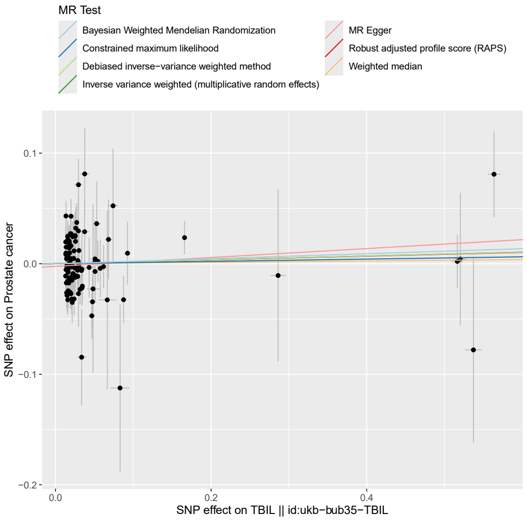
MR Test
Bayesian Weighted Mendelian Randomization
Constrained maximum likelihood
Debiased inverse−variance weighted method
Inverse variance weighted (multiplicative random effects)
MR Egger
Robust adjusted profile score (RAPS)
Weighted median
SNP effect on Prostate cancer
SNP effect on TBIL || id:ukb−bub35−TBIL

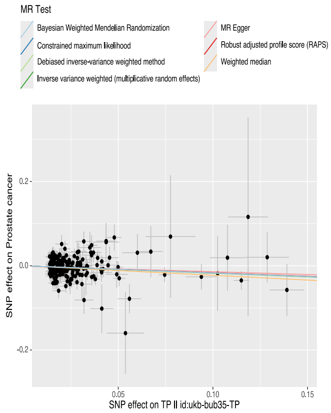
MR Test
Bayesian Weighted Mendelian Randomization
Constrained maximum likelihood
Debiased inverse-variance weighted method
Inverse variance weighted (multiplicative random effects)
MR Egger
Robust adjusted profile score (RAPS)
Weighted median
SNP effect on Prostate cancer
SNP effect on TP || id:ukb-bub35-TP

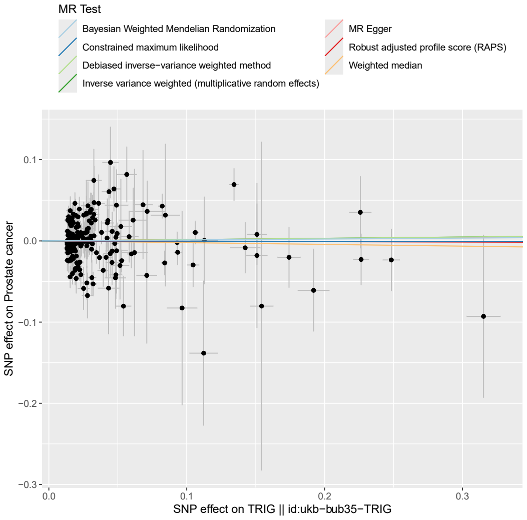
MR Test
Bayesian Weighted Mendelian Randomization
Constrained maximum likelihood
Debiased inverse−variance weighted method
Inverse variance weighted (multiplicative random effects)
MR Egger
Robust adjusted profile score (RAPS)
Weighted median
SNP effect on Prostate cancer
SNP effect on TRIG || id:ukb−bub35−TRIG

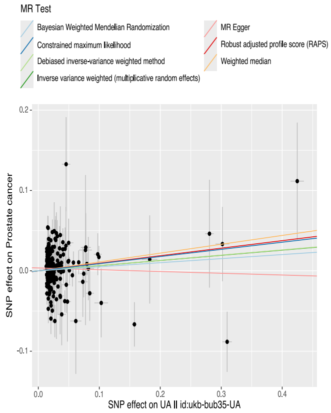
MR Test
Bayesian Weighted Mendelian Randomization
Constrained maximum likelihood
Debiased inverse-variance weighted method
Inverse variance weighted (multiplicative random effects)
MR Egger
Robust adjusted profile score (RAPS)
Weighted median
SNP effect on Prostate cancer
SNP effect on UA || id:ukb-bub35-UA

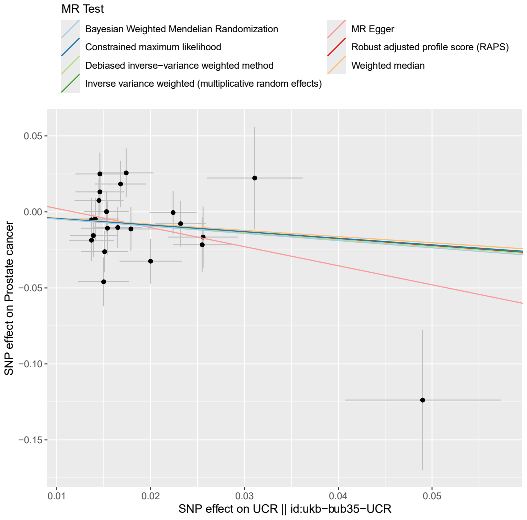
MR Test
Bayesian Weighted Mendelian Randomization
Constrained maximum likelihood
Debiased inverse−variance weighted method
Inverse variance weighted (multiplicative random effects)
MR Egger
Robust adjusted profile score (RAPS)
Weighted median
SNP effect on Prostate cancer
SNP effect on UCR || id:ukb−bub35−UCR

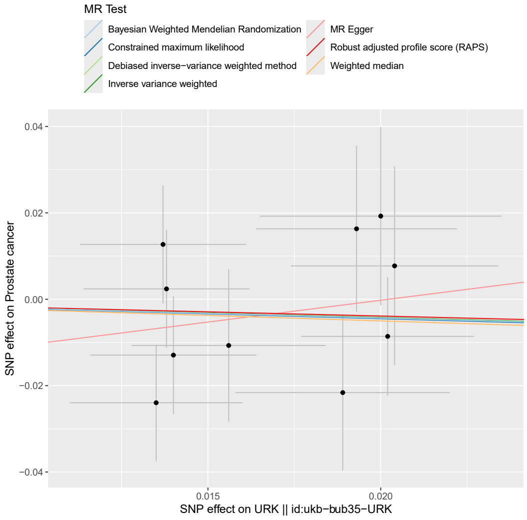
MR Test
Bayesian Weighted Mendelian Randomization
Constrained maximum likelihood
Debiased inverse−variance weighted method
Inverse variance weighted
MR Egger
Robust adjusted profile score (RAPS)
Weighted median
SNP effect on Prostate cancer
SNP effect on URK || id:ukb−bub35−URK

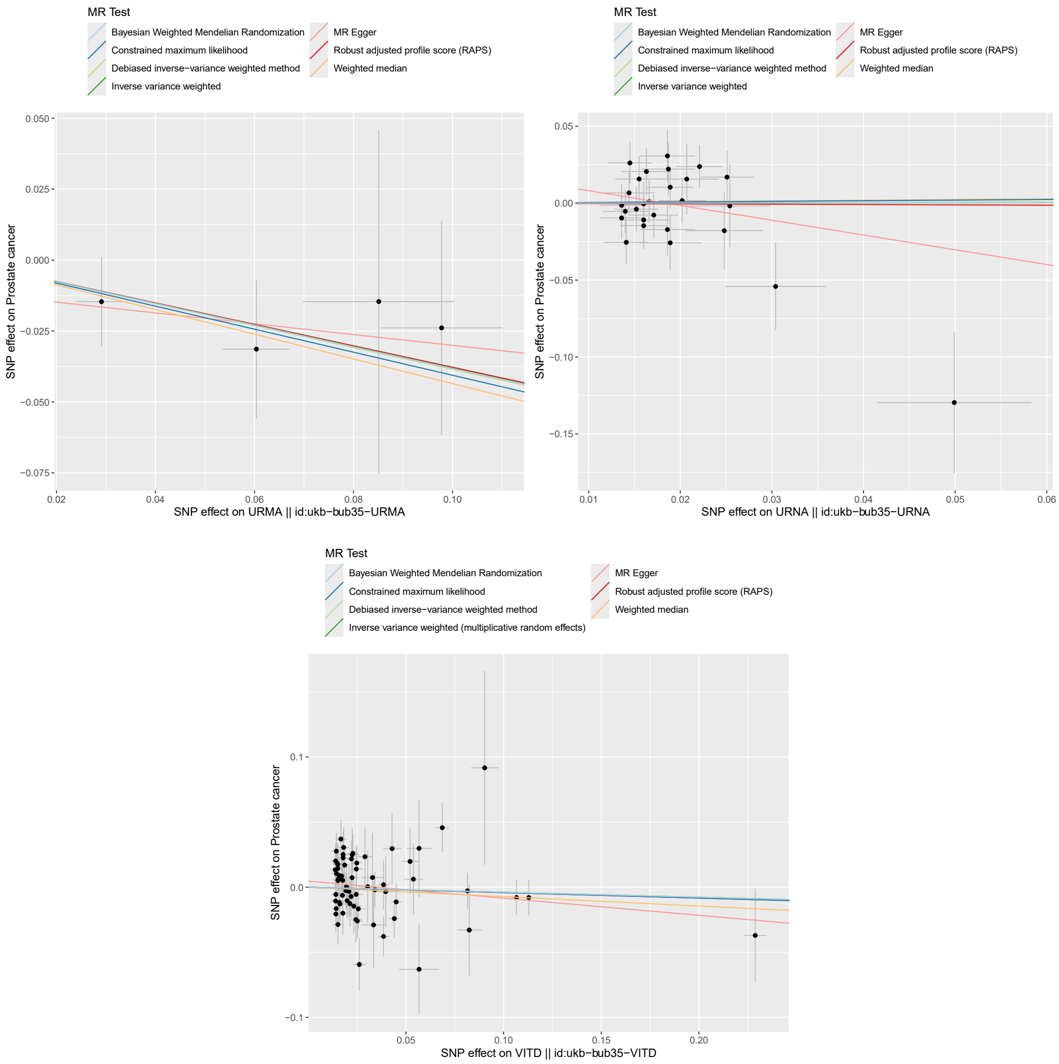

MR Test
Bayesian Weighted Mendelian Randomization
Constrained maximum likelihood
Debiased inverse−variance weighted method
Inverse variance weighted
MR Egger
Robust adjusted profile score (RAPS)
Weighted median
SNP effect on Prostate cancer
SNP effect on URMA || id:ukb−bub35−URMA
MR Test
Bayesian Weighted Mendelian Randomization
Constrained maximum likelihood
Debiased inverse−variance weighted method
Inverse variance weighted
MR Egger
Robust adjusted profile score (RAPS)
Weighted median
SNP effect on Prostate cancer
SNP effect on URNA || id:ukb−bub35−URNA
MR Test
Bayesian Weighted Mendelian Randomization
Constrained maximum likelihood
Debiased inverse−variance weighted method
Inverse variance weighted (multiplicative random effects)
MR Egger
Robust adjusted profile score (RAPS)
Weighted median
SNP effect on Prostate cancer
SNP effect on VITD || id:ukb−bub35−VITD

Scatter plot of RPCa

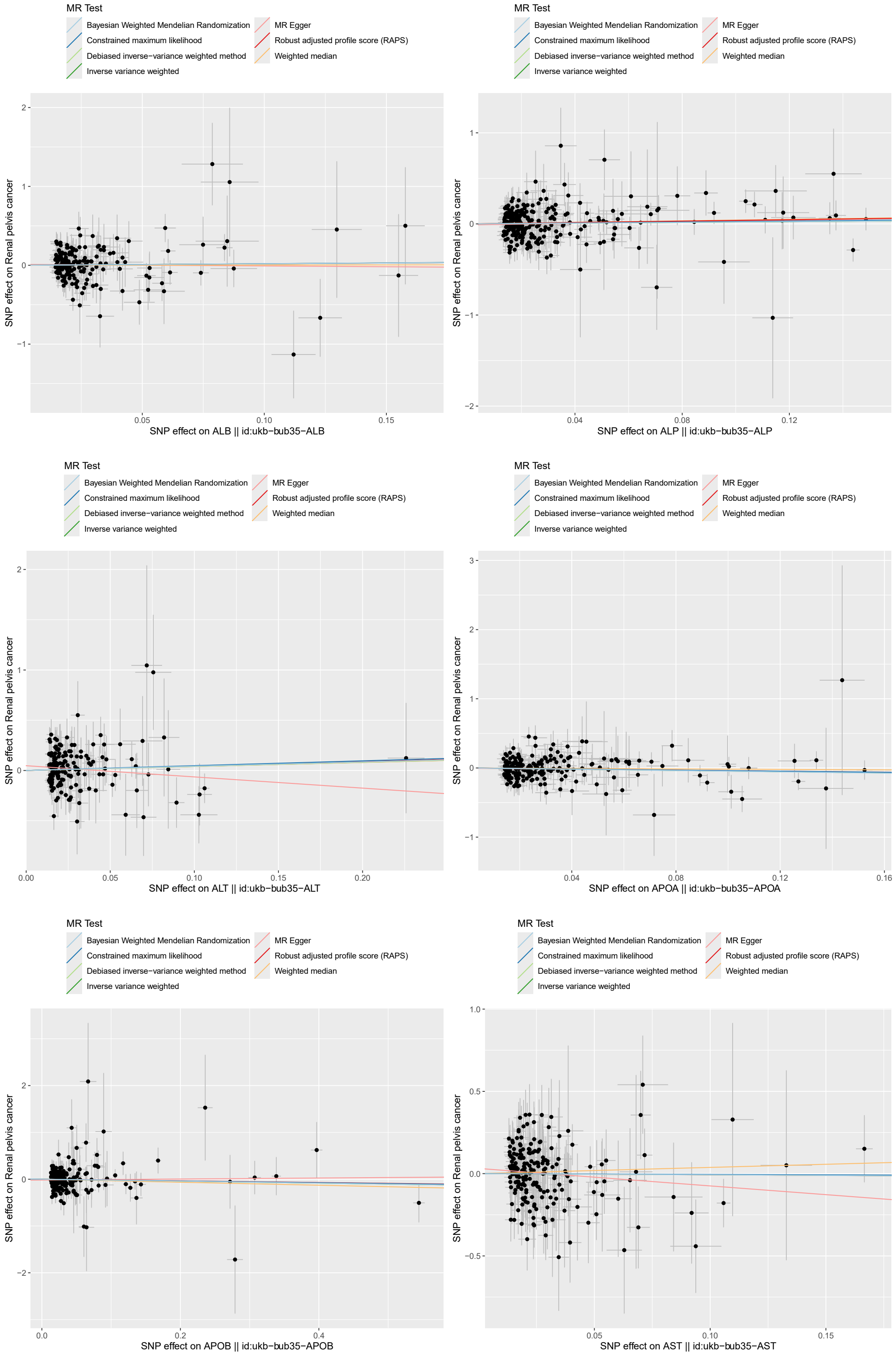

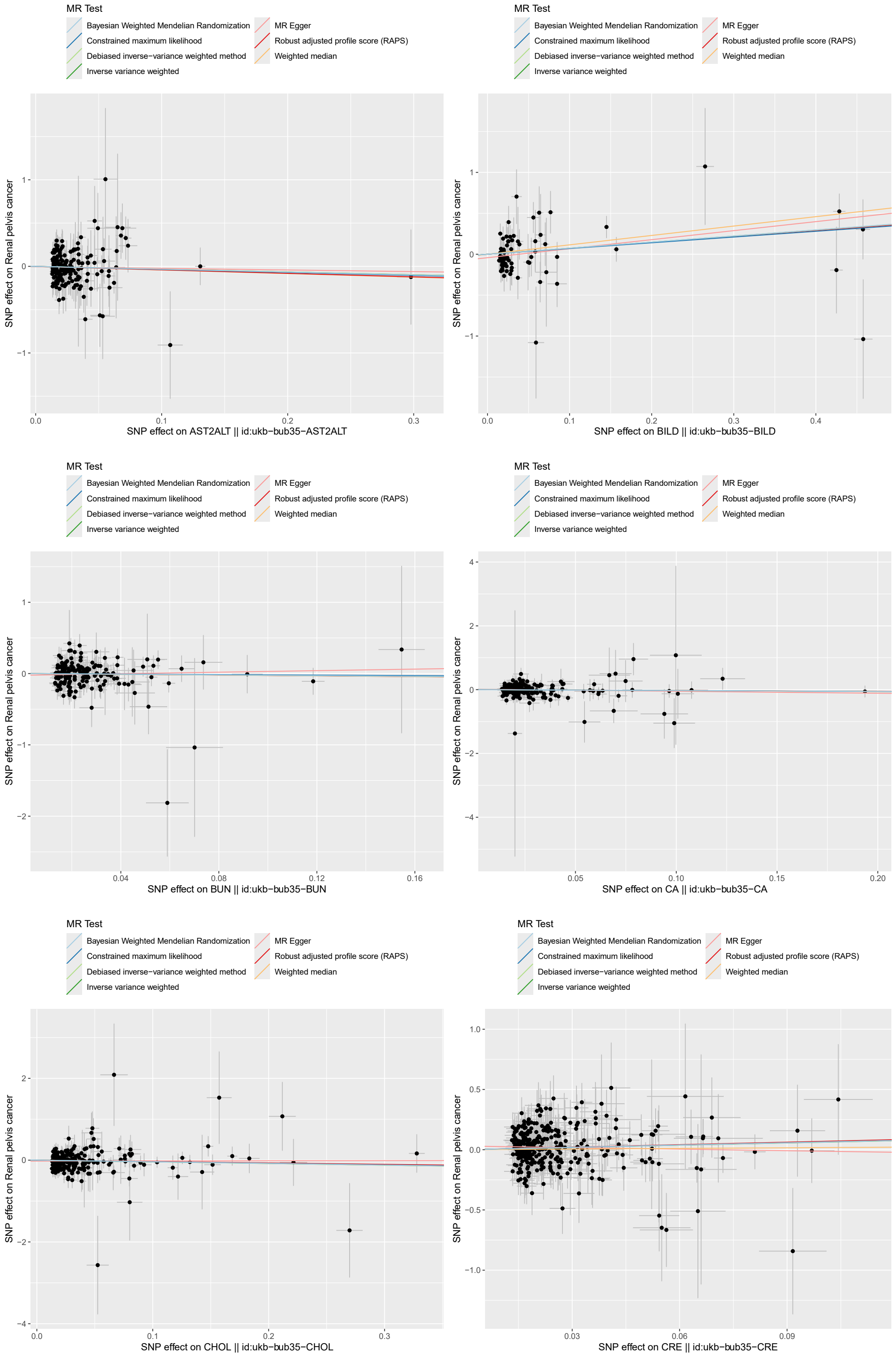
MR Test
Bayesian Weighted Mendelian Randomization
Constrained maximum likelihood
Debiased inverse−variance weighted method
Inverse variance weighted
MR Egger
Robust adjusted profile score (RAPS)
Weighted median
SNP effect on Renal pelvis cancer
SNP effect on AST2ALT || id:ukb−bub35−AST2ALT
SNP effect on BILD || id:ukb−bub35−BILD
SNP effect on BUN || id:ukb−bub35−BUN
SNP effect on CA || id:ukb−bub35−CA
SNP effect on CHOL || id:ukb−bub35−CHOL
SNP effect on CRE || id:ukb−bub35−CRE

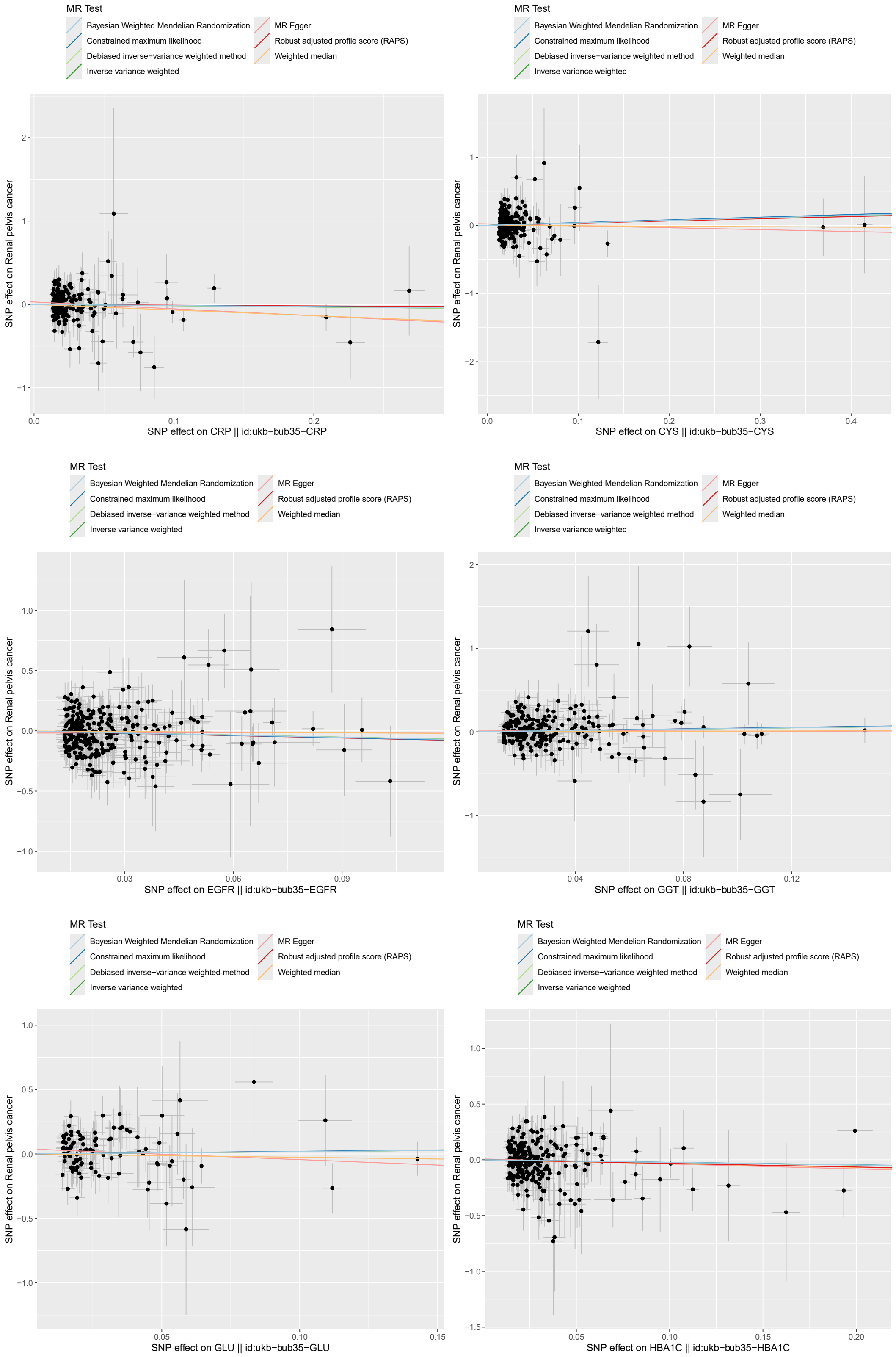
MR Test
Bayesian Weighted Mendelian Randomization
Constrained maximum likelihood
Debiased inverse−variance weighted method
Inverse variance weighted
MR Egger
Robust adjusted profile score (RAPS)
Weighted median
SNP effect on Renal pelvis cancer
SNP effect on CRP || id:ukb−bub35−CRP
SNP effect on CYS || id:ukb−bub35−CYS
SNP effect on EGFR || id:ukb−bub35−EGFR
SNP effect on GGT || id:ukb−bub35−GGT
SNP effect on GLU || id:ukb−bub35−GLU
SNP effect on HBA1C || id:ukb−bub35−HBA1C

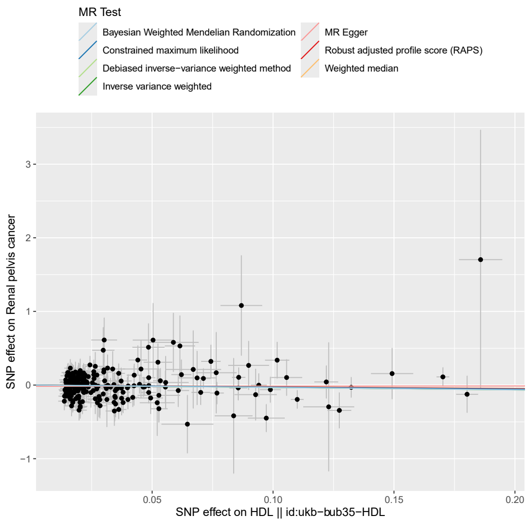
MR Test
Bayesian Weighted Mendelian Randomization
Constrained maximum likelihood
Debiased inverse−variance weighted method
Inverse variance weighted
MR Egger
Robust adjusted profile score (RAPS)
Weighted median
SNP effect on Renal pelvis cancer
SNP effect on HDL || id:ukb−bub35−HDL
3
2
1
0
−1
0.05
0.10
0.15
0.20

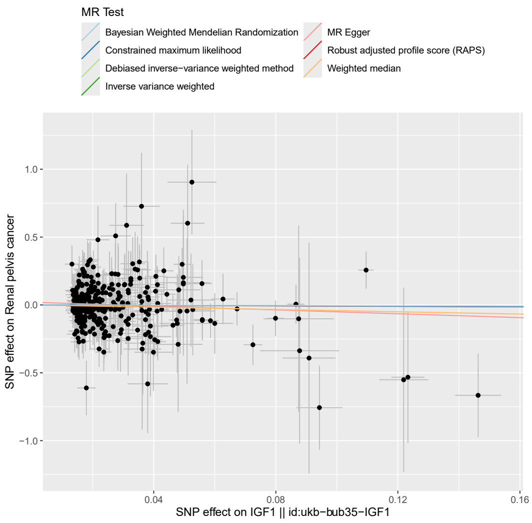
MR Test
Bayesian Weighted Mendelian Randomization
Constrained maximum likelihood
Debiased inverse−variance weighted method
Inverse variance weighted
MR Egger
Robust adjusted profile score (RAPS)
Weighted median
SNP effect on Renal pelvis cancer
SNP effect on IGF1 || id:ukb−bub35−IGF1
1.0
0.5
0.0
−0.5
−1.0
0.04
0.08
0.12
0.16

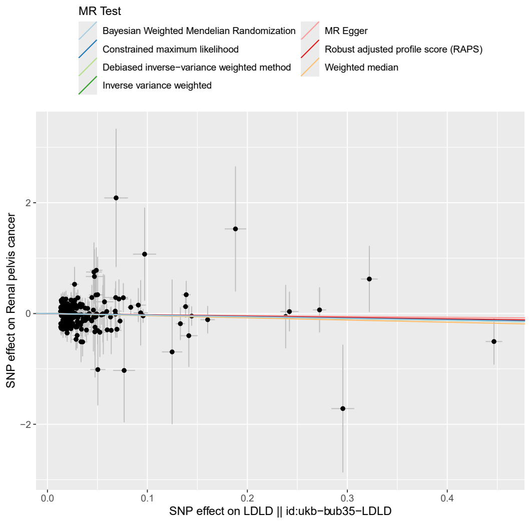
MR Test
Bayesian Weighted Mendelian Randomization
Constrained maximum likelihood
Debiased inverse−variance weighted method
Inverse variance weighted
MR Egger
Robust adjusted profile score (RAPS)
Weighted median
SNP effect on Renal pelvis cancer
SNP effect on LDLD || id:ukb−bub35−LDLD
2
0
−2
0.0
0.1
0.2
0.3
0.4

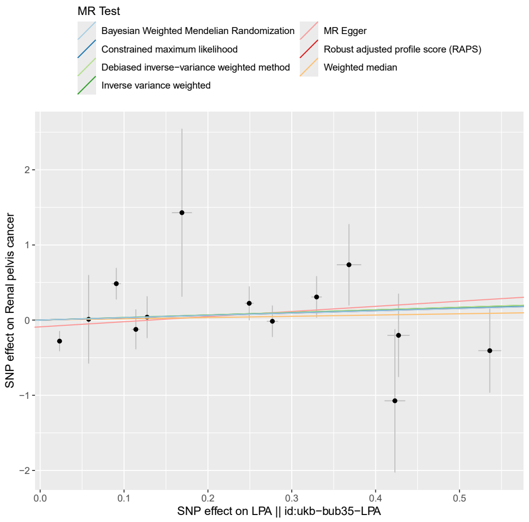
MR Test
Bayesian Weighted Mendelian Randomization
Constrained maximum likelihood
Debiased inverse−variance weighted method
Inverse variance weighted
MR Egger
Robust adjusted profile score (RAPS)
Weighted median
SNP effect on Renal pelvis cancer
SNP effect on LPA || id:ukb−bub35−LPA
2
1
0
−1
−2
0.0
0.1
0.2
0.3
0.4
0.5

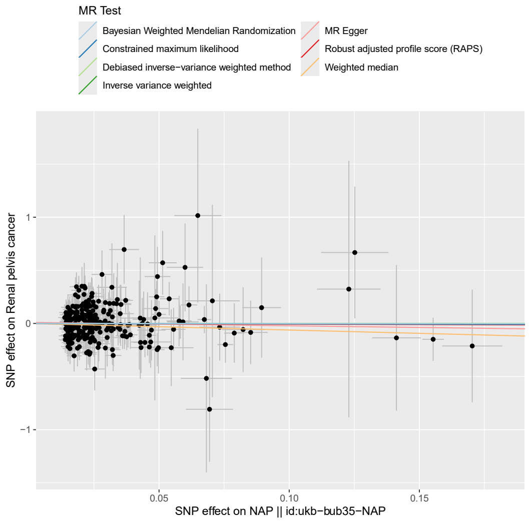
MR Test
Bayesian Weighted Mendelian Randomization
Constrained maximum likelihood
Debiased inverse−variance weighted method
Inverse variance weighted
MR Egger
Robust adjusted profile score (RAPS)
Weighted median
SNP effect on Renal pelvis cancer
SNP effect on NAP || id:ukb−bub35−NAP
1
0
−1
0.05
0.10
0.15

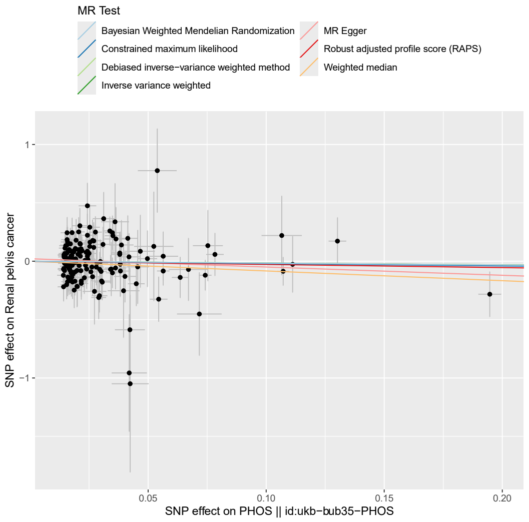
MR Test
Bayesian Weighted Mendelian Randomization
Constrained maximum likelihood
Debiased inverse−variance weighted method
Inverse variance weighted
MR Egger
Robust adjusted profile score (RAPS)
Weighted median
SNP effect on Renal pelvis cancer
SNP effect on PHOS || id:ukb−bub35−PHOS
1
0
−1
0.05
0.10
0.15
0.20

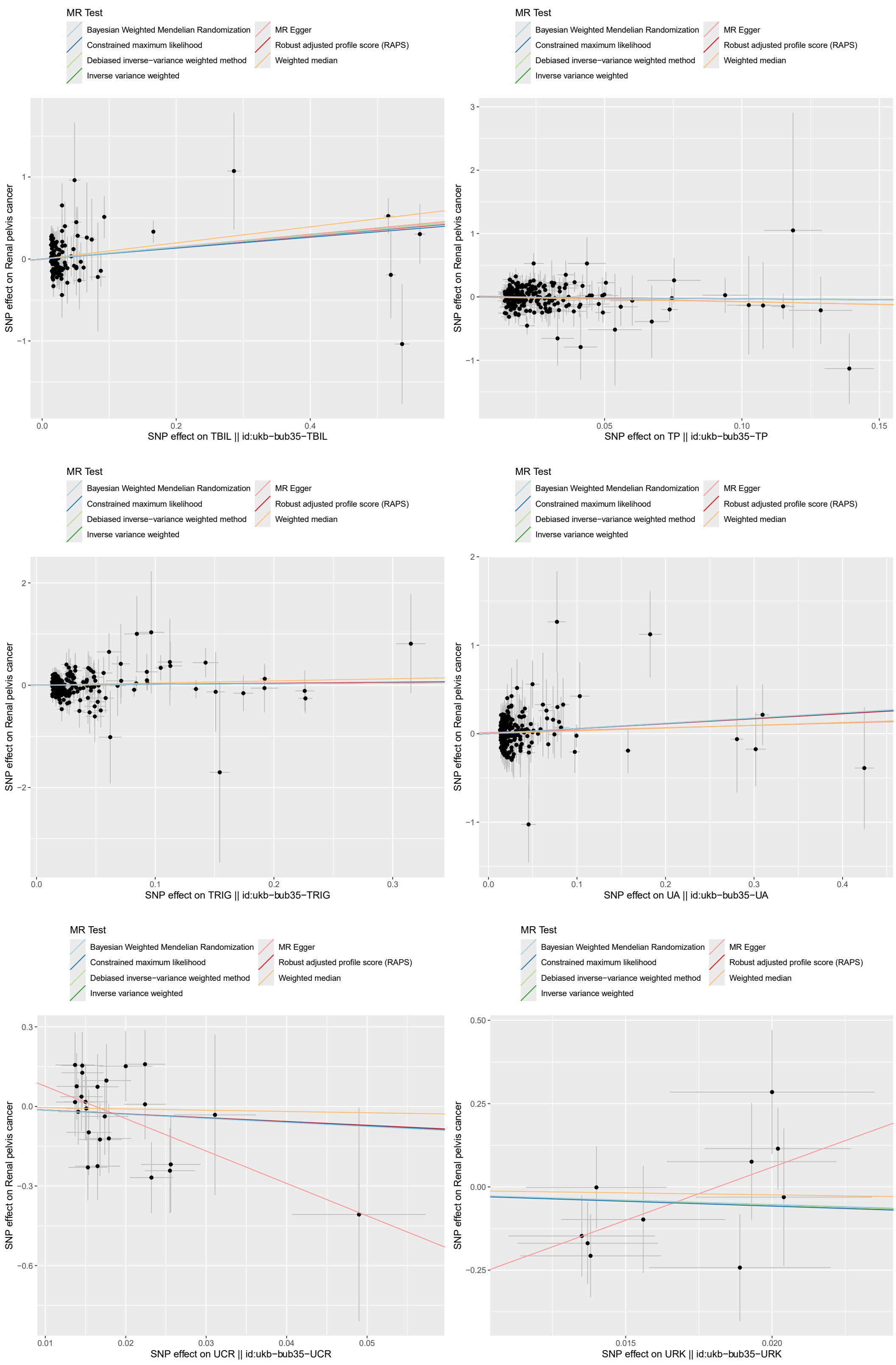

MR Test
Bayesian Weighted Mendelian Randomization
Constrained maximum likelihood
Debiased inverse−variance weighted method
Inverse variance weighted
MR Egger
Robust adjusted profile score (RAPS)
Weighted median
SNP effect on Renal pelvis cancer
1
0
−1
0.0
0.2
0.4
SNP effect on TBIL || id:ukb−bub35−TBIL
MR Test
Bayesian Weighted Mendelian Randomization
Constrained maximum likelihood
Debiased inverse−variance weighted method
Inverse variance weighted
MR Egger
Robust adjusted profile score (RAPS)
Weighted median
SNP effect on Renal pelvis cancer
3
2
1
0
−1
0.05
0.10
0.15
SNP effect on TP || id:ukb−bub35−TP
MR Test
Bayesian Weighted Mendelian Randomization
Constrained maximum likelihood
Debiased inverse−variance weighted method
Inverse variance weighted
MR Egger
Robust adjusted profile score (RAPS)
Weighted median
SNP effect on Renal pelvis cancer
2
0
−2
0.0
0.1
0.2
0.3
SNP effect on TRIG || id:ukb−bub35−TRIG
MR Test
Bayesian Weighted Mendelian Randomization
Constrained maximum likelihood
Debiased inverse−variance weighted method
Inverse variance weighted
MR Egger
Robust adjusted profile score (RAPS)
Weighted median
SNP effect on Renal pelvis cancer
2
1
0
−1
0.0
0.1
0.2
0.3
0.4
SNP effect on UA || id:ukb−bub35−UA
MR Test
Bayesian Weighted Mendelian Randomization
Constrained maximum likelihood
Debiased inverse−variance weighted method
Inverse variance weighted
MR Egger
Robust adjusted profile score (RAPS)
Weighted median
SNP effect on Renal pelvis cancer
0.3
0.0
−0.3
−0.6
0.01
0.02
0.03
0.04
0.05
SNP effect on UCR || id:ukb−bub35−UCR
MR Test
Bayesian Weighted Mendelian Randomization
Constrained maximum likelihood
Debiased inverse−variance weighted method
Inverse variance weighted
MR Egger
Robust adjusted profile score (RAPS)
Weighted median
SNP effect on Renal pelvis cancer
0.50
0.25
0.00
−0.25
0.015
0.020
SNP effect on URK || id:ukb−bub35−URK

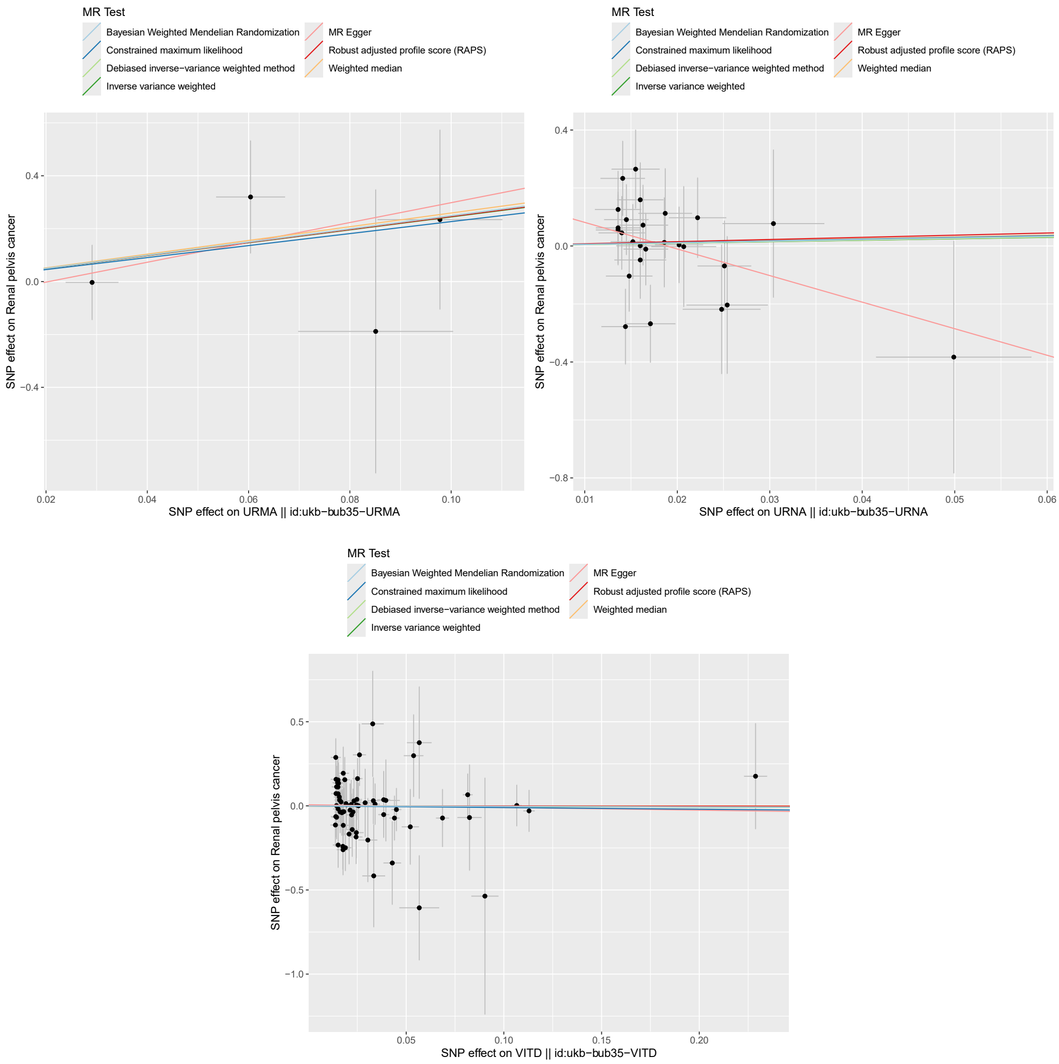
MR Test
Bayesian Weighted Mendelian Randomization
Constrained maximum likelihood
Debiased inverse−variance weighted method
Inverse variance weighted
MR Egger
Robust adjusted profile score (RAPS)
Weighted median
SNP effect on Renal pelvis cancer
0.4
0.0
−0.4
0.02
0.04
0.06
0.08
0.10
SNP effect on URMA || id:ukb−bub35−URMA
MR Test
Bayesian Weighted Mendelian Randomization
Constrained maximum likelihood
Debiased inverse−variance weighted method
Inverse variance weighted
MR Egger
Robust adjusted profile score (RAPS)
Weighted median
SNP effect on Renal pelvis cancer
0.4
0.0
−0.4
−0.8
0.01
0.02
0.03
0.04
0.05
0.06
SNP effect on URNA || id:ukb−bub35−URNA
MR Test
Bayesian Weighted Mendelian Randomization
Constrained maximum likelihood
Debiased inverse−variance weighted method
Inverse variance weighted
MR Egger
Robust adjusted profile score (RAPS)
Weighted median
SNP effect on Renal pelvis cancer
0.5
0.0
−0.5
−1.0
0.05
0.10
0.15
0.20
SNP effect on VITD || id:ukb−bub35−VITD

Scatter plot of KCa

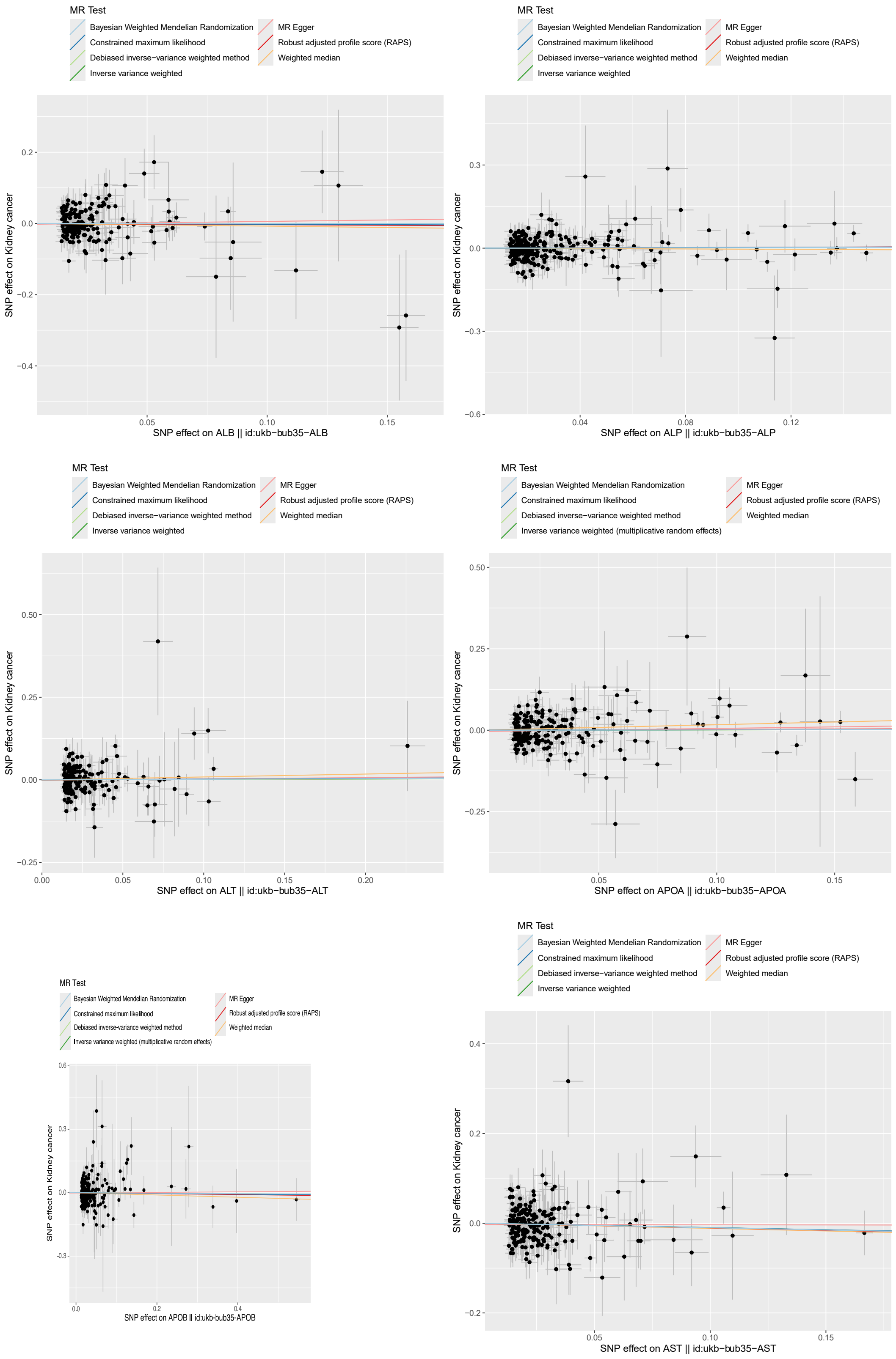

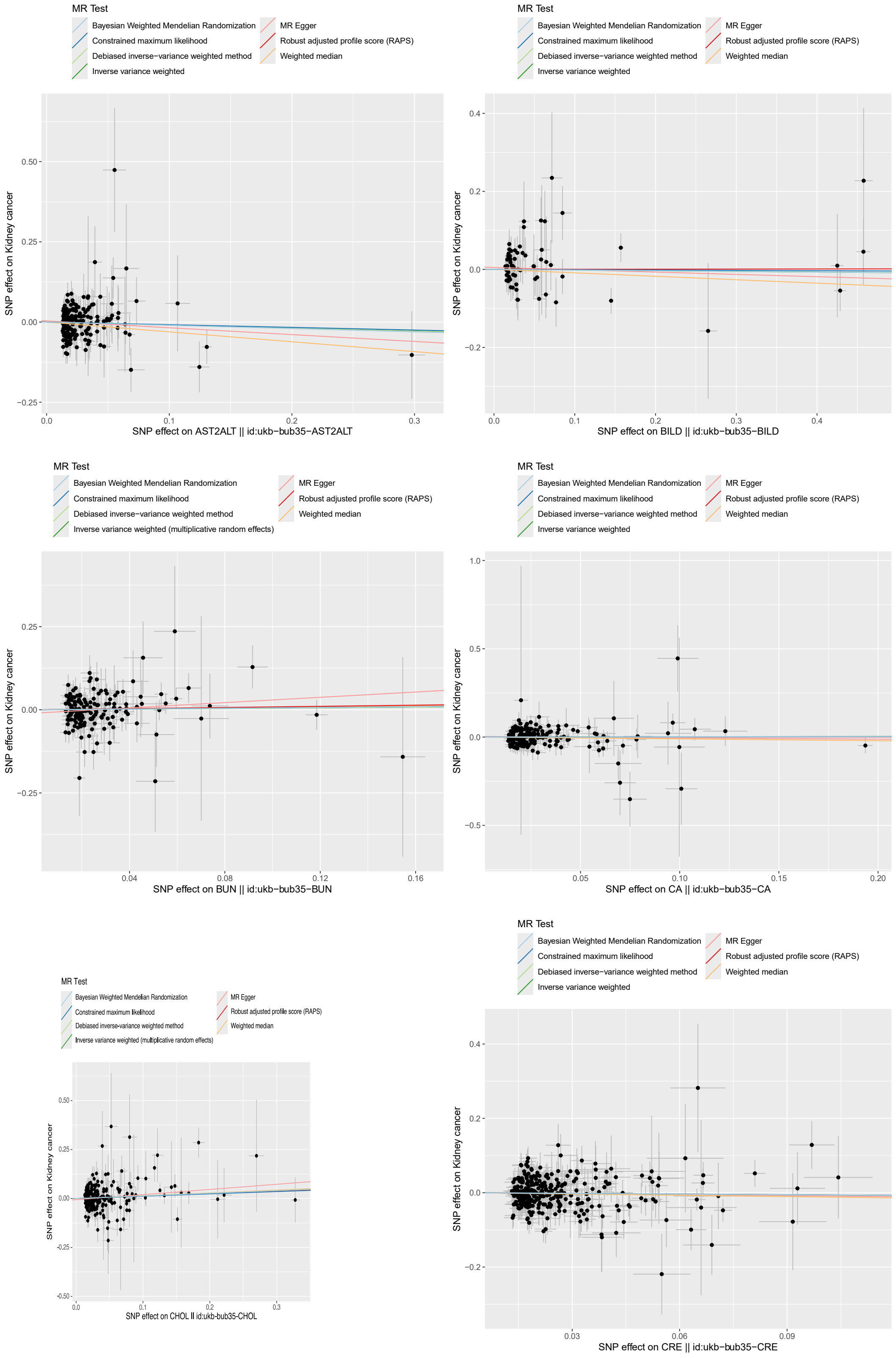
MR Test
Bayesian Weighted Mendelian Randomization
Constrained maximum likelihood
Debiased inverse−variance weighted method
Inverse variance weighted
MR Egger
Robust adjusted profile score (RAPS)
Weighted median
SNP effect on Kidney cancer
SNP effect on AST2ALT || id:ukb−bub35−AST2ALT
SNP effect on BILD || id:ukb−bub35−BILD
Inverse variance weighted (multiplicative random effects)
SNP effect on BUN || id:ukb−bub35−BUN
SNP effect on CA || id:ukb−bub35−CA
SNP effect on CHOL || id:ukb-bub35-CHOL
SNP effect on CRE || id:ukb−bub35−CRE

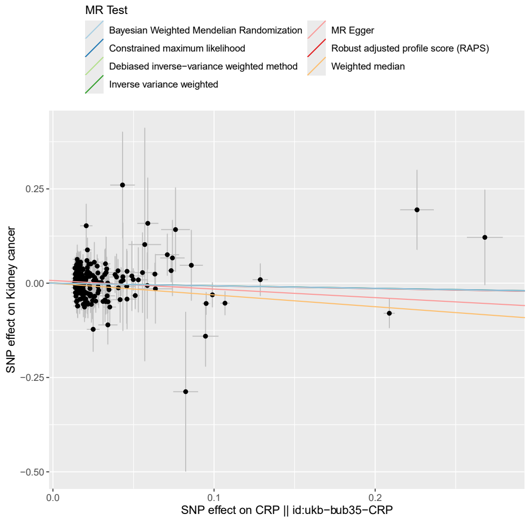
MR Test
Bayesian Weighted Mendelian Randomization
Constrained maximum likelihood
Debiased inverse−variance weighted method
Inverse variance weighted
MR Egger
Robust adjusted profile score (RAPS)
Weighted median
SNP effect on Kidney cancer
SNP effect on CRP || id:ukb−bub35−CRP

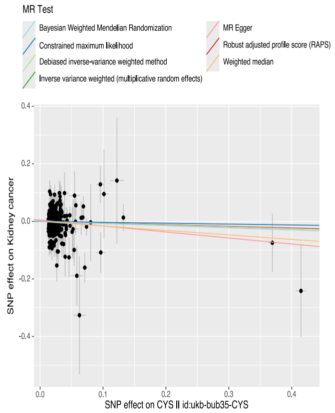
MR Test
Bayesian Weighted Mendelian Randomization
Constrained maximum likelihood
Debiased inverse-variance weighted method
Inverse variance weighted (multiplicative random effects)
MR Egger
Robust adjusted profile score (RAPS)
Weighted median
SNP effect on Kidney cancer
SNP effect on CYS || id:ukb-bub35-CYS

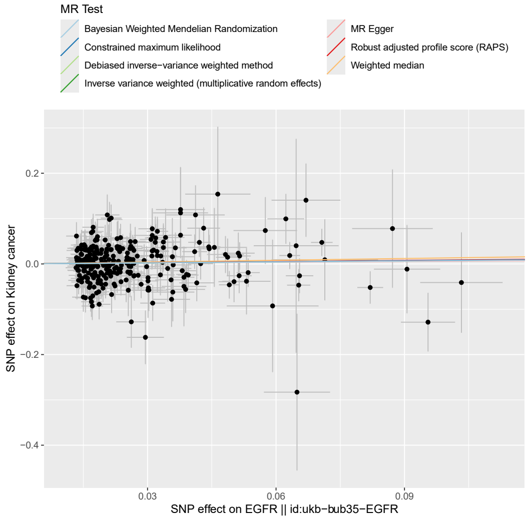
MR Test
Bayesian Weighted Mendelian Randomization
Constrained maximum likelihood
Debiased inverse−variance weighted method
Inverse variance weighted (multiplicative random effects)
MR Egger
Robust adjusted profile score (RAPS)
Weighted median
SNP effect on Kidney cancer
SNP effect on EGFR || id:ukb−bub35−EGFR

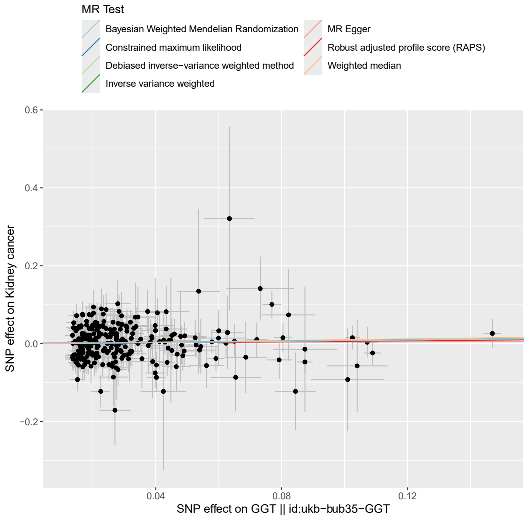
MR Test
Bayesian Weighted Mendelian Randomization
Constrained maximum likelihood
Debiased inverse−variance weighted method
Inverse variance weighted
MR Egger
Robust adjusted profile score (RAPS)
Weighted median
SNP effect on Kidney cancer
SNP effect on GGT || id:ukb−bub35−GGT

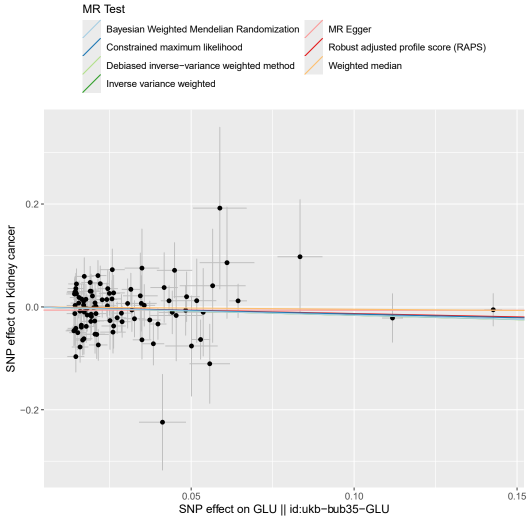
MR Test
Bayesian Weighted Mendelian Randomization
Constrained maximum likelihood
Debiased inverse−variance weighted method
Inverse variance weighted
MR Egger
Robust adjusted profile score (RAPS)
Weighted median
SNP effect on Kidney cancer
SNP effect on GLU || id:ukb−bub35−GLU

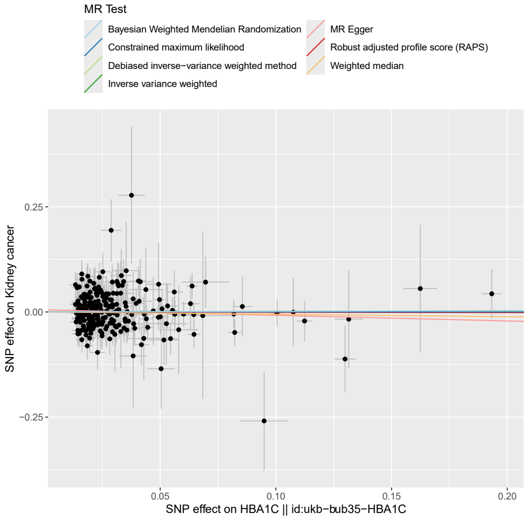
MR Test
Bayesian Weighted Mendelian Randomization
Constrained maximum likelihood
Debiased inverse−variance weighted method
Inverse variance weighted
MR Egger
Robust adjusted profile score (RAPS)
Weighted median
SNP effect on Kidney cancer
SNP effect on HBA1C || id:ukb−bub35−HBA1C

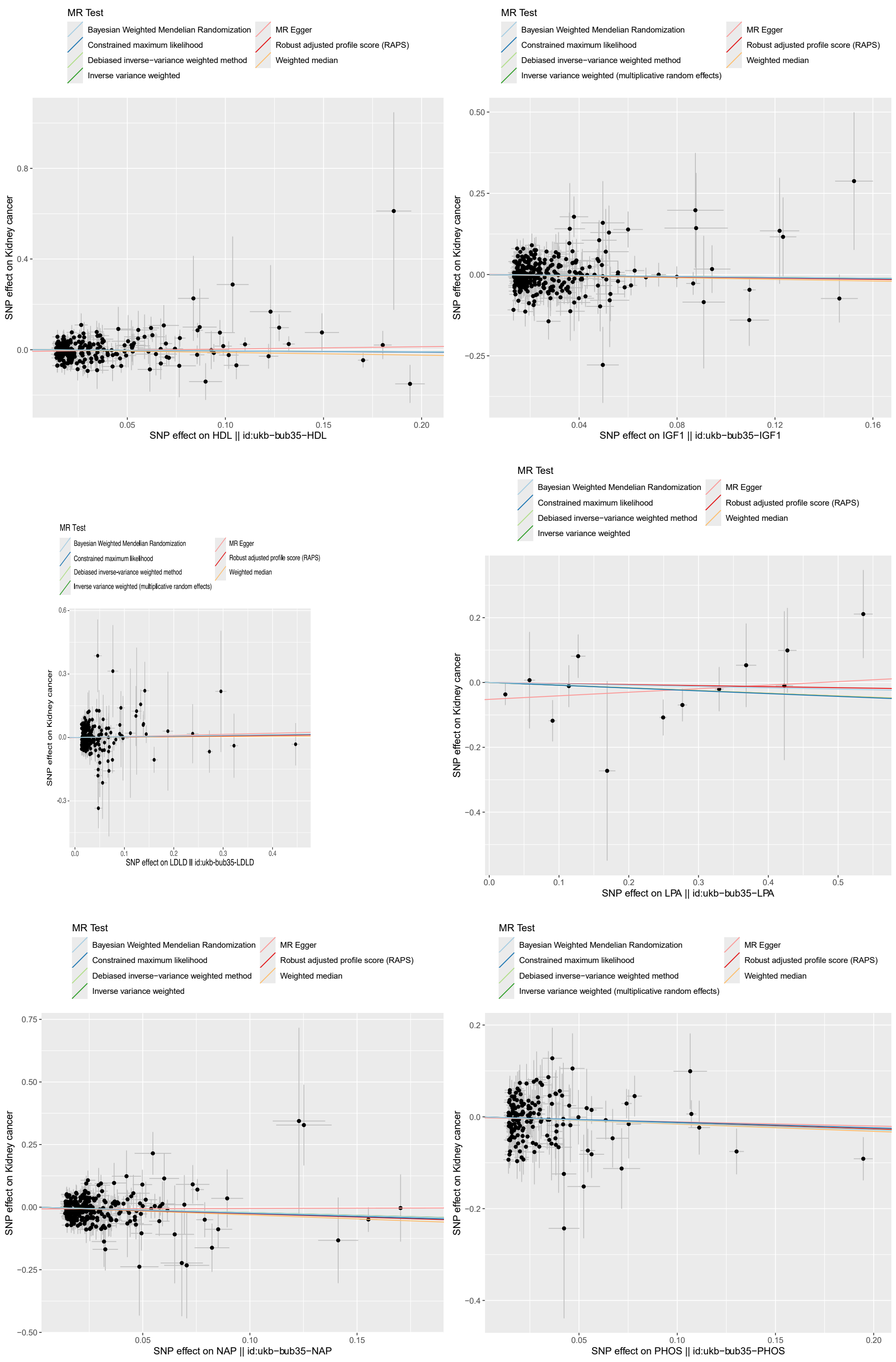
MR Test
Bayesian Weighted Mendelian Randomization
Constrained maximum likelihood
Debiased inverse−variance weighted method
Inverse variance weighted
MR Egger
Robust adjusted profile score (RAPS)
Weighted median
SNP effect on Kidney cancer
SNP effect on HDL || id:ukb−bub35−HDL
MR Test
Bayesian Weighted Mendelian Randomization
Constrained maximum likelihood
Debiased inverse−variance weighted method
Inverse variance weighted (multiplicative random effects)
MR Egger
Robust adjusted profile score (RAPS)
Weighted median
SNP effect on Kidney cancer
SNP effect on IGF1 || id:ukb−bub35−IGF1
MR Test
Bayesian Weighted Mendelian Randomization
Constrained maximum likelihood
Debiased inverse-variance weighted method
Inverse variance weighted (multiplicative random effects)
MR Egger
Robust adjusted profile score (RAPS)
Weighted median
SNP effect on Kidney cancer
SNP effect on LDLD || id:ukb-bub35-LDLD
MR Test
Bayesian Weighted Mendelian Randomization
Constrained maximum likelihood
Debiased inverse−variance weighted method
Inverse variance weighted
MR Egger
Robust adjusted profile score (RAPS)
Weighted median
SNP effect on Kidney cancer
SNP effect on LPA || id:ukb−bub35−LPA
MR Test
Bayesian Weighted Mendelian Randomization
Constrained maximum likelihood
Debiased inverse−variance weighted method
Inverse variance weighted
MR Egger
Robust adjusted profile score (RAPS)
Weighted median
SNP effect on Kidney cancer
SNP effect on NAP || id:ukb−bub35−NAP
MR Test
Bayesian Weighted Mendelian Randomization
Constrained maximum likelihood
Debiased inverse−variance weighted method
Inverse variance weighted (multiplicative random effects)
MR Egger
Robust adjusted profile score (RAPS)
Weighted median
SNP effect on Kidney cancer
SNP effect on PHOS || id:ukb−bub35−PHOS

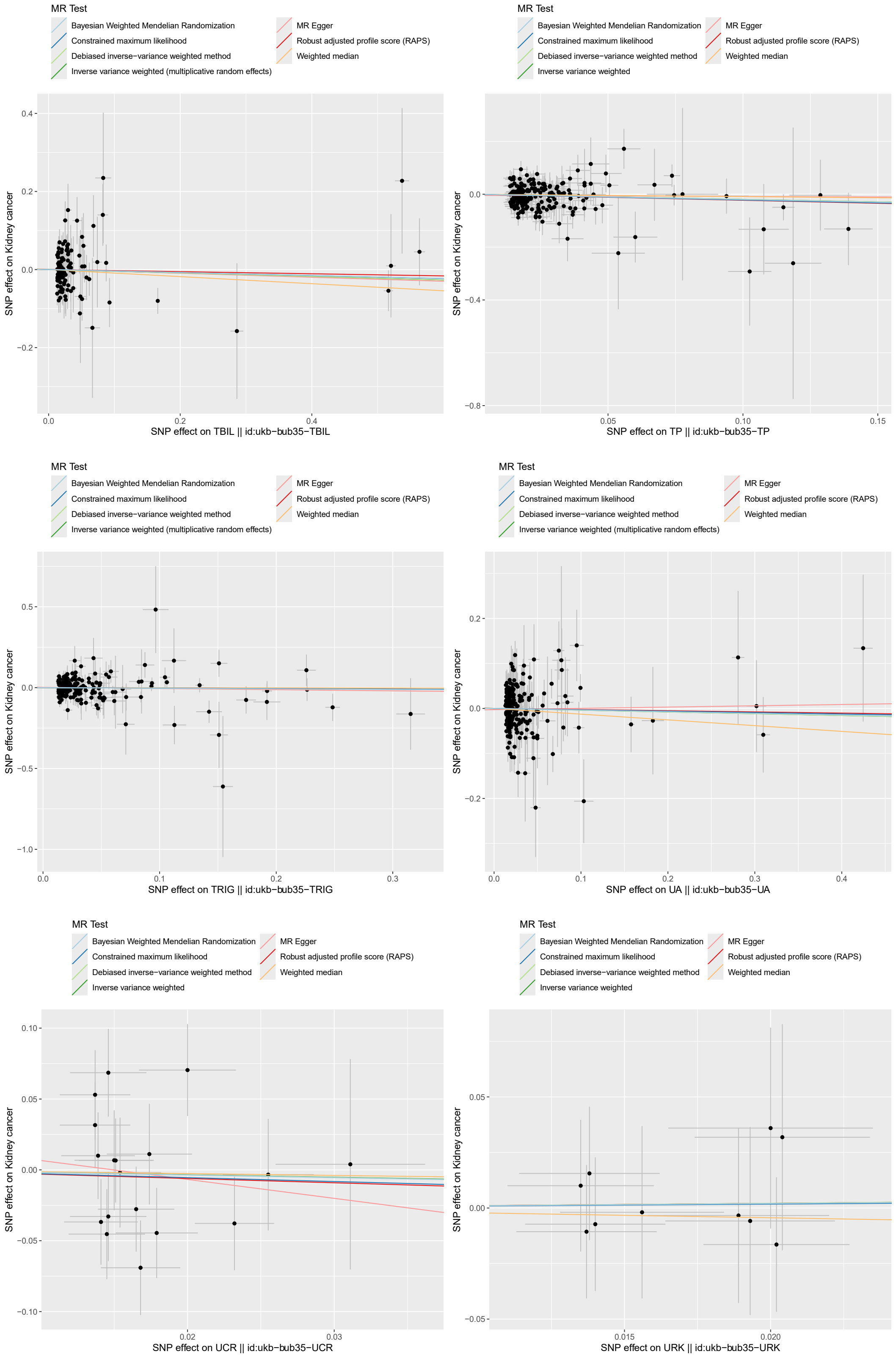

MR Test
Bayesian Weighted Mendelian Randomization
Constrained maximum likelihood
Debiased inverse−variance weighted method
Inverse variance weighted (multiplicative random effects)
MR Egger
Robust adjusted profile score (RAPS)
Weighted median
SNP effect on Kidney cancer
SNP effect on TBIL || id:ukb−bub35−TBIL
MR Test
Bayesian Weighted Mendelian Randomization
Constrained maximum likelihood
Debiased inverse−variance weighted method
Inverse variance weighted
MR Egger
Robust adjusted profile score (RAPS)
Weighted median
SNP effect on Kidney cancer
SNP effect on TP || id:ukb−bub35−TP
MR Test
Bayesian Weighted Mendelian Randomization
Constrained maximum likelihood
Debiased inverse−variance weighted method
Inverse variance weighted (multiplicative random effects)
MR Egger
Robust adjusted profile score (RAPS)
Weighted median
SNP effect on Kidney cancer
SNP effect on TRIG || id:ukb−bub35−TRIG
MR Test
Bayesian Weighted Mendelian Randomization
Constrained maximum likelihood
Debiased inverse−variance weighted method
Inverse variance weighted (multiplicative random effects)
MR Egger
Robust adjusted profile score (RAPS)
Weighted median
SNP effect on Kidney cancer
SNP effect on UA || id:ukb−bub35−UA
MR Test
Bayesian Weighted Mendelian Randomization
Constrained maximum likelihood
Debiased inverse−variance weighted method
Inverse variance weighted
MR Egger
Robust adjusted profile score (RAPS)
Weighted median
SNP effect on Kidney cancer
SNP effect on UCR || id:ukb−bub35−UCR
MR Test
Bayesian Weighted Mendelian Randomization
Constrained maximum likelihood
Debiased inverse−variance weighted method
Inverse variance weighted
MR Egger
Robust adjusted profile score (RAPS)
Weighted median
SNP effect on Kidney cancer
SNP effect on URK || id:ukb−bub35−URK

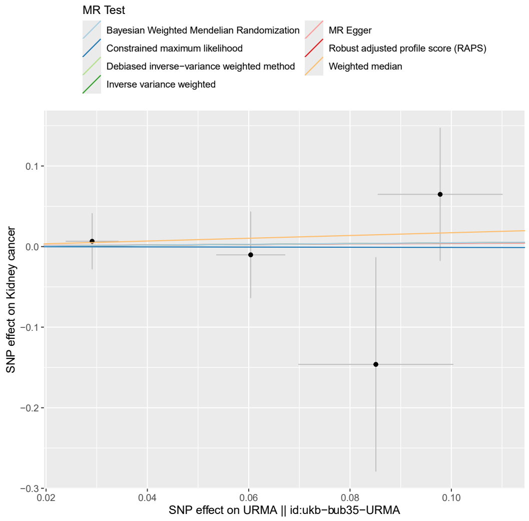
MR Test
Bayesian Weighted Mendelian Randomization
Constrained maximum likelihood
Debiased inverse−variance weighted method
Inverse variance weighted
MR Egger
Robust adjusted profile score (RAPS)
Weighted median
SNP effect on Kidney cancer
SNP effect on URMA || id:ukb−bub35−URMA

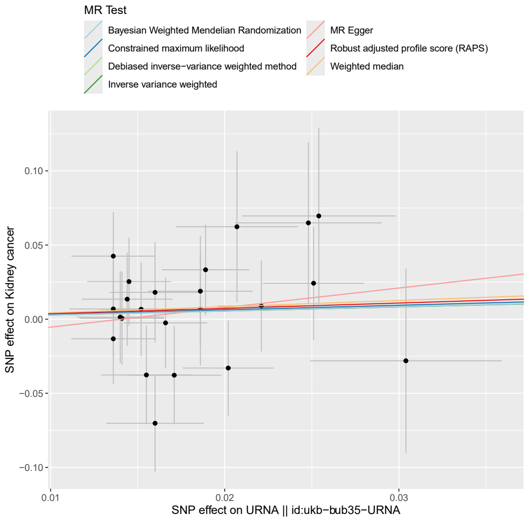
MR Test
Bayesian Weighted Mendelian Randomization
Constrained maximum likelihood
Debiased inverse−variance weighted method
Inverse variance weighted
MR Egger
Robust adjusted profile score (RAPS)
Weighted median
SNP effect on Kidney cancer
SNP effect on URNA || id:ukb−bub35−URNA

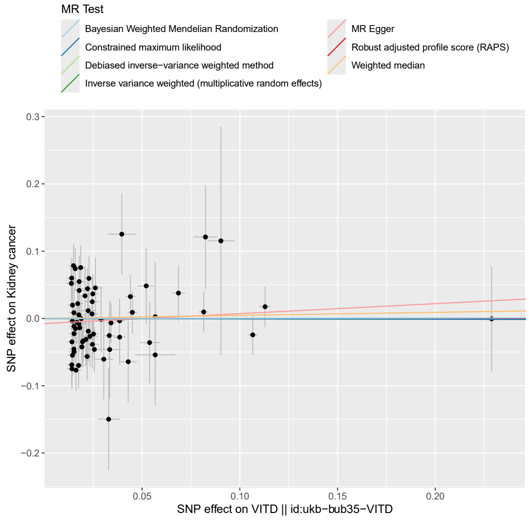
MR Test
Bayesian Weighted Mendelian Randomization
Constrained maximum likelihood
Debiased inverse−variance weighted method
Inverse variance weighted (multiplicative random effects)
MR Egger
Robust adjusted profile score (RAPS)
Weighted median
SNP effect on Kidney cancer
SNP effect on VITD || id:ukb−bub35−VITD

BCa's leave-one-out method

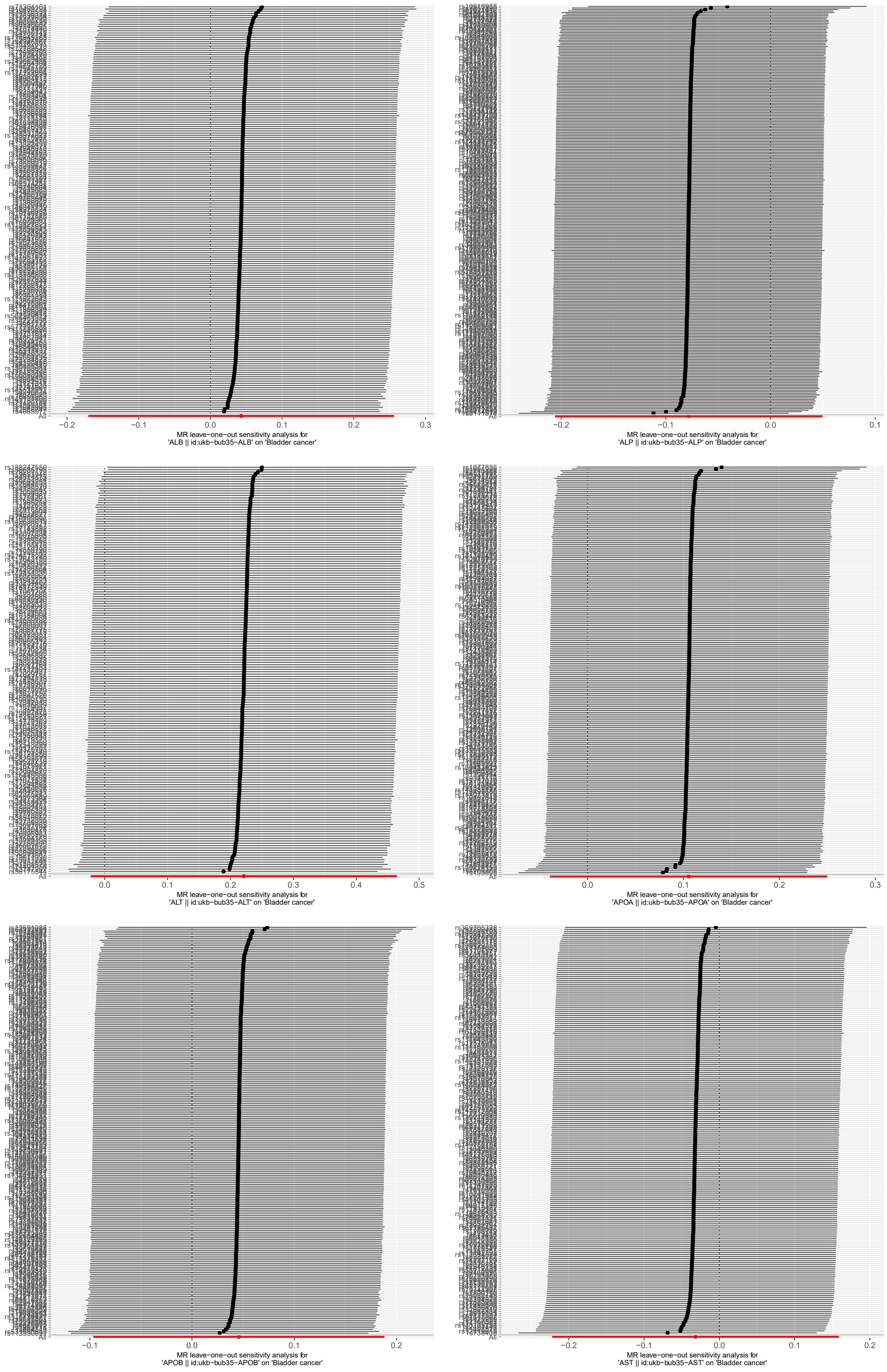

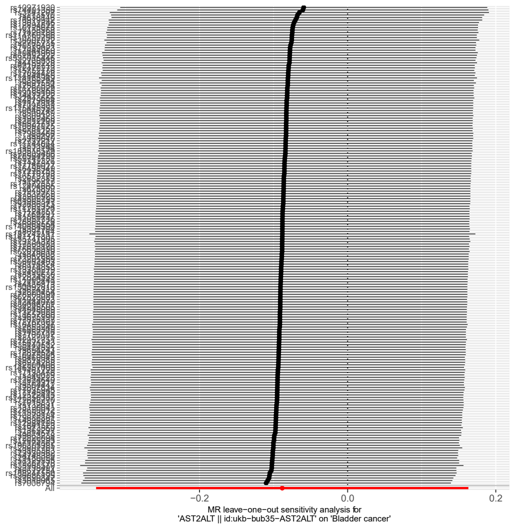
All
−0.2
0.0
0.2
MR leave−one−out sensitivity analysis for
'AST2ALT || id:ukb−bub35−AST2ALT' on 'Bladder cancer'

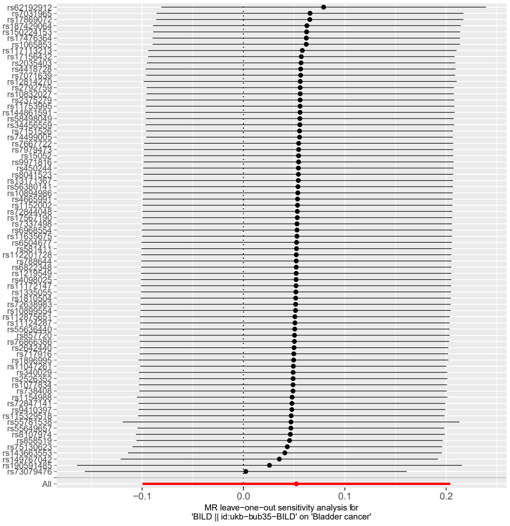
All
−0.1
0.0
0.1
0.2
MR leave−one−out sensitivity analysis for
'BILD || id:ukb−bub35−BILD' on 'Bladder cancer'

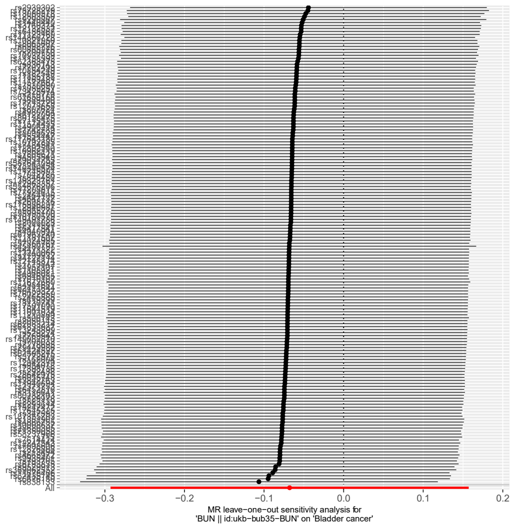
All
−0.3
−0.2
−0.1
0.0
0.1
0.2
MR leave−one−out sensitivity analysis for
'BUN || id:ukb−bub35−BUN' on 'Bladder cancer'

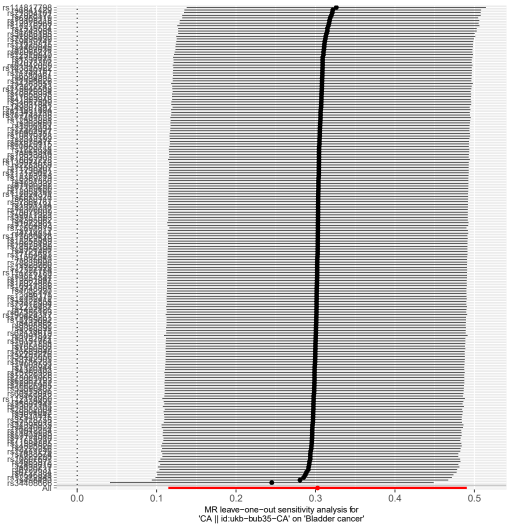
All
0.0
0.1
0.2
0.3
0.4
0.5
MR leave−one−out sensitivity analysis for
'CA || id:ukb−bub35−CA' on 'Bladder cancer'

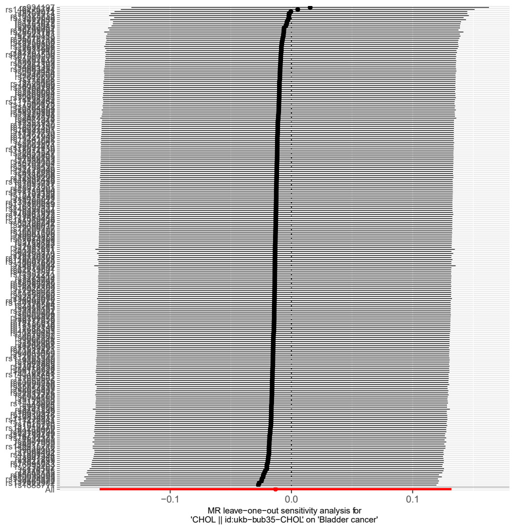
All
−0.1
0.0
0.1
MR leave−one−out sensitivity analysis for
'CHOL || id:ukb−bub35−CHOL' on 'Bladder cancer'

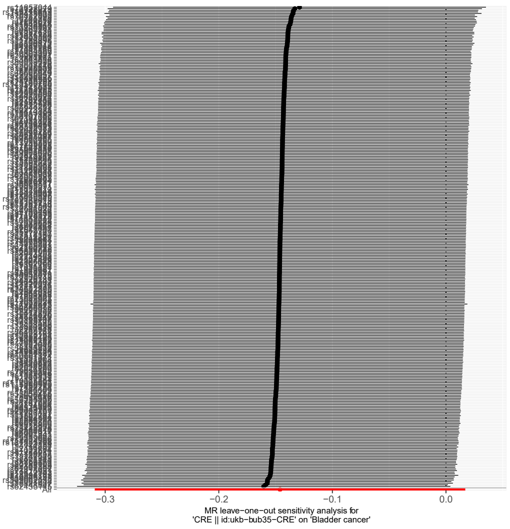
All
−0.3
−0.2
−0.1
0.0
MR leave−one−out sensitivity analysis for
'CRE || id:ukb−bub35−CRE' on 'Bladder cancer'

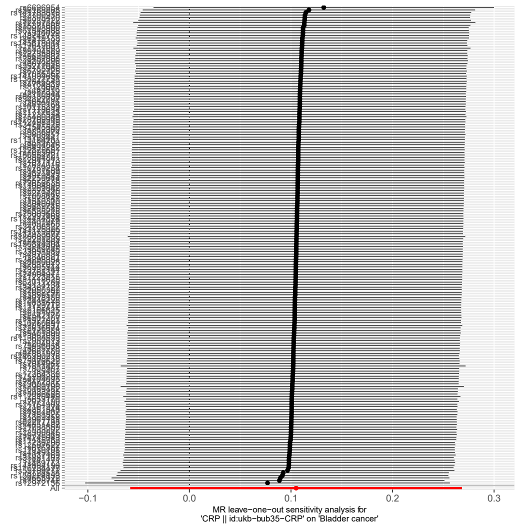
All
−0.1
0.0
0.1
0.2
0.3
MR leave−one−out sensitivity analysis for
'CRP || id:ukb−bub35−CRP' on 'Bladder cancer'

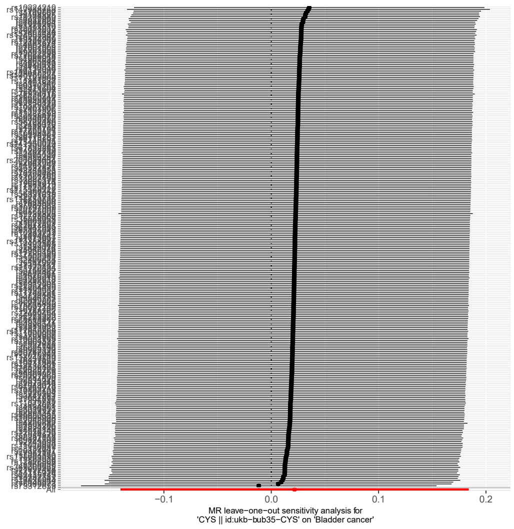
All
−0.1
0.0
0.1
0.2
MR leave−one−out sensitivity analysis for
'CYS || id:ukb−bub35−CYS' on 'Bladder cancer'

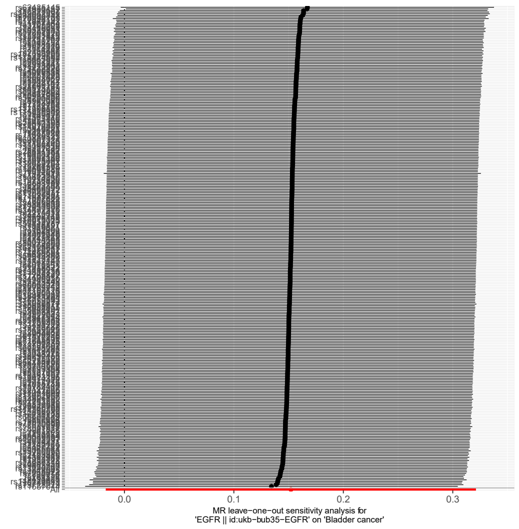
All
0.0
0.1
0.2
0.3
MR leave−one−out sensitivity analysis for
'EGFR || id:ukb−bub35−EGFR' on 'Bladder cancer'

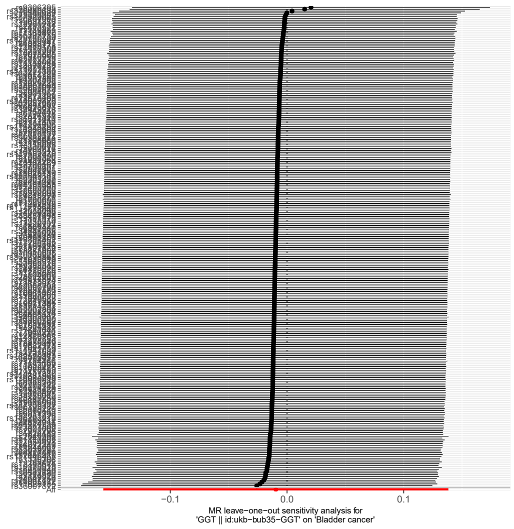
All
−0.1
0.0
0.1
MR leave−one−out sensitivity analysis for
'GGT || id:ukb−bub35−GGT' on 'Bladder cancer'

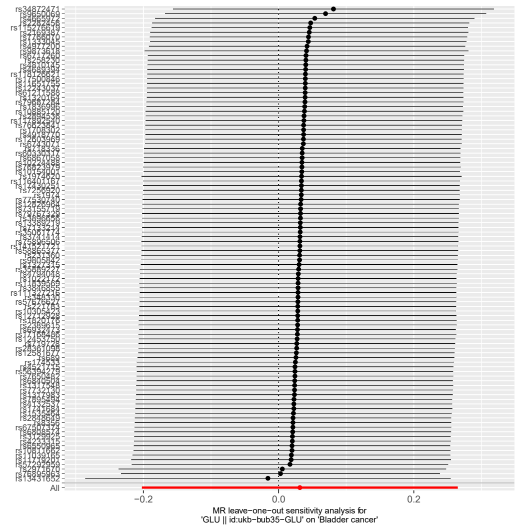
All
−0.2
0.0
0.2
MR leave−one−out sensitivity analysis for
'GLU || id:ukb−bub35−GLU' on 'Bladder cancer'

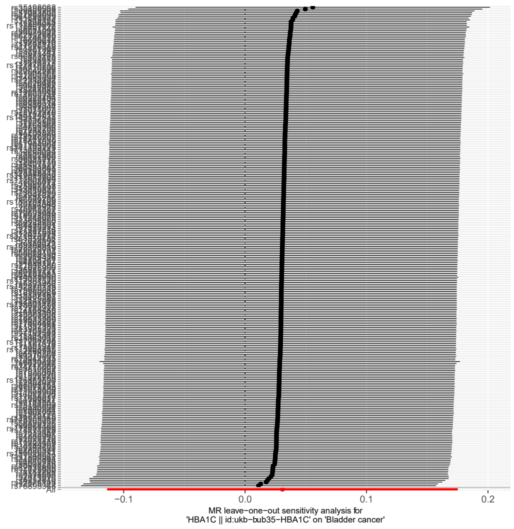
All
−0.1
0.0
0.1
0.2
MR leave−one−out sensitivity analysis for
'HBA1C || id:ukb−bub35−HBA1C' on 'Bladder cancer'

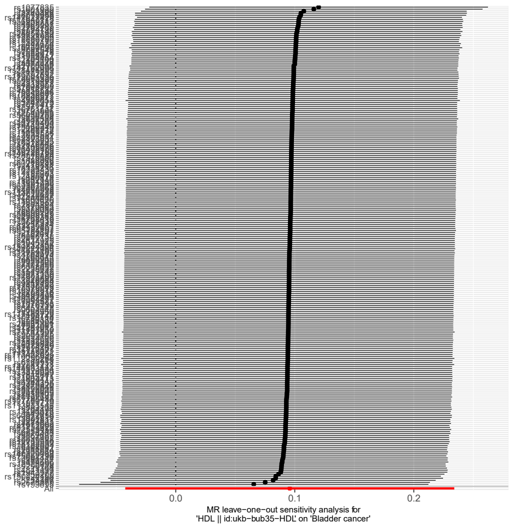
All
0.0
0.1
0.2
MR leave−one−out sensitivity analysis for
'HDL || id:ukb-bub35-HDL' on 'Bladder cancer'

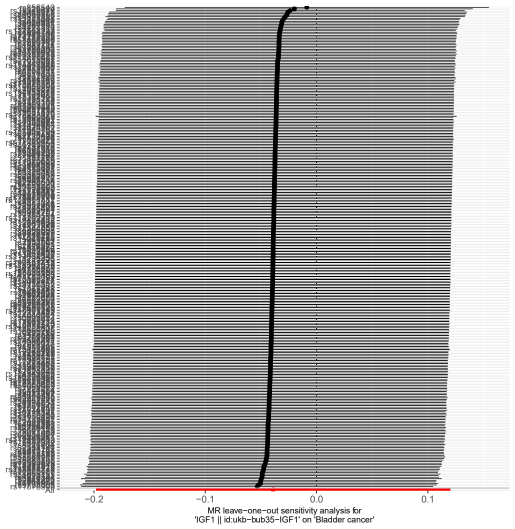
All
−0.2
−0.1
0.0
0.1
MR leave−one−out sensitivity analysis for
'IGF1 || id:ukb-bub35-IGF1' on 'Bladder cancer'

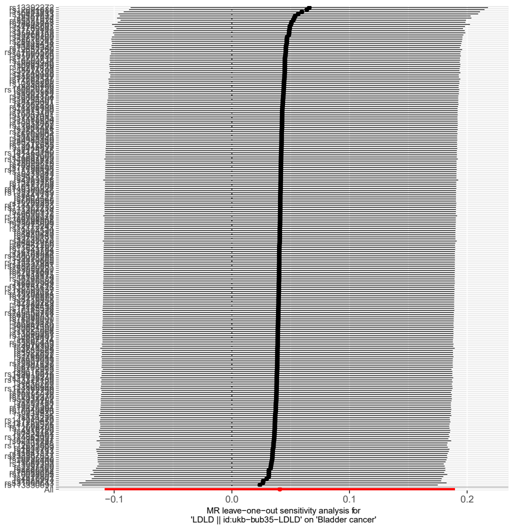
All
−0.1
0.0
0.1
0.2
MR leave−one−out sensitivity analysis for
'LDLD || id:ukb-bub35-LDLD' on 'Bladder cancer'

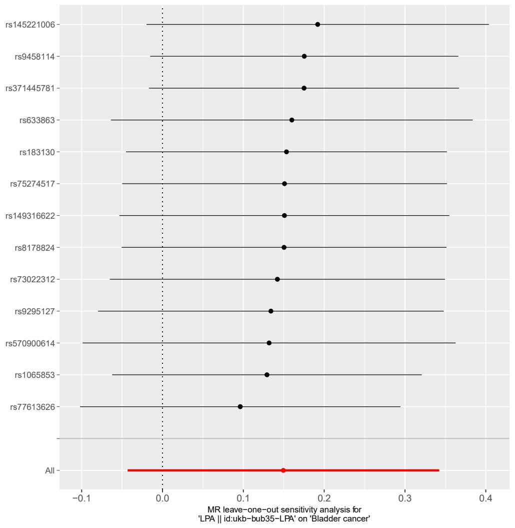
rs145221006
rs9458114
rs371445781
rs633863
rs183130
rs75274517
rs149316622
rs8178824
rs73022312
rs9295127
rs570900614
rs1065853
rs77613626
All
−0.1
0.0
0.1
0.2
0.3
0.4
MR leave−one−out sensitivity analysis for
'LPA || id:ukb-bub35-LPA' on 'Bladder cancer'

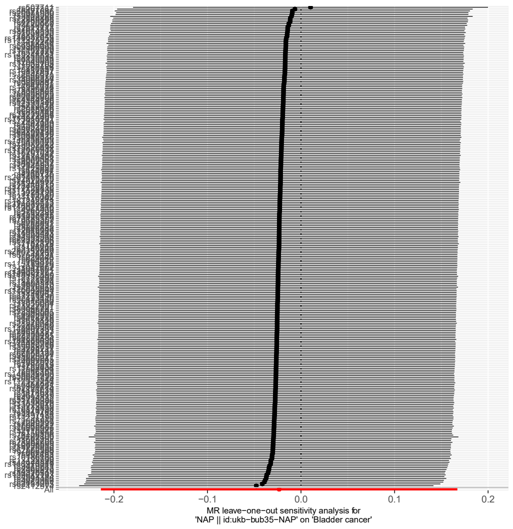
All
−0.2
−0.1
0.0
0.1
0.2
MR leave−one−out sensitivity analysis for
'NAP || id:ukb-bub35-NAP' on 'Bladder cancer'

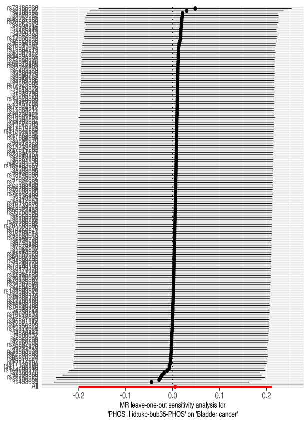
All
−0.2
−0.1
0.0
0.1
0.2
MR leave−one−out sensitivity analysis for
'PHOS || id:ukb-bub35-PHOS' on 'Bladder cancer'

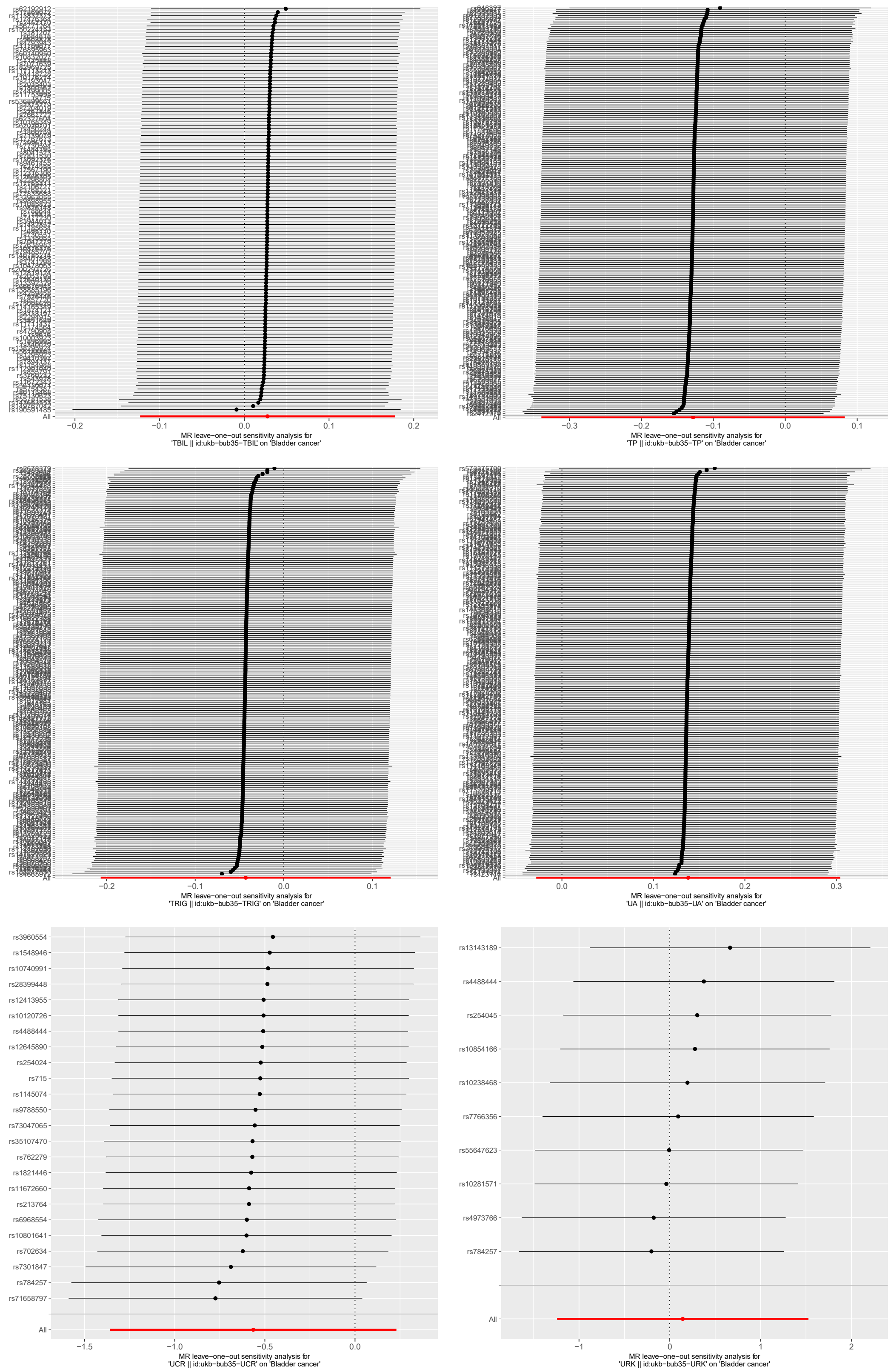

MR leave−one−out sensitivity analysis for
'TBIL || id:ukb−bub35−TBIL' on 'Bladder cancer'
MR leave−one−out sensitivity analysis for
'TP || id:ukb−bub35−TP' on 'Bladder cancer'
MR leave−one−out sensitivity analysis for
'TRIG || id:ukb−bub35−TRIG' on 'Bladder cancer'
MR leave−one−out sensitivity analysis for
'UA || id:ukb−bub35−UA' on 'Bladder cancer'
rs3960554
rs1548946
rs10740991
rs28399448
rs12413955
rs10120726
rs4488444
rs12645890
rs254024
rs715
rs1145074
rs9788550
rs73047065
rs35107470
rs762279
rs1821446
rs11672660
rs213764
rs6968554
rs10801641
rs702634
rs7301847
rs784257
rs71658797
All
MR leave−one−out sensitivity analysis for
'UCR || id:ukb−bub35−UCR' on 'Bladder cancer'
rs13143189
rs4488444
rs254045
rs10854166
rs10238468
rs7766356
rs55647623
rs10281571
rs4973766
rs784257
All
MR leave−one−out sensitivity analysis for
'URK || id:ukb−bub35−URK' on 'Bladder cancer'

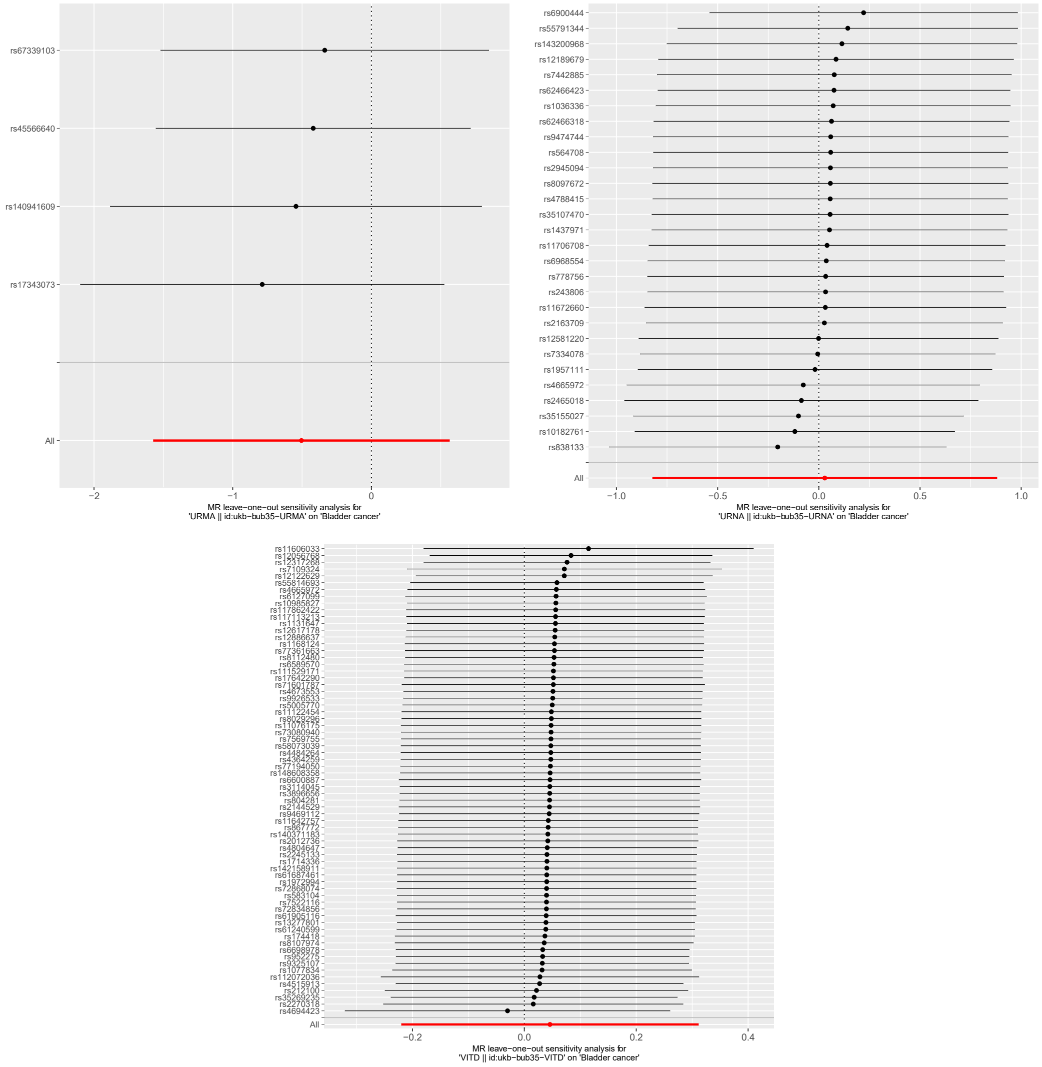
rs67339103
rs45566640
rs140941609
rs17343073
All
−2
−1
0
MR leave−one−out sensitivity analysis for
'URMA || id:ukb−bub35−URMA' on 'Bladder cancer'
rs6900444
rs55791344
rs143200968
rs12189679
rs7442885
rs62466423
rs1036336
rs62466318
rs9474744
rs564708
rs2945094
rs8097672
rs4788415
rs35107470
rs1437971
rs11706708
rs6968554
rs778756
rs243806
rs11672660
rs2163709
rs12581220
rs7334078
rs1957111
rs4665972
rs2465018
rs35155027
rs10182761
rs838133
All
−1.0
−0.5
0.0
0.5
1.0
MR leave−one−out sensitivity analysis for
'URNA || id:ukb−bub35−URNA' on 'Bladder cancer'
All
−0.2
0.0
0.2
0.4
MR leave−one−out sensitivity analysis for
'VITD || id:ukb−bub35−VITD' on 'Bladder cancer'

PCa's leave-one-out method

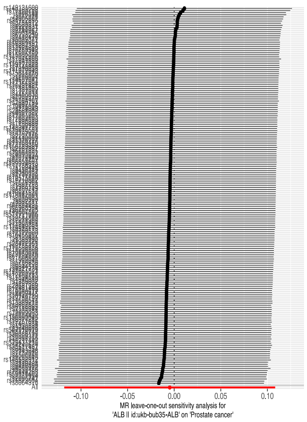

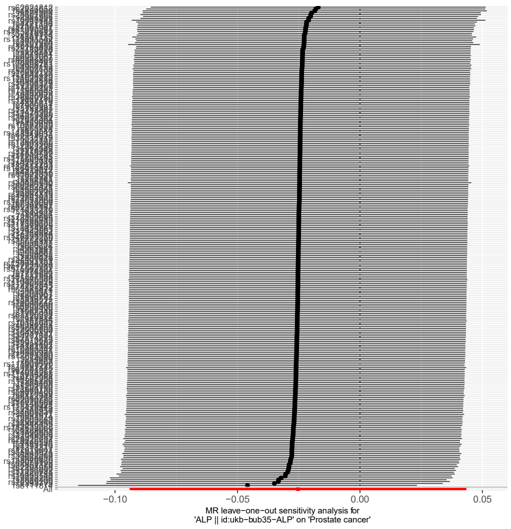

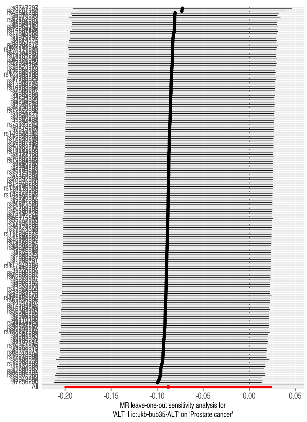

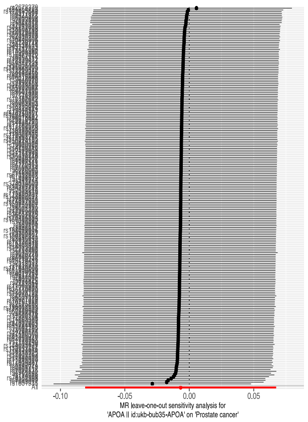

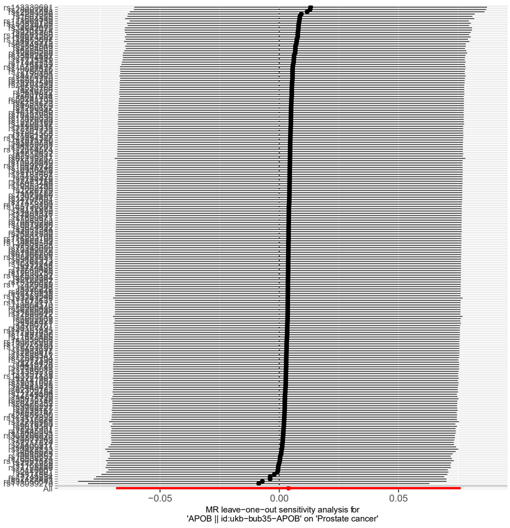

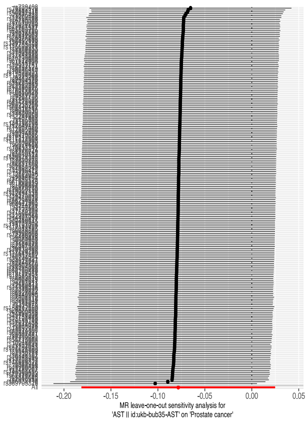

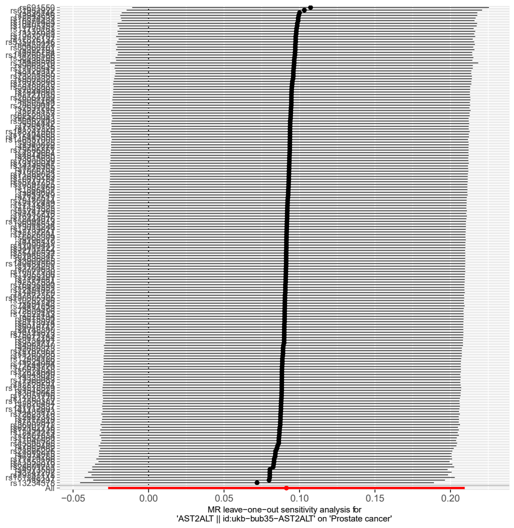
All
−0.05 0.00 0.05 0.10 0.15 0.20
MR leave−one−out sensitivity analysis for
'AST2ALT || id:ukb−bub35−AST2ALT' on 'Prostate cancer'

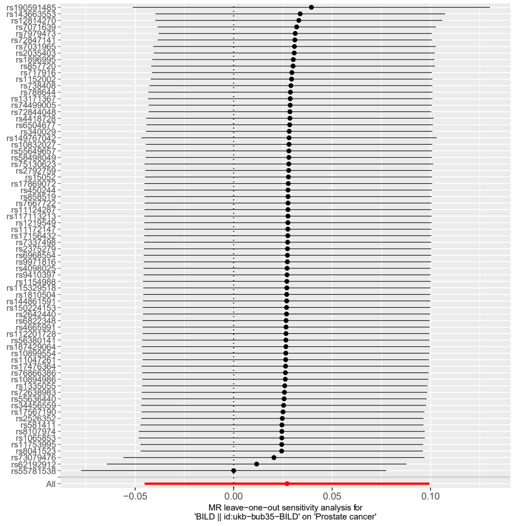
All
−0.05 0.00 0.05 0.10
MR leave−one−out sensitivity analysis for
'BILD || id:ukb−bub35−BILD' on 'Prostate cancer'

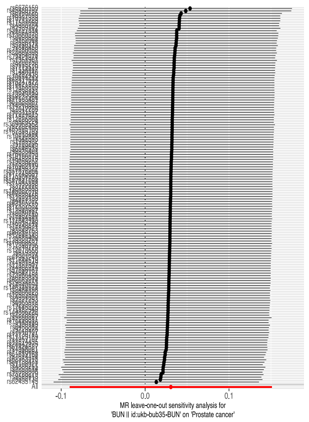
All
-0.1 0.0 0.1
MR leave-one-out sensitivity analysis for
'BUN || id:ukb-bub35-BUN' on 'Prostate cancer'

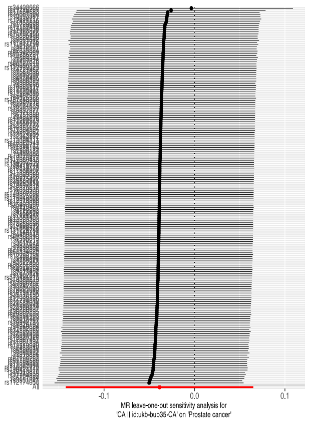
All
-0.1 0.0 0.1
MR leave-one-out sensitivity analysis for
'CA || id:ukb-bub35-CA' on 'Prostate cancer'

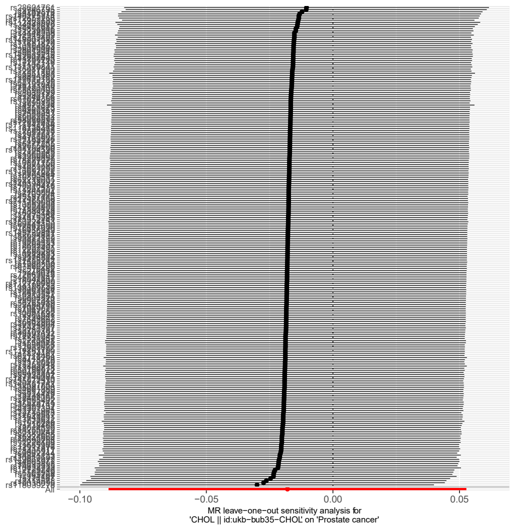
All
−0.10 −0.05 0.00 0.05
MR leave−one−out sensitivity analysis for
'CHOL || id:ukb−bub35−CHOL' on 'Prostate cancer'

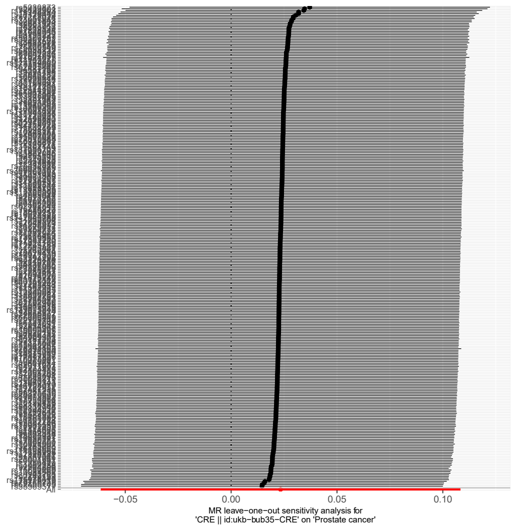
All
−0.05 0.00 0.05 0.10
MR leave−one−out sensitivity analysis for
'CRE || id:ukb−bub35−CRE' on 'Prostate cancer'

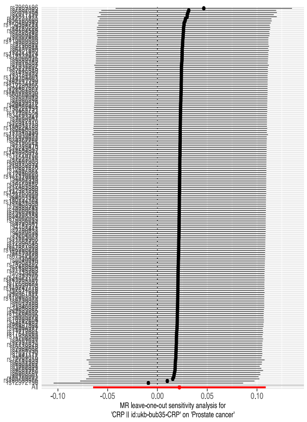
-0.10
-0.05
0.00
0.05
0.10
MR leave-one-out sensitivity analysis for
'CRP || id:ukb-bub35-CRP' on 'Prostate cancer'

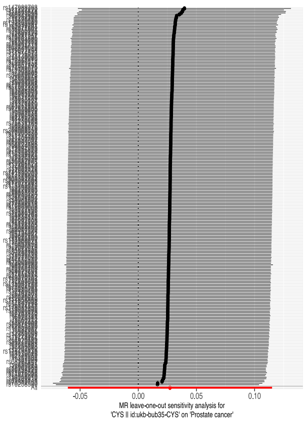
-0.05
0.00
0.05
0.10
MR leave-one-out sensitivity analysis for
'CYS || id:ukb-bub35-CYS' on 'Prostate cancer'

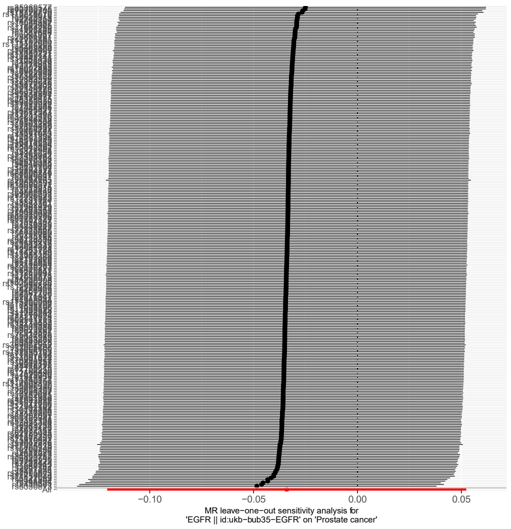
-0.10
-0.05
0.00
0.05
MR leave-one-out sensitivity analysis for
'EGFR || id:ukb-bub35-EGFR' on 'Prostate cancer'

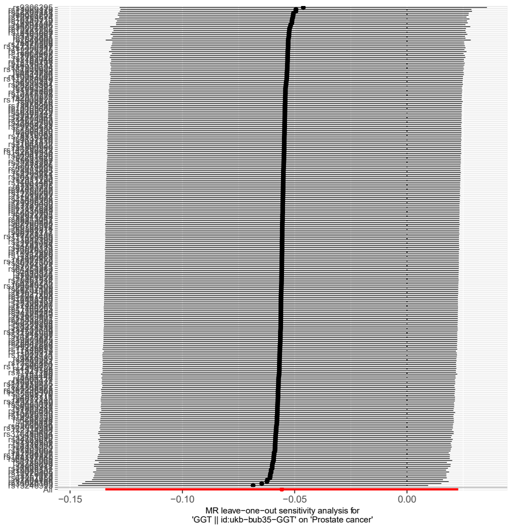
-0.15
-0.10
-0.05
0.00
MR leave-one-out sensitivity analysis for
'GGT || id:ukb-bub35-GGT' on 'Prostate cancer'

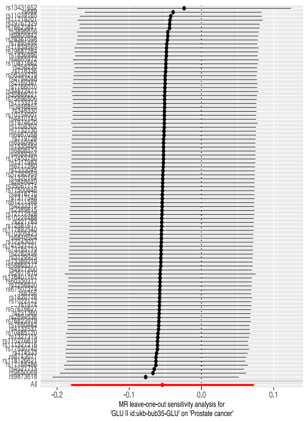
-0.2
-0.1
0.0
0.1
MR leave-one-out sensitivity analysis for
'GLU || id:ukb-bub35-GLU' on 'Prostate cancer'

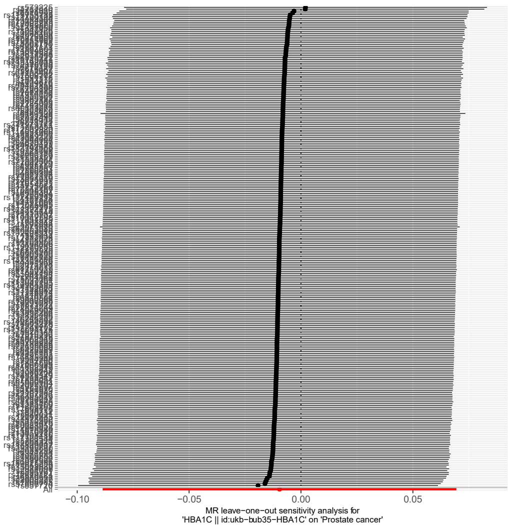
-0.10
-0.05
0.00
0.05
MR leave-one-out sensitivity analysis for
'HBA1C || id:ukb-bub35-HBA1C' on 'Prostate cancer'

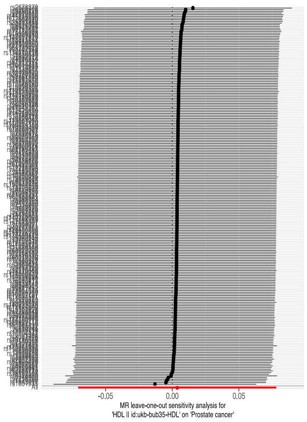
-0.05
0.00
0.05
MR leave-one-out sensitivity analysis for
'HDL || id:ukb-bub35-HDL' on 'Prostate cancer'

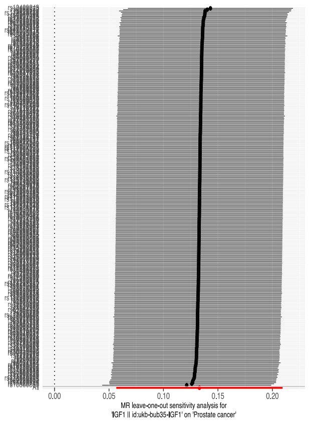
0.00
0.05
0.10
0.15
0.20
MR leave-one-out sensitivity analysis for
'IGF1 || id:ukb-bub35-IGF1' on 'Prostate cancer'

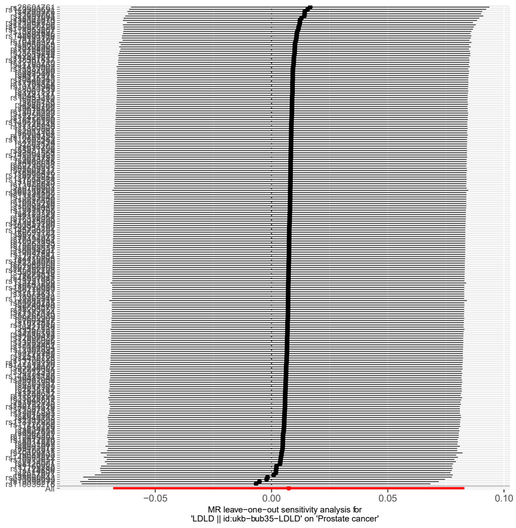
All
−0.05
0.00
0.05
0.10
MR leave−one−out sensitivity analysis for
'LDLD || id:ukb−bub35−LDLD' on 'Prostate cancer'

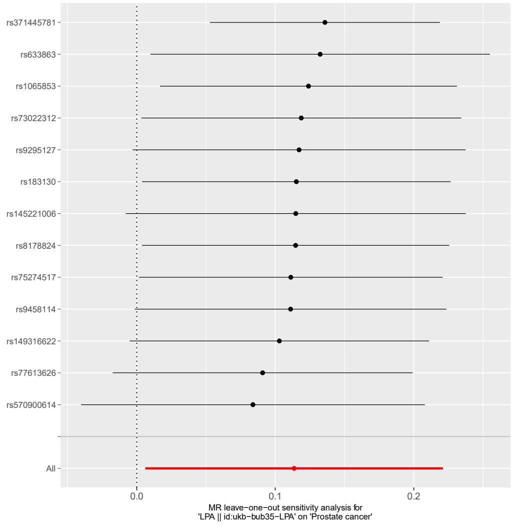
rs371445781
rs633863
rs1065853
rs73022312
rs9295127
rs183130
rs145221006
rs8178824
rs75274517
rs9458114
rs149316622
rs77613626
rs570900614
All
0.0
0.1
0.2
MR leave−one−out sensitivity analysis for
'LPA || id:ukb−bub35−LPA' on 'Prostate cancer'

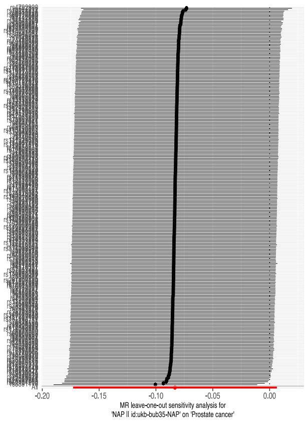
-0.20
-0.15
-0.10
-0.05
0.00
MR leave-one-out sensitivity analysis for
'NAP || id:ukb-bub35-NAP' on 'Prostate cancer'

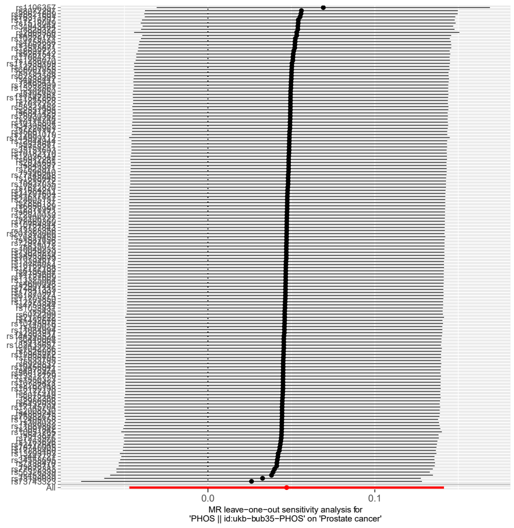
All
0.0
0.1
MR leave−one−out sensitivity analysis for
'PHOS || id:ukb−bub35−PHOS' on 'Prostate cancer'

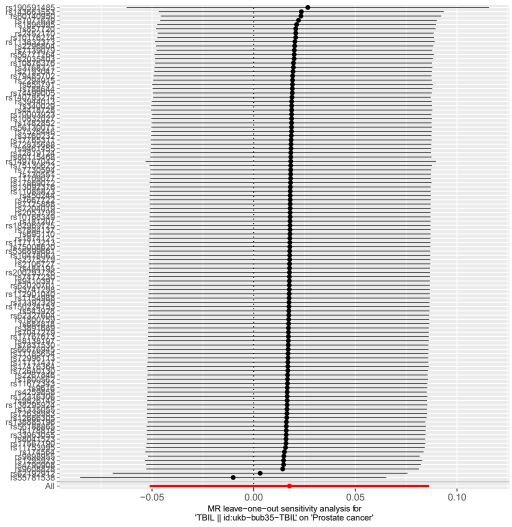
All
−0.05
0.00
0.05
0.10
MR leave−one−out sensitivity analysis for
'TBIL || id:ukb−bub35−TBIL' on 'Prostate cancer'

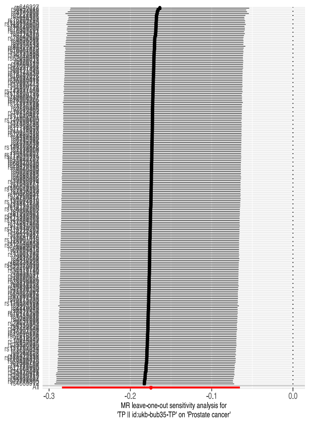
All
-0.3
-0.2
-0.1
0.0
MR leave-one-out sensitivity analysis for
'TP || id:ukb-bub35-TP' on 'Prostate cancer'

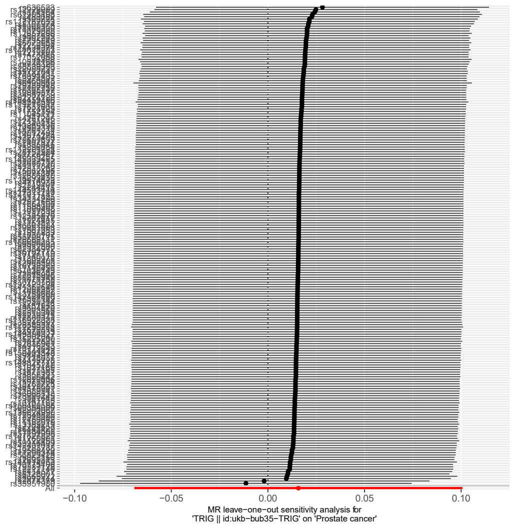
All
−0.10
−0.05
0.00
0.05
0.10
MR leave−one−out sensitivity analysis for
'TRIG || id:ukb−bub35−TRIG' on 'Prostate cancer'

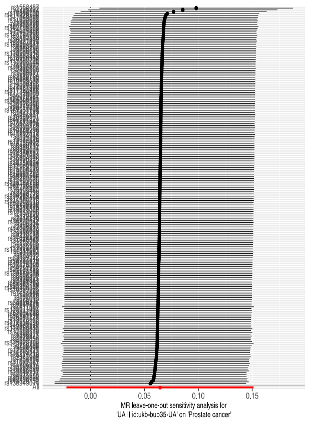
All
0.00
0.05
0.10
0.15
MR leave-one-out sensitivity analysis for
'UA || id:ukb-bub35-UA' on 'Prostate cancer'

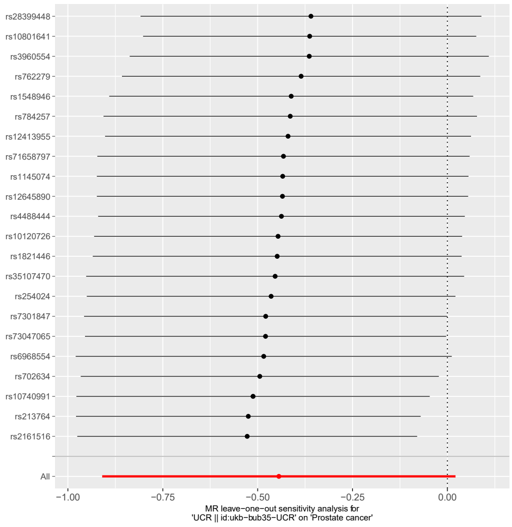
rs28399448
rs10801641
rs3960554
rs762279
rs1548946
rs784257
rs12413955
rs71658797
rs1145074
rs12645890
rs4488444
rs10120726
rs1821446
rs35107470
rs254024
rs7301847
rs73047065
rs6968554
rs702634
rs10740991
rs213764
rs2161516
All
−1.00
−0.75
−0.50
−0.25
0.00
MR leave−one−out sensitivity analysis for
'UCR || id:ukb−bub35−UCR' on 'Prostate cancer'

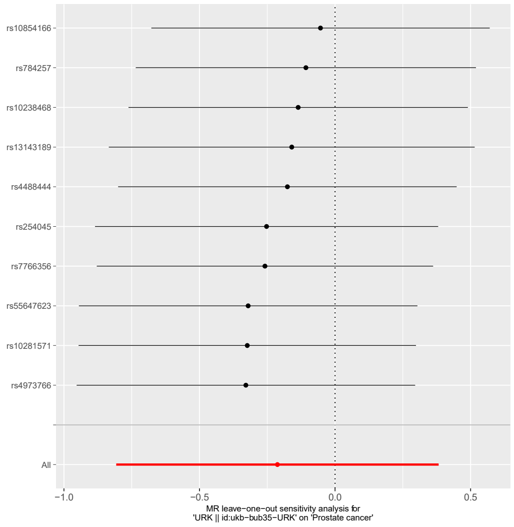
rs10854166
rs784257
rs10238468
rs13143189
rs4488444
rs254045
rs7766356
rs55647623
rs10281571
rs4973766
All
−1.0
−0.5
0.0
0.5
MR leave−one−out sensitivity analysis for
'URK || id:ukb−bub35−URK' on 'Prostate cancer'

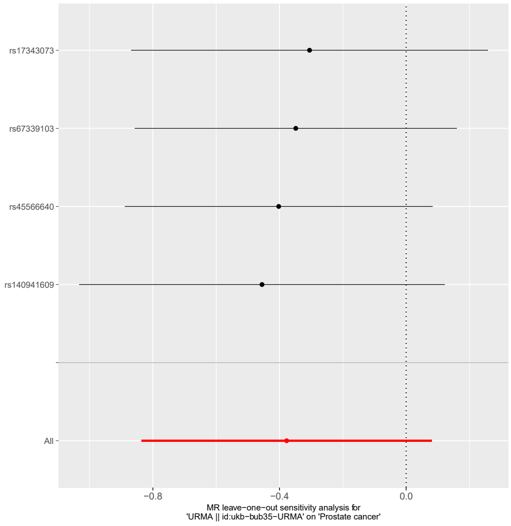
rs17343073
rs67339103
rs45566640
rs140941609
All
−0.8
−0.4
0.0
MR leave−one−out sensitivity analysis for
'URMA || id:ukb−bub35−URMA' on 'Prostate cancer'

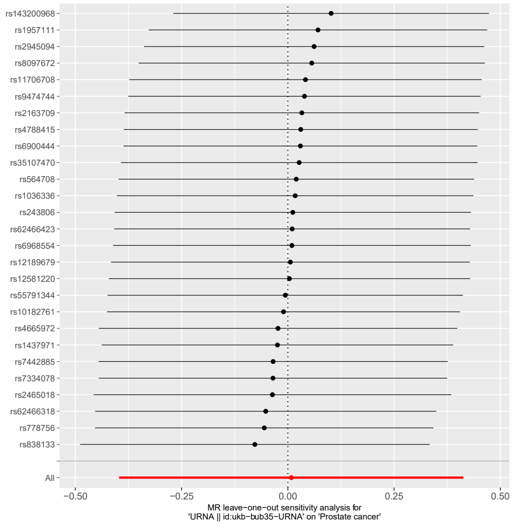
rs143200968
rs1957111
rs2945094
rs8097672
rs11706708
rs9474744
rs2163709
rs4788415
rs6900444
rs35107470
rs564708
rs1036336
rs243806
rs62466423
rs6968554
rs12189679
rs12581220
rs55791344
rs10182761
rs4665972
rs1437971
rs7442885
rs7334078
rs2465018
rs62466318
rs778756
rs838133
All
−0.50
−0.25
0.00
0.25
0.50
MR leave−one−out sensitivity analysis for
'URNA || id:ukb−bub35−URNA' on 'Prostate cancer'

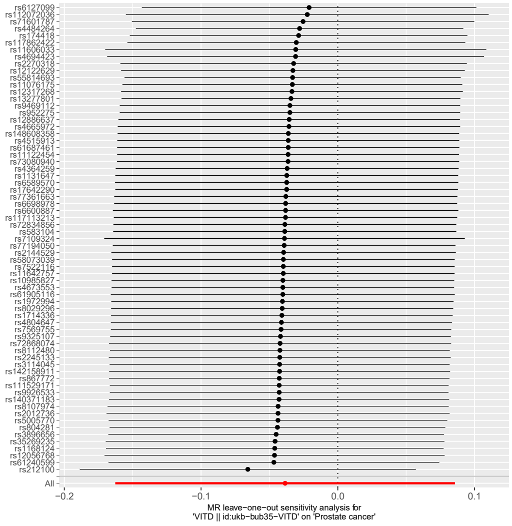
All
−0.2
−0.1
0.0
0.1
MR leave−one−out sensitivity analysis for
'VITD || id:ukb−bub35−VITD' on 'Prostate cancer'

RPCa's leave-one-out method

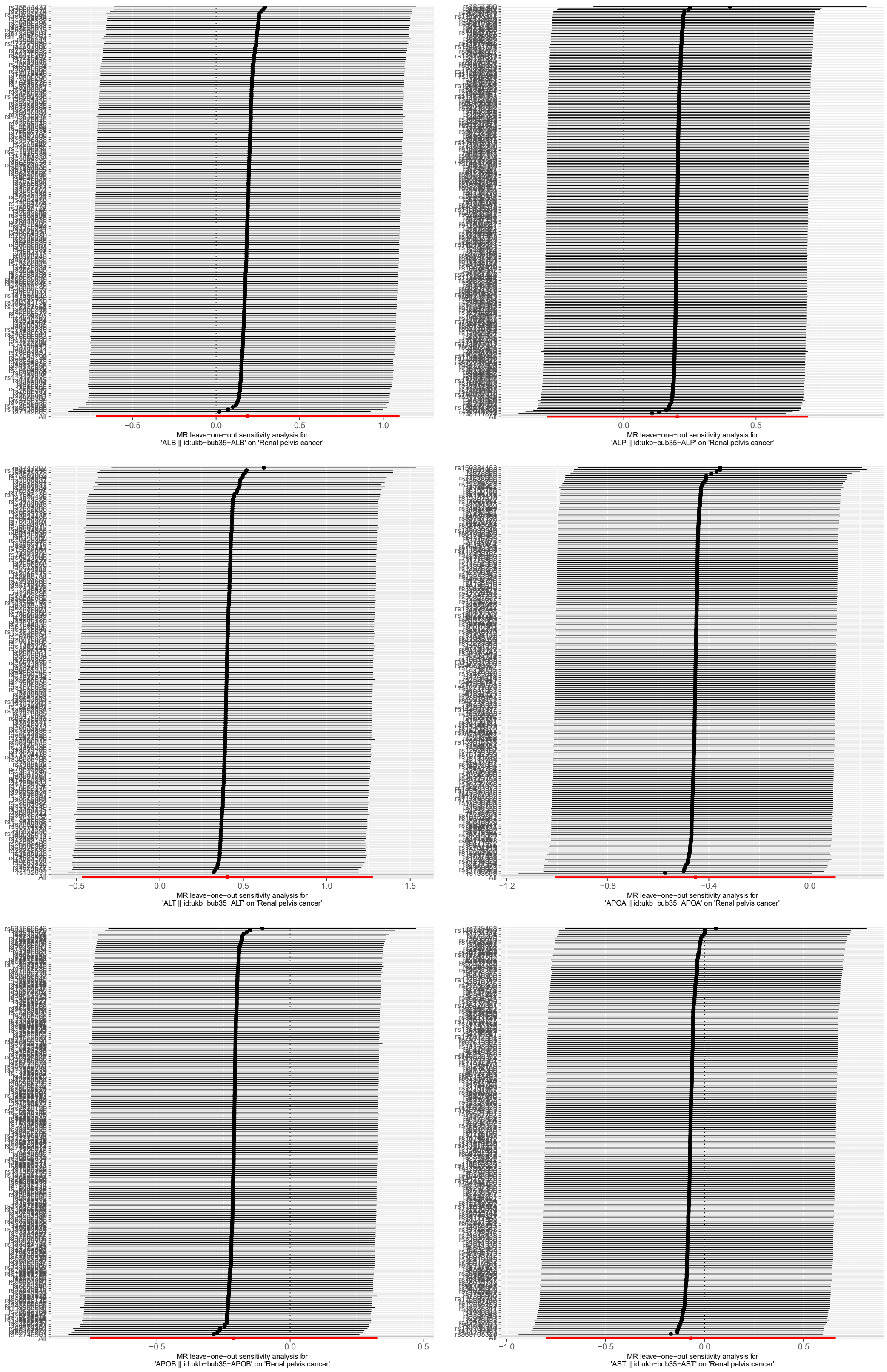

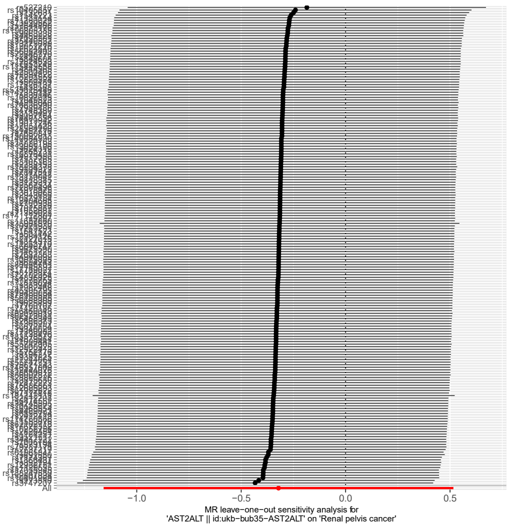
All
−1.0
−0.5
0.0
0.5
MR leave−one−out sensitivity analysis for
'AST2ALT || id:ukb-bub35-AST2ALT' on 'Renal pelvis cancer'

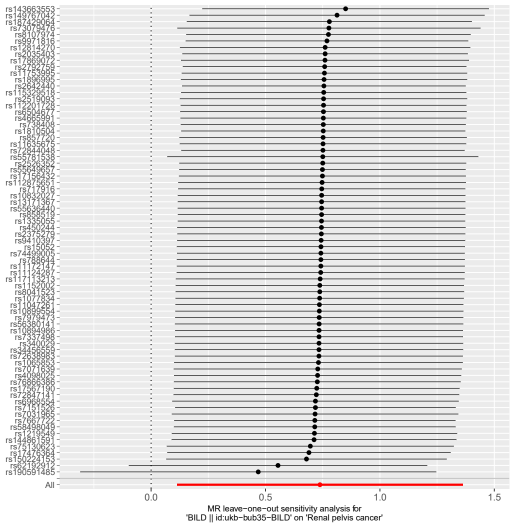
rs143663553
All
0.0
0.5
1.0
1.5
MR leave−one−out sensitivity analysis for
'BILD || id:ukb-bub35-BILD' on 'Renal pelvis cancer'

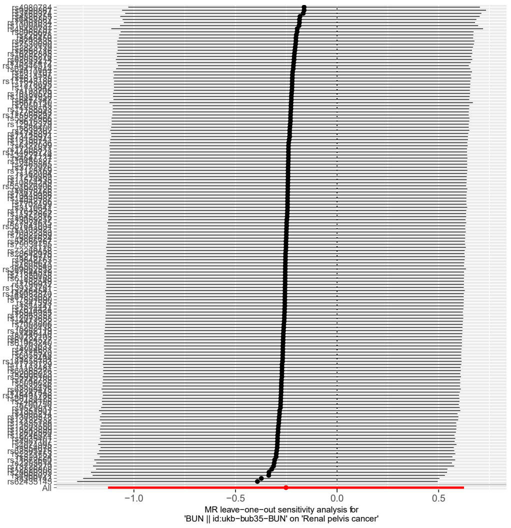
All
−1.0
−0.5
0.0
0.5
MR leave−one−out sensitivity analysis for
'BUN || id:ukb-bub35-BUN' on 'Renal pelvis cancer'

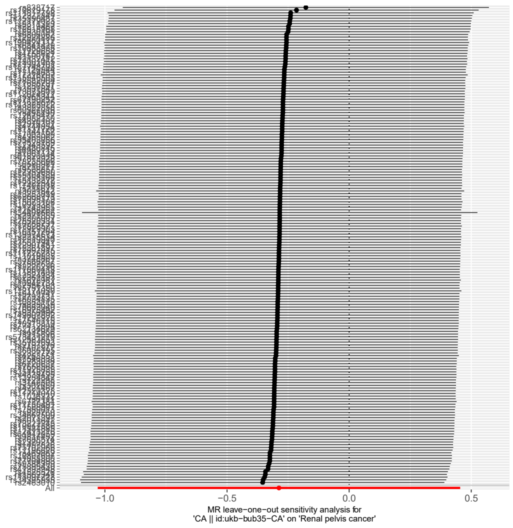
All
−1.0
−0.5
0.0
0.5
MR leave−one−out sensitivity analysis for
'CA || id:ukb-bub35-CA' on 'Renal pelvis cancer'

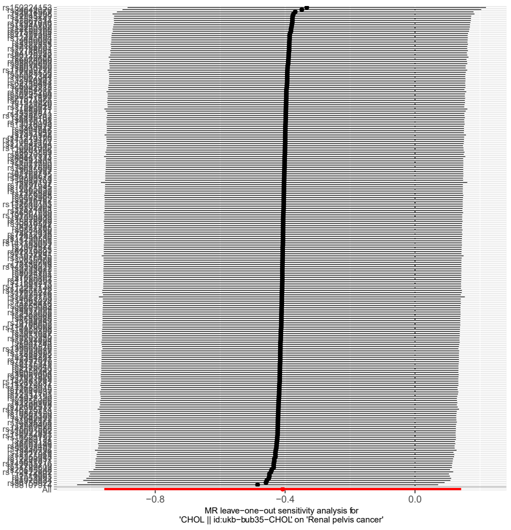
All
−0.8
−0.4
0.0
MR leave−one−out sensitivity analysis for
'CHOL || id:ukb-bub35-CHOL' on 'Renal pelvis cancer'

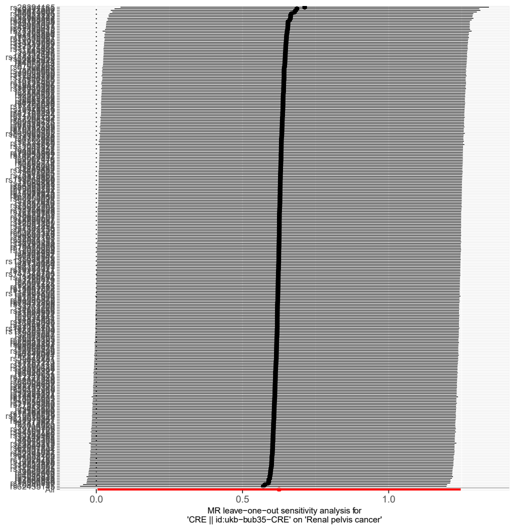
All
0.0
0.5
1.0
MR leave−one−out sensitivity analysis for
'CRE || id:ukb-bub35-CRE' on 'Renal pelvis cancer'

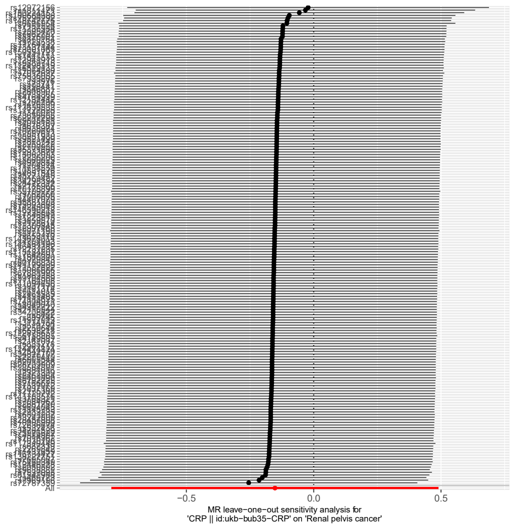
All
-0.5
0.0
0.5
MR leave−one−out sensitivity analysis for
'CRP || id:ukb−bub35−CRP' on 'Renal pelvis cancer'

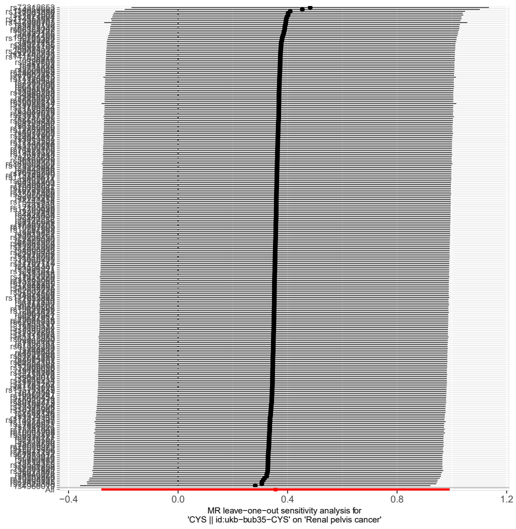
All
-0.4
0.0
0.4
0.8
1.2
MR leave−one−out sensitivity analysis for
'CYS || id:ukb−bub35−CYS' on 'Renal pelvis cancer'

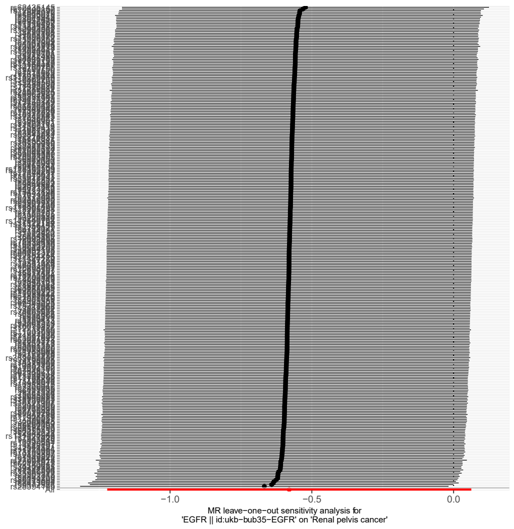
All
-1.0
-0.5
0.0
MR leave−one−out sensitivity analysis for
'EGFR || id:ukb−bub35−EGFR' on 'Renal pelvis cancer'

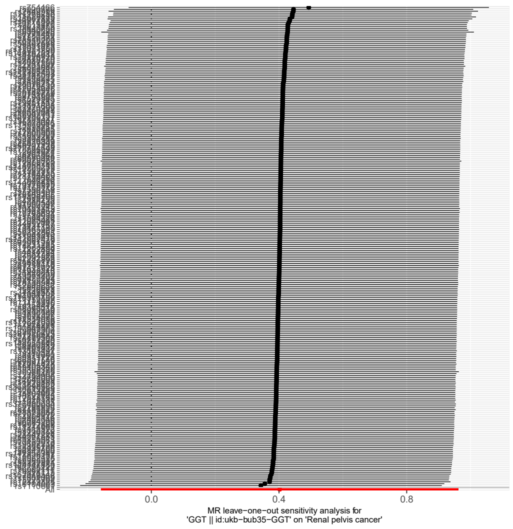
All
0.0
0.4
0.8
MR leave−one−out sensitivity analysis for
'GGT || id:ukb−bub35−GGT' on 'Renal pelvis cancer'

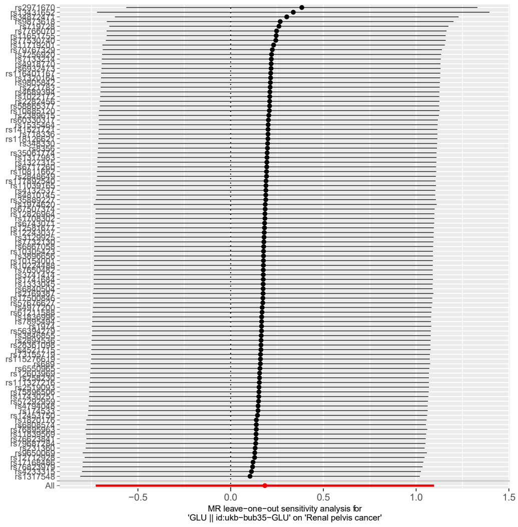
All
-0.5
0.0
0.5
1.0
1.5
MR leave−one−out sensitivity analysis for
'GLU || id:ukb−bub35−GLU' on 'Renal pelvis cancer'

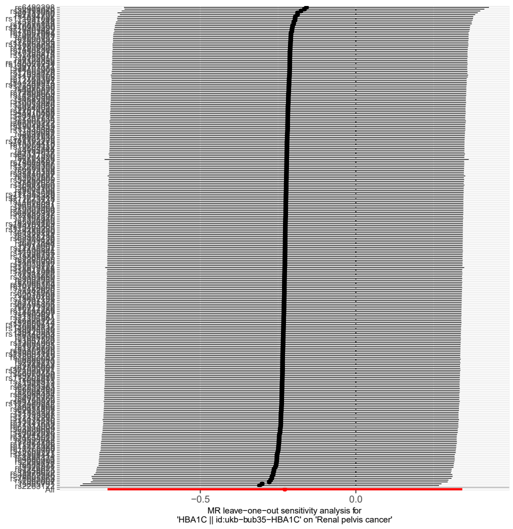
All
-0.5
0.0
MR leave−one−out sensitivity analysis for
'HBA1C || id:ukb−bub35−HBA1C' on 'Renal pelvis cancer'

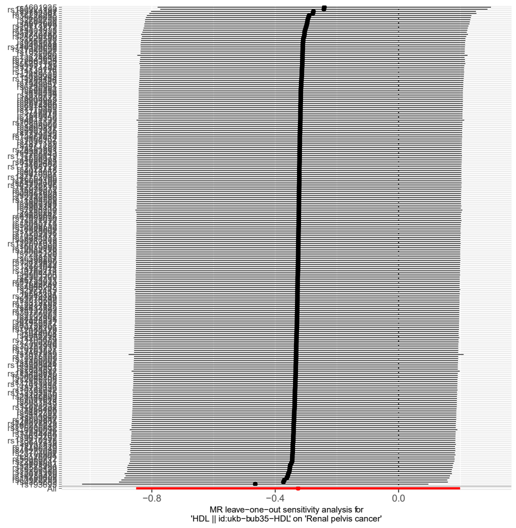
All
−0.8
−0.4
0.0
MR leave−one−out sensitivity analysis for
'HDL || id:ukb−bub35−HDL' on 'Renal pelvis cancer'

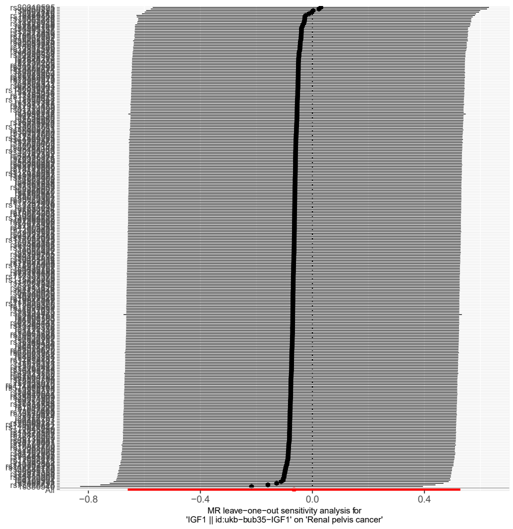
All
−0.8
−0.4
0.0
0.4
MR leave−one−out sensitivity analysis for
'IGF1 || id:ukb−bub35−IGF1' on 'Renal pelvis cancer'

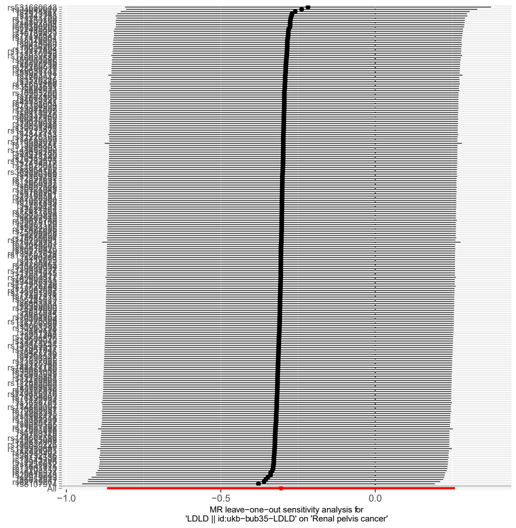
All
−1.0
−0.5
0.0
MR leave−one−out sensitivity analysis for
'LDLD || id:ukb−bub35−LDLD' on 'Renal pelvis cancer'

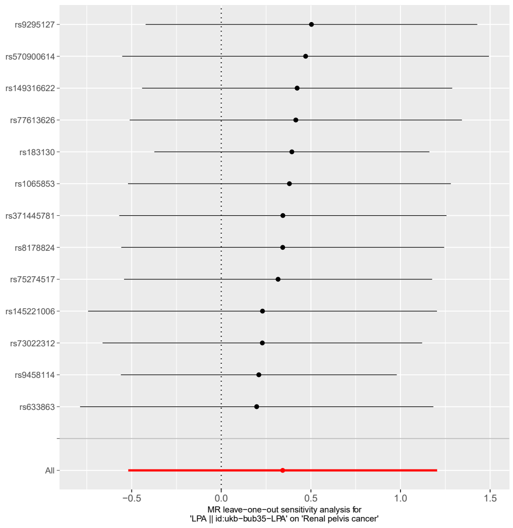
rs9295127
rs570900614
rs149316622
rs77613626
rs183130
rs1065853
rs371445781
rs8178824
rs75274517
rs145221006
rs73022312
rs9458114
rs633863
All
−0.5
0.0
0.5
1.0
1.5
MR leave−one−out sensitivity analysis for
'LPA || id:ukb−bub35−LPA' on 'Renal pelvis cancer'

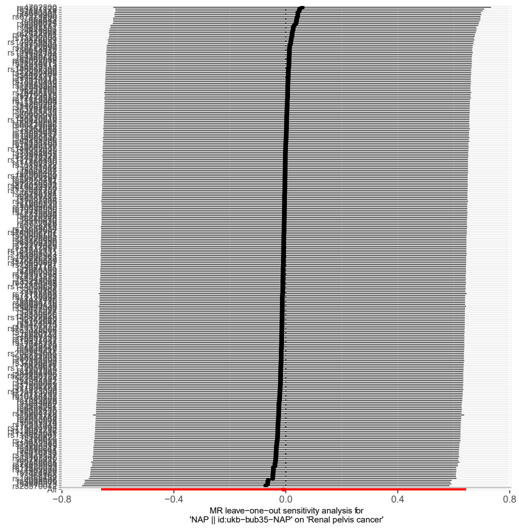
All
−0.8
−0.4
0.0
0.4
0.8
MR leave−one−out sensitivity analysis for
'NAP || id:ukb−bub35−NAP' on 'Renal pelvis cancer'

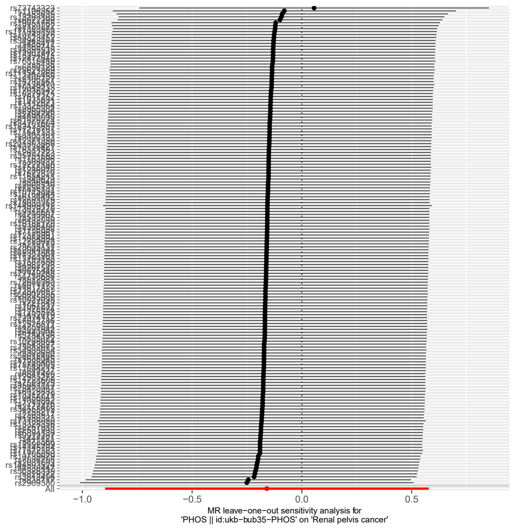
All
−1.0
−0.5
0.0
0.5
MR leave−one−out sensitivity analysis for
'PHOS || id:ukb−bub35−PHOS' on 'Renal pelvis cancer'

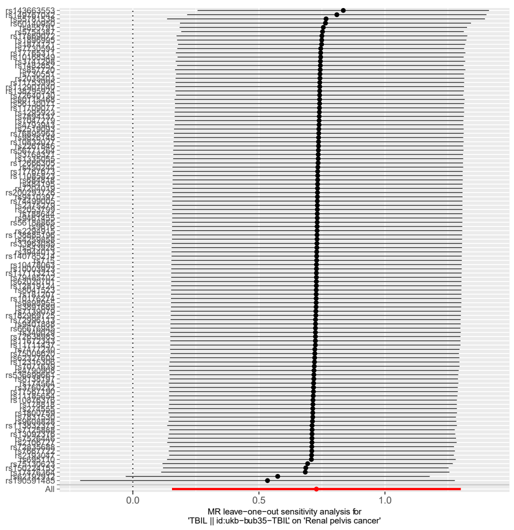
All
0.0
0.5
1.0
MR leave−one−out sensitivity analysis for
'TBIL || id:ukb−bub35−TBIL' on 'Renal pelvis cancer'

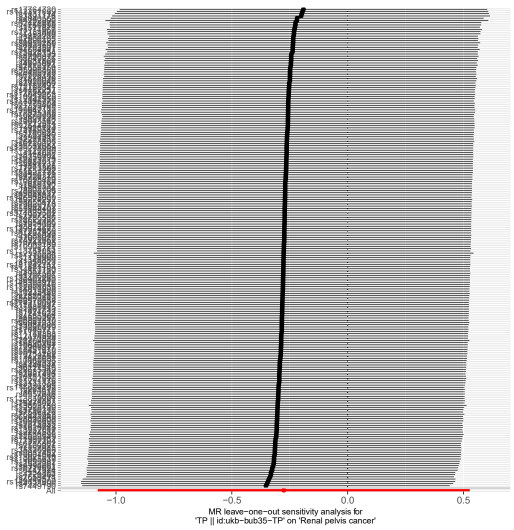
All
−1.0
−0.5
0.0
0.5
MR leave−one−out sensitivity analysis for
'TP || id:ukb−bub35−TP' on 'Renal pelvis cancer'

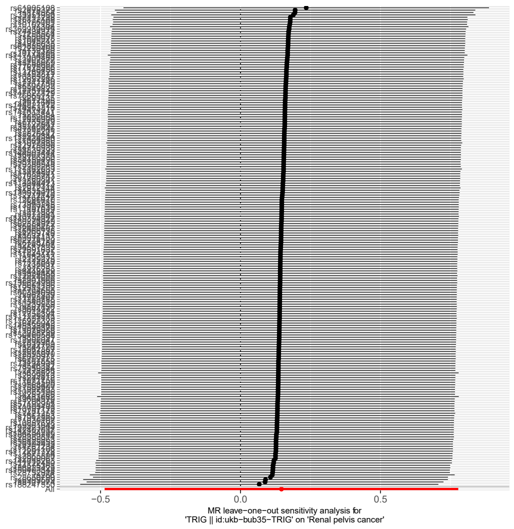
All
−0.5
0.0
0.5
MR leave−one−out sensitivity analysis for
'TRIG || id:ukb−bub35−TRIG' on 'Renal pelvis cancer'

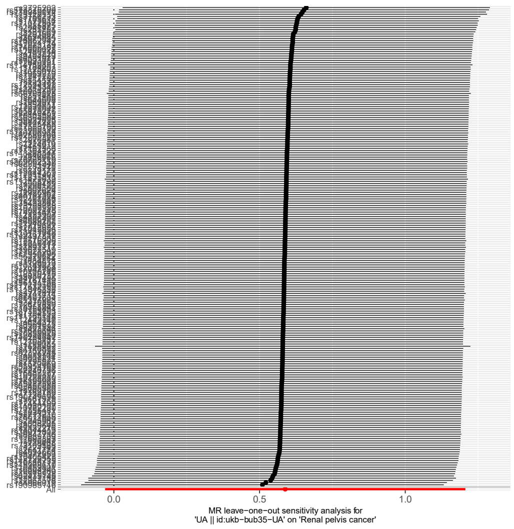
All
0.0
0.5
1.0
MR leave−one−out sensitivity analysis for
'UA || id:ukb−bub35−UA' on 'Renal pelvis cancer'

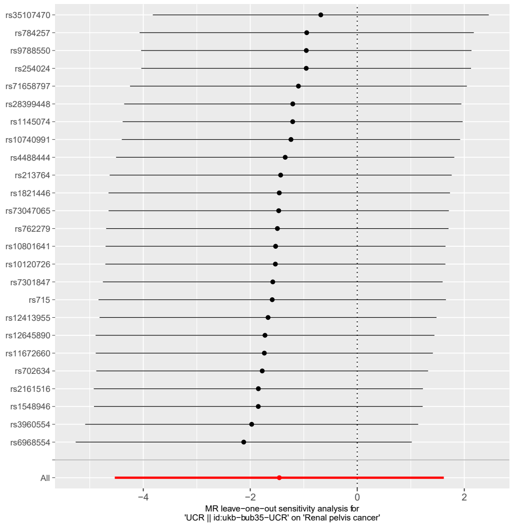
rs35107470
rs784257
rs9788550
rs254024
rs71658797
rs28399448
rs1145074
rs10740991
rs4488444
rs213764
rs1821446
rs73047065
rs762279
rs10801641
rs10120726
rs7301847
rs715
rs12413955
rs12645890
rs11672660
rs702634
rs2161516
rs1548946
rs3960554
rs6968554
All
−4
−2
0
2
MR leave−one−out sensitivity analysis for
'UCR || id:ukb−bub35−UCR' on 'Renal pelvis cancer'

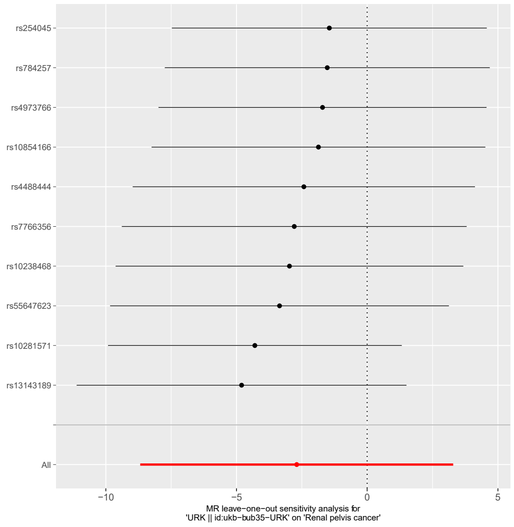
rs254045
rs784257
rs4973766
rs10854166
rs4488444
rs7766356
rs10238468
rs55647623
rs10281571
rs13143189
All
−10
−5
0
5
MR leave−one−out sensitivity analysis for
'URK || id:ukb−bub35−URK' on 'Renal pelvis cancer'

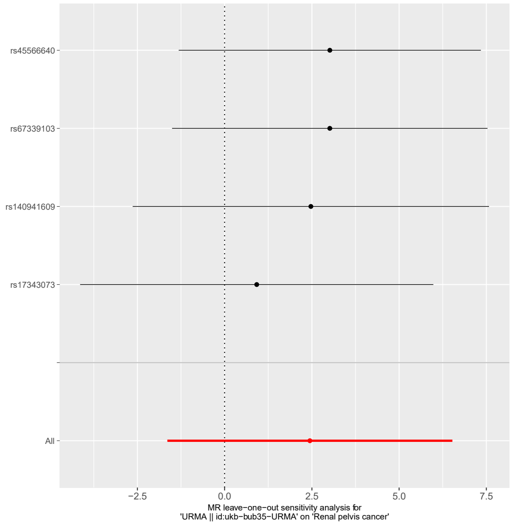
rs45566640
rs67339103
rs140941609
rs17343073
All
−2.5
0.0
2.5
5.0
7.5
MR leave−one−out sensitivity analysis for
'URMA || id:ukb−bub35−URMA' on 'Renal pelvis cancer'

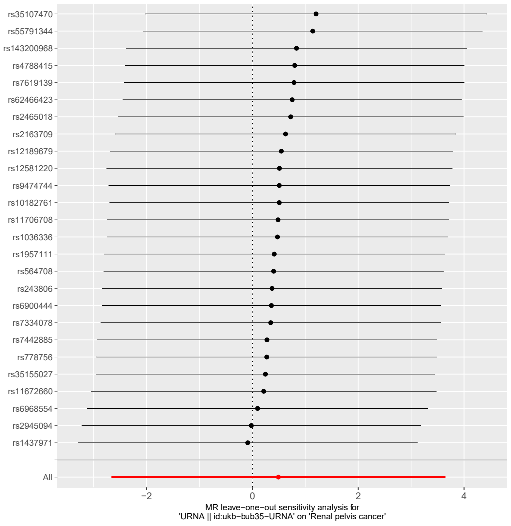
rs35107470
rs55791344
rs143200968
rs4788415
rs7619139
rs62466423
rs2465018
rs2163709
rs12189679
rs12581220
rs9474744
rs10182761
rs11706708
rs1036336
rs1957111
rs564708
rs243806
rs6900444
rs7334078
rs7442885
rs778756
rs35155027
rs11672660
rs6968554
rs2945094
rs1437971
All
−2
0
2
4
MR leave−one−out sensitivity analysis for
'URNA || id:ukb−bub35−URNA' on 'Renal pelvis cancer'

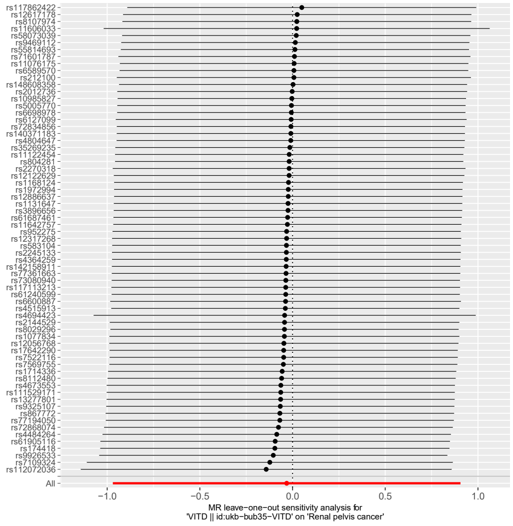
All
−1.0
−0.5
0.0
0.5
1.0
MR leave−one−out sensitivity analysis for
'VITD || id:ukb−bub35−VITD' on 'Renal pelvis cancer'

KCa's leave-one-out method

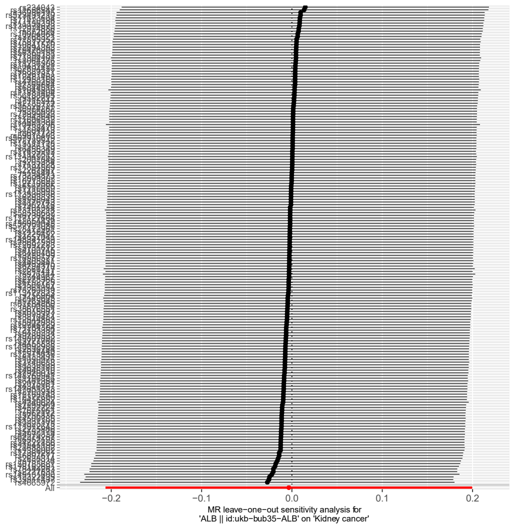

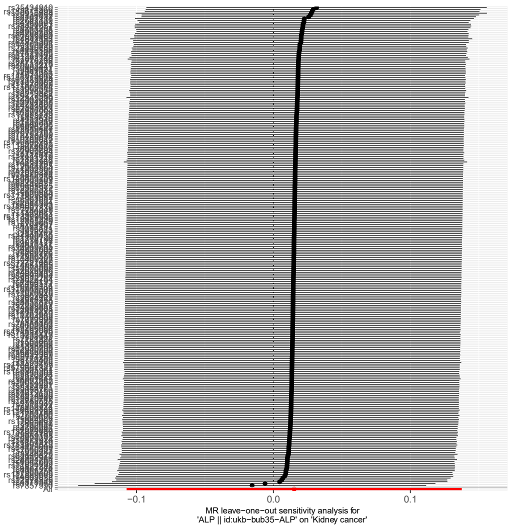

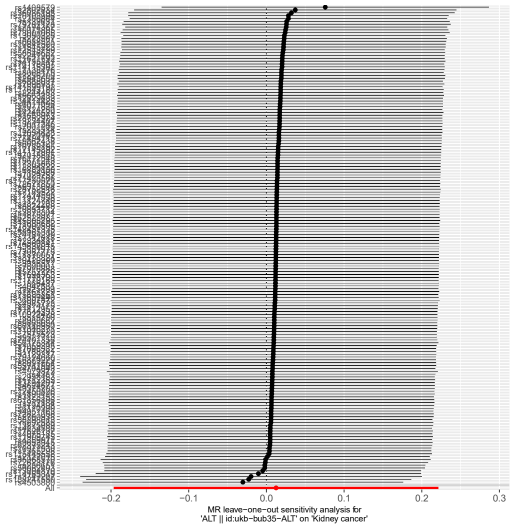

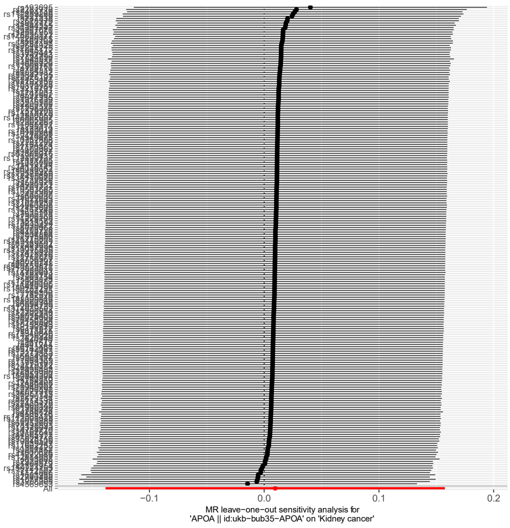

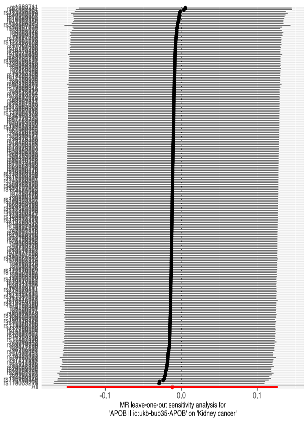

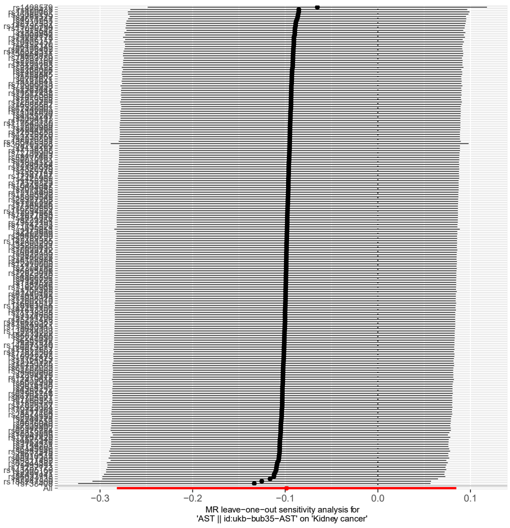

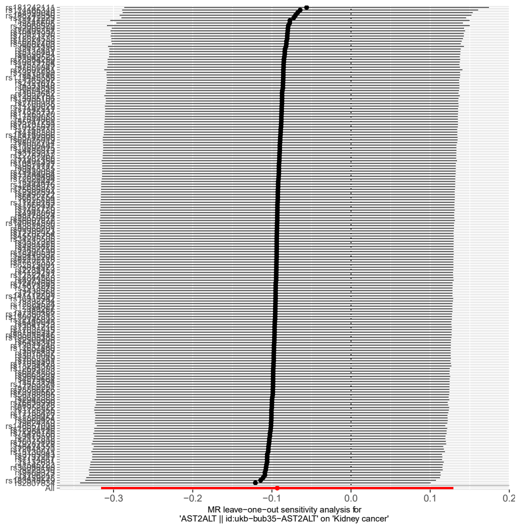
All
−0.3
−0.2
−0.1
0.0
0.1
0.2
MR leave−one−out sensitivity analysis for
'AST2ALT || id:ukb−bub35−AST2ALT' on 'Kidney cancer'

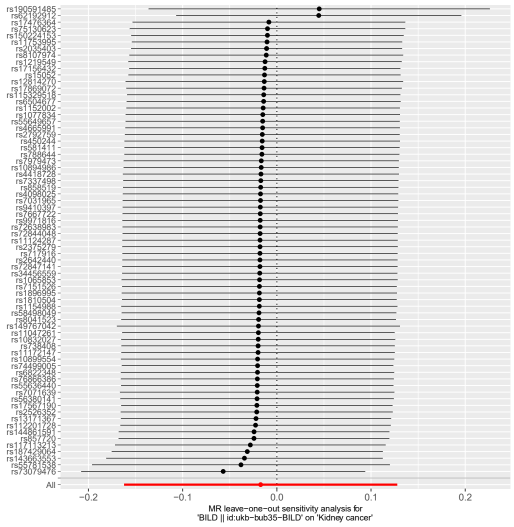
All
−0.2
−0.1
0.0
0.1
0.2
MR leave−one−out sensitivity analysis for
'BILD || id:ukb−bub35−BILD' on 'Kidney cancer'

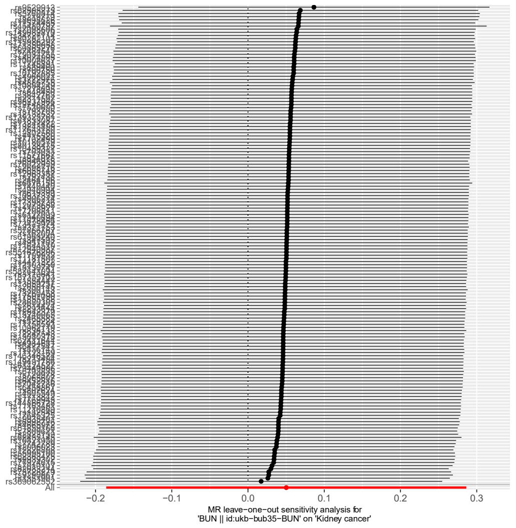
All
−0.2
−0.1
0.0
0.1
0.2
0.3
MR leave−one−out sensitivity analysis for
'BUN || id:ukb−bub35−BUN' on 'Kidney cancer'

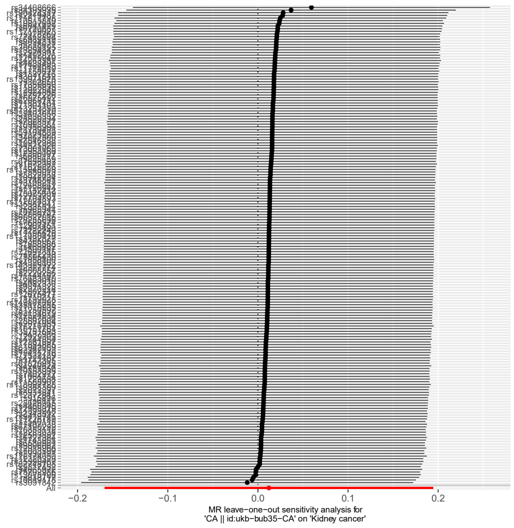
All
−0.2
−0.1
0.0
0.1
0.2
MR leave−one−out sensitivity analysis for
'CA || id:ukb−bub35−CA' on 'Kidney cancer'

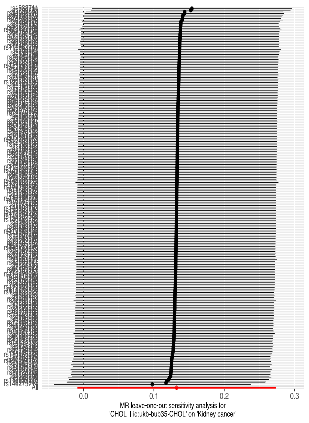
All
0.0
0.1
0.2
0.3
MR leave-one-out sensitivity analysis for
'CHOL || id:ukb-bub35-CHOL' on 'Kidney cancer'

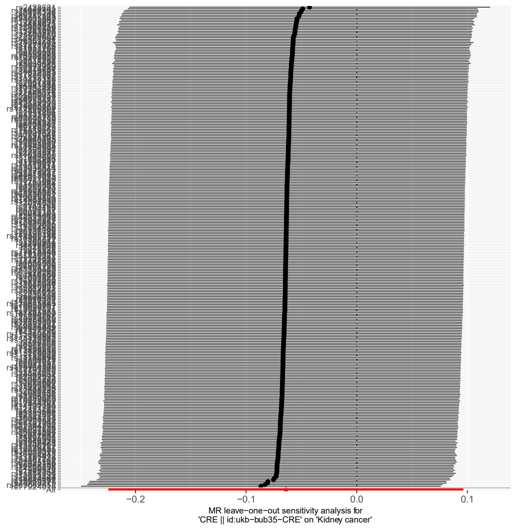
All
−0.2
−0.1
0.0
0.1
MR leave−one−out sensitivity analysis for
'CRE || id:ukb−bub35−CRE' on 'Kidney cancer'

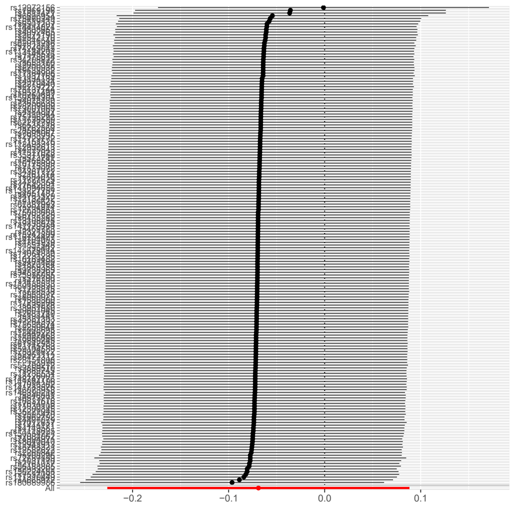

MR leave−one−out sensitivity analysis for
'CRP || id:ukb−bub35−CRP' on 'Kidney cancer'

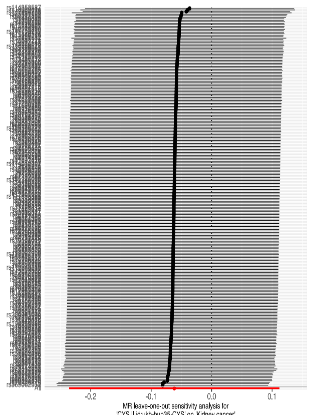

MR leave-one-out sensitivity analysis for
'CYS || id:ukb-bub35-CYS' on 'Kidney cancer'

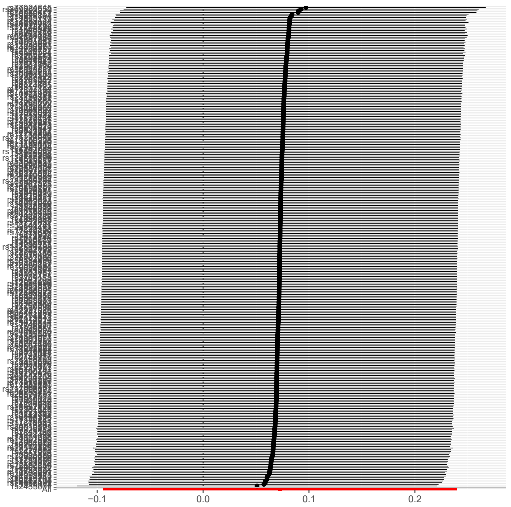

MR leave−one−out sensitivity analysis for
'EGFR || id:ukb−bub35−EGFR' on 'Kidney cancer'

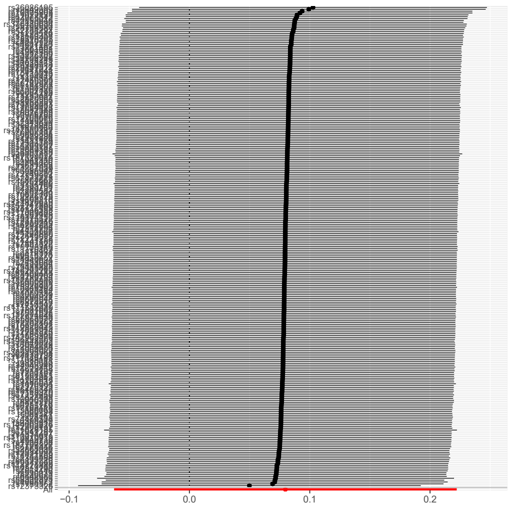

MR leave−one−out sensitivity analysis for
'GGT || id:ukb−bub35−GGT' on 'Kidney cancer'

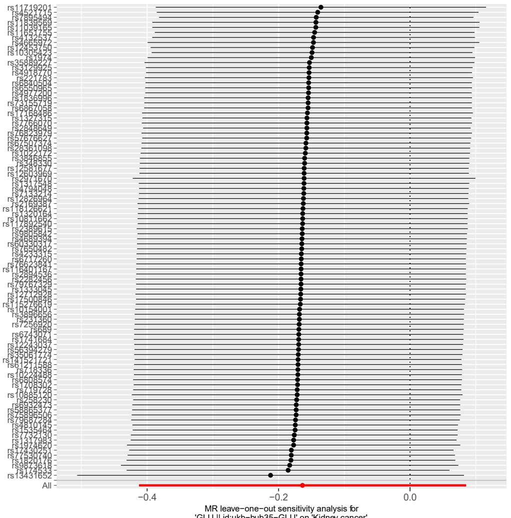

MR leave−one−out sensitivity analysis for
'GLU || id:ukb−bub35−GLU' on 'Kidney cancer'

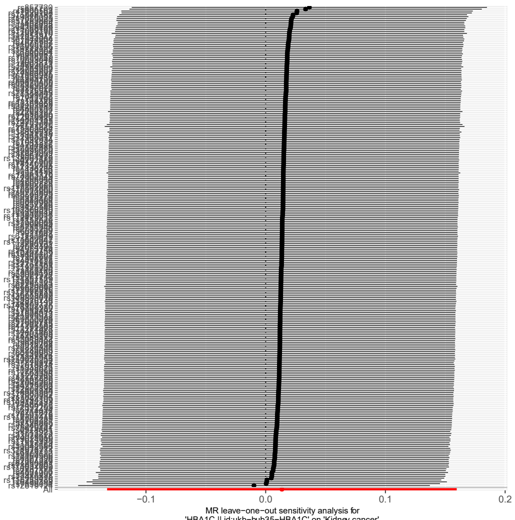

MR leave−one−out sensitivity analysis for
'HBA1C || id:ukb−bub35−HBA1C' on 'Kidney cancer'

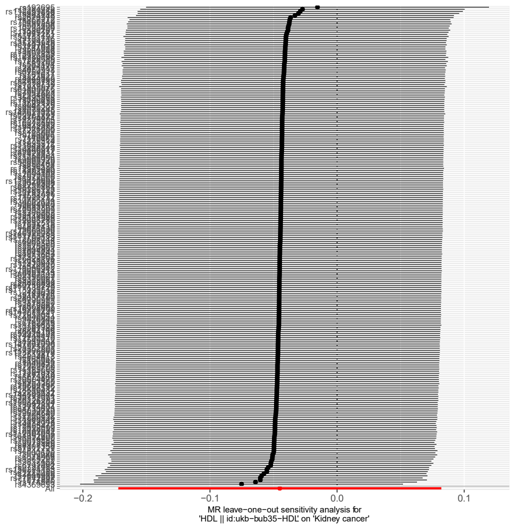
All
−0.2
−0.1
0.0
0.1
MR leave−one−out sensitivity analysis for
'HDL || id:ukb−bub35−HDL' on 'Kidney cancer'

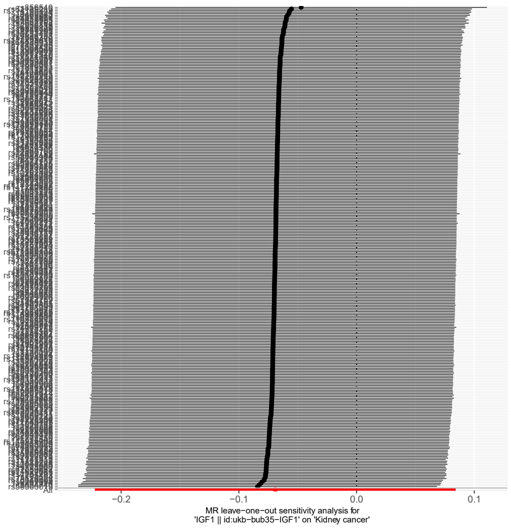
All
−0.2
−0.1
0.0
0.1
MR leave−one−out sensitivity analysis for
'IGF1 || id:ukb−bub35−IGF1' on 'Kidney cancer'

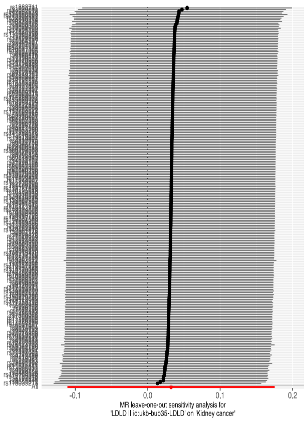
All
-0.1
0.0
0.1
0.2
MR leave-one-out sensitivity analysis for
'LDLD || id:ukb-bub35-LDLD' on 'Kidney cancer'

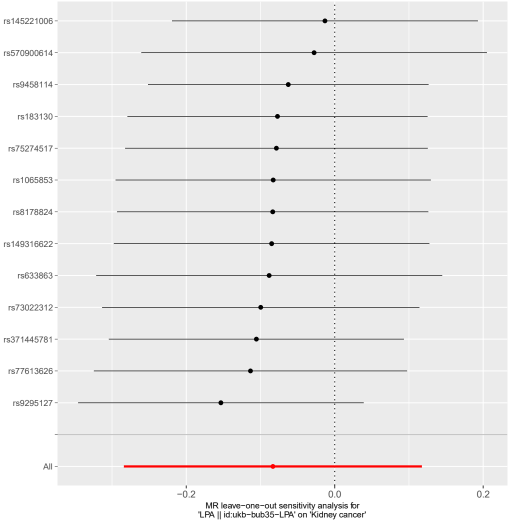
rs145221006
rs570900614
rs9458114
rs183130
rs75274517
rs1065853
rs8178824
rs149316622
rs633863
rs73022312
rs371445781
rs77613626
rs9295127
All
−0.2
0.0
0.2
MR leave−one−out sensitivity analysis for
'LPA || id:ukb−bub35−LPA' on 'Kidney cancer'

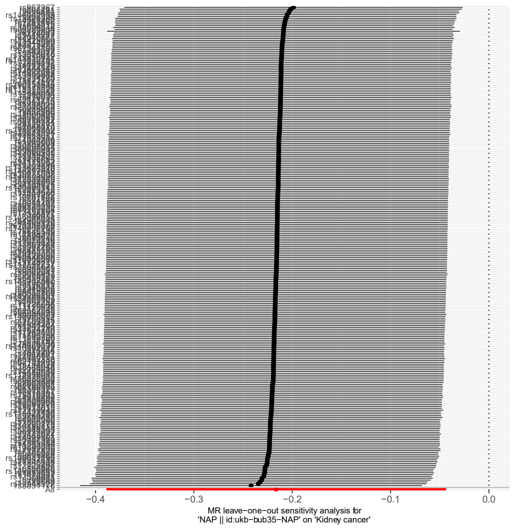
All
−0.4
−0.3
−0.2
−0.1
0.0
MR leave−one−out sensitivity analysis for
'NAP || id:ukb−bub35−NAP' on 'Kidney cancer'

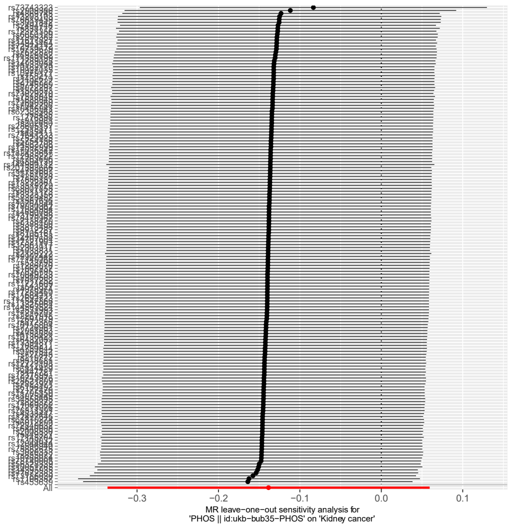
All
−0.3
−0.2
−0.1
0.0
0.1
MR leave−one−out sensitivity analysis for
'PHOS || id:ukb−bub35−PHOS' on 'Kidney cancer'

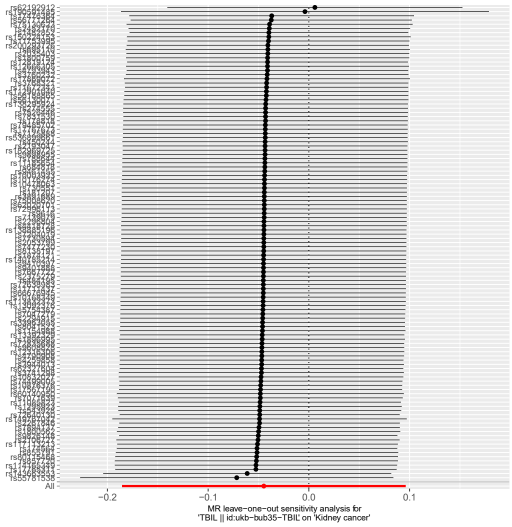

rs62192912
rs55781538
All
−0.2
−0.1
0.0
0.1
MR leave−one−out sensitivity analysis for
'TBIL || id:ukb−bub35−TBIL' on 'Kidney cancer'

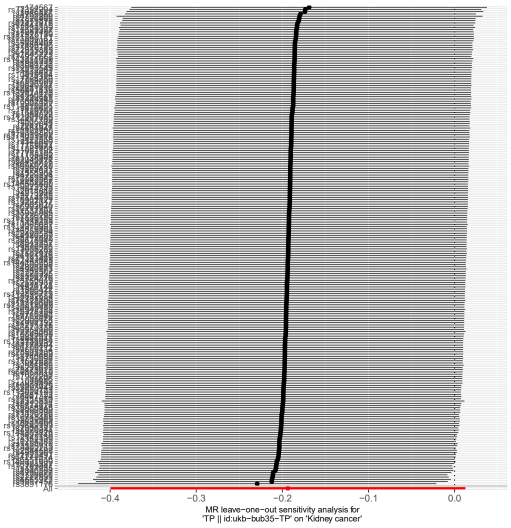

All
−0.4
−0.3
−0.2
−0.1
0.0
MR leave−one−out sensitivity analysis for
'TP || id:ukb−bub35−TP' on 'Kidney cancer'

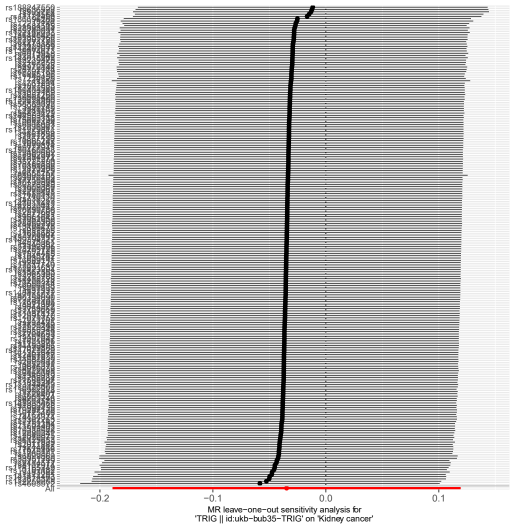

All
−0.2
−0.1
0.0
0.1
MR leave−one−out sensitivity analysis for
'TRIG || id:ukb−bub35−TRIG' on 'Kidney cancer'

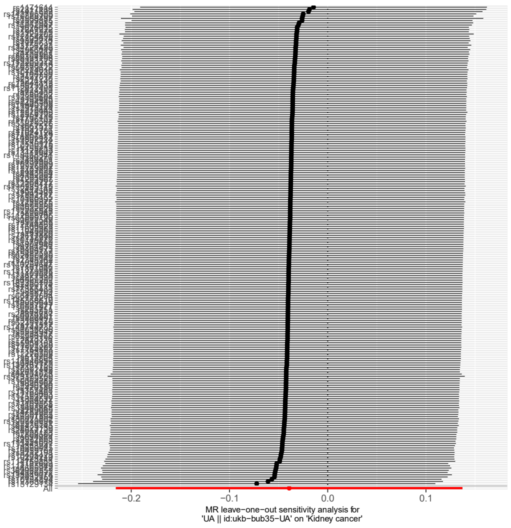

rs1512935
All
−0.2
−0.1
0.0
0.1
MR leave−one−out sensitivity analysis for
'UA || id:ukb−bub35−UA' on 'Kidney cancer'

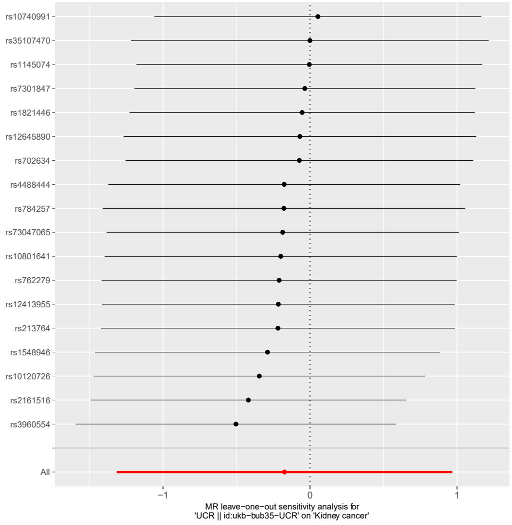

rs10740991
rs35107470
rs1145074
rs7301847
rs1821446
rs12645890
rs702634
rs4488444
rs784257
rs73047065
rs10801641
rs762279
rs12413955
rs213764
rs1548946
rs10120726
rs2161516
rs3960554
All
−1
0
1
MR leave−one−out sensitivity analysis for
'UCR || id:ukb−bub35−UCR' on 'Kidney cancer'

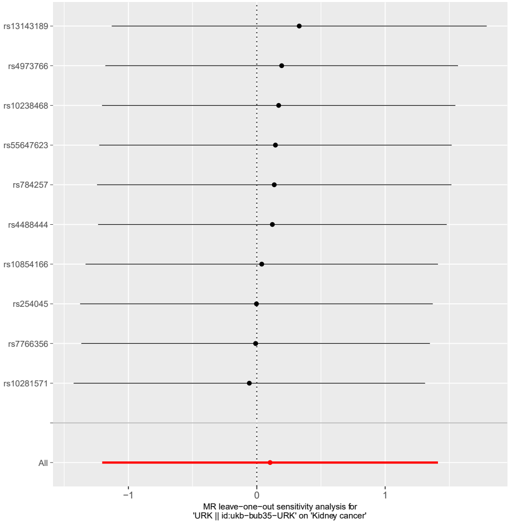

rs13143189
rs4973766
rs10238468
rs55647623
rs784257
rs4488444
rs10854166
rs254045
rs7766356
rs10281571
All
−1
0
1
MR leave−one−out sensitivity analysis for
'URK || id:ukb−bub35−URK' on 'Kidney cancer'

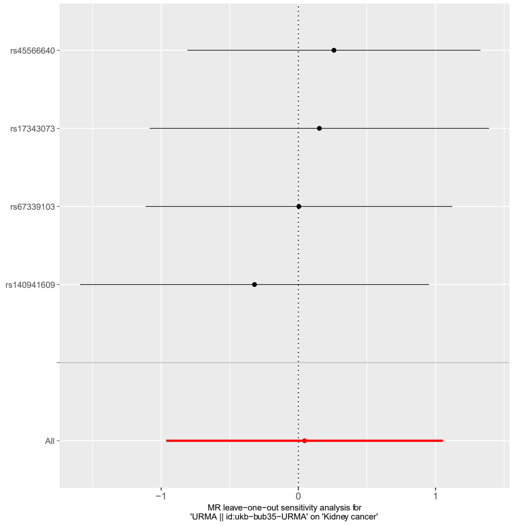

rs45566640
rs17343073
rs67339103
rs140941609
All
−1
0
1
MR leave−one−out sensitivity analysis for
'URMA || id:ukb−bub35−URMA' on 'Kidney cancer'

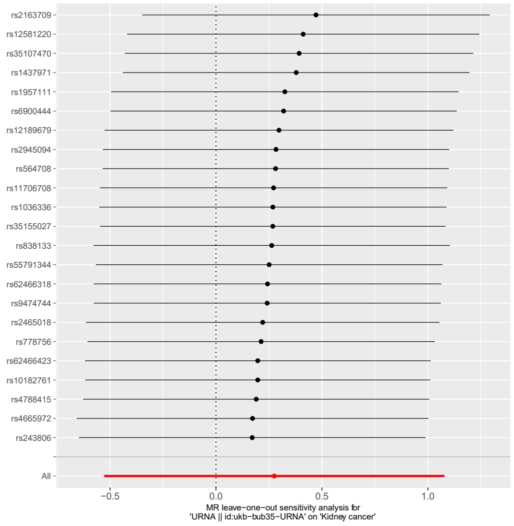

rs2163709
rs12581220
rs35107470
rs1437971
rs1957111
rs6900444
rs12189679
rs2945094
rs564708
rs11706708
rs1036336
rs35155027
rs838133
rs55791344
rs62466318
rs9474744
rs2465018
rs778756
rs62466423
rs10182761
rs4788415
rs4665972
rs243806
All
−0.5
0.0
0.5
1.0
MR leave−one−out sensitivity analysis for
'URNA || id:ukb−bub35−URNA' on 'Kidney cancer'

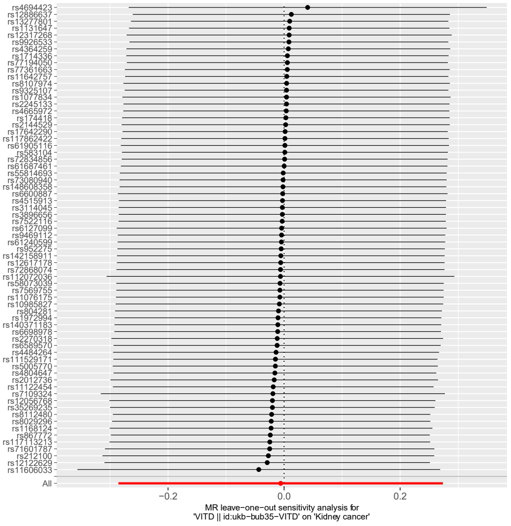

rs4694423
rs12886637
rs13277801
rs1131647
rs12317268
rs9926533
rs4364259
rs1714336
rs77194050
rs77361663
rs11642757
rs8107974
rs9325107
rs1077834
rs2245133
rs4665972
rs174418
rs2144529
rs17642290
rs117862422
rs61905116
rs583104
rs72834856
rs61687461
rs55814693
rs73080940
rs148608358
rs6600887
rs4515913
rs3114045
rs3896656
rs7522116
rs6127099
rs9469112
rs61240599
rs952275
rs142158911
rs12617178
rs72868074
rs112072036
rs58073039
rs7569755
rs11076175
rs10985827
rs804281
rs1972994
rs140371183
rs6698978
rs2270318
rs6589570
rs4484264
rs111529171
rs5005770
rs4804647
rs2012736
rs11122454
rs7109324
rs12056768
rs35269235
rs8112480
rs8029296
rs1168124
rs867772
rs117113213
rs71601787
rs212100
rs12122629
rs11606033
All
−0.2
0.0
0.2
MR leave−one−out sensitivity analysis for
'VITD || id:ukb−bub35−VITD' on 'Kidney cancer'

Forest plot of BCa

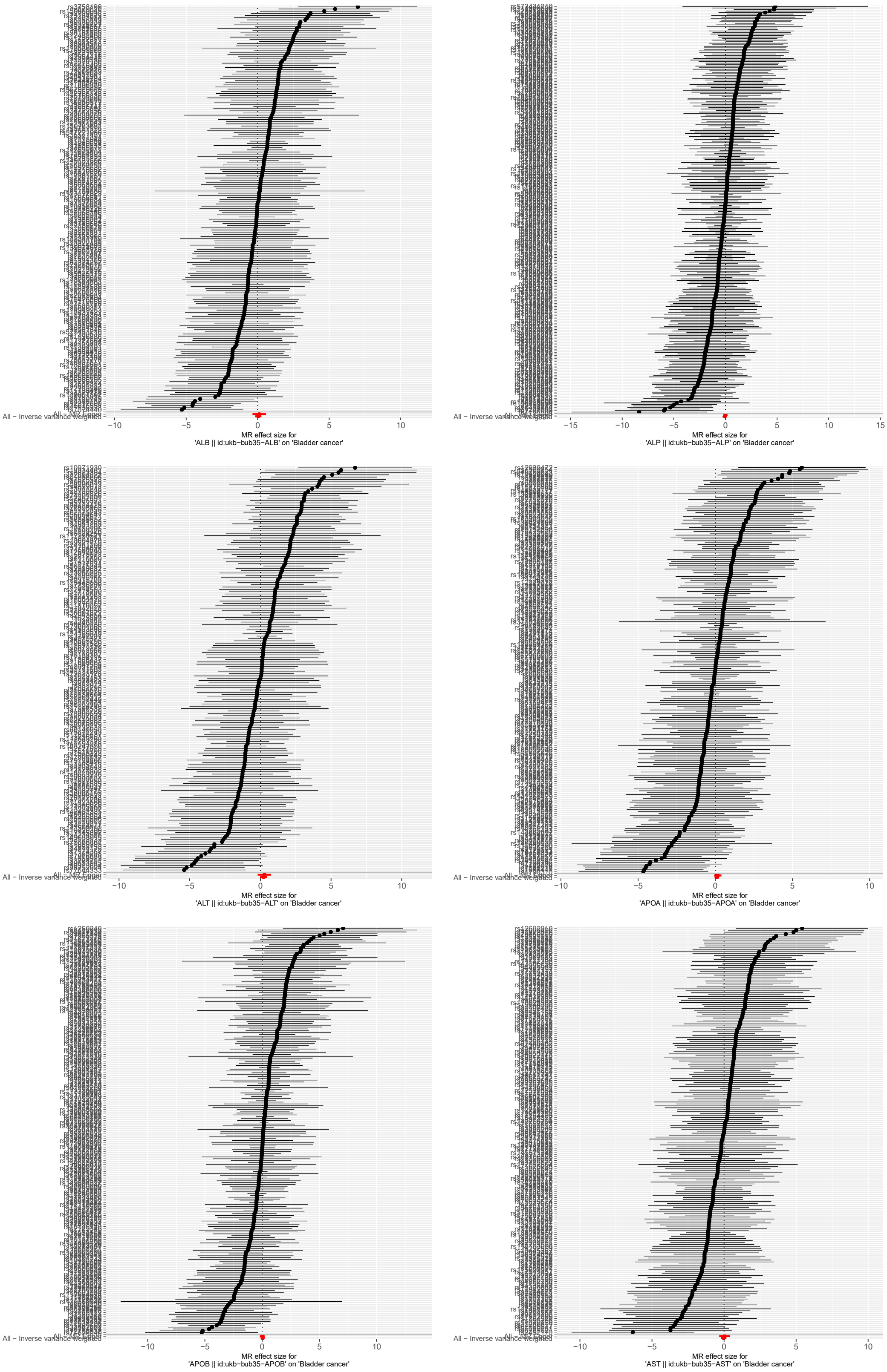

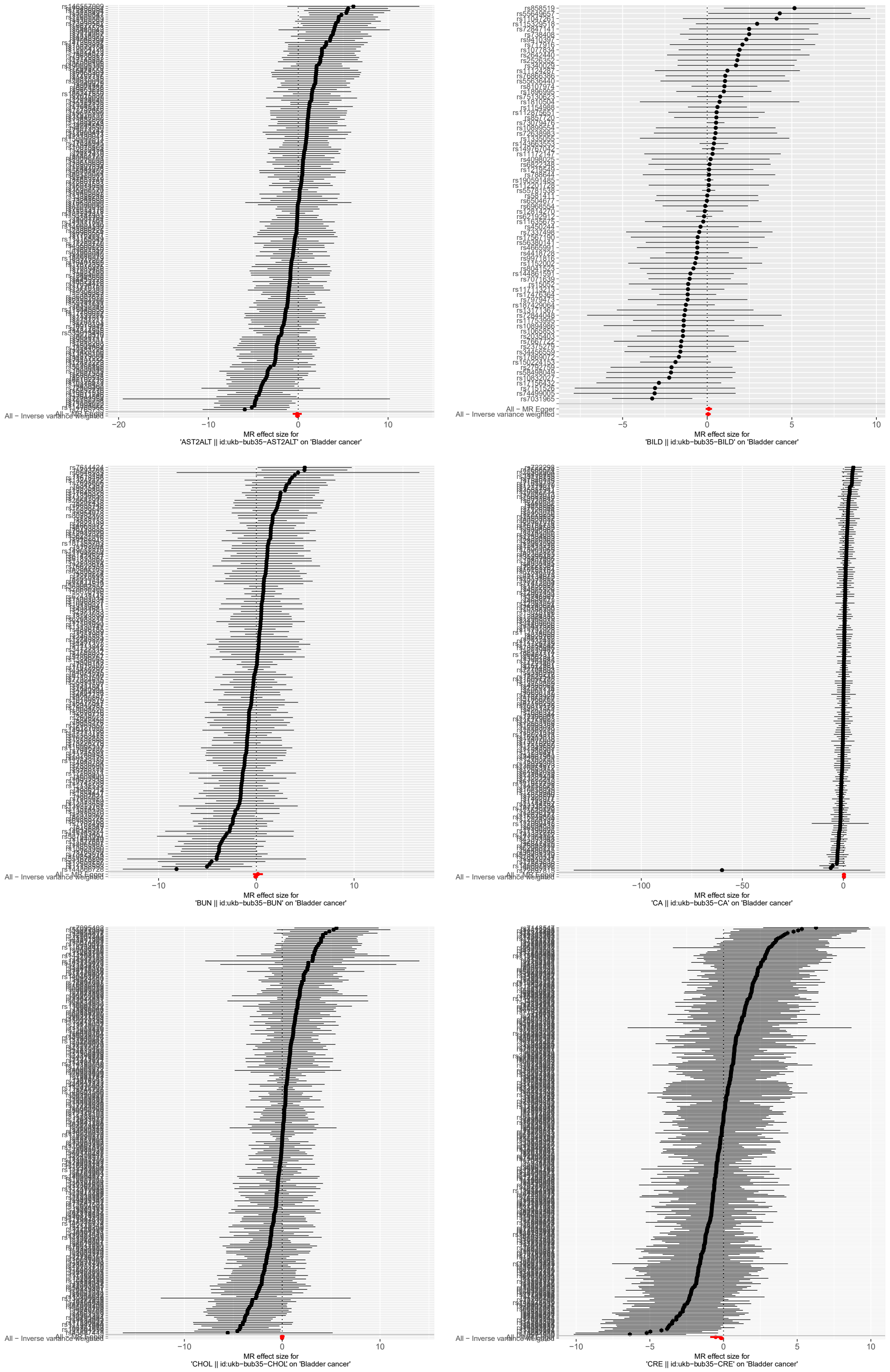

All − Inverse variance weighted
−20
−10
0
10
MR effect size for
'AST2ALT || id:ukb−bub35−AST2ALT' on 'Bladder cancer'
All − MR Egger
All − Inverse variance weighted
−5
0
5
10
MR effect size for
'BILD || id:ukb−bub35−BILD' on 'Bladder cancer'
All − Inverse variance weighted
−10
0
10
MR effect size for
'BUN || id:ukb−bub35−BUN' on 'Bladder cancer'
All − Inverse variance weighted
−100
−50
0
MR effect size for
'CA || id:ukb−bub35−CA' on 'Bladder cancer'
All − Inverse variance weighted
−10
0
10
MR effect size for
'CHOL || id:ukb−bub35−CHOL' on 'Bladder cancer'
All − Inverse variance weighted
−10
−5
0
5
10
MR effect size for
'CRE || id:ukb−bub35−CRE' on 'Bladder cancer'

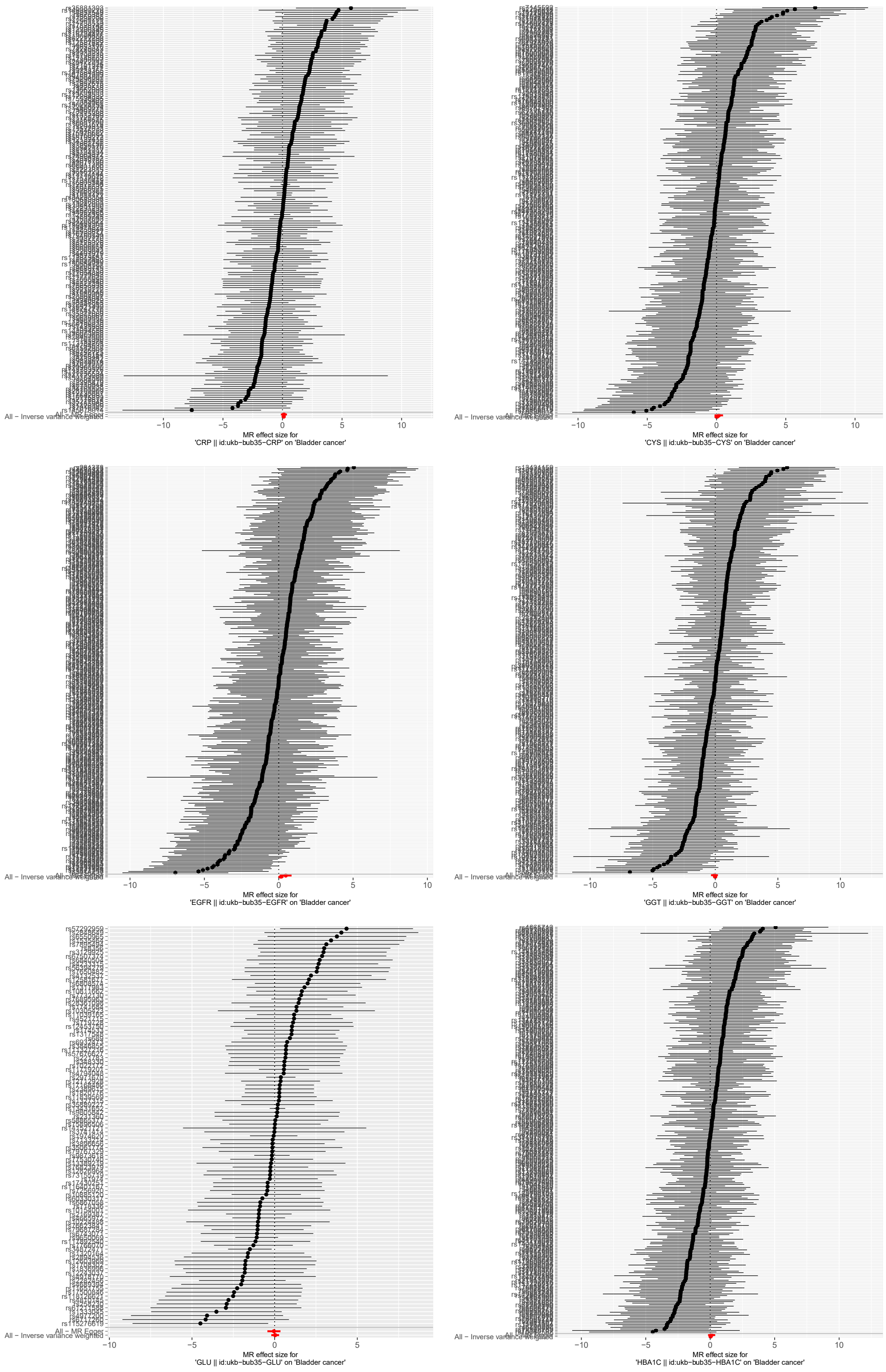
All − Inverse variance weighted
MR effect size for
'CRP || id:ukb−bub35−CRP' on 'Bladder cancer'
All − Inverse variance weighted
MR effect size for
'CYS || id:ukb−bub35−CYS' on 'Bladder cancer'
All − Inverse variance weighted
MR effect size for
'EGFR || id:ukb−bub35−EGFR' on 'Bladder cancer'
All − Inverse variance weighted
MR effect size for
'GGT || id:ukb−bub35−GGT' on 'Bladder cancer'
All − MR Egger
All − Inverse variance weighted
MR effect size for
'GLU || id:ukb−bub35−GLU' on 'Bladder cancer'
All − Inverse variance weighted
MR effect size for
'HBA1C || id:ukb−bub35−HBA1C' on 'Bladder cancer'

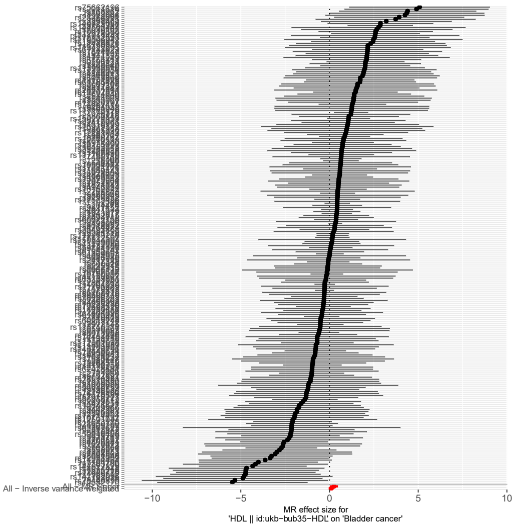

All − Inverse variance weighted
−10 −5 0 5 10
MR effect size for
'HDL || id:ukb−bub35−HDL' on 'Bladder cancer'

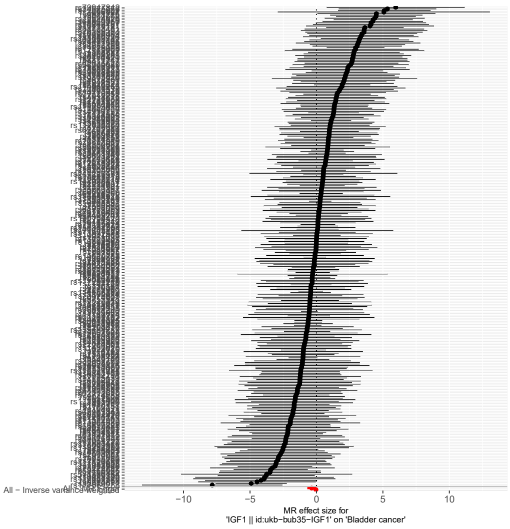

All − Inverse variance weighted
−10 −5 0 5 10
MR effect size for
'IGF1 || id:ukb−bub35−IGF1' on 'Bladder cancer'

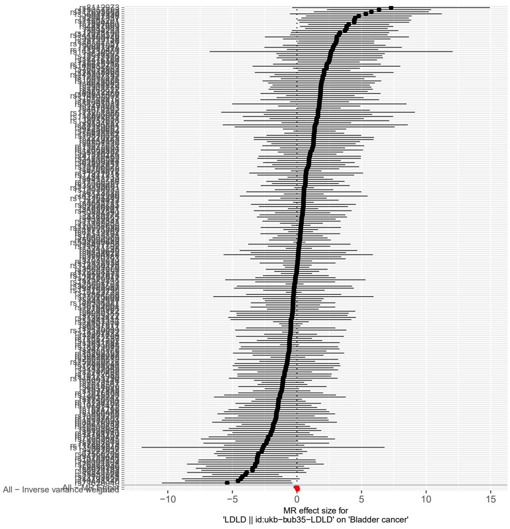

All − Inverse variance weighted
−10 −5 0 5 10 15
MR effect size for
'LDLD || id:ukb−bub35−LDLD' on 'Bladder cancer'

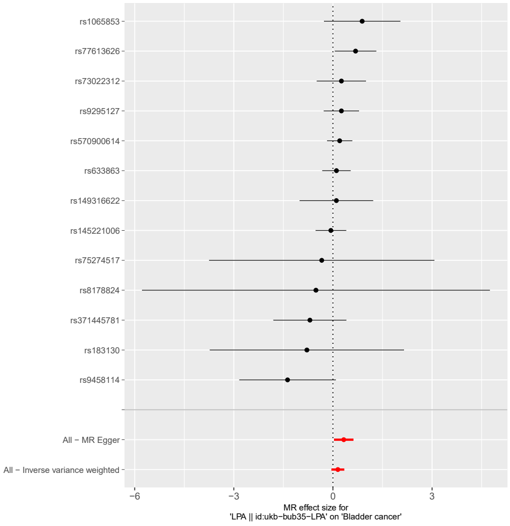

rs1065853
rs77613626
rs73022312
rs9295127
rs570900614
rs633863
rs149316622
rs145221006
rs75274517
rs8178824
rs371445781
rs183130
rs9458114
All − MR Egger
All − Inverse variance weighted
−6 −3 0 3
MR effect size for
'LPA || id:ukb−bub35−LPA' on 'Bladder cancer'

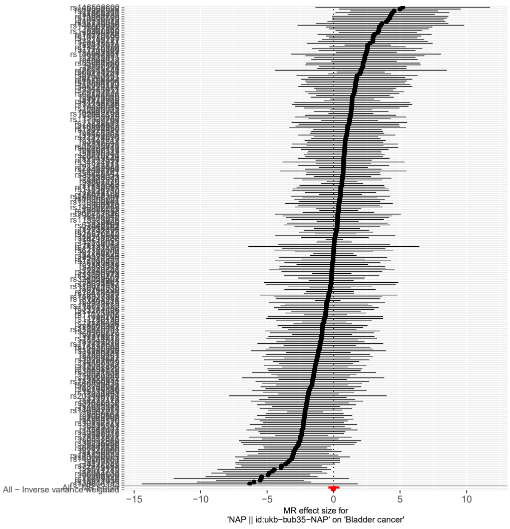

All − Inverse variance weighted
−15 −10 −5 0 5 10
MR effect size for
'NAP || id:ukb−bub35−NAP' on 'Bladder cancer'

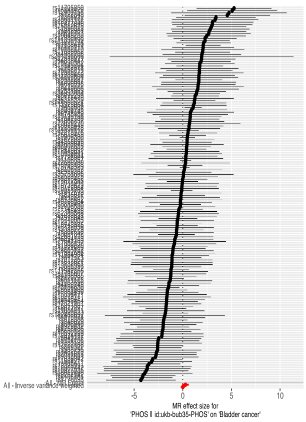

All − Inverse variance weighted
−5 0 5 10
MR effect size for
'PHOS || id:ukb−bub35−PHOS' on 'Bladder cancer'

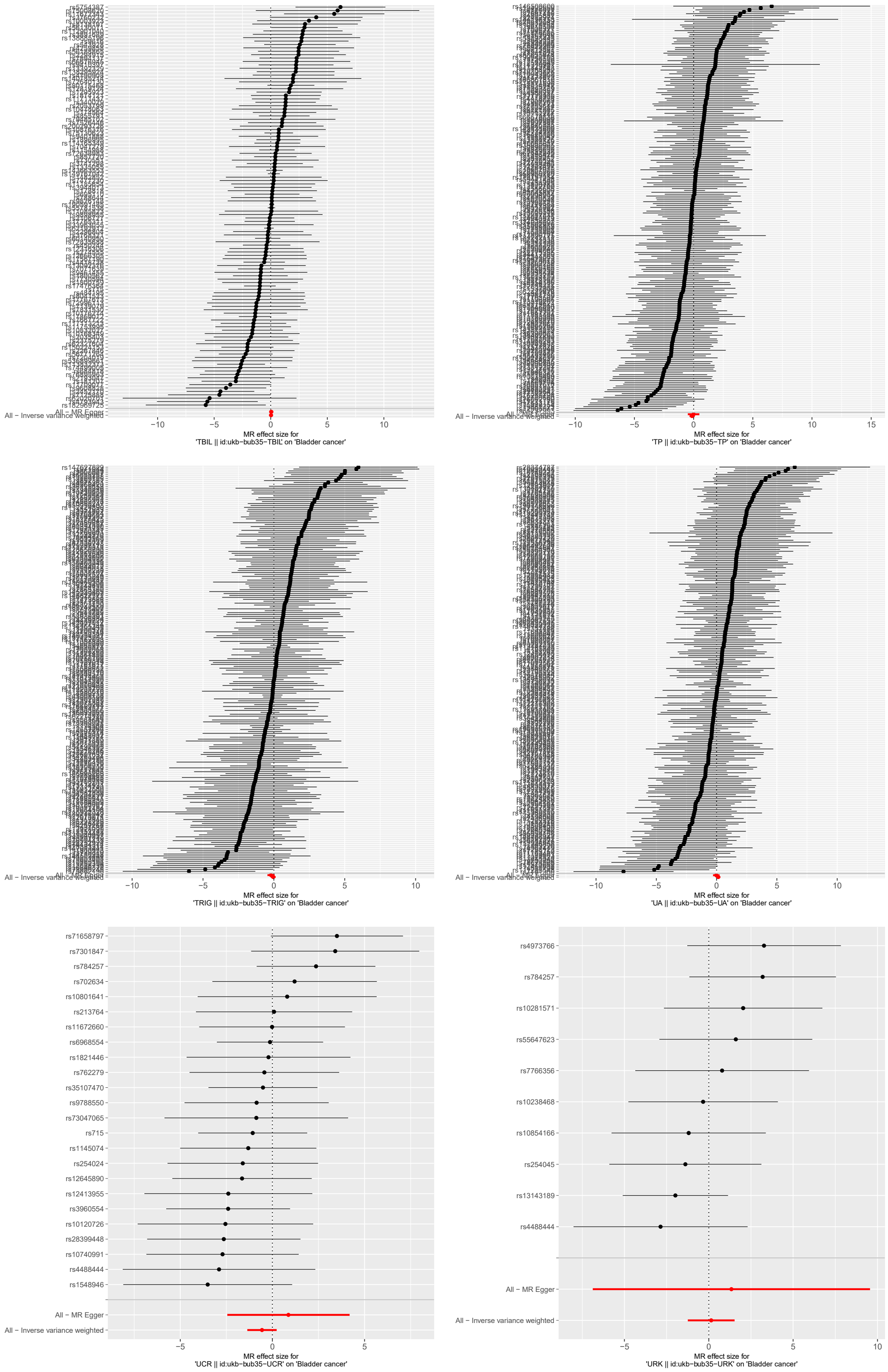

All − MR Egger
All − Inverse variance weighted
MR effect size for
'TBIL || id:ukb−bub35−TBIL' on 'Bladder cancer'
MR effect size for
'TP || id:ukb−bub35−TP' on 'Bladder cancer'
MR effect size for
'TRIG || id:ukb−bub35−TRIG' on 'Bladder cancer'
MR effect size for
'UA || id:ukb−bub35−UA' on 'Bladder cancer'
rs71658797
rs7301847
rs784257
rs702634
rs10801641
rs213764
rs11672660
rs6968554
rs1821446
rs762279
rs35107470
rs9788550
rs73047065
rs715
rs1145074
rs254024
rs12645890
rs12413955
rs3960554
rs10120726
rs28399448
rs10740991
rs4488444
rs1548946
MR effect size for
'UCR || id:ukb−bub35−UCR' on 'Bladder cancer'
rs4973766
rs784257
rs10281571
rs55647623
rs7766356
rs10238468
rs10854166
rs254045
rs13143189
rs4488444
MR effect size for
'URK || id:ukb−bub35−URK' on 'Bladder cancer'

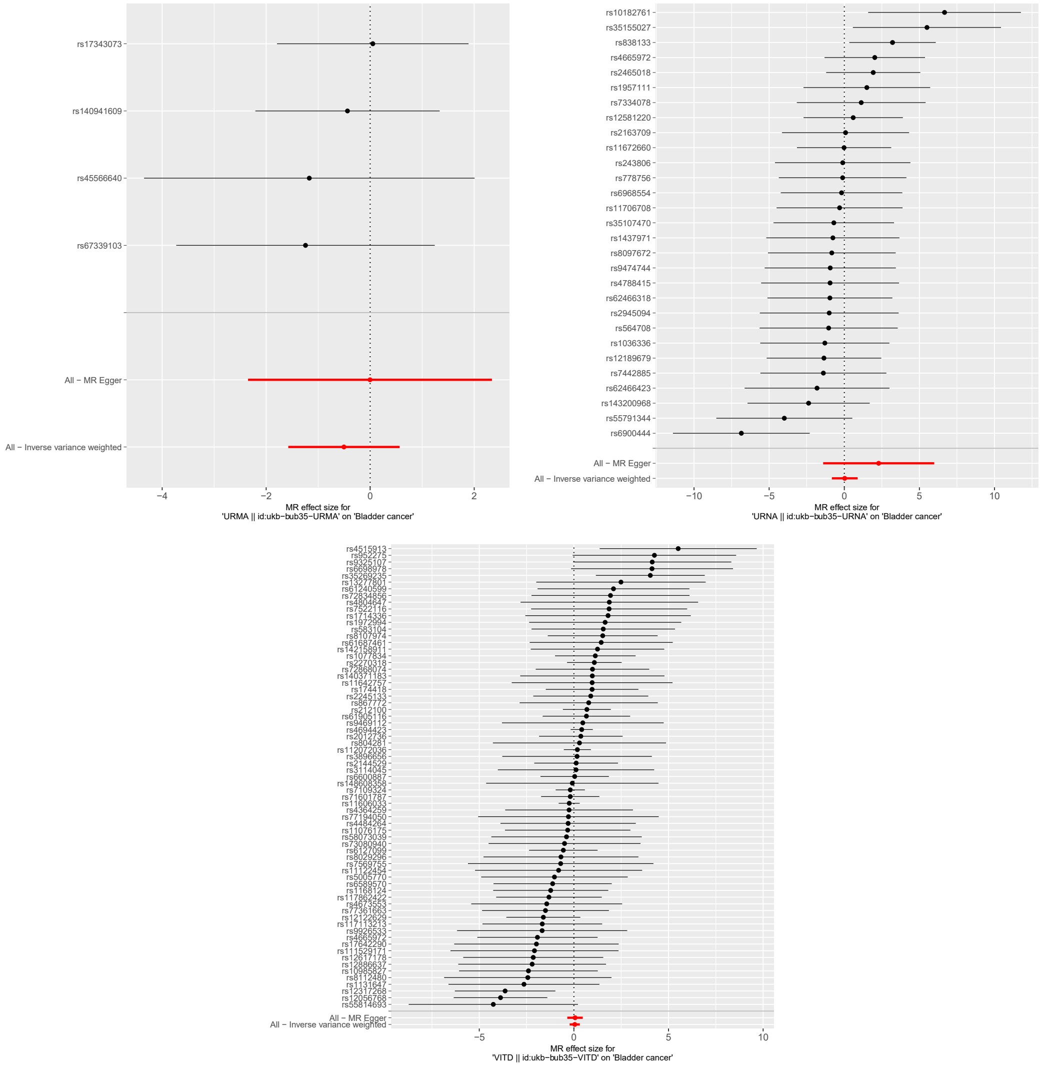

rs17343073
rs140941609
rs45566640
rs67339103
All − MR Egger
All − Inverse variance weighted
−4
−2
0
2
MR effect size for
'URMA || id:ukb−bub35−URMA' on 'Bladder cancer'
rs10182761
rs35155027
rs838133
rs4665972
rs2465018
rs1957111
rs7334078
rs12581220
rs2163709
rs11672660
rs243806
rs778756
rs6968554
rs11706708
rs35107470
rs1437971
rs8097672
rs9474744
rs4788415
rs62466318
rs2945094
rs564708
rs1036336
rs12189679
rs7442885
rs62466423
rs143200968
rs55791344
rs6900444
All − MR Egger
All − Inverse variance weighted
−10
−5
0
5
10
MR effect size for
'URNA || id:ukb−bub35−URNA' on 'Bladder cancer'
All − MR Egger
All − Inverse variance weighted
−5
0
5
10
MR effect size for
'VITD || id:ukb−bub35−VITD' on 'Bladder cancer'

Forest plot of PCa

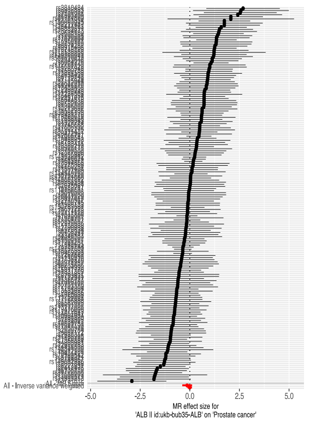

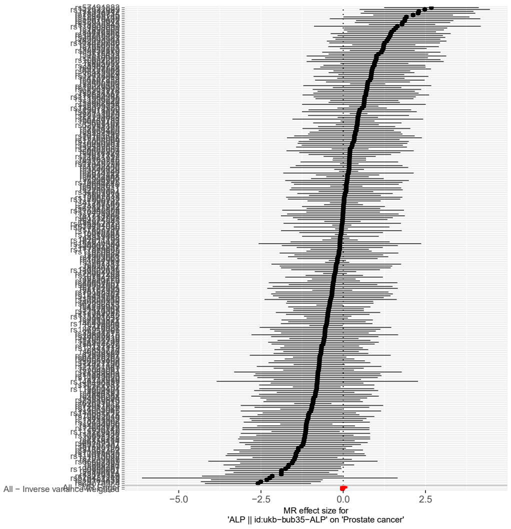

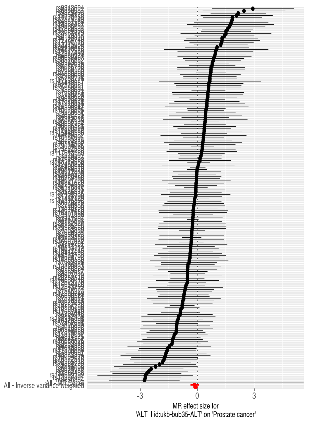

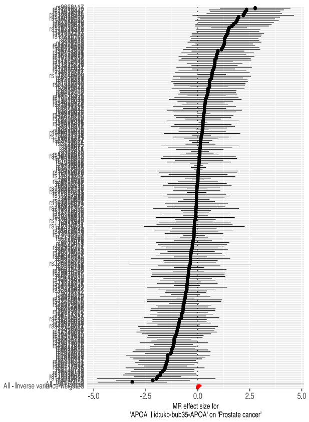

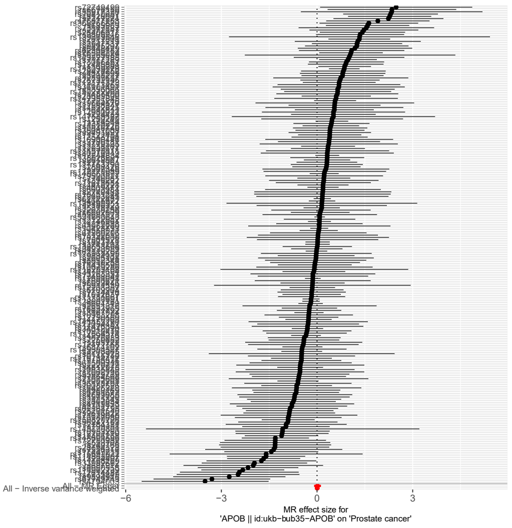

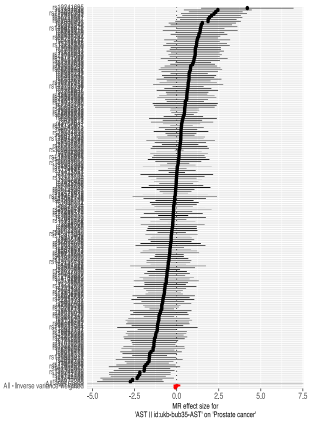

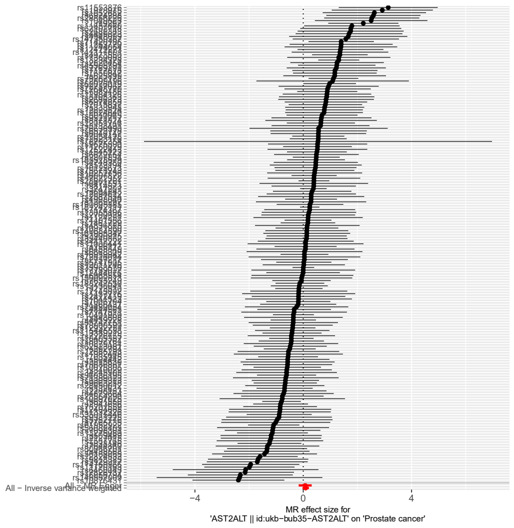
All − Inverse variance weighted
−4
0
4
MR effect size for
'AST2ALT || id:ukb−bub35−AST2ALT' on 'Prostate cancer'

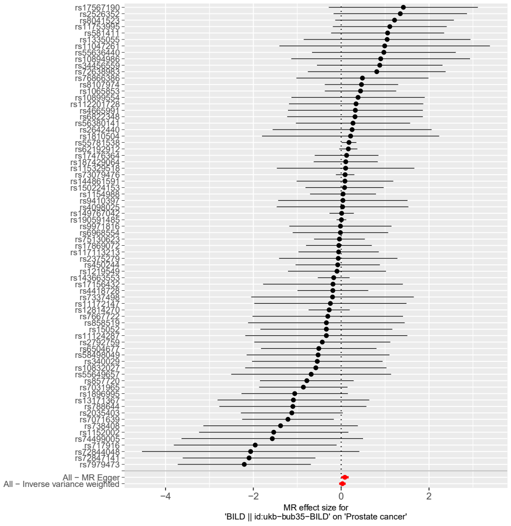
All − MR Egger
All − Inverse variance weighted
−4
−2
0
2
MR effect size for
'BILD || id:ukb−bub35−BILD' on 'Prostate cancer'

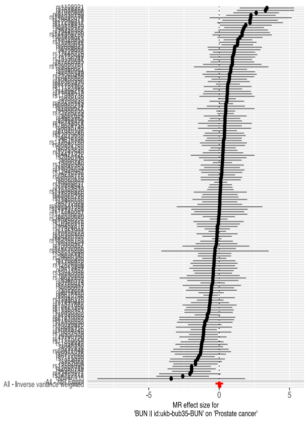
All − Inverse variance weighted
-5
0
5
MR effect size for
'BUN || id:ukb-bub35-BUN' on 'Prostate cancer'

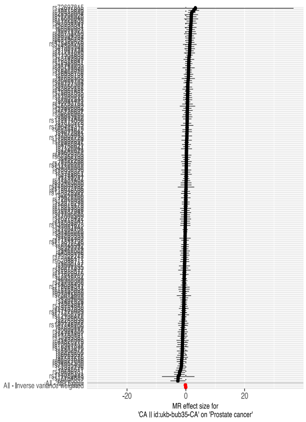
All − Inverse variance weighted
-20
0
20
MR effect size for
'CA || id:ukb-bub35-CA' on 'Prostate cancer'

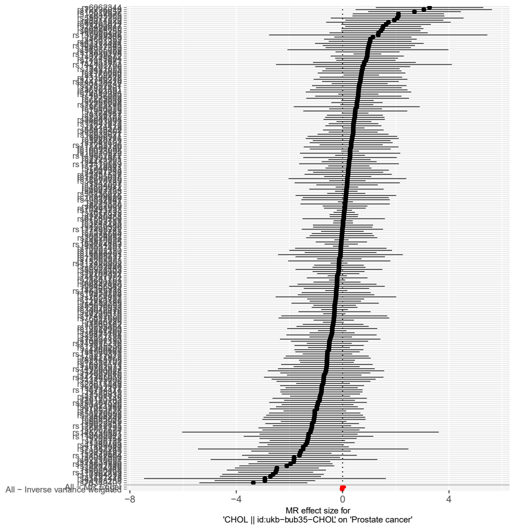
All − Inverse variance weighted
−8
−4
0
4
MR effect size for
'CHOL || id:ukb−bub35−CHOL' on 'Prostate cancer'

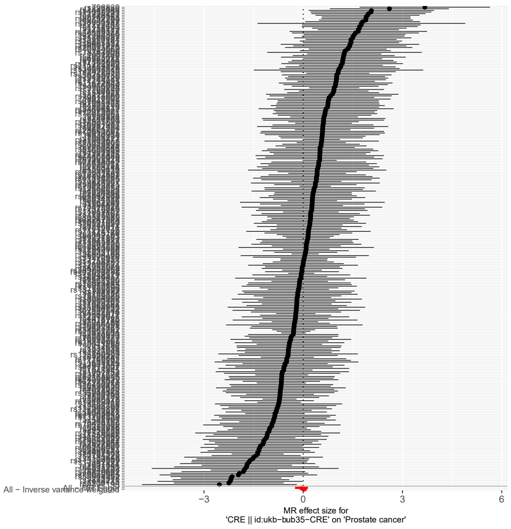
All − Inverse variance weighted
−3
0
3
6
MR effect size for
'CRE || id:ukb−bub35−CRE' on 'Prostate cancer'

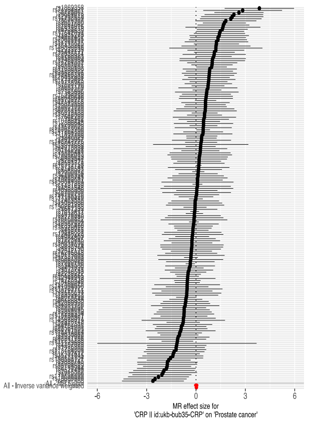
All - Inverse variance weighted
-6
-3
0
3
6
MR effect size for
'CRP || id:ukb-bub35-CRP' on 'Prostate cancer'

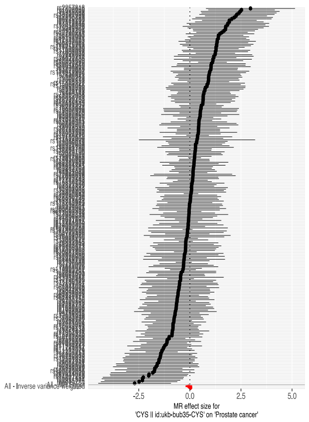
All - Inverse variance weighted
-2.5
0.0
2.5
5.0
MR effect size for
'CYS || id:ukb-bub35-CYS' on 'Prostate cancer'

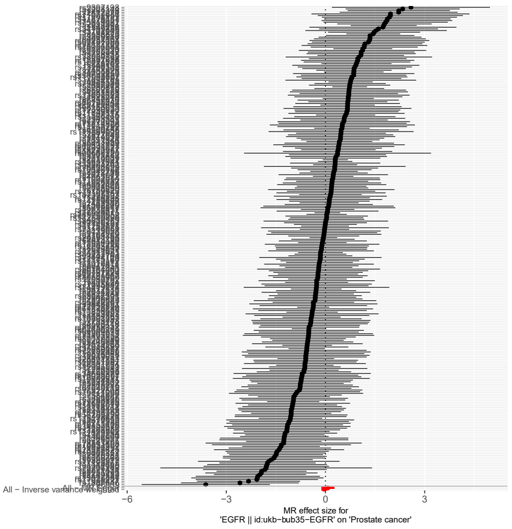
All − Inverse variance weighted
−6
−3
0
3
MR effect size for
'EGFR || id:ukb−bub35−EGFR' on 'Prostate cancer'

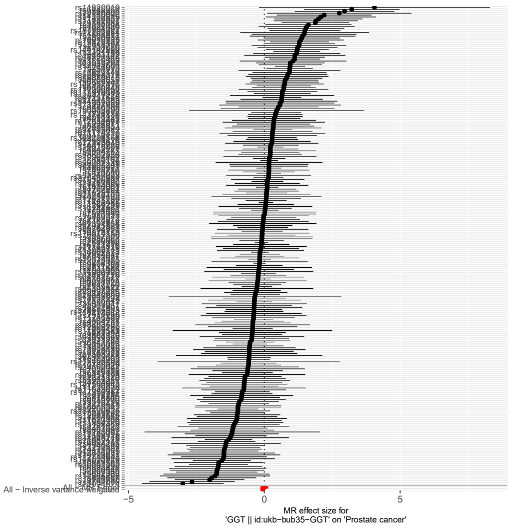
All − Inverse variance weighted
−5
0
5
MR effect size for
'GGT || id:ukb−bub35−GGT' on 'Prostate cancer'

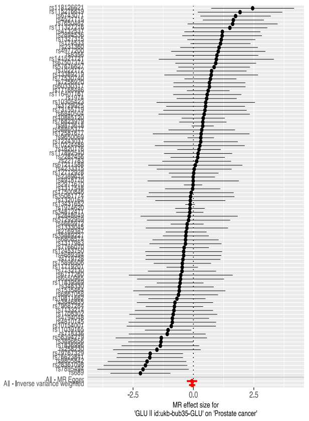
All - MR Egger
All - Inverse variance weighted
-2.5
0.0
2.5
MR effect size for
'GLU || id:ukb-bub35-GLU' on 'Prostate cancer'

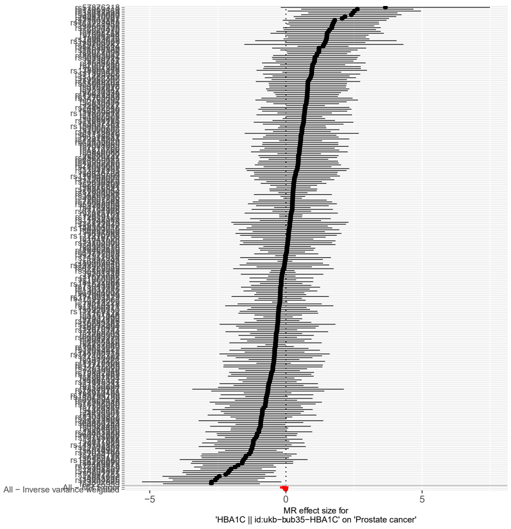
All − Inverse variance weighted
−5
0
5
MR effect size for
'HBA1C || id:ukb−bub35−HBA1C' on 'Prostate cancer'

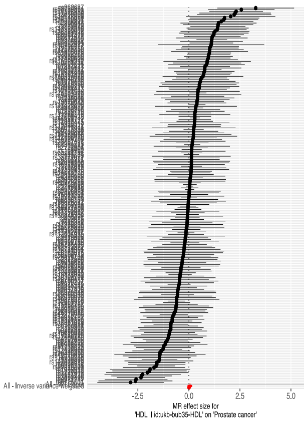
All - Inverse variance weighted
-2.5
0.0
2.5
5.0
MR effect size for
'HDL || id:ukb-bub35-HDL' on 'Prostate cancer'

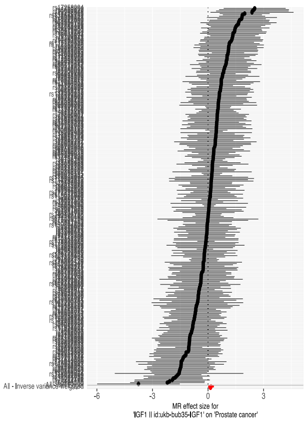
All - Inverse variance weighted
-6
-3
0
3
MR effect size for
'IGF1 || id:ukb-bub35-IGF1' on 'Prostate cancer'

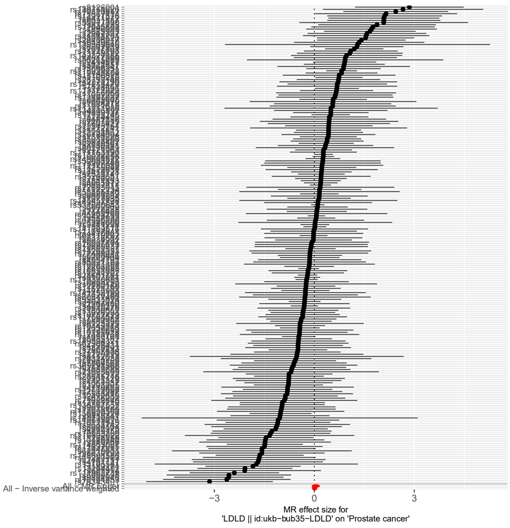
All − Inverse variance weighted
−3
0
3
MR effect size for
'LDLD || id:ukb−bub35−LDLD' on 'Prostate cancer'

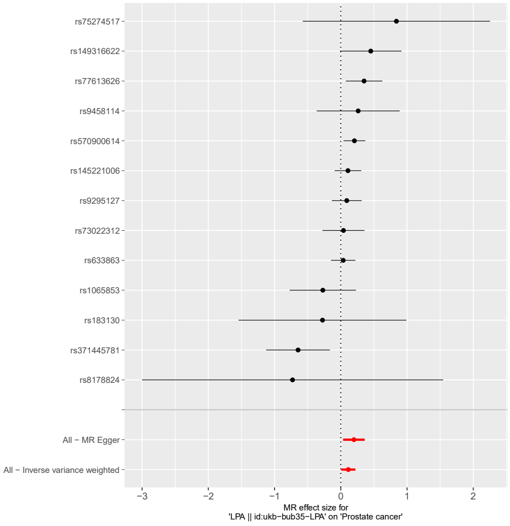
rs75274517
rs149316622
rs77613626
rs9458114
rs570900614
rs145221006
rs9295127
rs73022312
rs633863
rs1065853
rs183130
rs371445781
rs8178824
All − MR Egger
All − Inverse variance weighted
−3
−2
−1
0
1
2
MR effect size for
'LPA || id:ukb−bub35−LPA' on 'Prostate cancer'

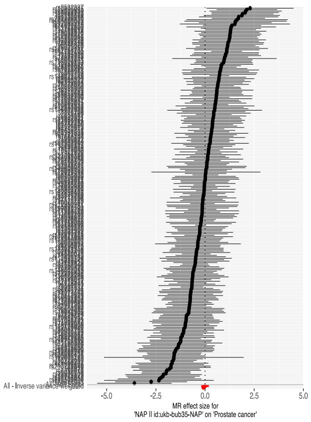
All - Inverse variance weighted
-5.0
-2.5
0.0
2.5
5.0
MR effect size for
'NAP || id:ukb-bub35-NAP' on 'Prostate cancer'

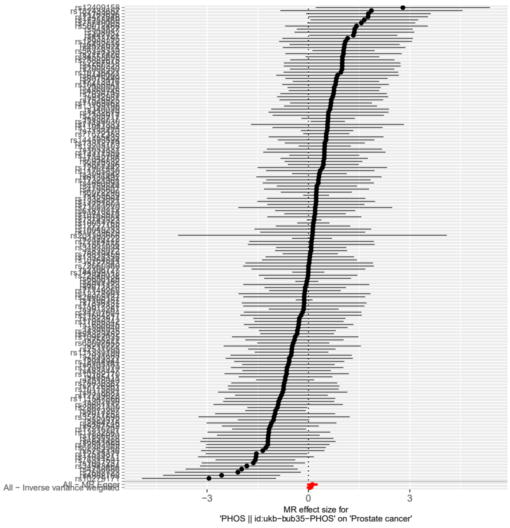
All − Inverse variance weighted
−3
0
3
MR effect size for
'PHOS || id:ukb−bub35−PHOS' on 'Prostate cancer'

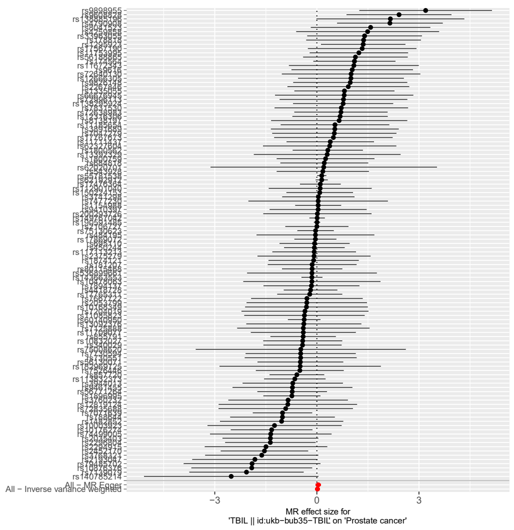
All – MR Egger
All – Inverse variance weighted
−3
0
3
MR effect size for
'TBIL || id:ukb−bub35−TBIL' on 'Prostate cancer'

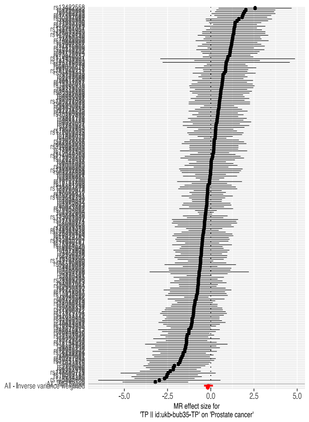
−5.0
−2.5
0.0
2.5
5.0
MR effect size for
'TP || id:ukb-bub35-TP' on 'Prostate cancer'

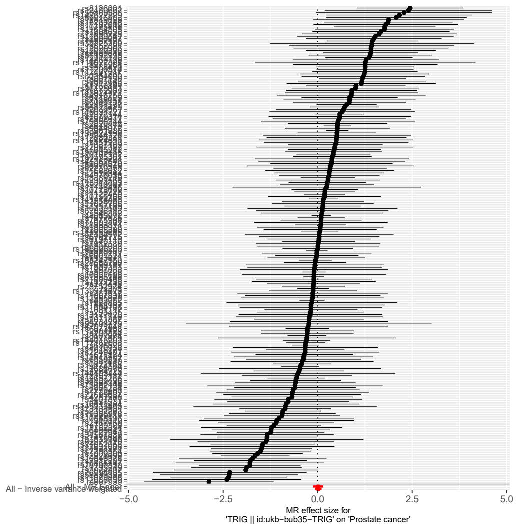
−5.0
−2.5
0.0
2.5
5.0
MR effect size for
'TRIG || id:ukb−bub35−TRIG' on 'Prostate cancer'

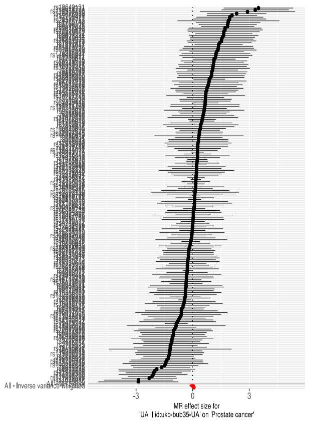
−3
0
3
MR effect size for
'UA || id:ukb-bub35-UA' on 'Prostate cancer'

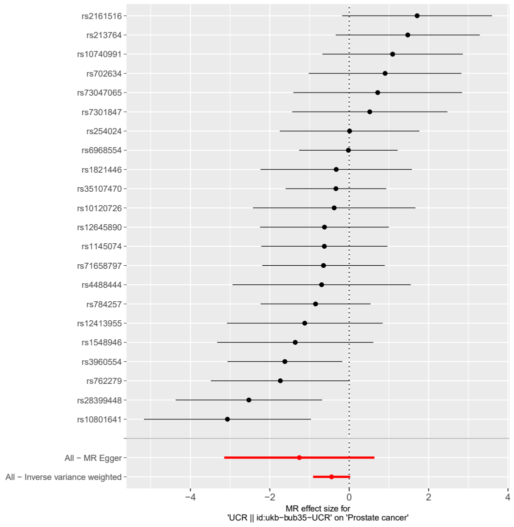
rs2161516
rs213764
rs10740991
rs702634
rs73047065
rs7301847
rs254024
rs6968554
rs1821446
rs35107470
rs10120726
rs12645890
rs1145074
rs71658797
rs4488444
rs784257
rs12413955
rs1548946
rs3960554
rs762279
rs28399448
rs10801641
All − MR Egger
All − Inverse variance weighted
−4
−2
0
2
4
MR effect size for
'UCR || id:ukb−bub35−UCR' on 'Prostate cancer'

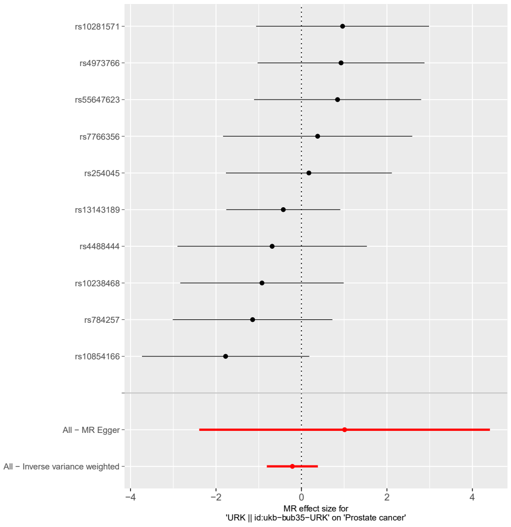
rs10281571
rs4973766
rs55647623
rs7766356
rs254045
rs13143189
rs4488444
rs10238468
rs784257
rs10854166
All − MR Egger
All − Inverse variance weighted
−4
−2
0
2
4
MR effect size for
'URK || id:ukb−bub35−URK' on 'Prostate cancer'

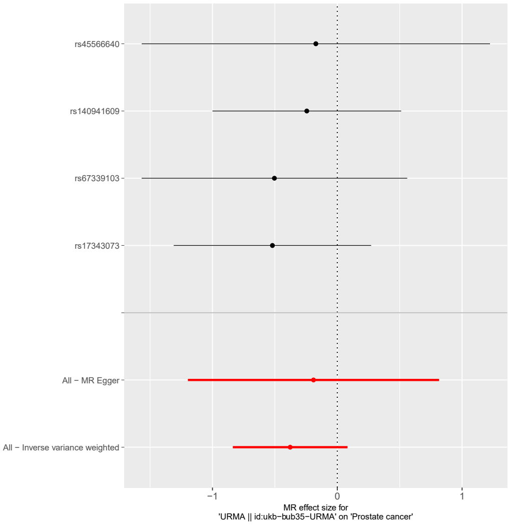

rs45566640
rs140941609
rs67339103
rs17343073
All − MR Egger
All − Inverse variance weighted
−1
0
1
MR effect size for
'URMA || id:ukb−bub35−URMA' on 'Prostate cancer'

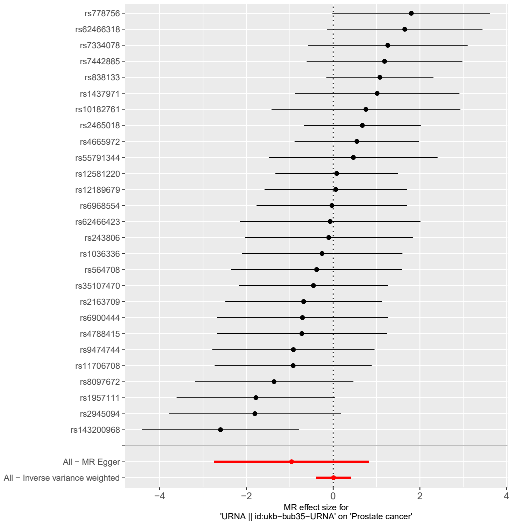

rs778756
rs62466318
rs7334078
rs7442885
rs838133
rs1437971
rs10182761
rs2465018
rs4665972
rs55791344
rs12581220
rs12189679
rs6968554
rs62466423
rs243806
rs1036336
rs564708
rs35107470
rs2163709
rs6900444
rs4788415
rs9474744
rs11706708
rs8097672
rs1957111
rs2945094
rs143200968
All − MR Egger
All − Inverse variance weighted
−4
−2
0
2
4
MR effect size for
'URNA || id:ukb−bub35−URNA' on 'Prostate cancer'

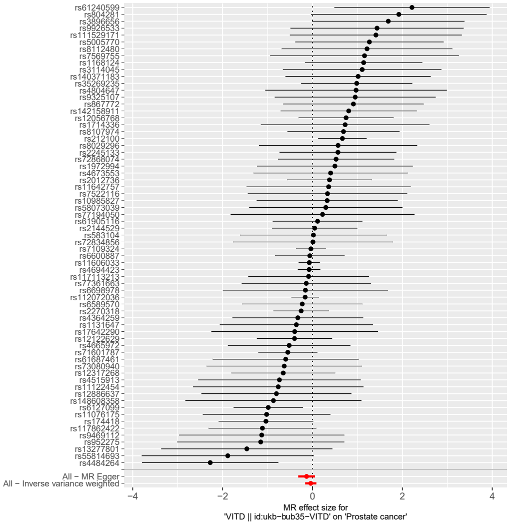

rs61240599
rs804281
rs3896656
rs9926533
rs111529171
rs5005770
rs8112480
rs7569755
rs1168124
rs3114045
rs140371183
rs35269235
rs4804647
rs9325107
rs867772
rs142158911
rs12056768
rs1714336
rs8107974
rs212100
rs8029296
rs2245133
rs72868074
rs1972994
rs4673553
rs2012736
rs11642757
rs7522116
rs10985827
rs58073039
rs77194050
rs61905116
rs2144529
rs583104
rs72834856
rs7109324
rs6600887
rs11606033
rs4694423
rs117113213
rs77361663
rs6698978
rs112072036
rs6589570
rs2270318
rs4364259
rs1131647
rs17642290
rs12122629
rs4665972
rs71601787
rs61687461
rs73080940
rs12317268
rs4515913
rs11122454
rs12886637
rs148608358
rs6127099
rs11076175
rs174418
rs117862422
rs9469112
rs952275
rs13277801
rs55814693
rs4484264
All − MR Egger
All − Inverse variance weighted
−4
−2
0
2
4
MR effect size for
'VITD || id:ukb−bub35−VITD' on 'Prostate cancer'

Forest plot of RPCa

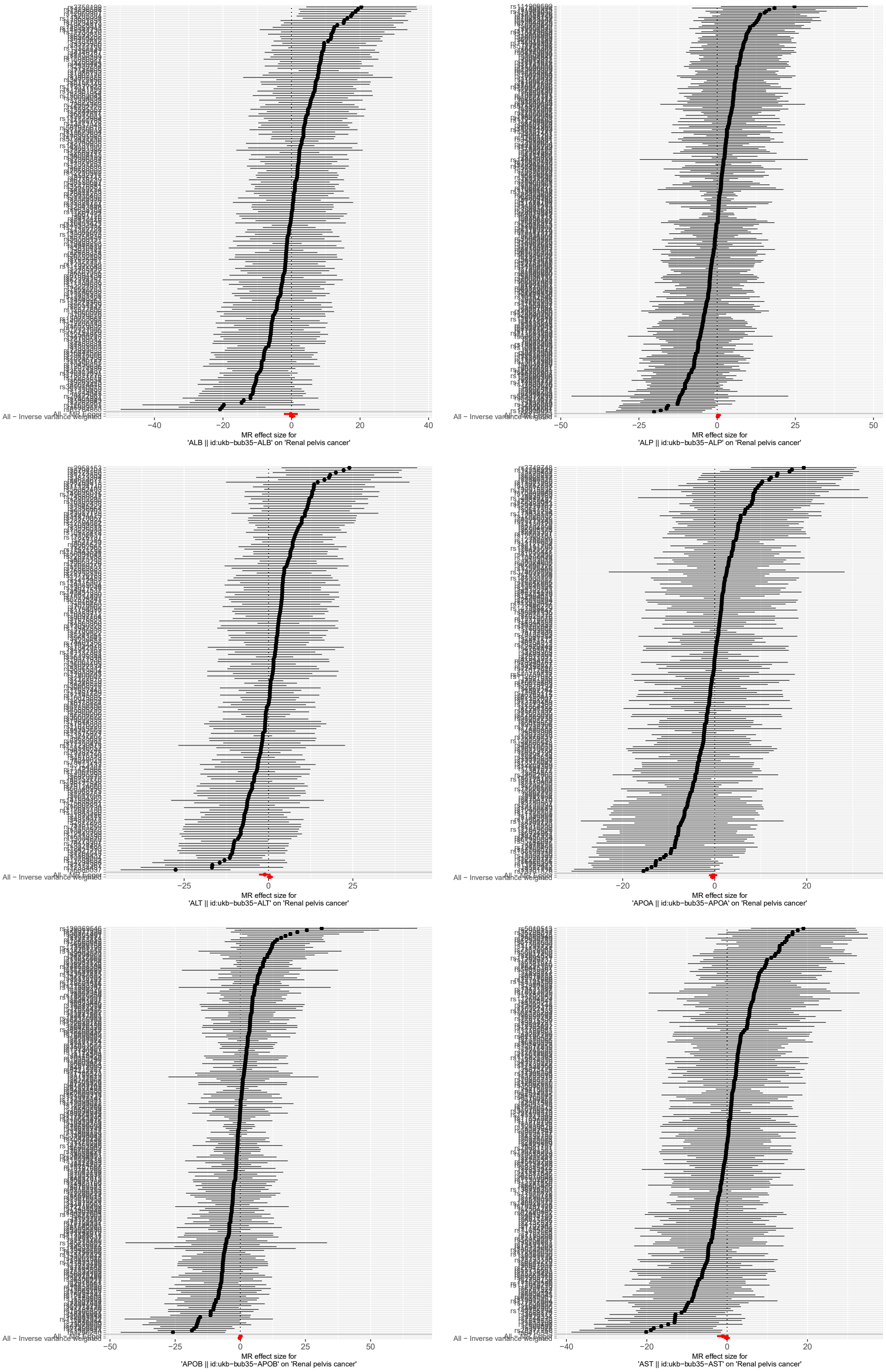

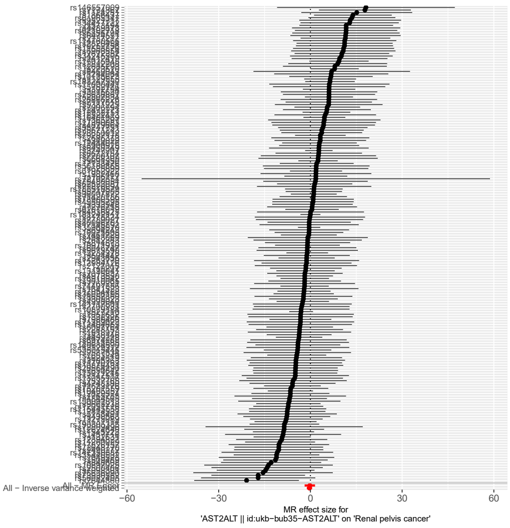
All − Inverse variance weighted
−60
−30
0
30
60
MR effect size for
'AST2ALT || id:ukb−bub35−AST2ALT' on 'Renal pelvis cancer'

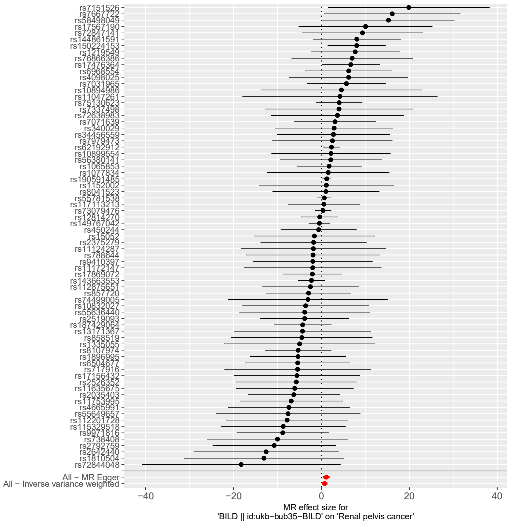
All − MR Egger
All − Inverse variance weighted
−40
−20
0
20
40
MR effect size for
'BILD || id:ukb−bub35−BILD' on 'Renal pelvis cancer'

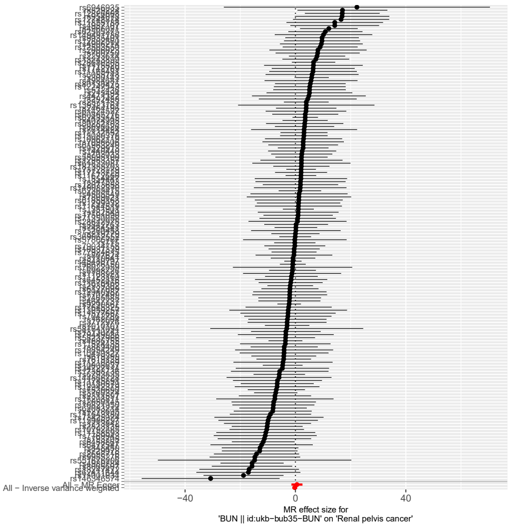
All − Inverse variance weighted
−40
0
40
MR effect size for
'BUN || id:ukb−bub35−BUN' on 'Renal pelvis cancer'

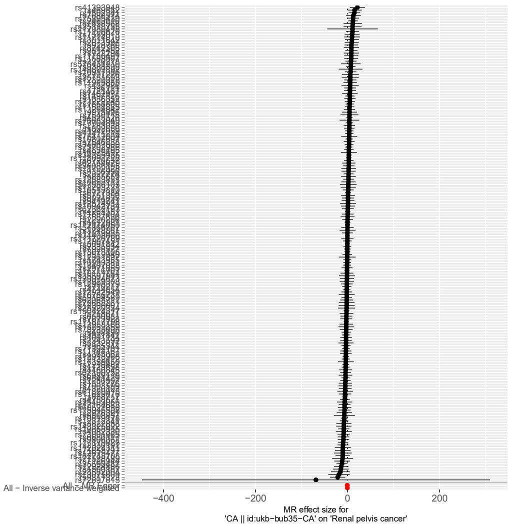
All − Inverse variance weighted
−400
−200
0
200
MR effect size for
'CA || id:ukb−bub35−CA' on 'Renal pelvis cancer'

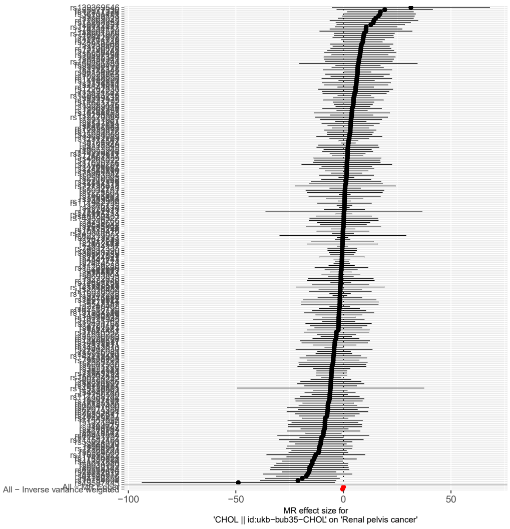
All − Inverse variance weighted
−100
−50
0
50
MR effect size for
'CHOL || id:ukb−bub35−CHOL' on 'Renal pelvis cancer'

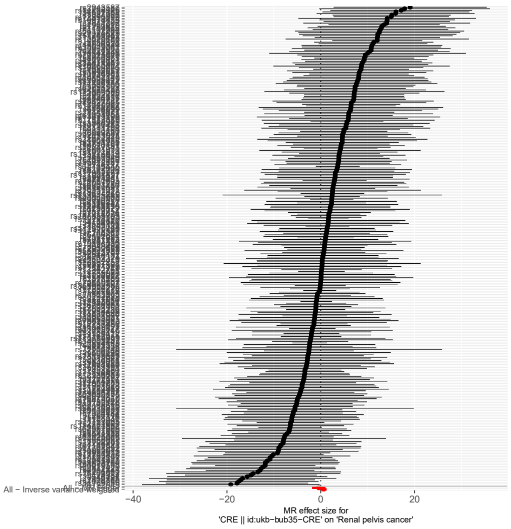
All − Inverse variance weighted
−40
−20
0
20
MR effect size for
'CRE || id:ukb−bub35−CRE' on 'Renal pelvis cancer'

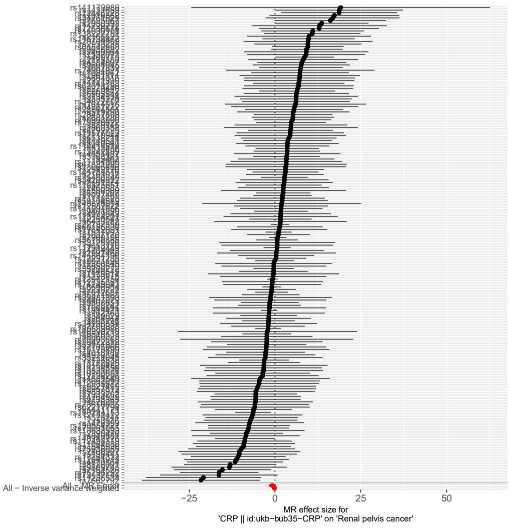
All − Inverse variance weighted
−25
0
25
50
MR effect size for
'CRP || id:ukb-bub35-CRP' on 'Renal pelvis cancer'

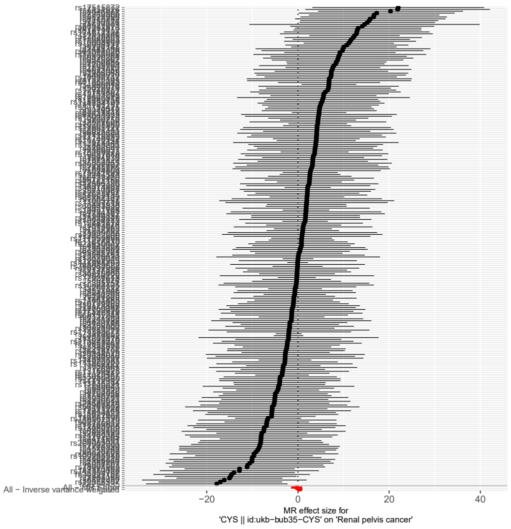
All − Inverse variance weighted
−20
0
20
40
MR effect size for
'CYS || id:ukb-bub35-CYS' on 'Renal pelvis cancer'

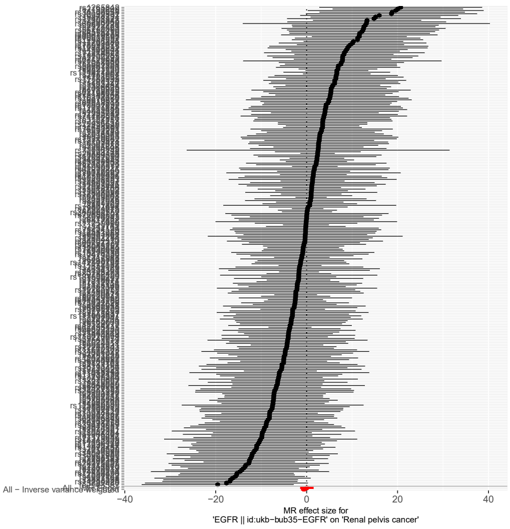
All − Inverse variance weighted
−40
−20
0
20
40
MR effect size for
'EGFR || id:ukb-bub35-EGFR' on 'Renal pelvis cancer'

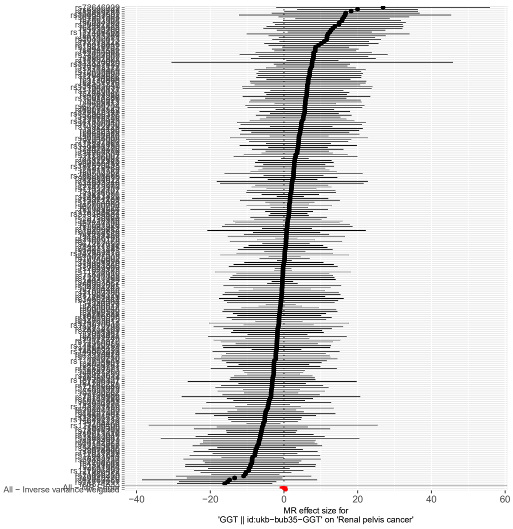
All − Inverse variance weighted
−40
−20
0
20
40
60
MR effect size for
'GGT || id:ukb-bub35-GGT' on 'Renal pelvis cancer'

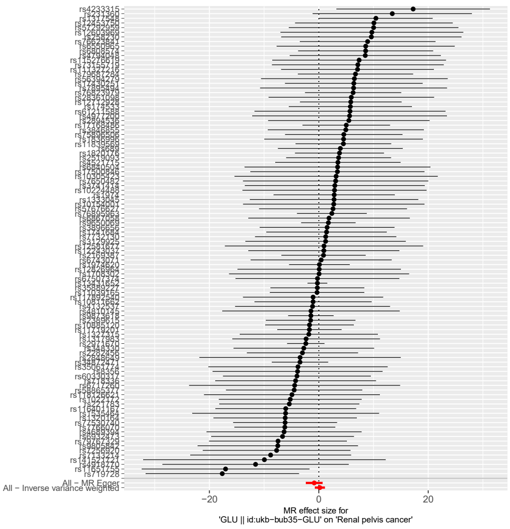
All − MR Egger
All − Inverse variance weighted
−20
0
20
MR effect size for
'GLU || id:ukb-bub35-GLU' on 'Renal pelvis cancer'

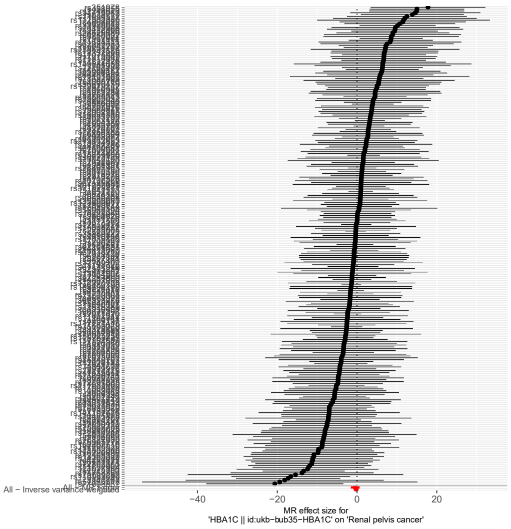
All − Inverse variance weighted
−40
−20
0
20
MR effect size for
'HBA1C || id:ukb-bub35-HBA1C' on 'Renal pelvis cancer'

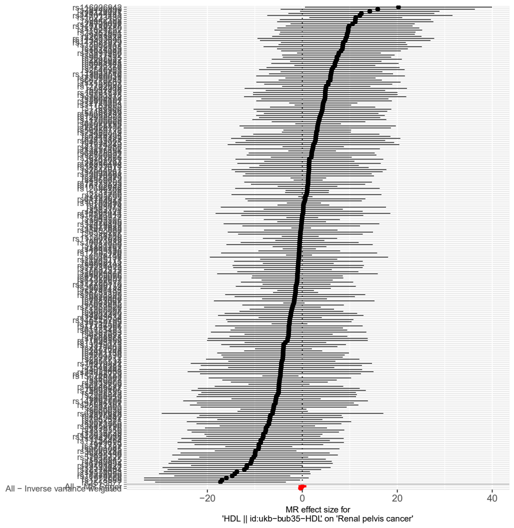
All − Inverse variance weighted
−20
0
20
40
MR effect size for
'HDL || id:ukb−bub35−HDL' on 'Renal pelvis cancer'

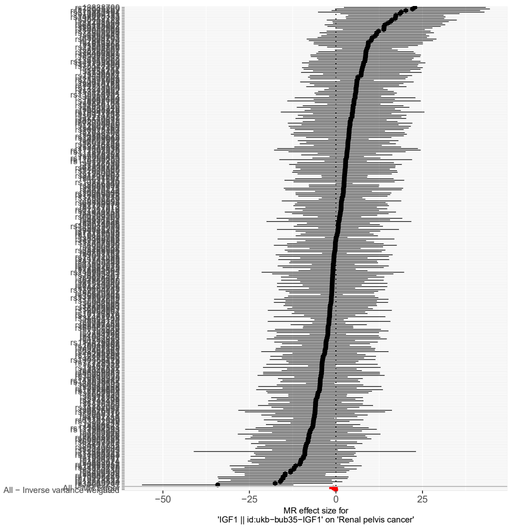
All − Inverse variance weighted
−50
−25
0
25
MR effect size for
'IGF1 || id:ukb−bub35−IGF1' on 'Renal pelvis cancer'

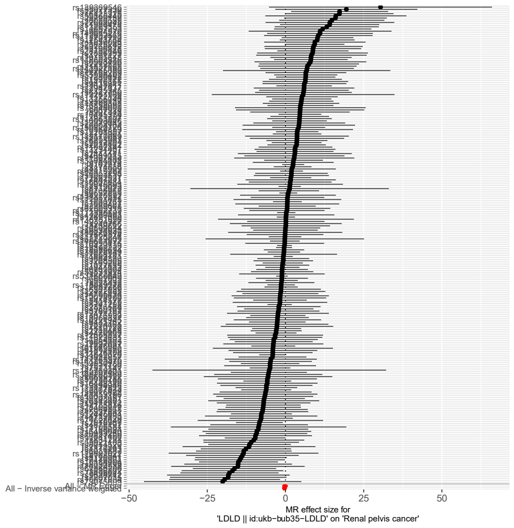
All − Inverse variance weighted
−50
−25
0
25
50
MR effect size for
'LDLD || id:ukb−bub35−LDLD' on 'Renal pelvis cancer'

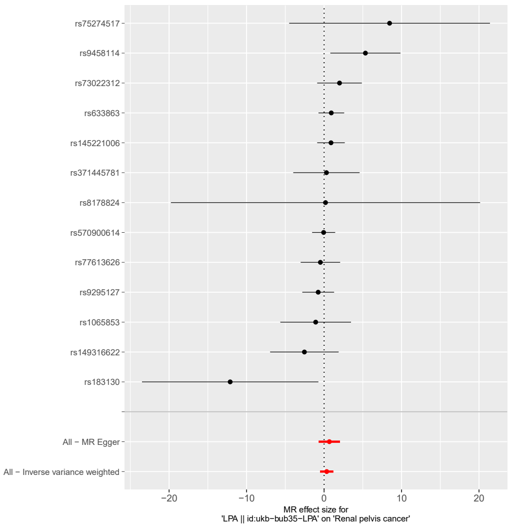
rs75274517
rs9458114
rs73022312
rs633863
rs145221006
rs371445781
rs8178824
rs570900614
rs77613626
rs9295127
rs1065853
rs149316622
rs183130
All − MR Egger
All − Inverse variance weighted
−20
−10
0
10
20
MR effect size for
'LPA || id:ukb−bub35−LPA' on 'Renal pelvis cancer'

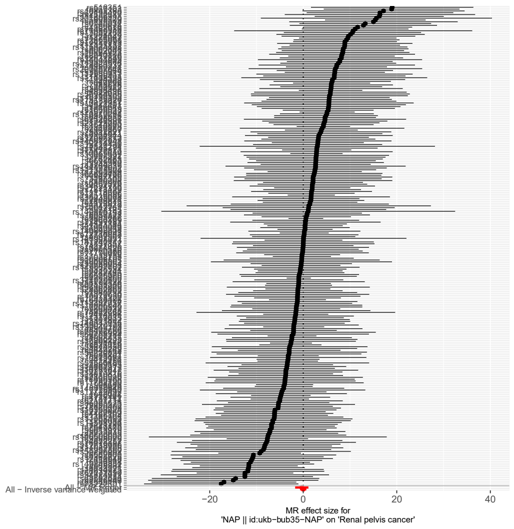
All − Inverse variance weighted
−20
0
20
40
MR effect size for
'NAP || id:ukb−bub35−NAP' on 'Renal pelvis cancer'

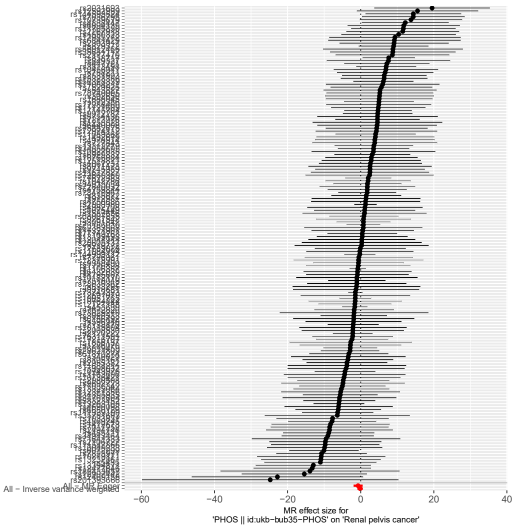
All − Inverse variance weighted
−60
−40
−20
0
20
40
MR effect size for
'PHOS || id:ukb−bub35−PHOS' on 'Renal pelvis cancer'

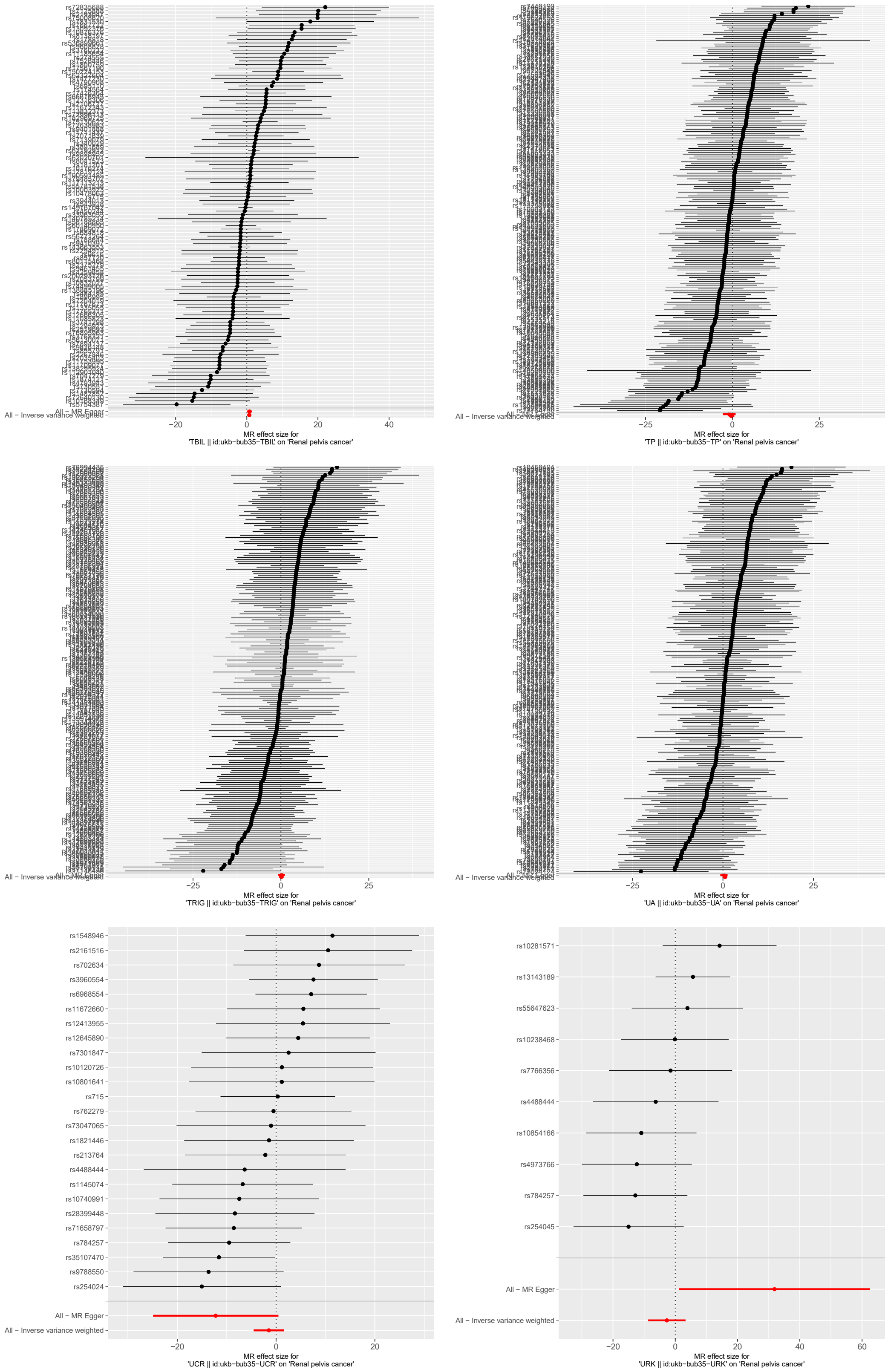
rs72835688
rs5754367
All − MR Egger
All − Inverse variance weighted
−20
0
20
40
MR effect size for
'TBIL || id:ukb−bub35−TBIL' on 'Renal pelvis cancer'
All − Inverse variance weighted
−25
0
25
MR effect size for
'TP || id:ukb−bub35−TP' on 'Renal pelvis cancer'
All − Inverse variance weighted
−25
0
25
MR effect size for
'TRIG || id:ukb−bub35−TRIG' on 'Renal pelvis cancer'
All − Inverse variance weighted
−25
0
25
MR effect size for
'UA || id:ukb−bub35−UA' on 'Renal pelvis cancer'
rs1548946
rs2161516
rs702634
rs3960554
rs6968554
rs11672660
rs12413955
rs12645890
rs7301847
rs10120726
rs10801641
rs715
rs762279
rs73047065
rs1821446
rs213764
rs4488444
rs1145074
rs10740991
rs28399448
rs71658797
rs784257
rs35107470
rs9788550
rs254024
All − MR Egger
All − Inverse variance weighted
−20
0
20
MR effect size for
'UCR || id:ukb−bub35−UCR' on 'Renal pelvis cancer'
rs10281571
rs13143189
rs55647623
rs10238468
rs7766356
rs4488444
rs10854166
rs4973766
rs784257
rs254045
All − MR Egger
All − Inverse variance weighted
−20
0
20
40
60
MR effect size for
'URK || id:ukb−bub35−URK' on 'Renal pelvis cancer'

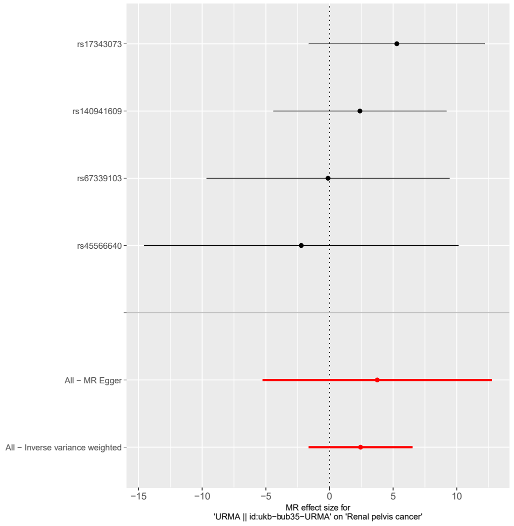
rs17343073
rs140941609
rs67339103
rs45566640
All − MR Egger
All − Inverse variance weighted
−15
−10
−5
0
5
10
MR effect size for
'URMA || id:ukb−bub35−URMA' on 'Renal pelvis cancer'

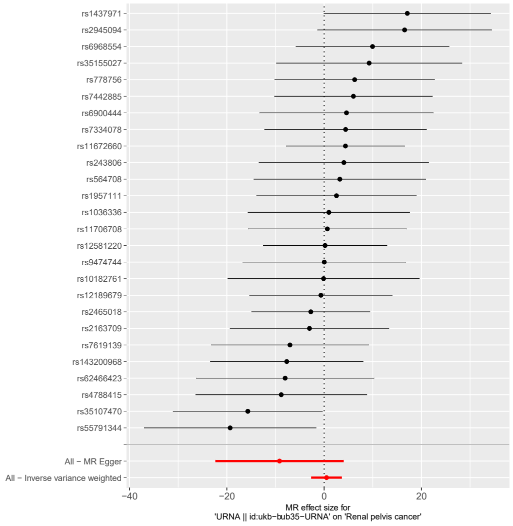
rs1437971
rs2945094
rs6968554
rs35155027
rs778756
rs7442885
rs6900444
rs7334078
rs11672660
rs243806
rs564708
rs1957111
rs1036336
rs11706708
rs12581220
rs9474744
rs10182761
rs12189679
rs2465018
rs2163709
rs7619139
rs143200968
rs62466423
rs4788415
rs35107470
rs55791344
All − MR Egger
All − Inverse variance weighted
−40
−20
0
20
MR effect size for
'URNA || id:ukb−bub35−URNA' on 'Renal pelvis cancer'

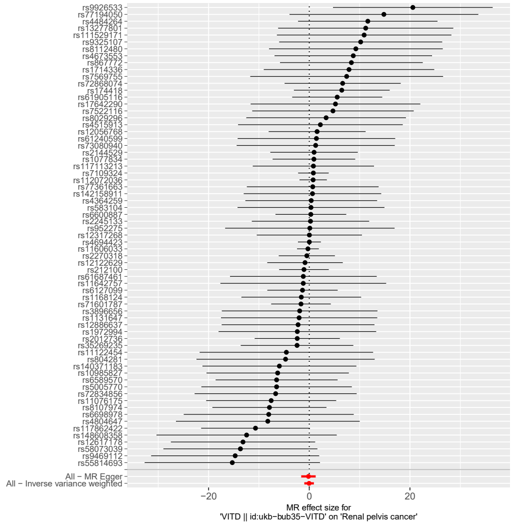
rs9926533
rs77194050
rs4484264
rs13277801
rs111529171
rs9325107
rs8112480
rs4673553
rs867772
rs1714336
rs7569755
rs72868074
rs174418
rs61905116
rs17642290
rs7522116
rs8029296
rs4515913
rs12056768
rs61240599
rs73080940
rs2144529
rs1077834
rs117113213
rs7109324
rs112072036
rs77361663
rs142158911
rs4364259
rs583104
rs6600887
rs2245133
rs952275
rs12317268
rs4694423
rs11606033
rs2270318
rs12122629
rs212100
rs61687461
rs11642757
rs6127099
rs1168124
rs71601787
rs3896656
rs1131647
rs12886637
rs1972994
rs2012736
rs35269235
rs11122454
rs804281
rs140371183
rs10985827
rs6589570
rs5005770
rs72834856
rs11076175
rs8107974
rs6698978
rs4804647
rs117862422
rs148608358
rs12617178
rs58073039
rs9469112
rs55814693
All − MR Egger
All − Inverse variance weighted
−20
0
20
MR effect size for
'VITD || id:ukb−bub35−VITD' on 'Renal pelvis cancer'

Forest plot of KCa

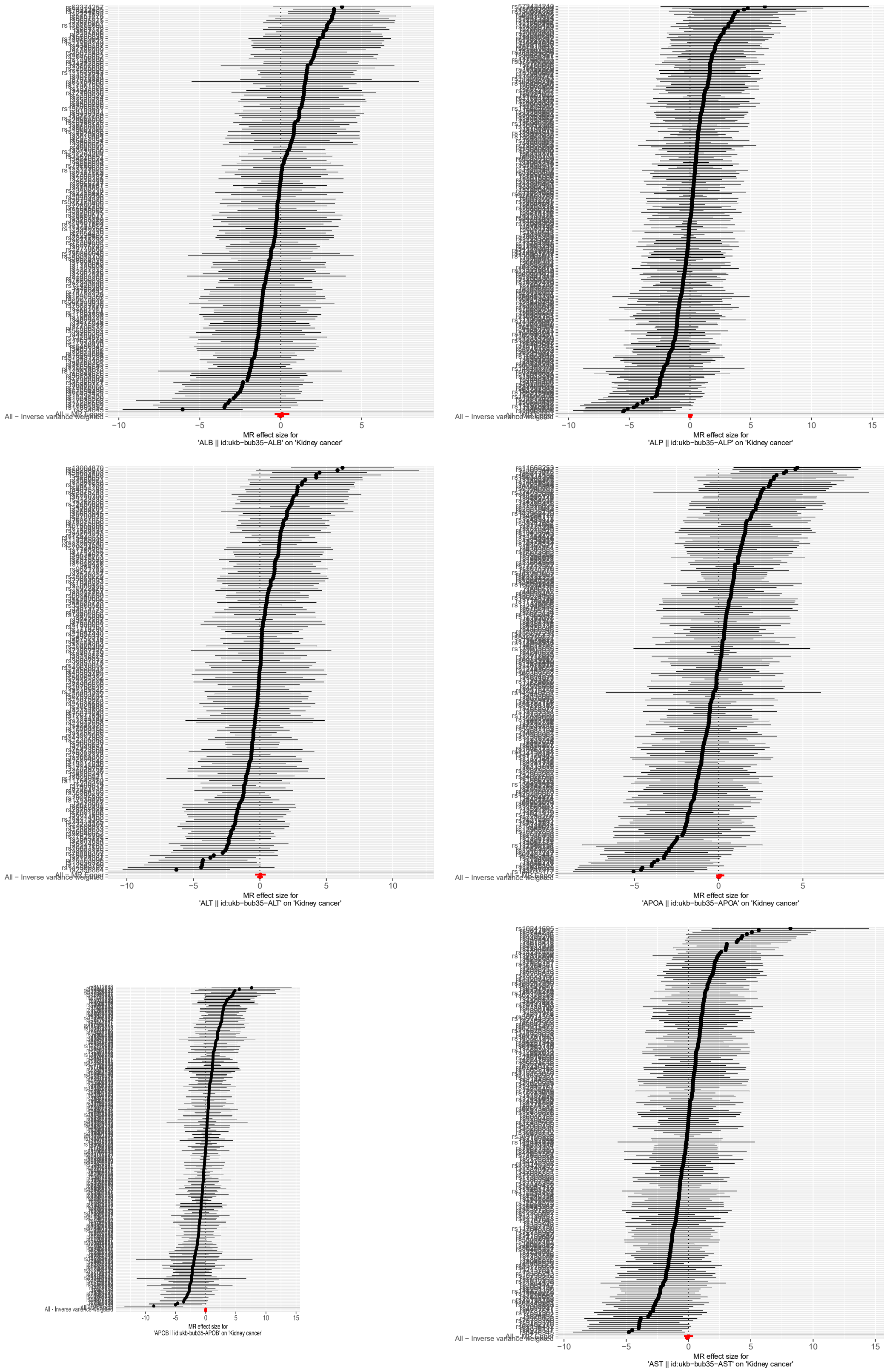

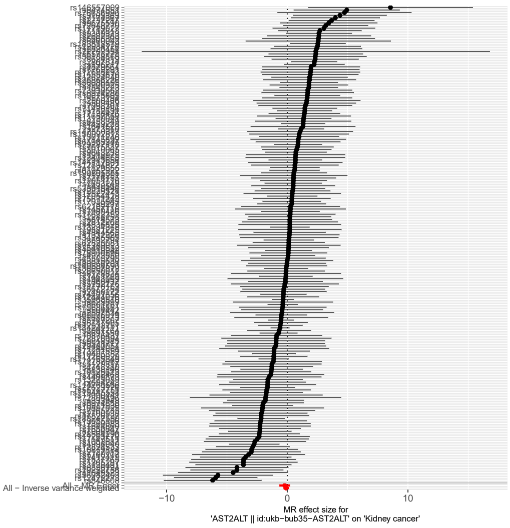
All − Inverse variance weighted
−10
0
10
MR effect size for
'AST2ALT || id:ukb−bub35−AST2ALT' on 'Kidney cancer'

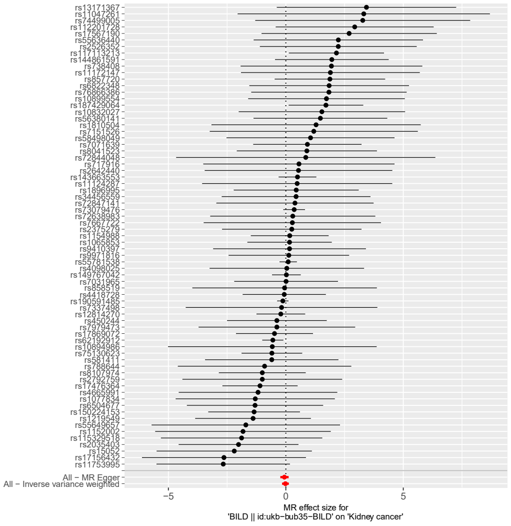
All − MR Egger
All − Inverse variance weighted
−5
0
5
MR effect size for
'BILD || id:ukb−bub35−BILD' on 'Kidney cancer'

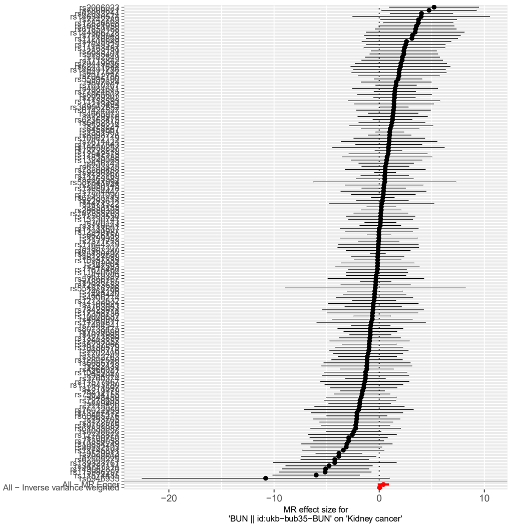
All − Inverse variance weighted
−20
−10
0
10
MR effect size for
'BUN || id:ukb−bub35−BUN' on 'Kidney cancer'

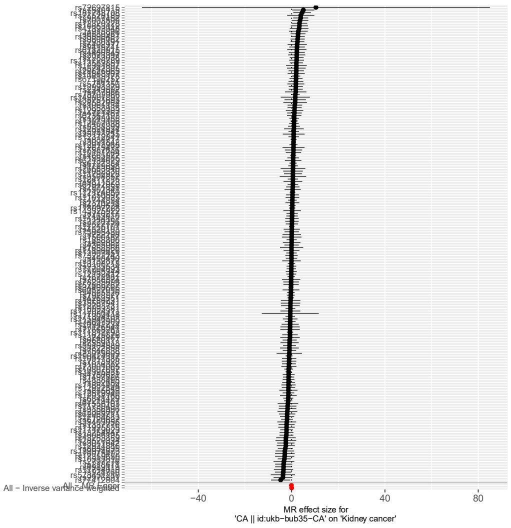
All − Inverse variance weighted
−40
0
40
80
MR effect size for
'CA || id:ukb−bub35−CA' on 'Kidney cancer'

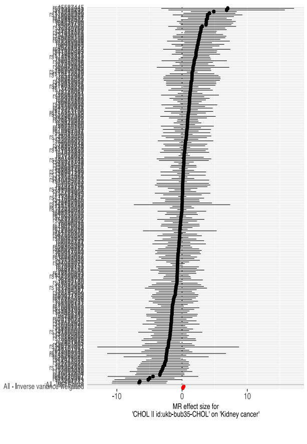
All - Inverse variance weighted
-10
0
10
MR effect size for
'CHOL || id:ukb-bub35-CHOL' on 'Kidney cancer'

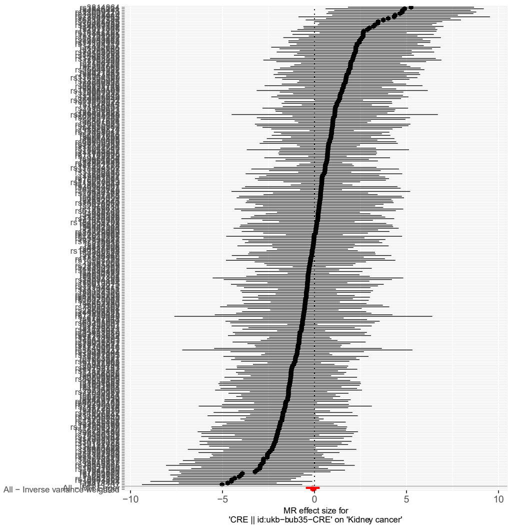
All − Inverse variance weighted
−10
−5
0
5
10
MR effect size for
'CRE || id:ukb−bub35−CRE' on 'Kidney cancer'

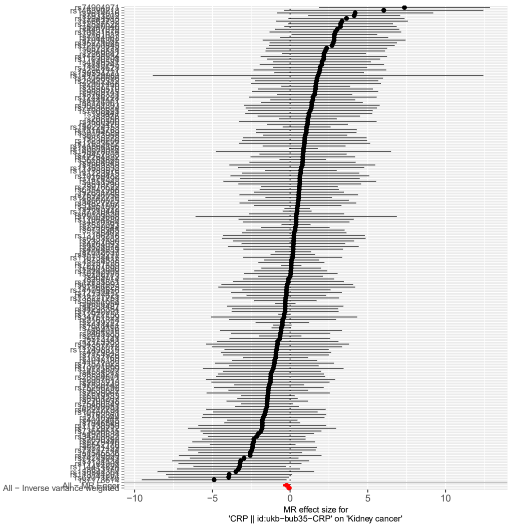
All − Inverse variance weighted
−10
−5
0
5
10
MR effect size for
'CRP || id:ukb−bub35−CRP' on 'Kidney cancer'

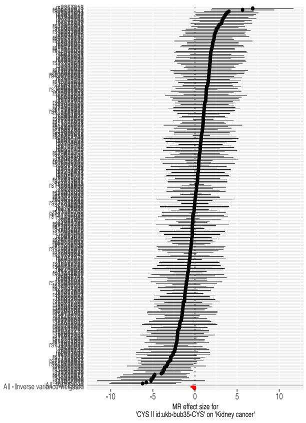
All - Inverse variance weighted
-10
-5
0
5
10
MR effect size for
'CYS || id:ukb-bub35-CYS' on 'Kidney cancer'

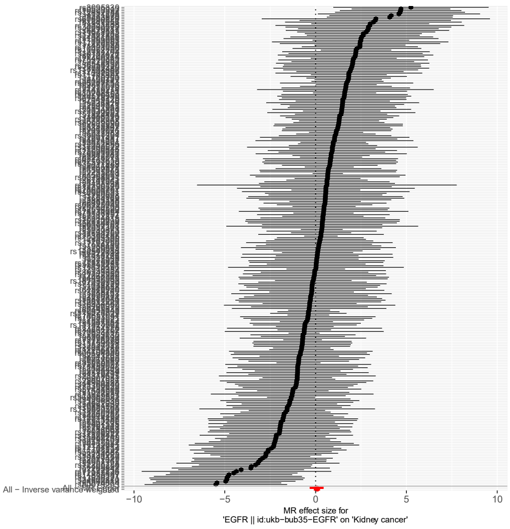
All − Inverse variance weighted
−10
−5
0
5
10
MR effect size for
'EGFR || id:ukb−bub35−EGFR' on 'Kidney cancer'

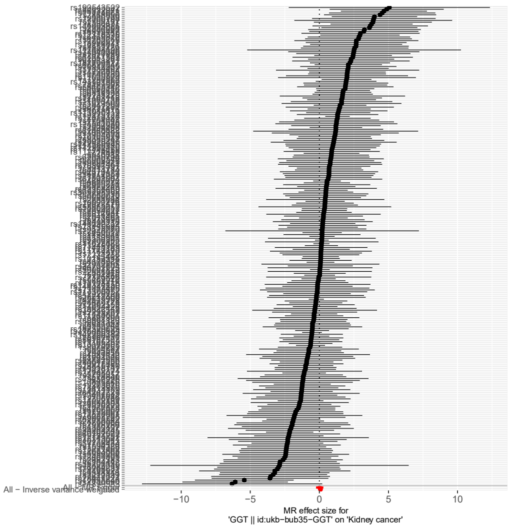
All − Inverse variance weighted
−10
−5
0
5
10
MR effect size for
'GGT || id:ukb−bub35−GGT' on 'Kidney cancer'

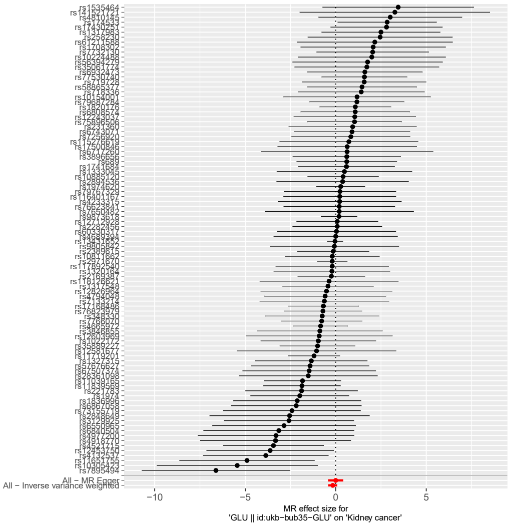
All − MR Egger
All − Inverse variance weighted
−10
−5
0
5
MR effect size for
'GLU || id:ukb−bub35−GLU' on 'Kidney cancer'

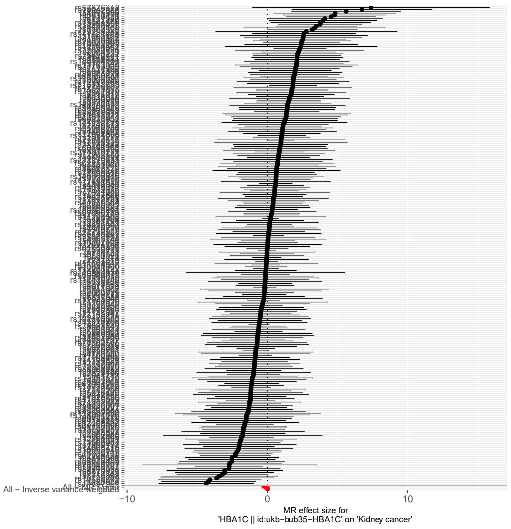
All − Inverse variance weighted
−10
0
10
MR effect size for
'HBA1C || id:ukb−bub35−HBA1C' on 'Kidney cancer'

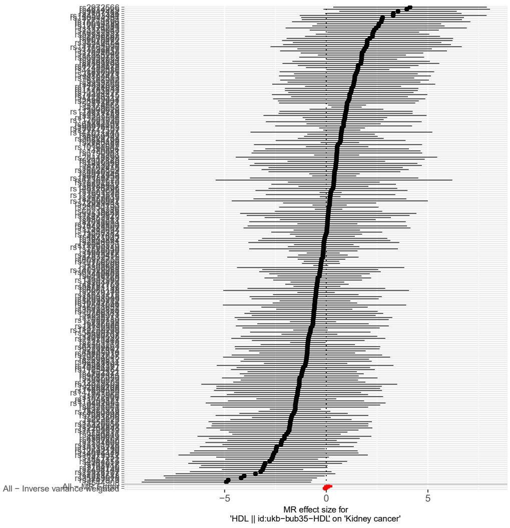
All − Inverse variance weighted
−5
0
5
MR effect size for
'HDL || id:ukb−bub35−HDL' on 'Kidney cancer'

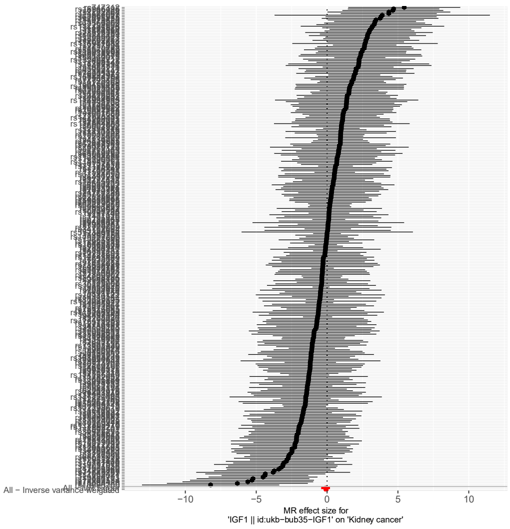
All − Inverse variance weighted
−10
−5
0
5
10
MR effect size for
'IGF1 || id:ukb−bub35−IGF1' on 'Kidney cancer'

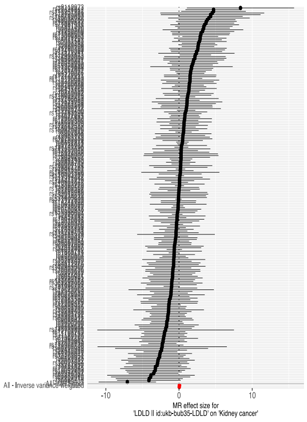
All - Inverse variance weighted
-10
0
10
MR effect size for
'LDLD || id:ukb-bub35-LDLD' on 'Kidney cancer'

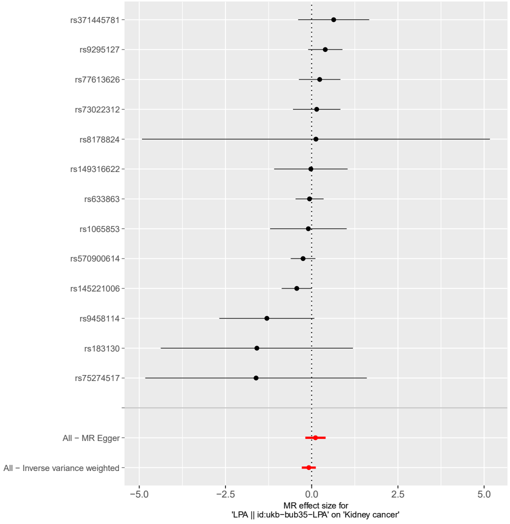
rs371445781
rs9295127
rs77613626
rs73022312
rs8178824
rs149316622
rs633863
rs1065853
rs570900614
rs145221006
rs9458114
rs183130
rs75274517
All − MR Egger
All − Inverse variance weighted
−5.0
−2.5
0.0
2.5
5.0
MR effect size for
'LPA || id:ukb−bub35−LPA' on 'Kidney cancer'

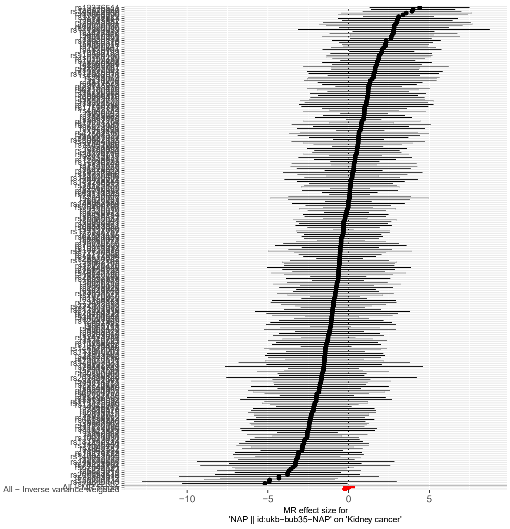
All − Inverse variance weighted
−10
−5
0
5
MR effect size for
'NAP || id:ukb−bub35−NAP' on 'Kidney cancer'

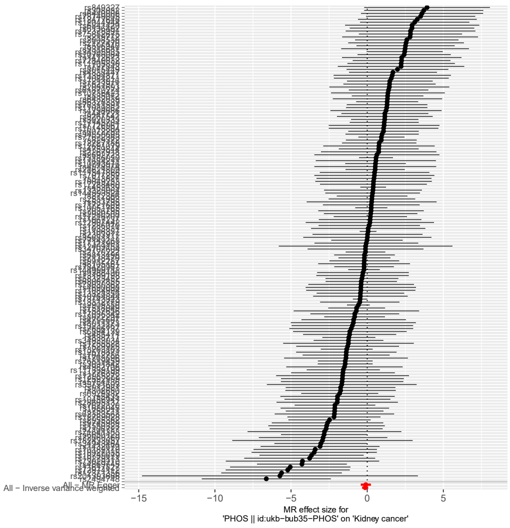
All − Inverse variance weighted
−15
−10
−5
0
5
MR effect size for
'PHOS || id:ukb−bub35−PHOS' on 'Kidney cancer'

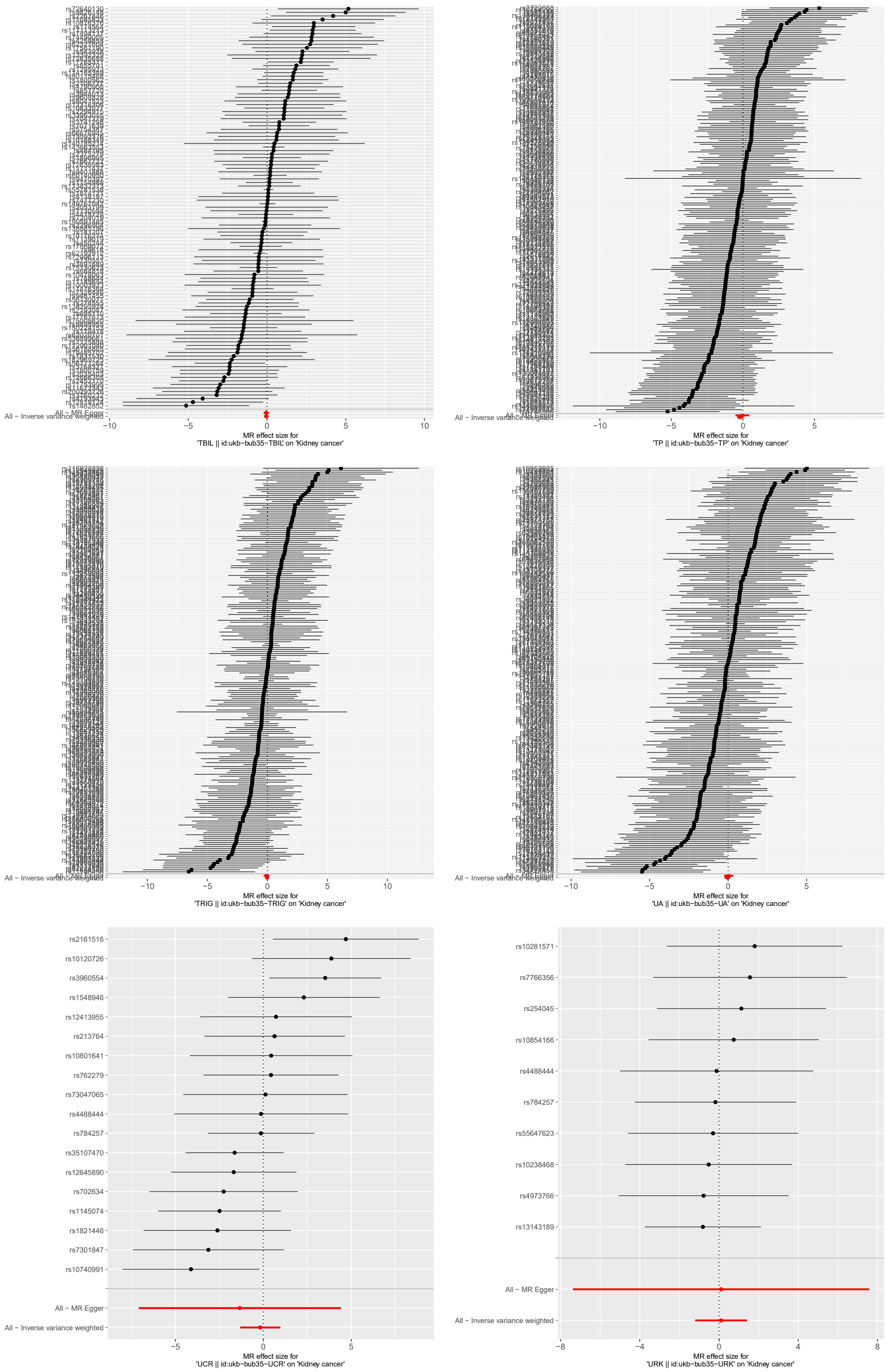
MR effect size for
'TBIL || id:ukb−bub35−TBIL' on 'Kidney cancer'
MR effect size for
'TP || id:ukb−bub35−TP' on 'Kidney cancer'
MR effect size for
'TRIG || id:ukb−bub35−TRIG' on 'Kidney cancer'
MR effect size for
'UA || id:ukb−bub35−UA' on 'Kidney cancer'
rs2161516
rs10120726
rs3960554
rs1548946
rs12413955
rs213764
rs10801641
rs762279
rs73047065
rs4488444
rs784257
rs35107470
rs12645890
rs702634
rs1145074
rs1821446
rs7301847
rs10740991
All − MR Egger
All − Inverse variance weighted
MR effect size for
'UCR || id:ukb−bub35−UCR' on 'Kidney cancer'
rs10281571
rs7766356
rs254045
rs10854166
rs4488444
rs784257
rs55647623
rs10238468
rs4973766
rs13143189
All − MR Egger
All − Inverse variance weighted
MR effect size for
'URK || id:ukb−bub35−URK' on 'Kidney cancer'

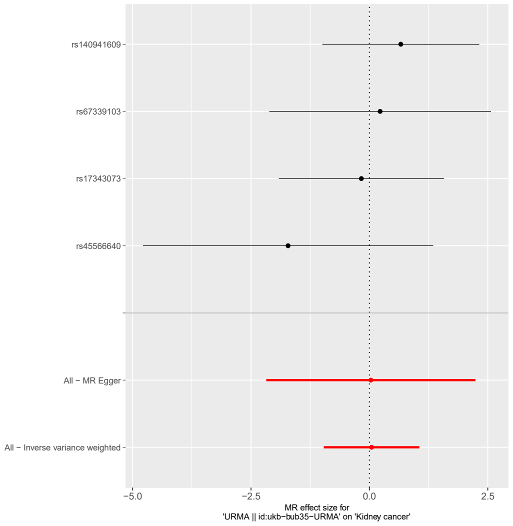
rs140941609
rs67339103
rs17343073
rs45566640
All − MR Egger
All − Inverse variance weighted
−5.0
−2.5
0.0
2.5
MR effect size for
'URMA || id:ukb−bub35−URMA' on 'Kidney cancer'

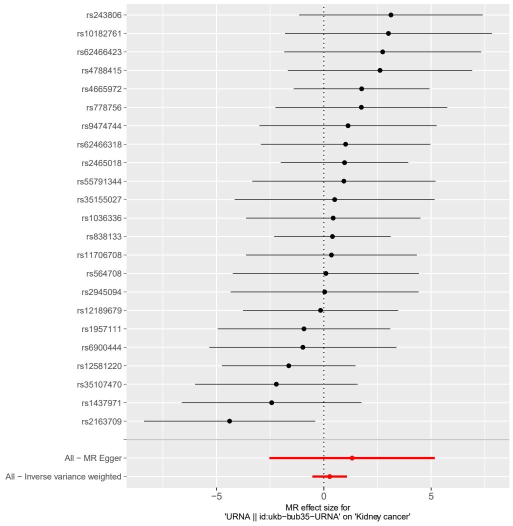
rs243806
rs10182761
rs62466423
rs4788415
rs4665972
rs778756
rs9474744
rs62466318
rs2465018
rs55791344
rs35155027
rs1036336
rs838133
rs11706708
rs564708
rs2945094
rs12189679
rs1957111
rs6900444
rs12581220
rs35107470
rs1437971
rs2163709
All − MR Egger
All − Inverse variance weighted
−5
0
5
MR effect size for
'URNA || id:ukb−bub35−URNA' on 'Kidney cancer'

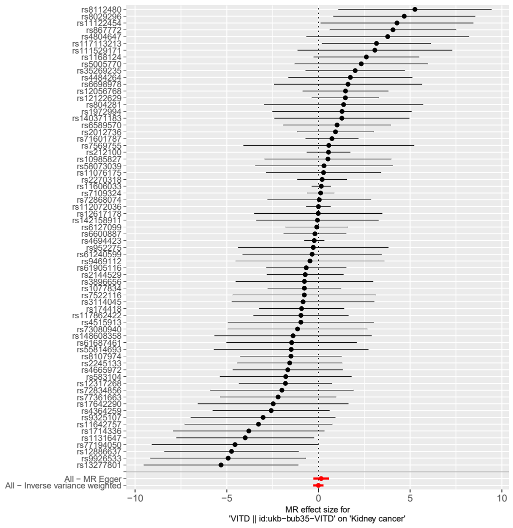
rs8112480
rs8029296
rs11122454
rs867772
rs4804647
rs117113213
rs111529171
rs1168124
rs5005770
rs35269235
rs4484264
rs6698978
rs12056768
rs12122629
rs804281
rs1972994
rs140371183
rs6589570
rs2012736
rs71601787
rs7569755
rs212100
rs10985827
rs58073039
rs11076175
rs2270318
rs11606033
rs7109324
rs72868074
rs112072036
rs12617178
rs142158911
rs6127099
rs6600887
rs4694423
rs952275
rs61240599
rs9469112
rs61905116
rs2144529
rs3896656
rs1077834
rs7522116
rs3114045
rs174418
rs117862422
rs4515913
rs73080940
rs148608358
rs61687461
rs55814693
rs8107974
rs2245133
rs4665972
rs583104
rs12317268
rs72834856
rs77361663
rs17642290
rs4364259
rs9325107
rs11642757
rs1714336
rs1131647
rs77194050
rs12886637
rs9926533
rs13277801
All − MR Egger
All − Inverse variance weighted
−10
−5
0
5
10
MR effect size for
'VITD || id:ukb−bub35−VITD' on 'Kidney cancer'

funnel plot of BCa

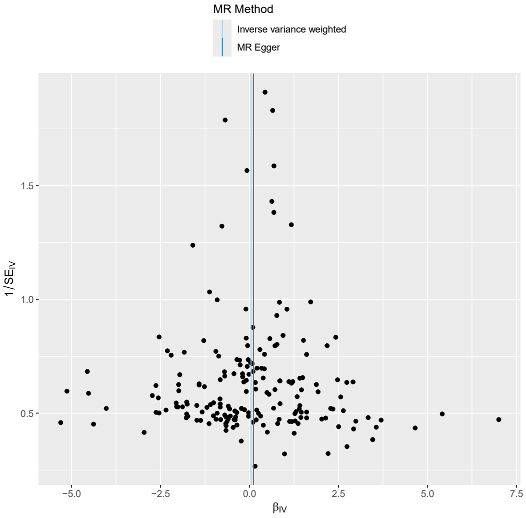

ALB

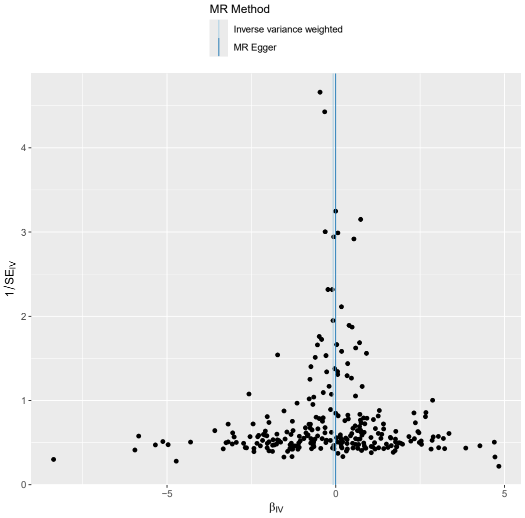

ALP

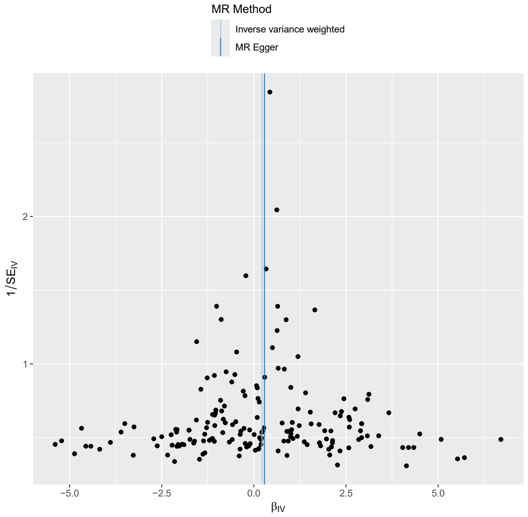

ALT

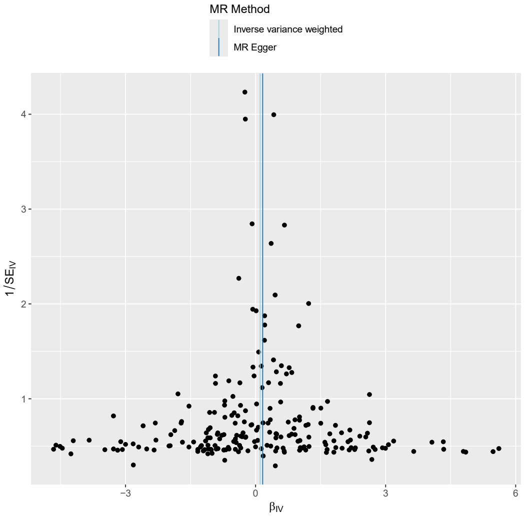

APOA

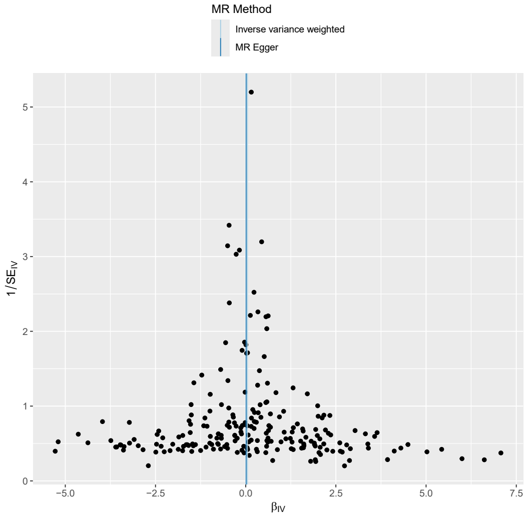

APOB

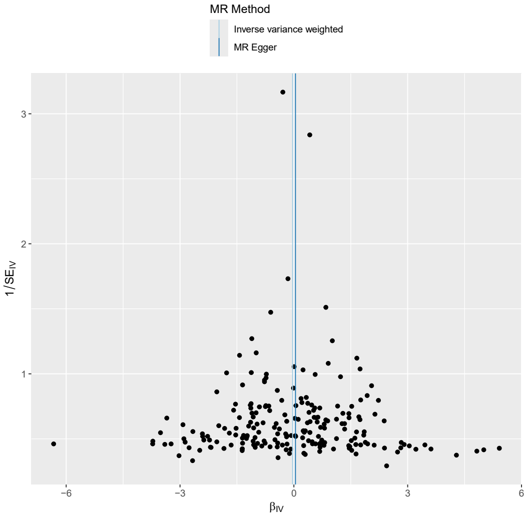

AST

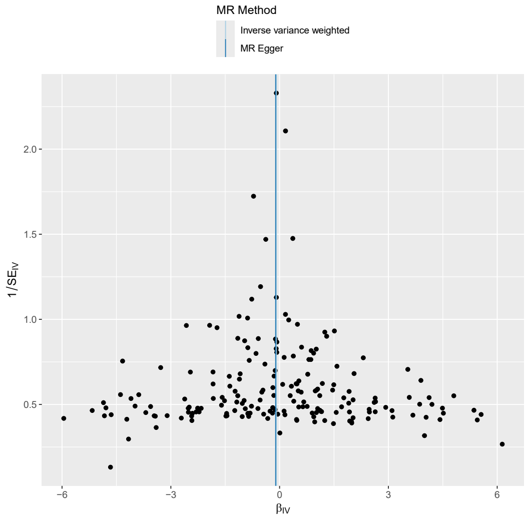

AST/ALT

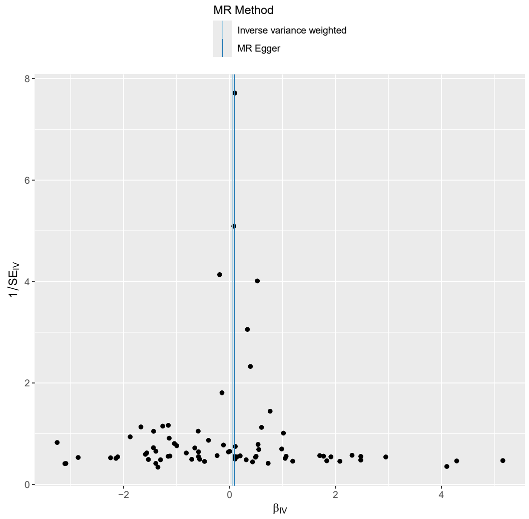

BILD

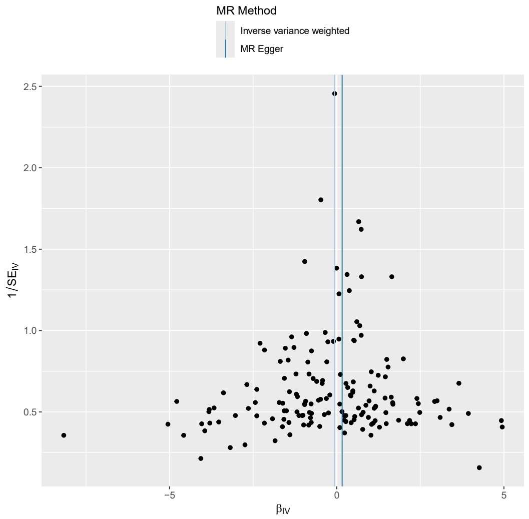

BUN

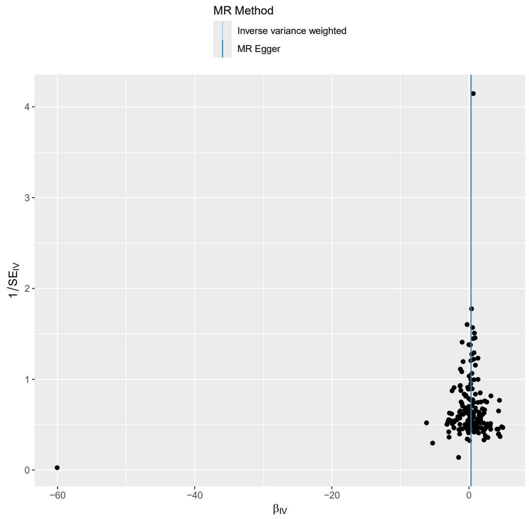

CA

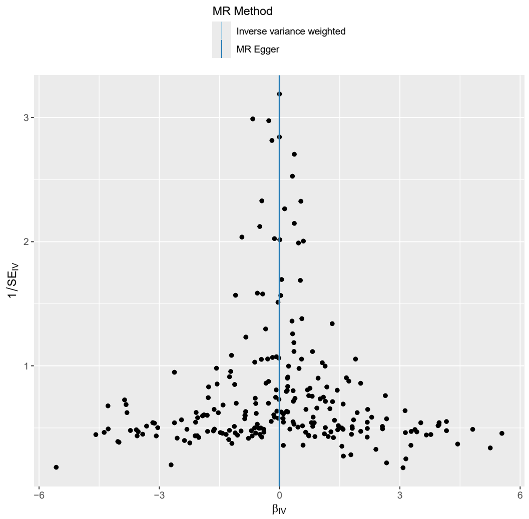

CHOL

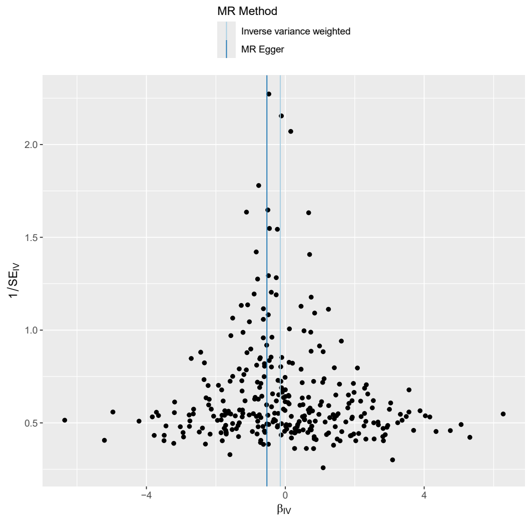

CRE

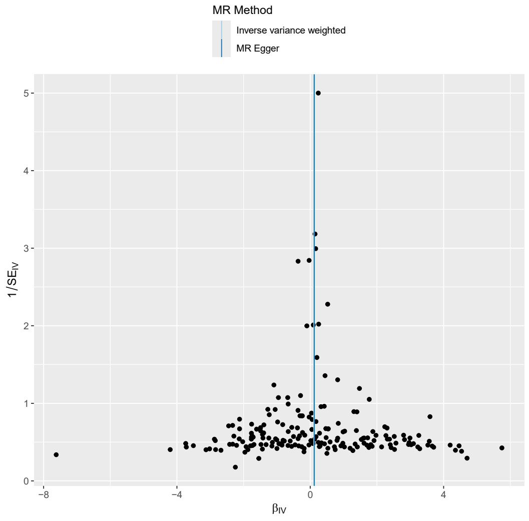

CRP

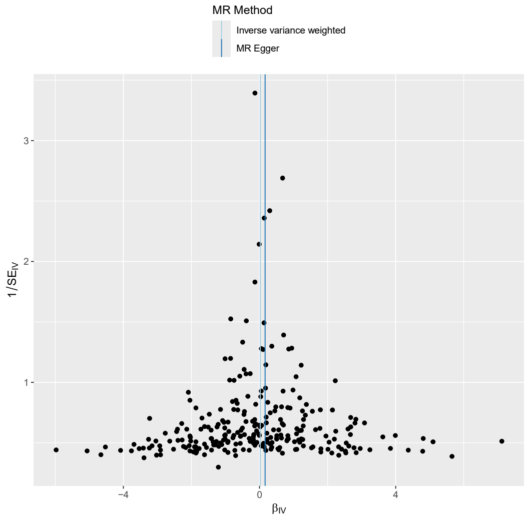

CYS

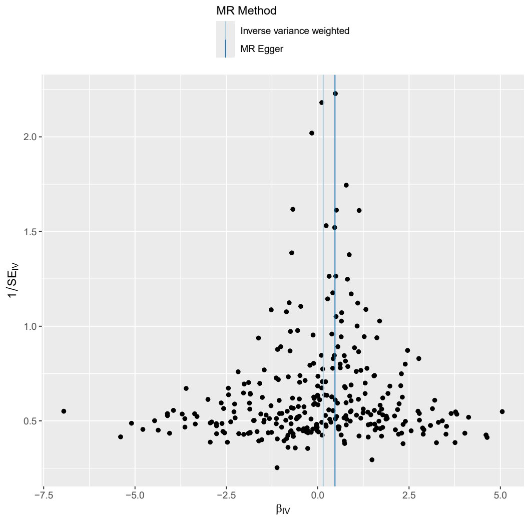

EGFR

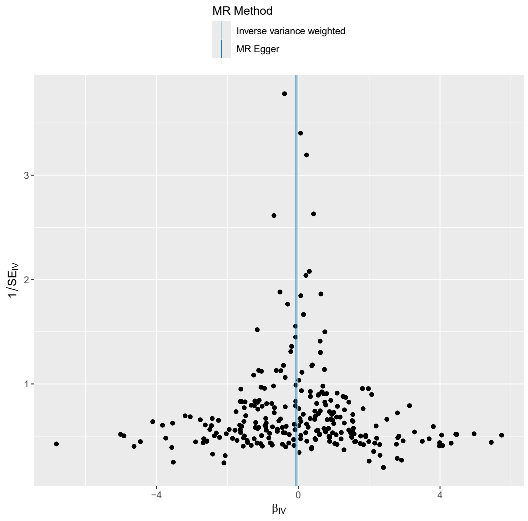

GGT

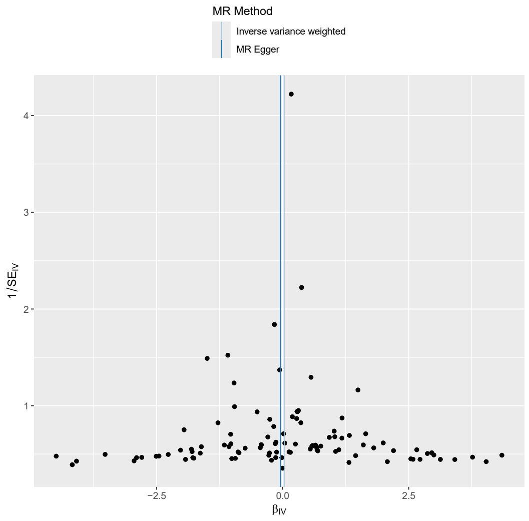

GLU

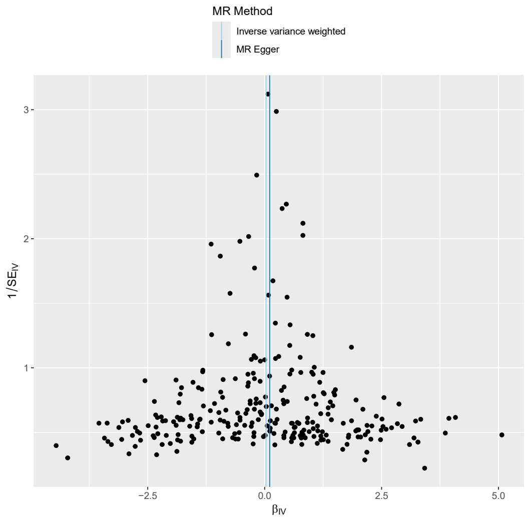

HBA1C

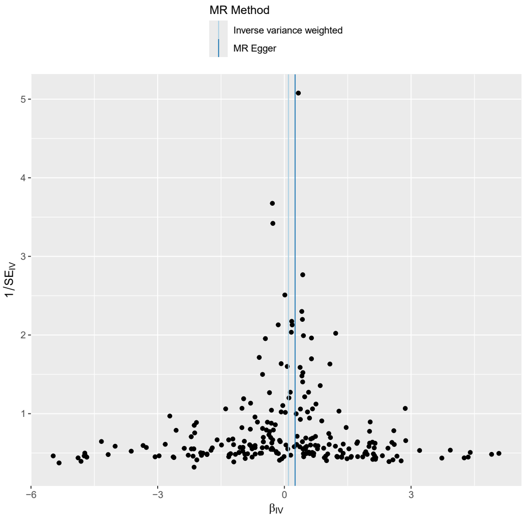

HDL

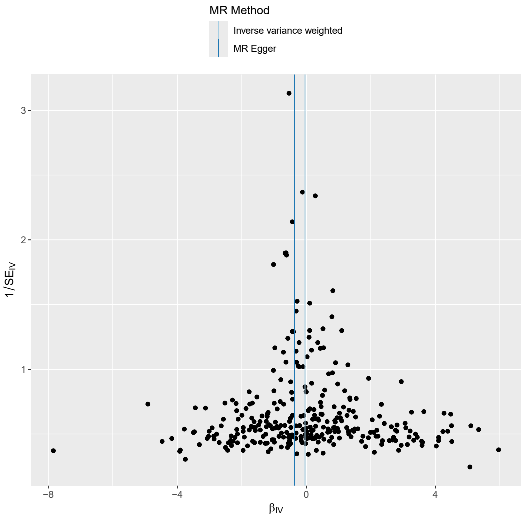

IGF-1

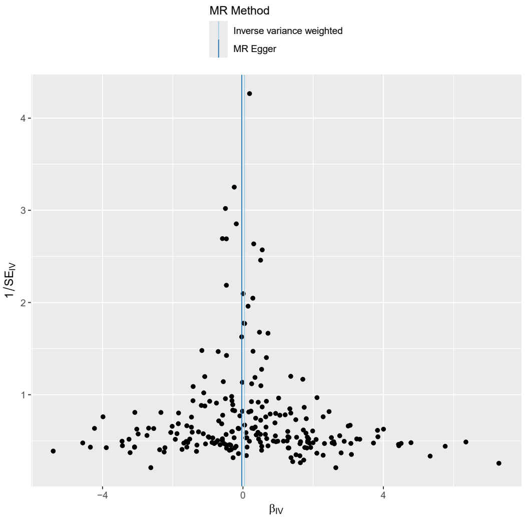

LDLD

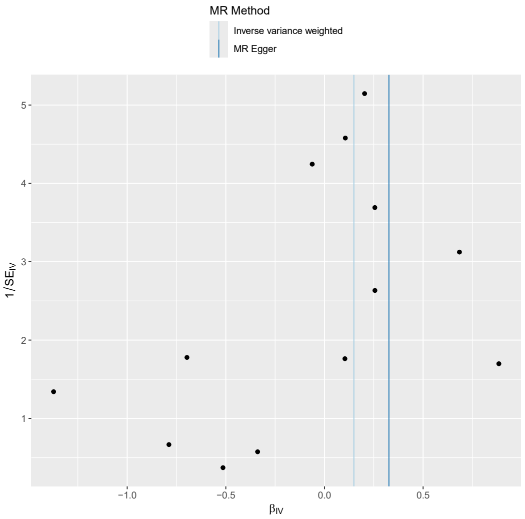

LPA

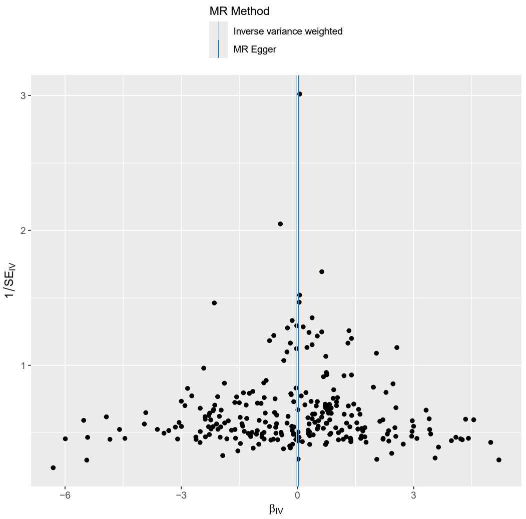

NAP

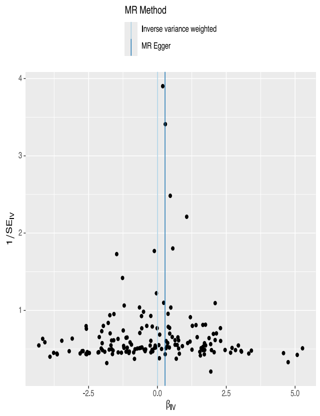

PHOS

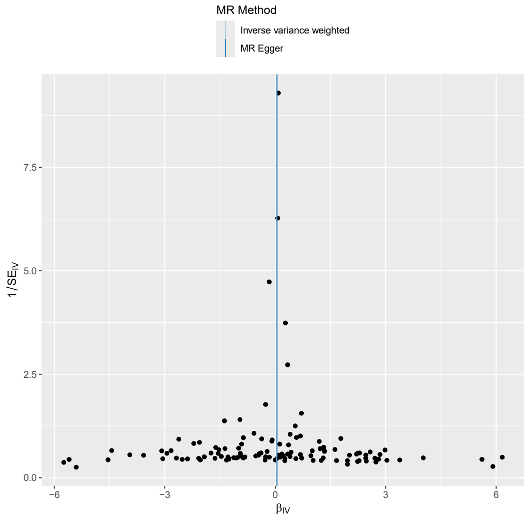

TBIL

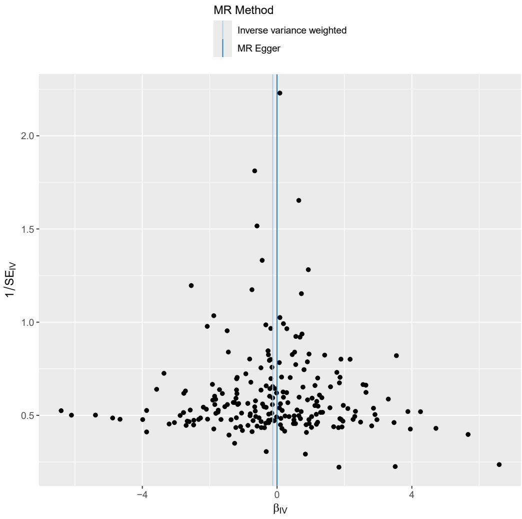

TP

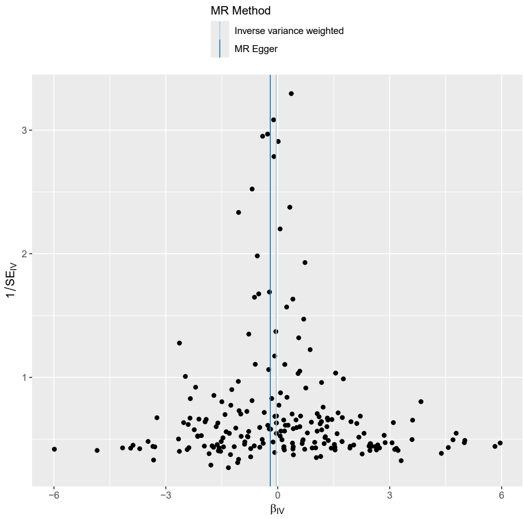

TRIG

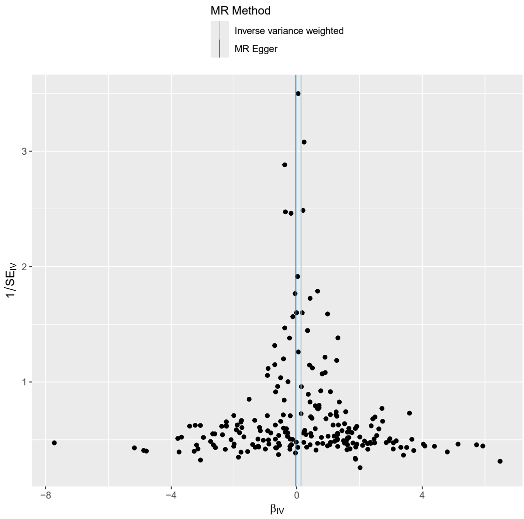

UA

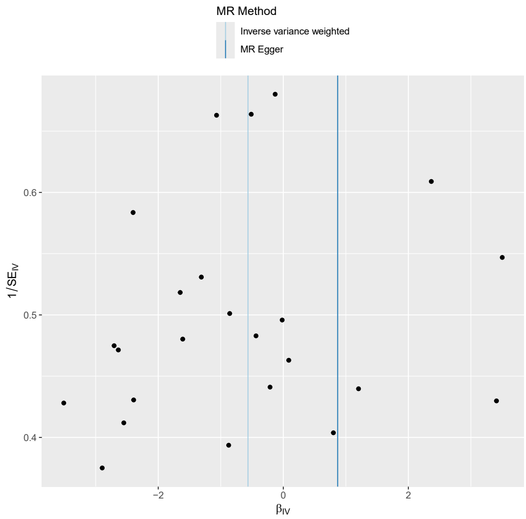

UCR

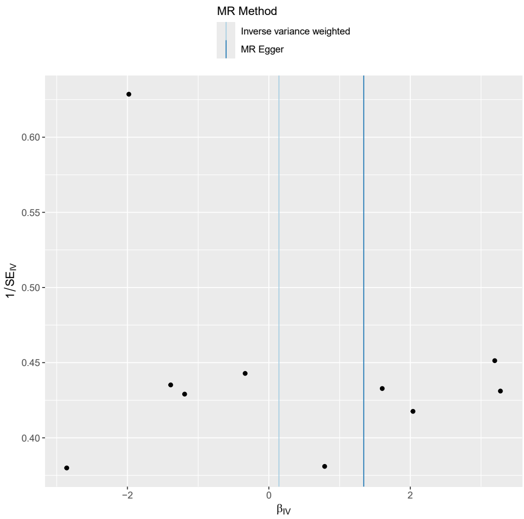

URK

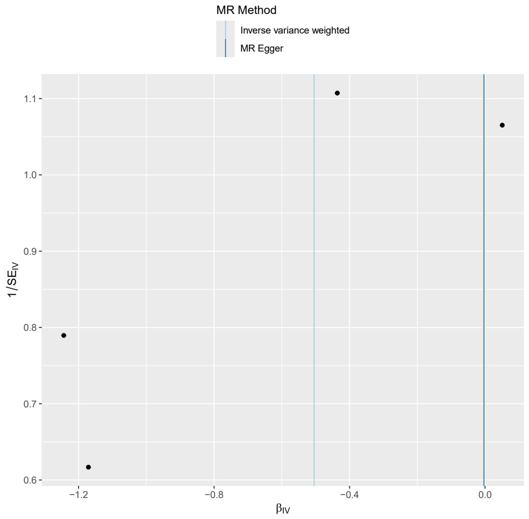

URMA

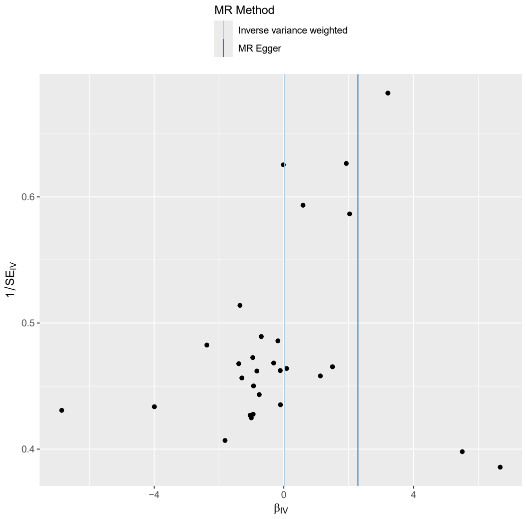

URNA

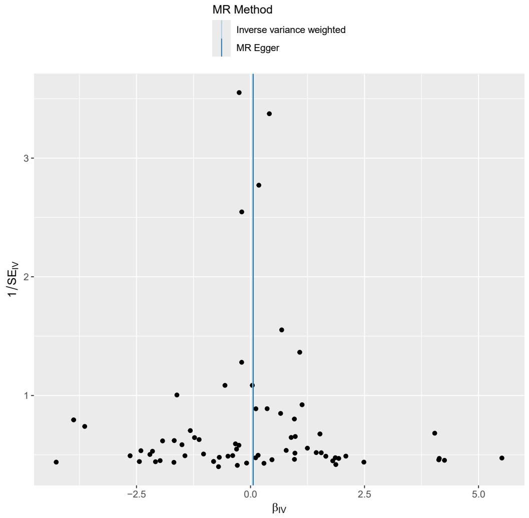

VITD

funnel plot of PCa

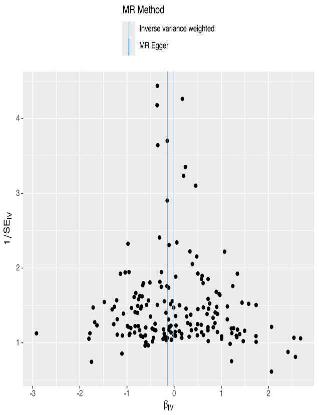

ALB

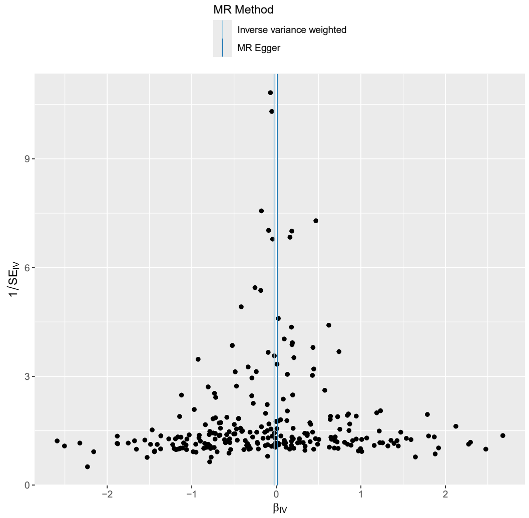

ALP

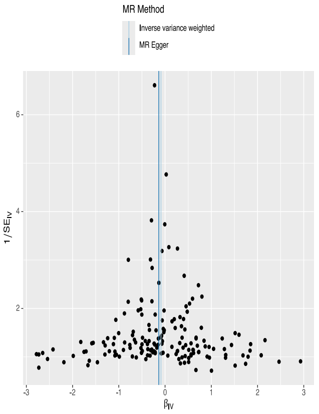

ALT

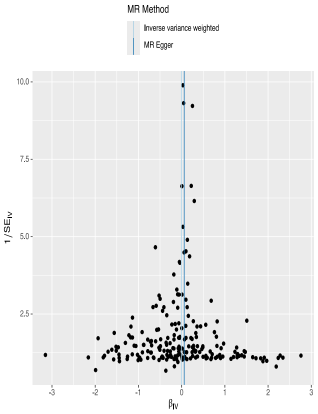

APOA

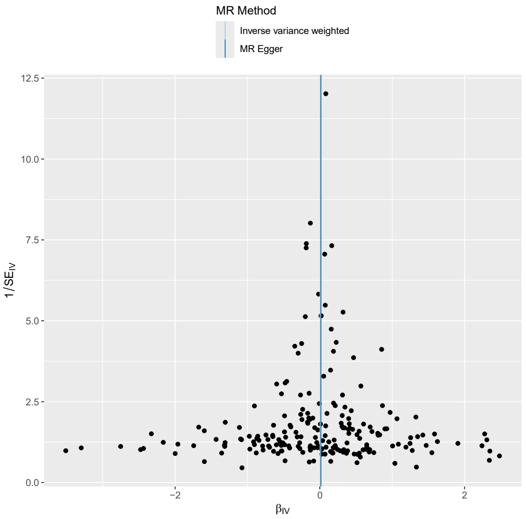

APOB

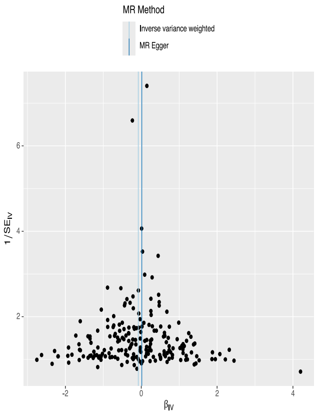

AST

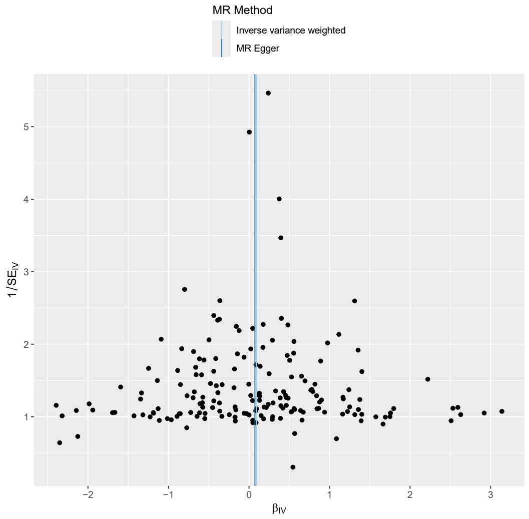

AST/ALT

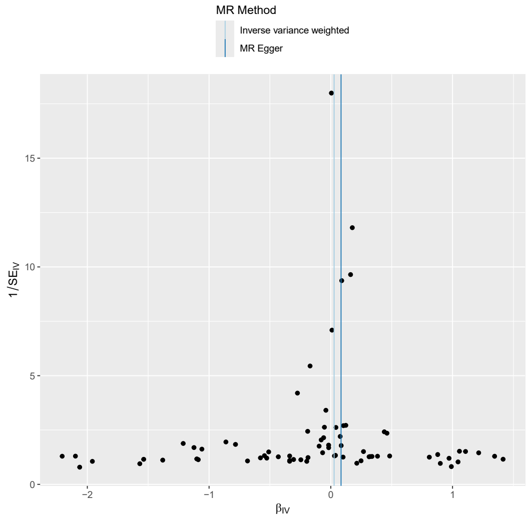

BILD

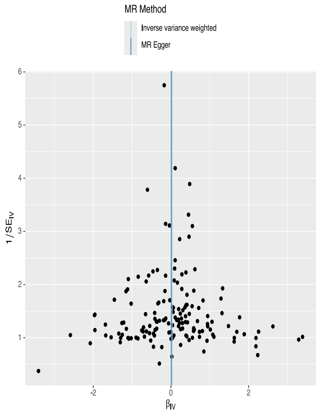

BUN

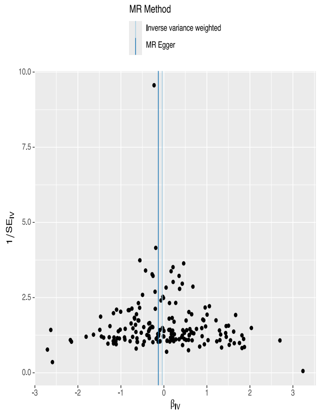

CA

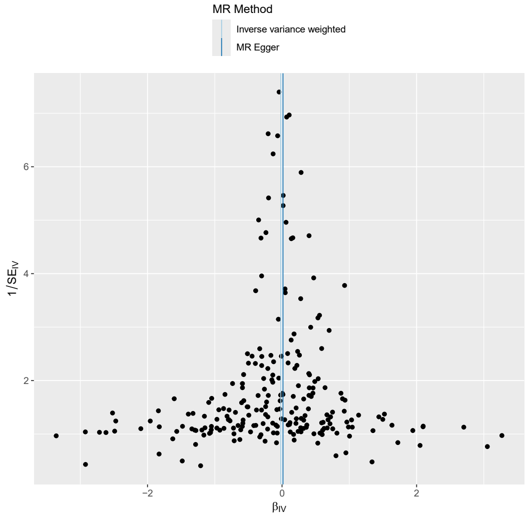

CHOL

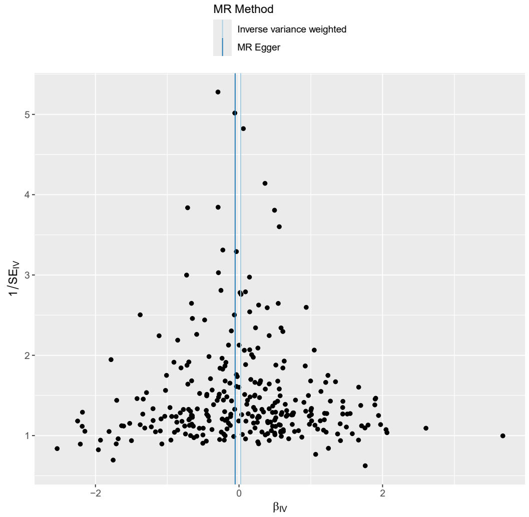

CRE

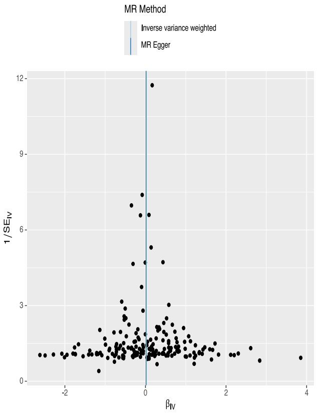

CRP

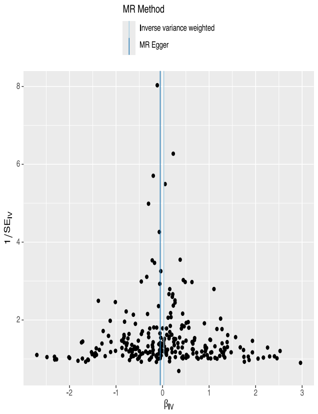

CYS

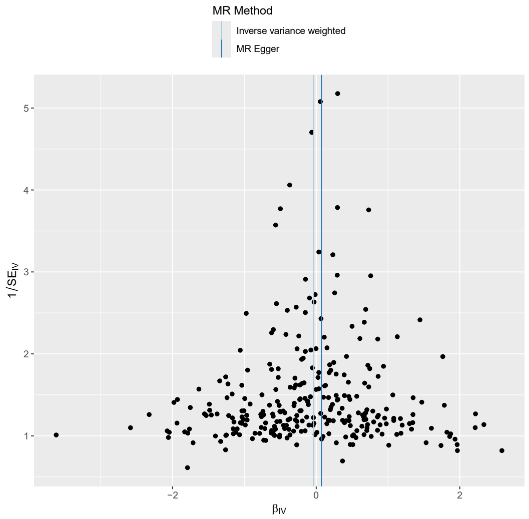

EGFR

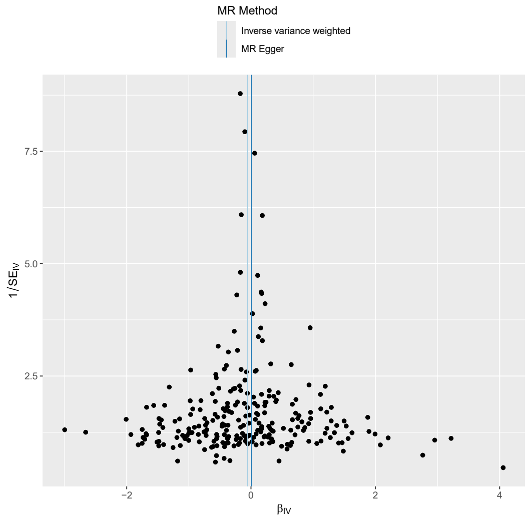

GGT

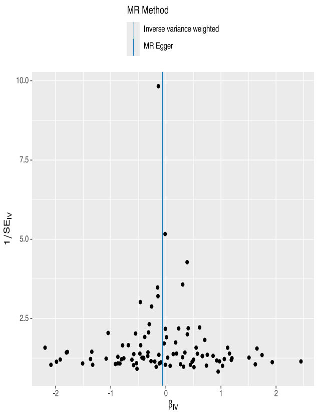

GLU

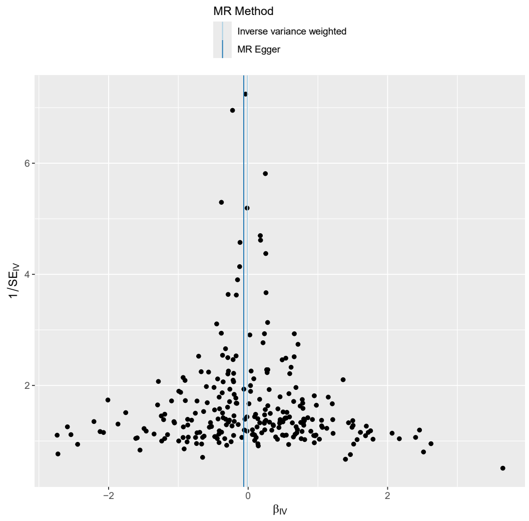

HBA1C

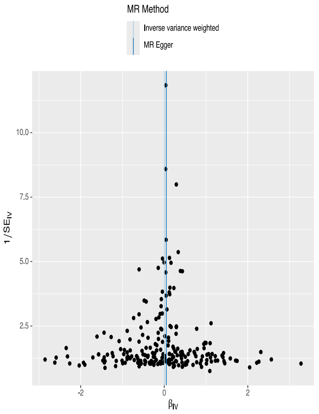

HDL

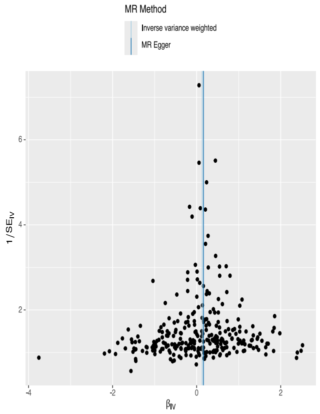

IGF-1

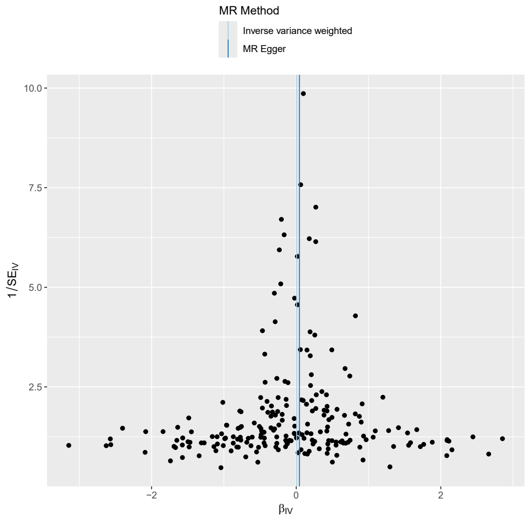

LDLD

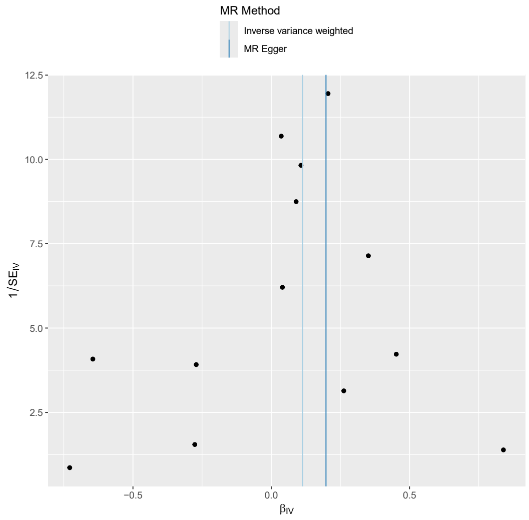

LPA

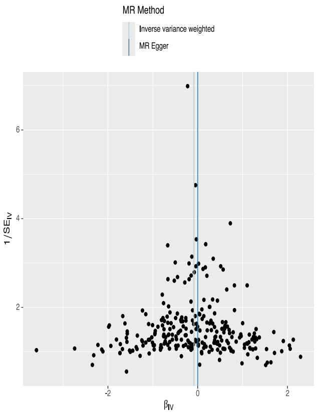

NAP

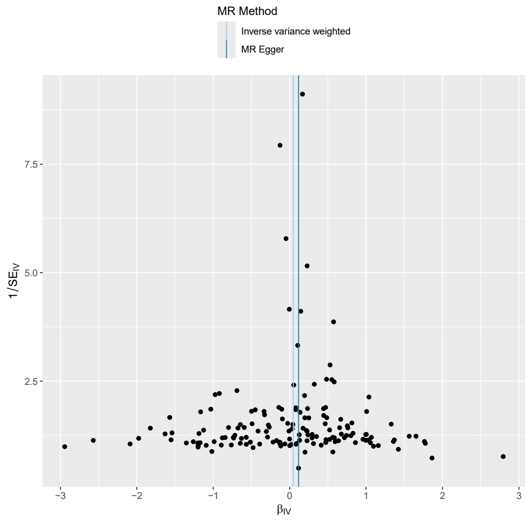

PHOS

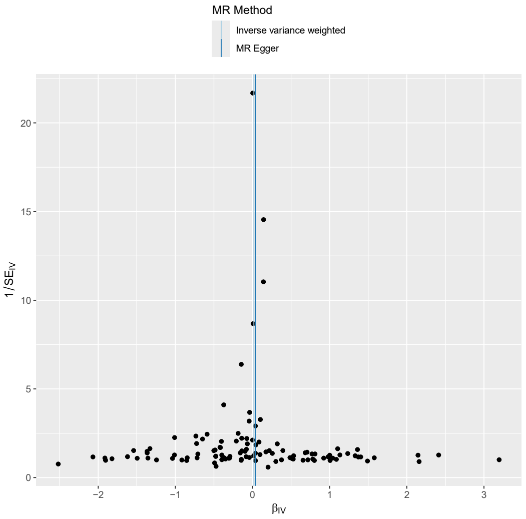

TBIL

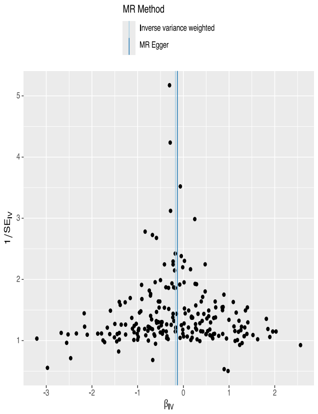

TP

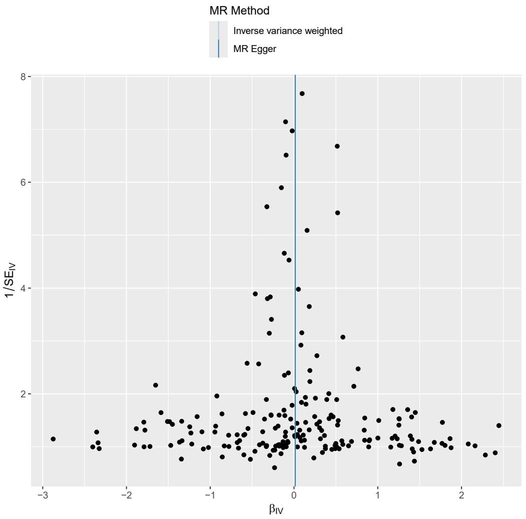

TRIG

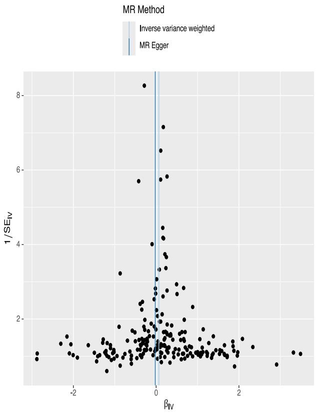

UA

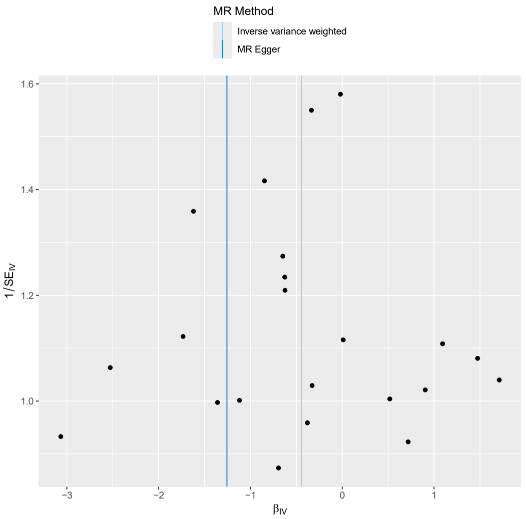

UCR

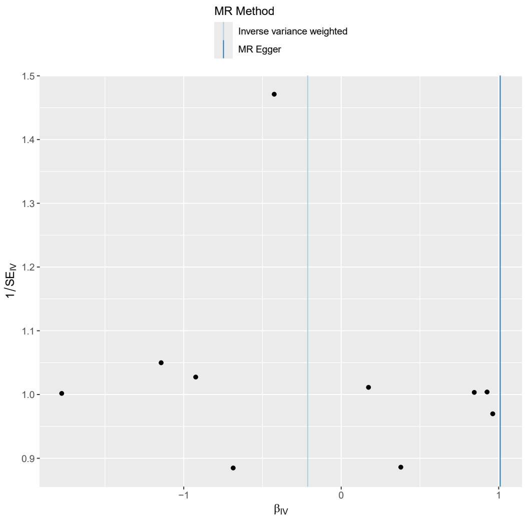

URK

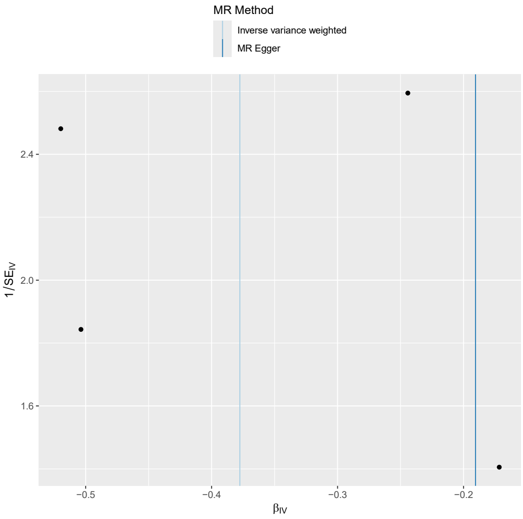

URMA

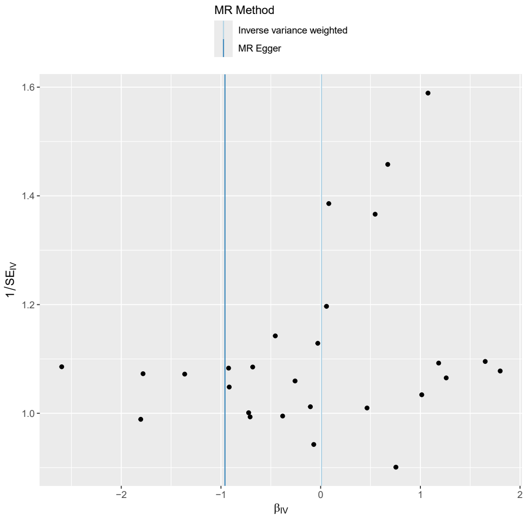

URNA

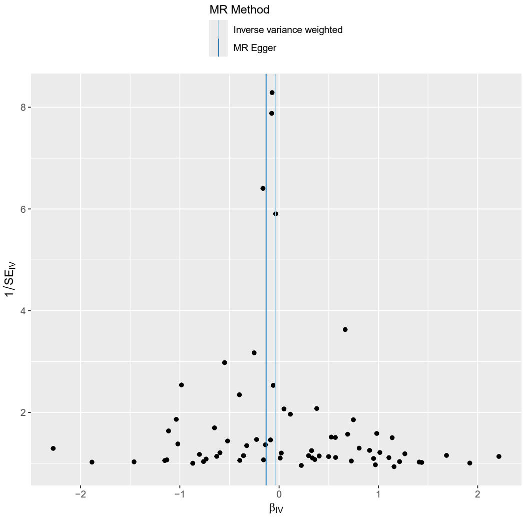

VITD

funnel plot of RPCa

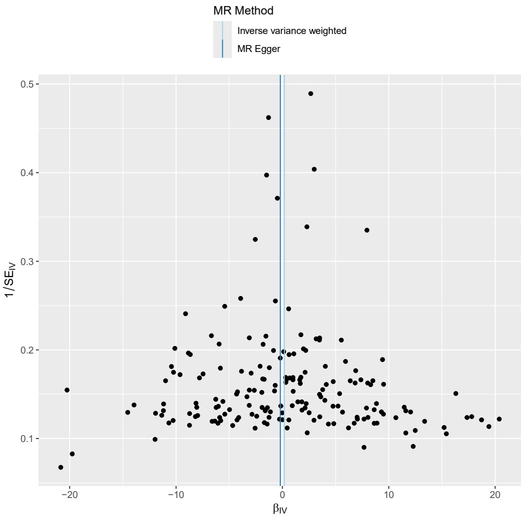

ALB

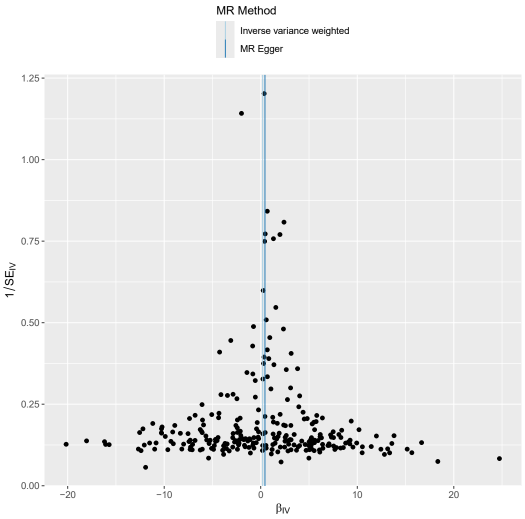

ALP

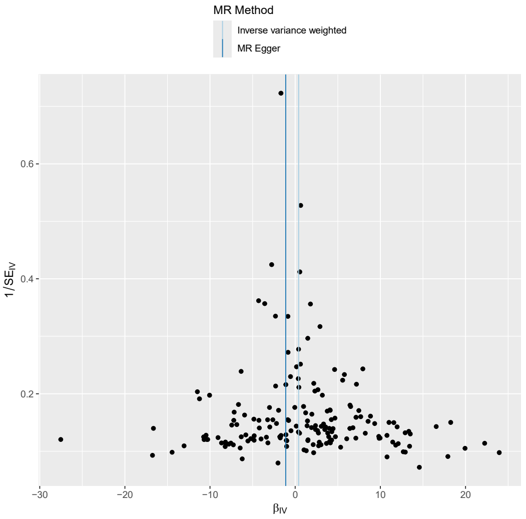

ALT

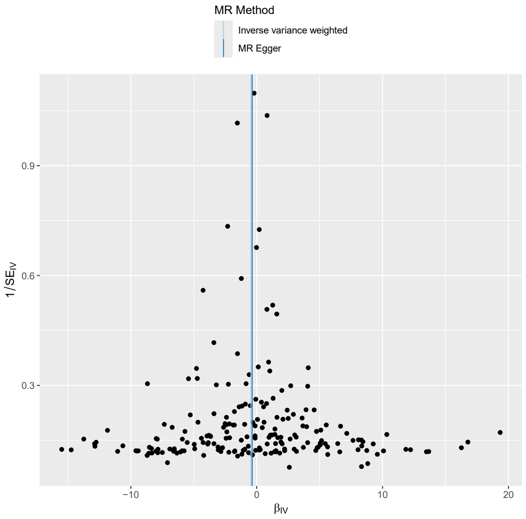

APOA

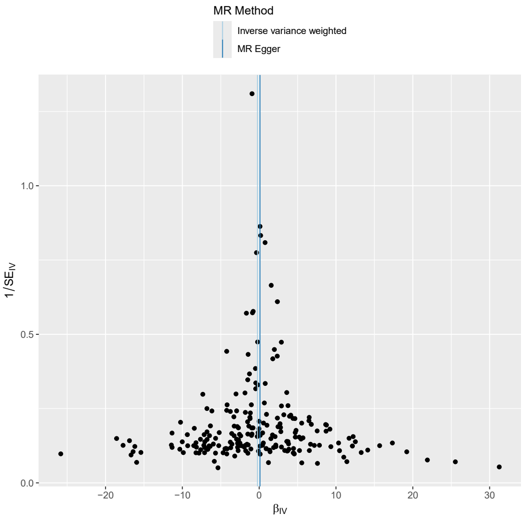

APOB

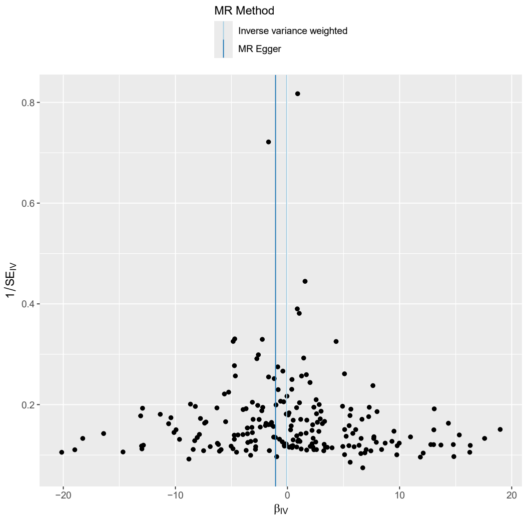

AST

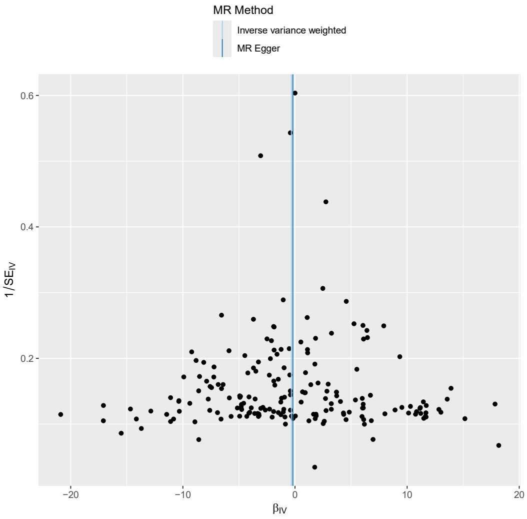

AST/ALT

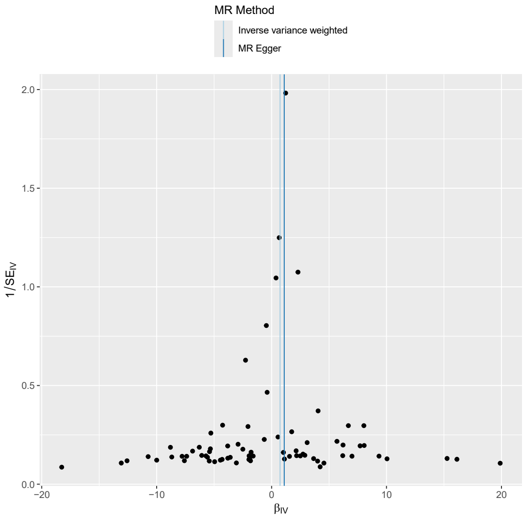

BILD

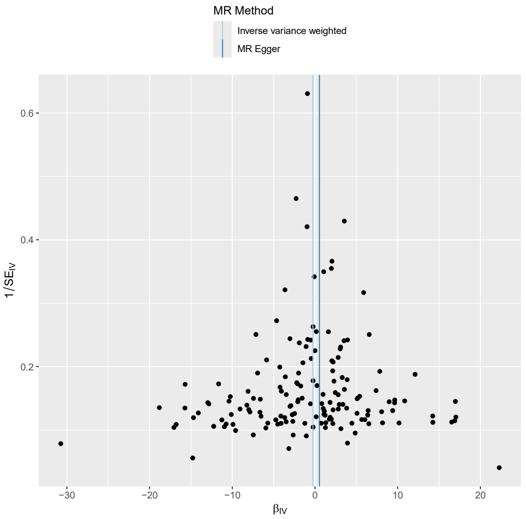

BUN

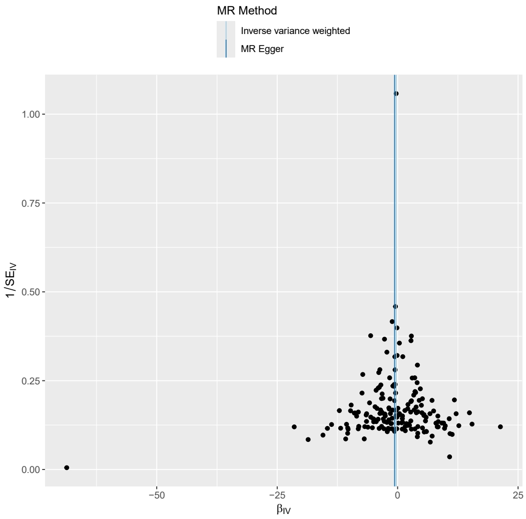

CA

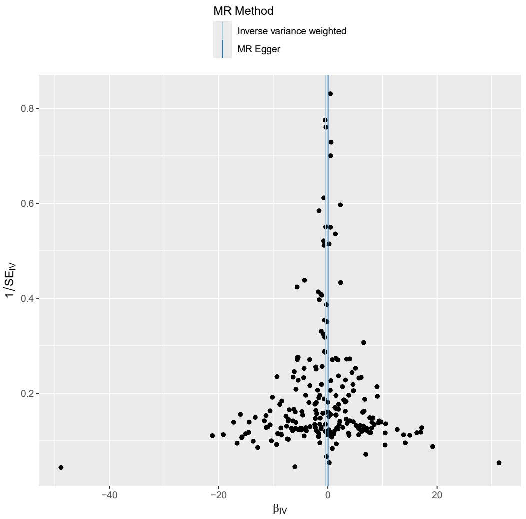

CHOL

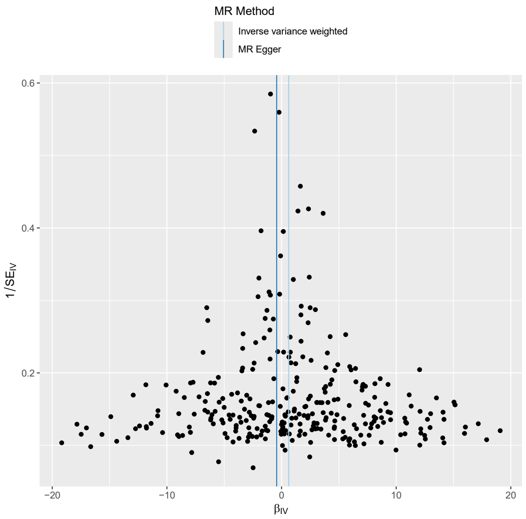

CRE

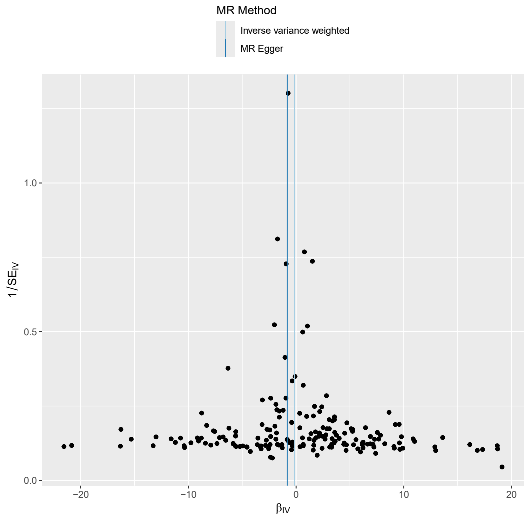

CRP

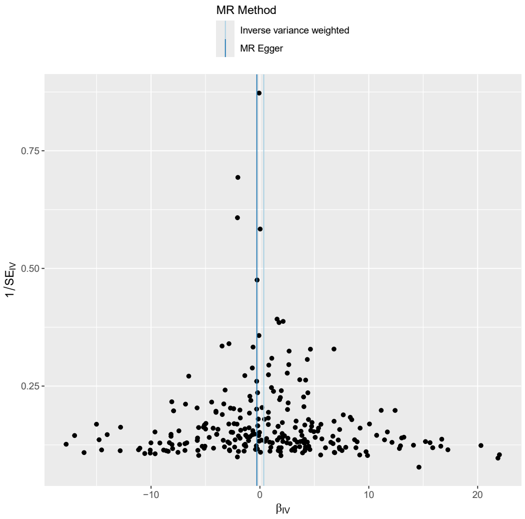

CYS

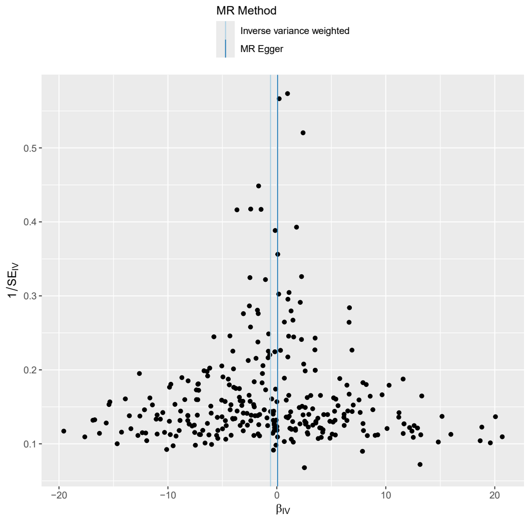

EGFR

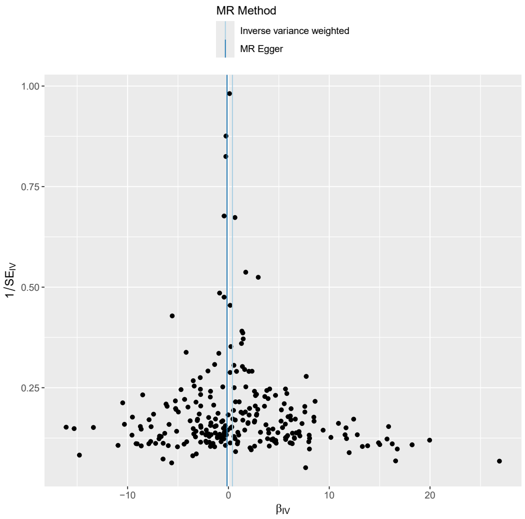

GGT

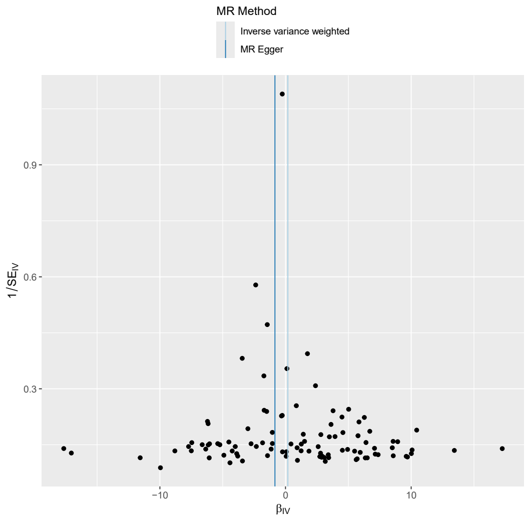

GLU

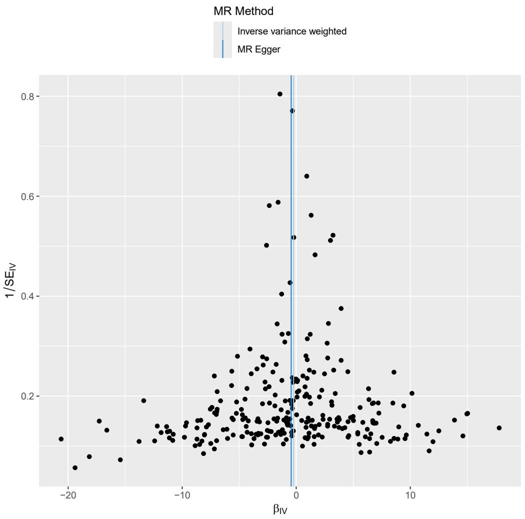

HBA1C

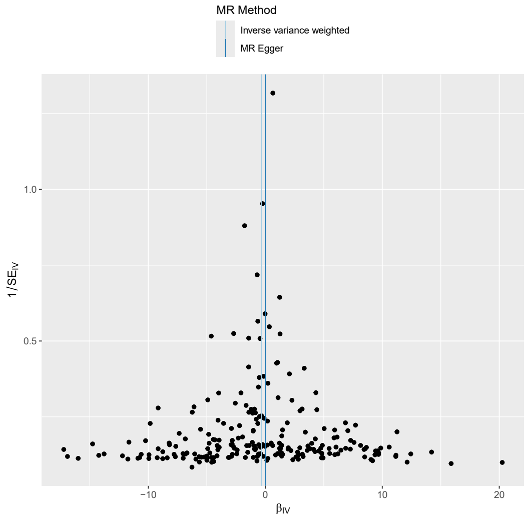

HDL

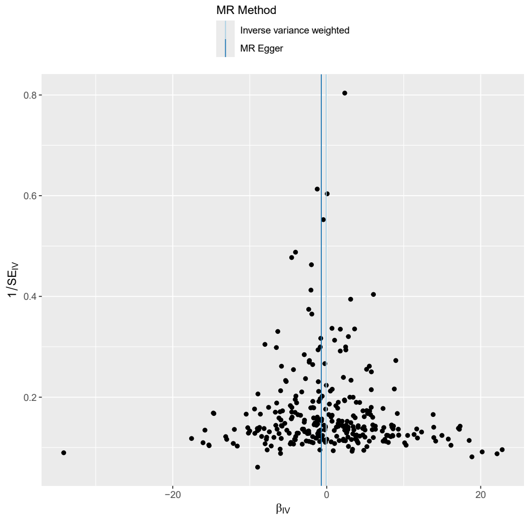

IGF-1

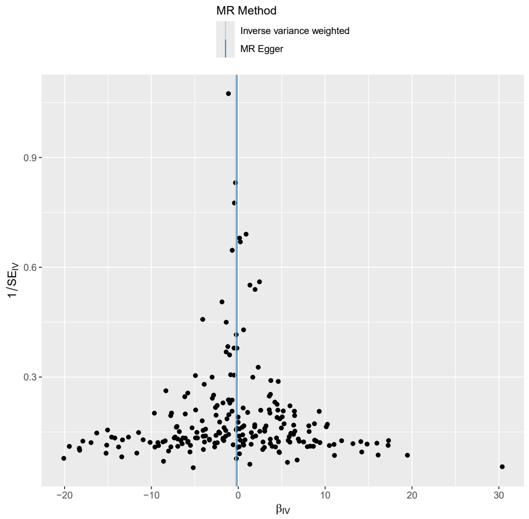

LDLD

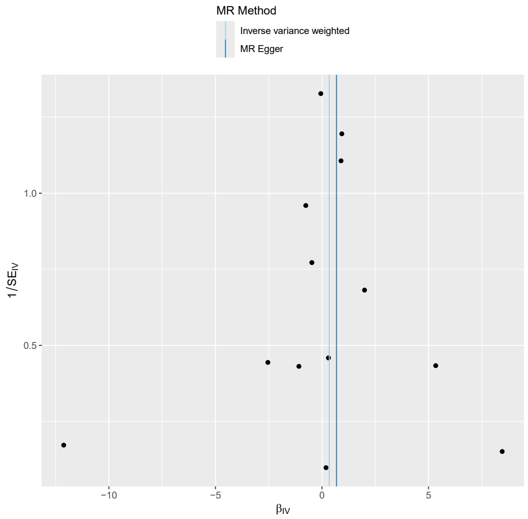

LPA

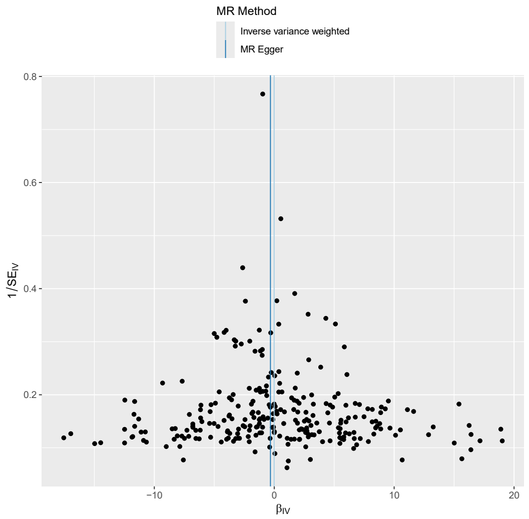

NAP

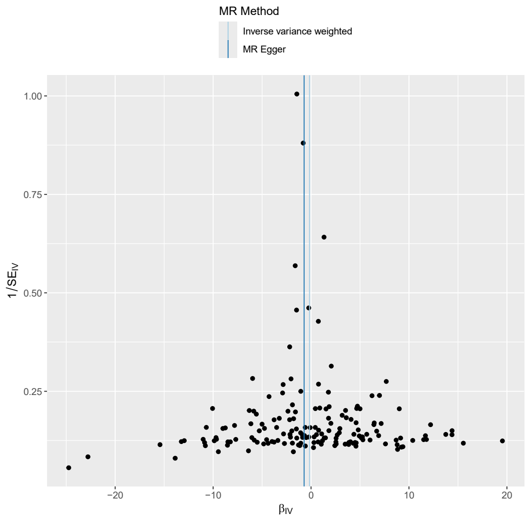

PHOS

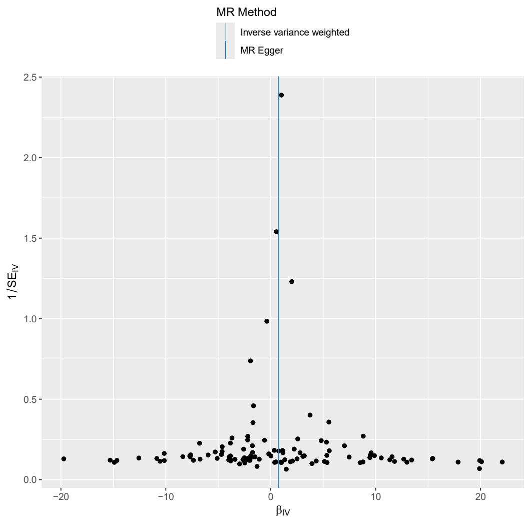

TBIL

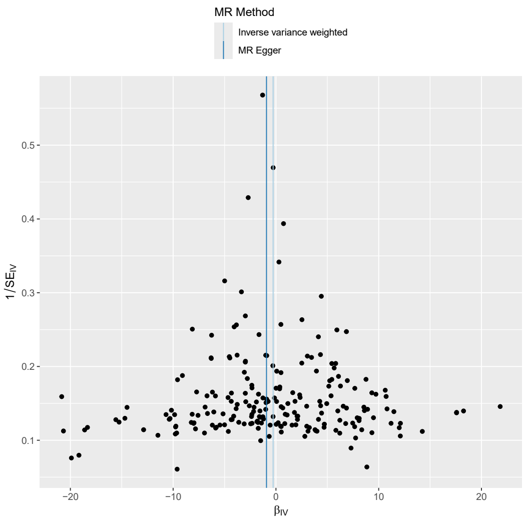

TP

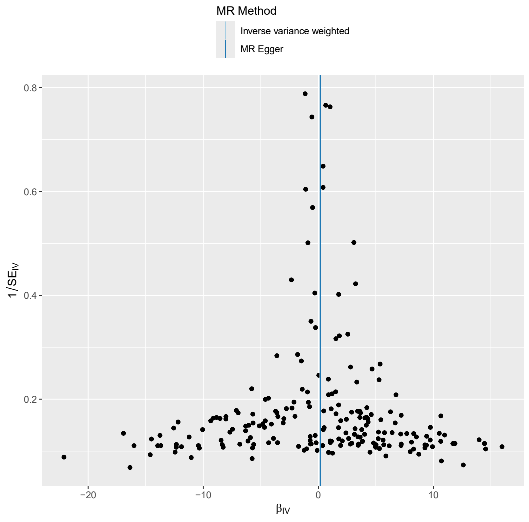

TRIG

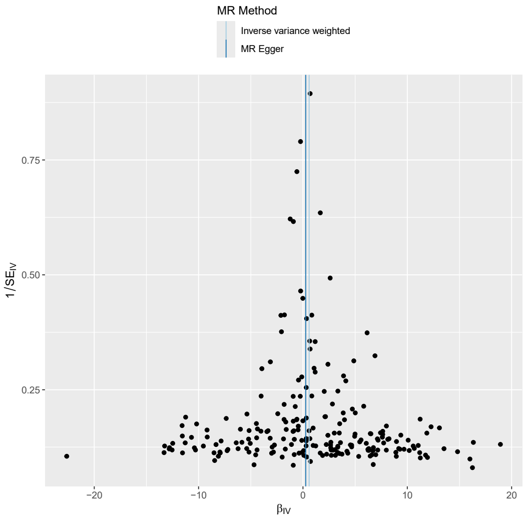

UA

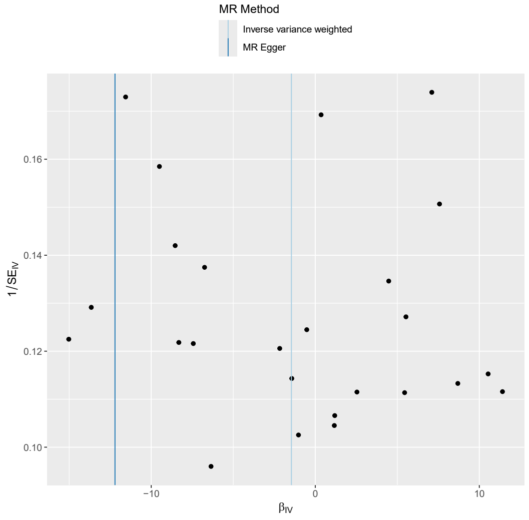

UCR

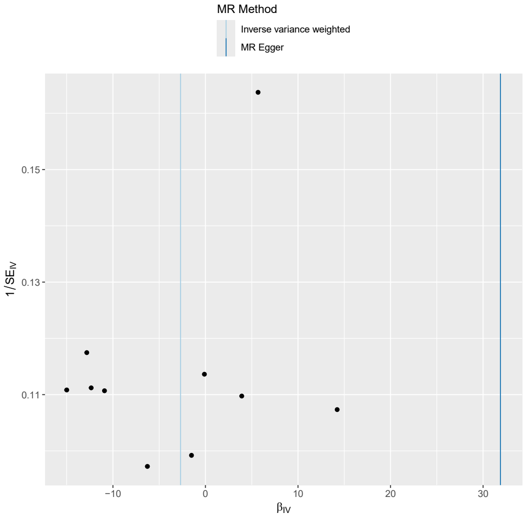

URK

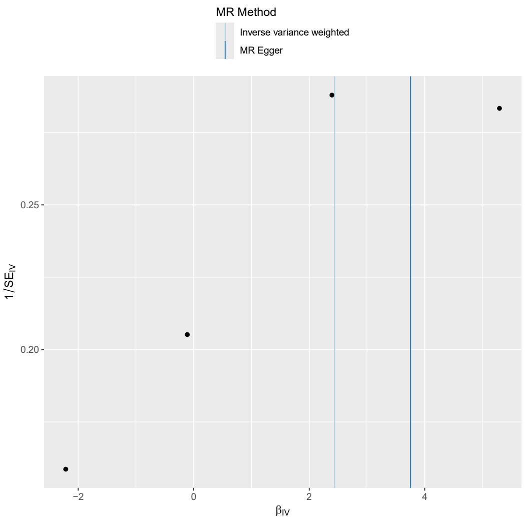

URMA

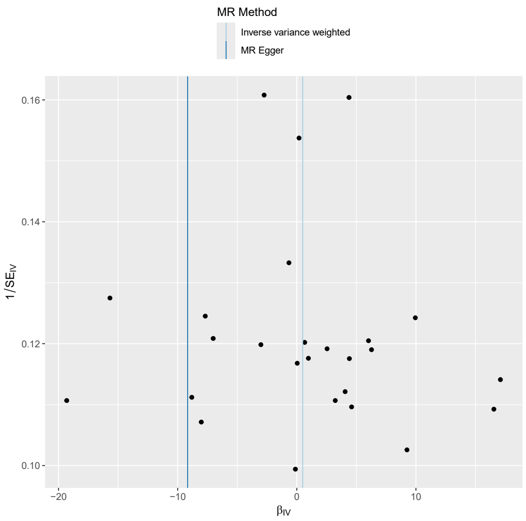

URNA

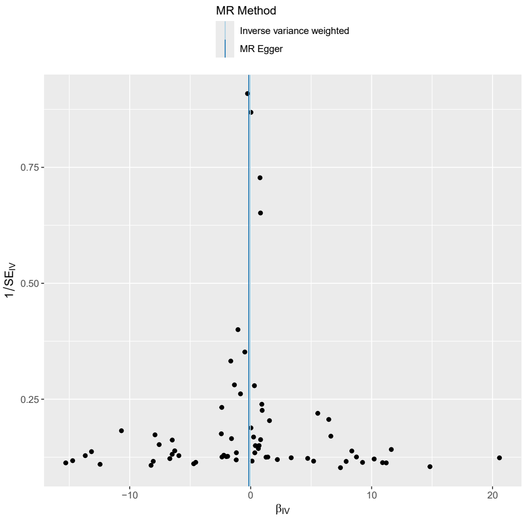

VITD

funnel plot of KCa

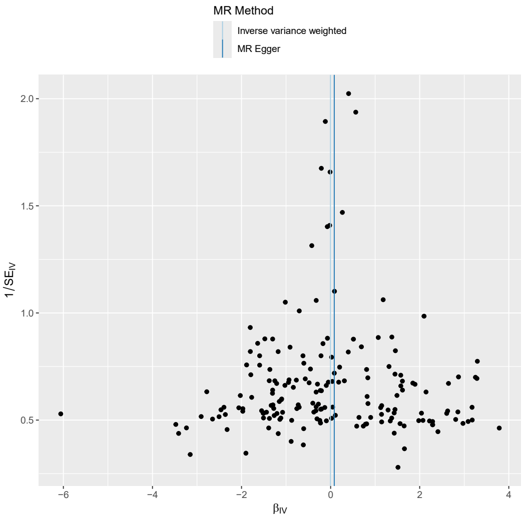

ALB

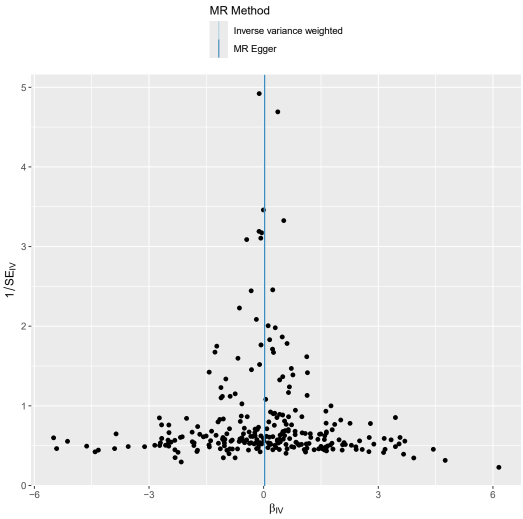

ALP

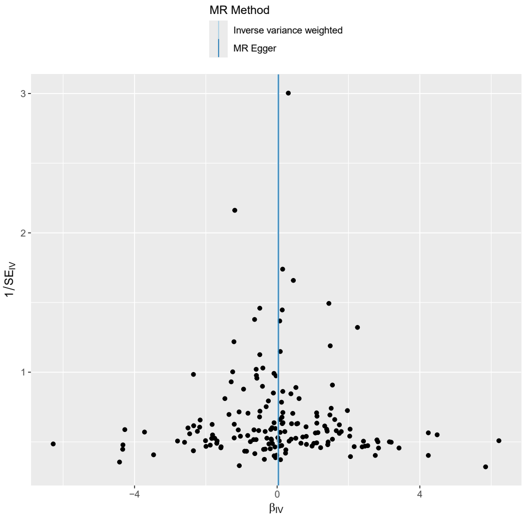

ALT

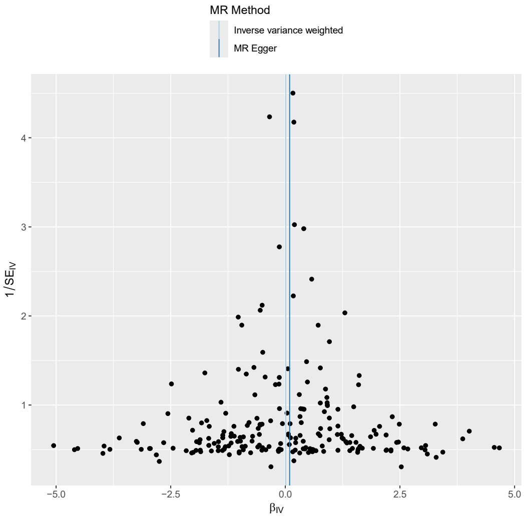

APOA

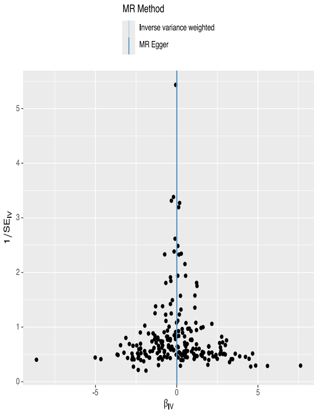

APOB

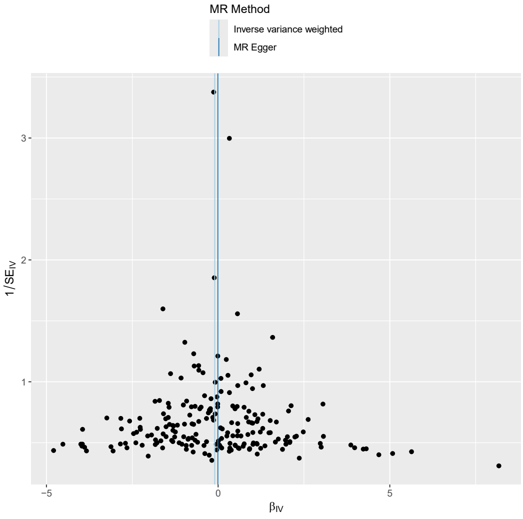

AST

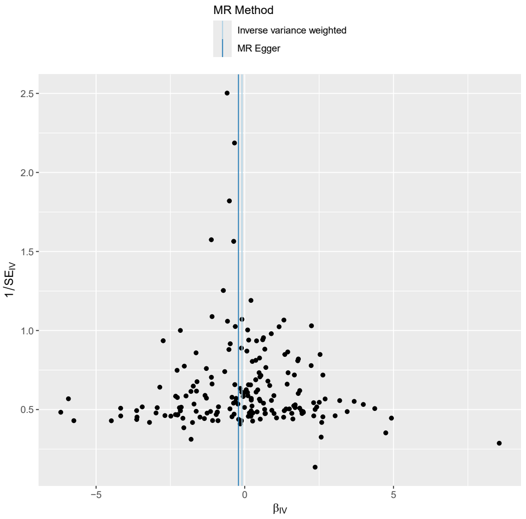

AST/ALT

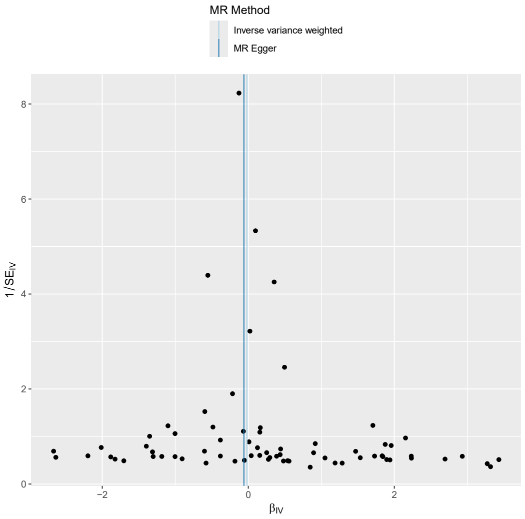

BILD

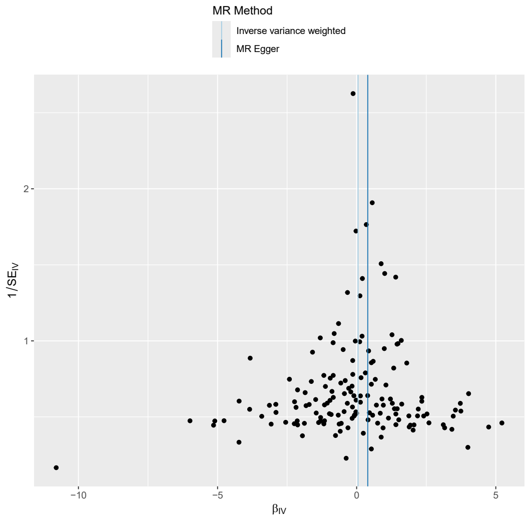

BUN

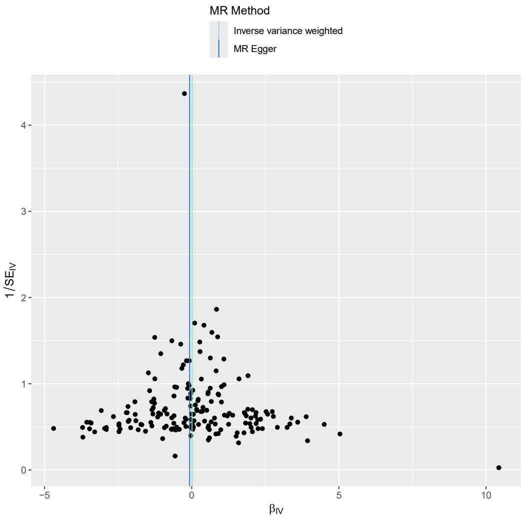

CA

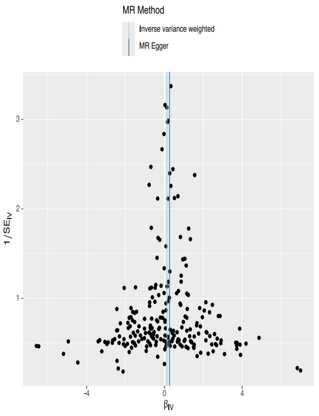

CHOL

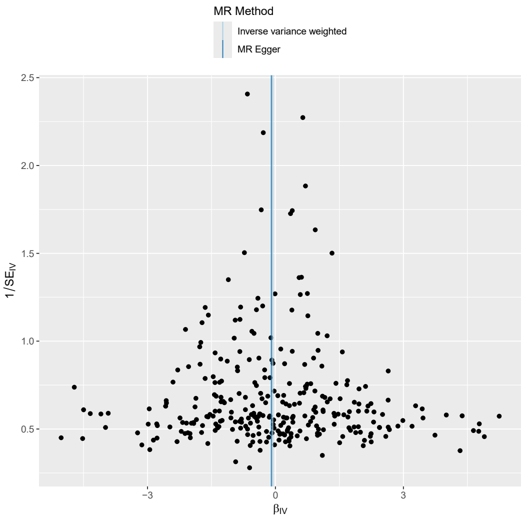

CRE

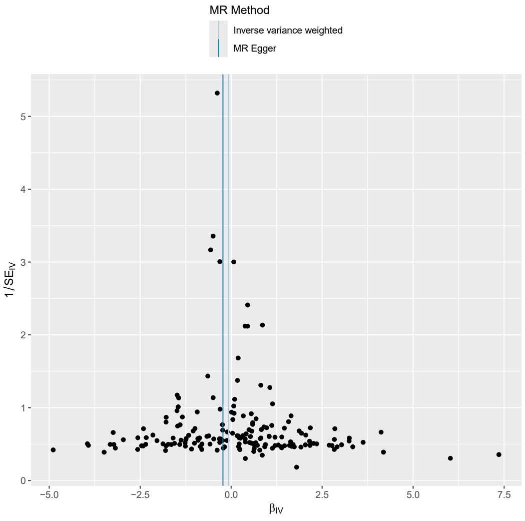

CRP

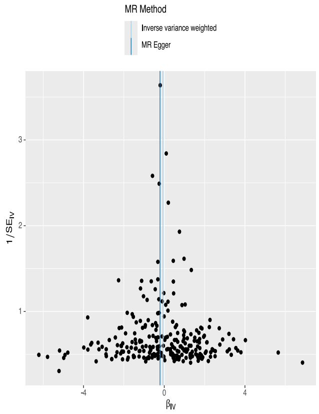

CYS

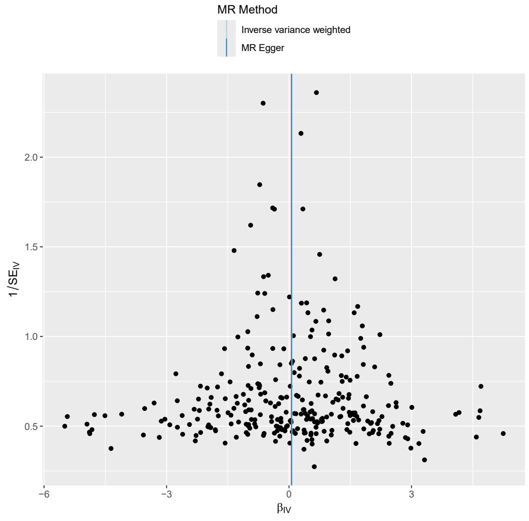

EGFR

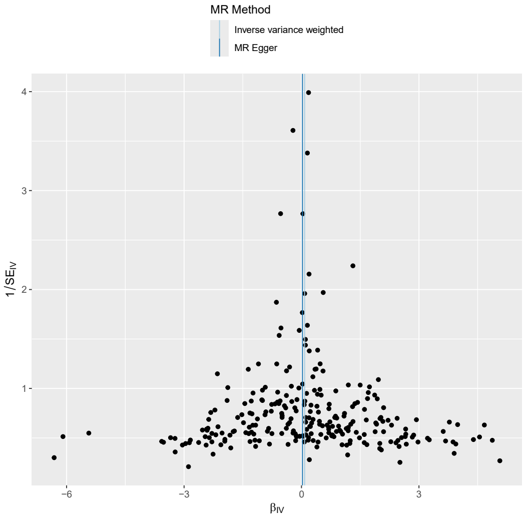

GGT

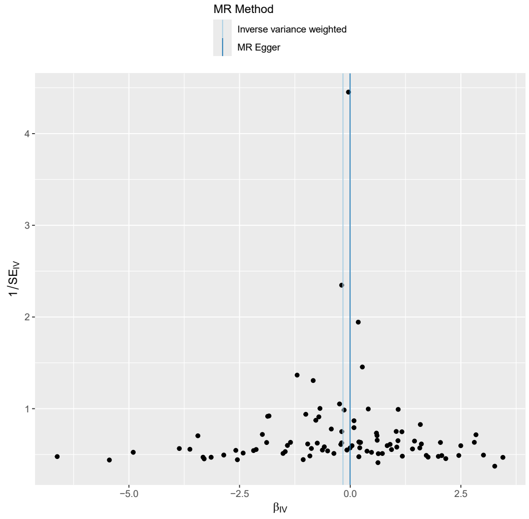

GLU

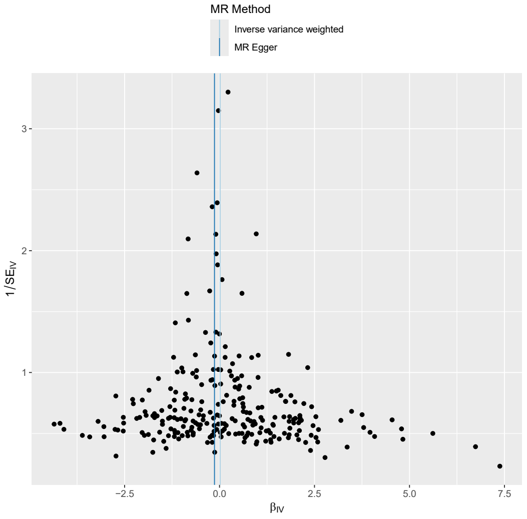

HBA1C

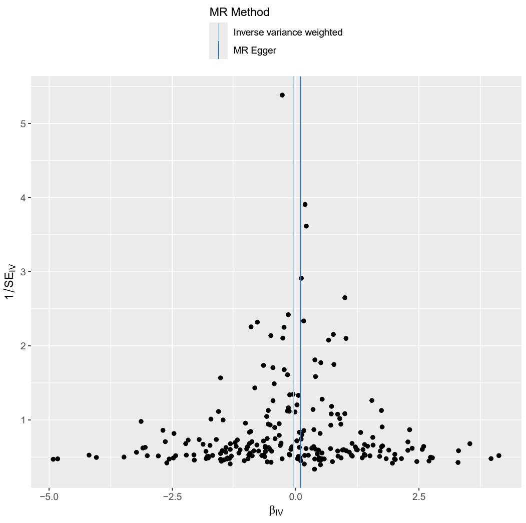

HDL

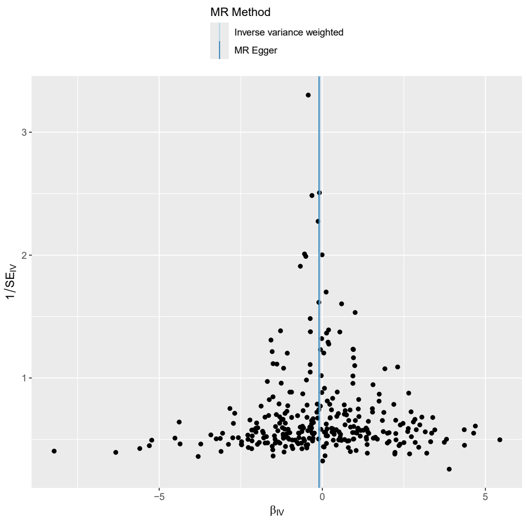

IGF-1

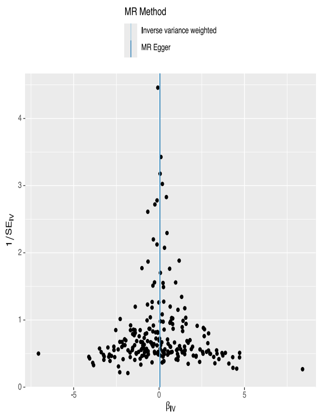

LDLD

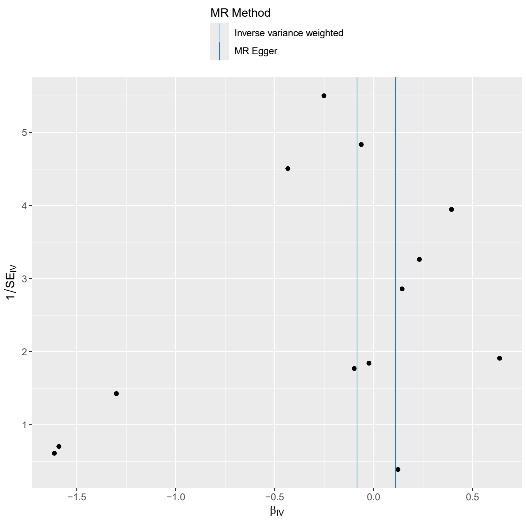

LPA

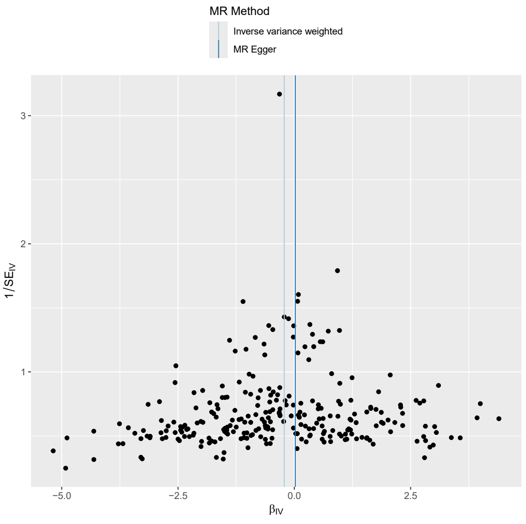

NAP

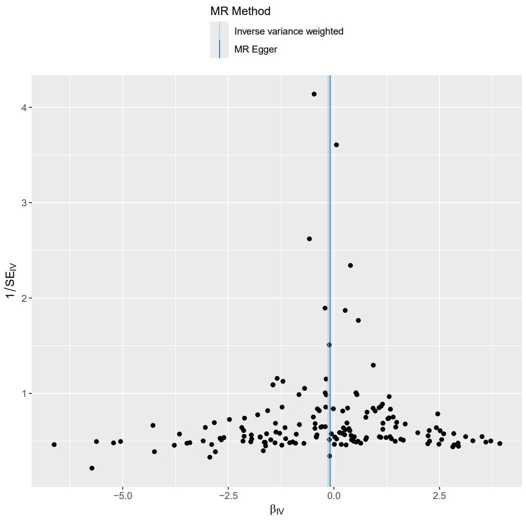

PHOS

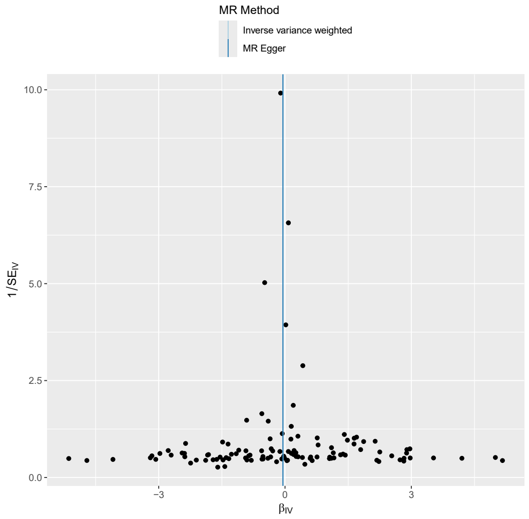

TBIL

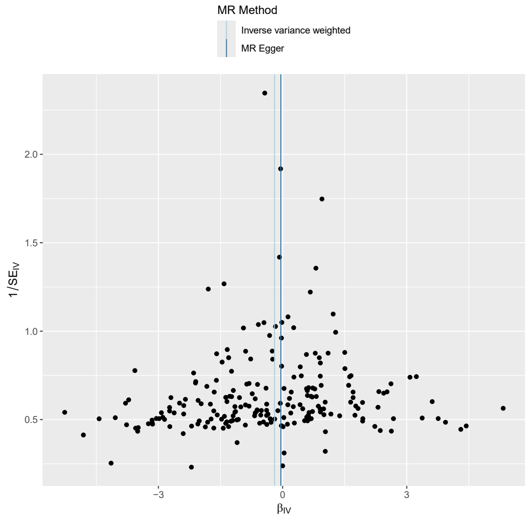

TP

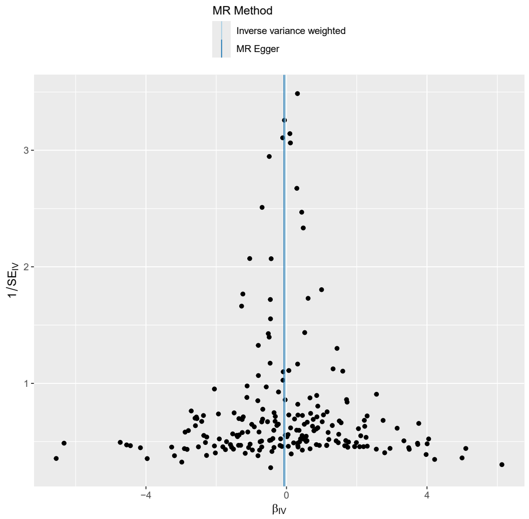

TRIG

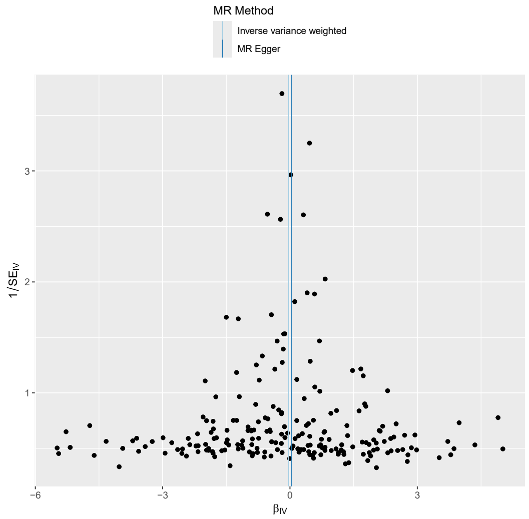

UA

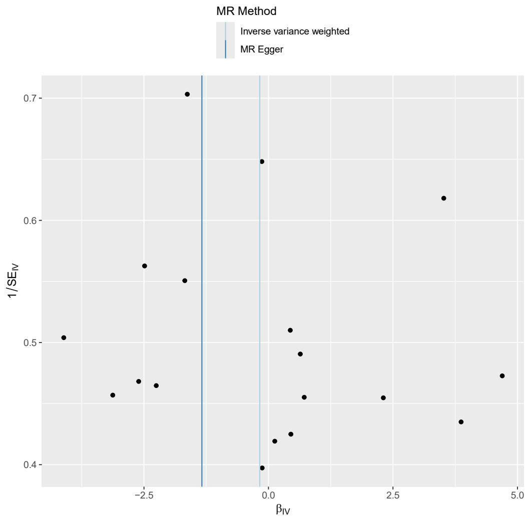

UCR

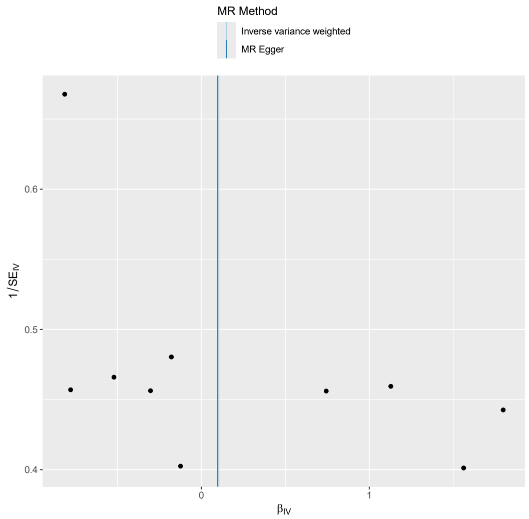

URK

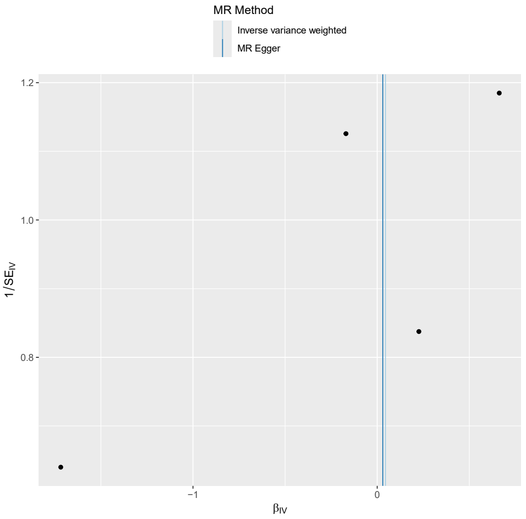

URMA

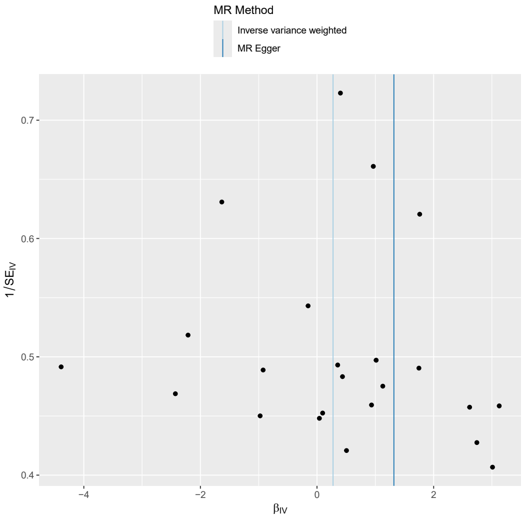

URNA

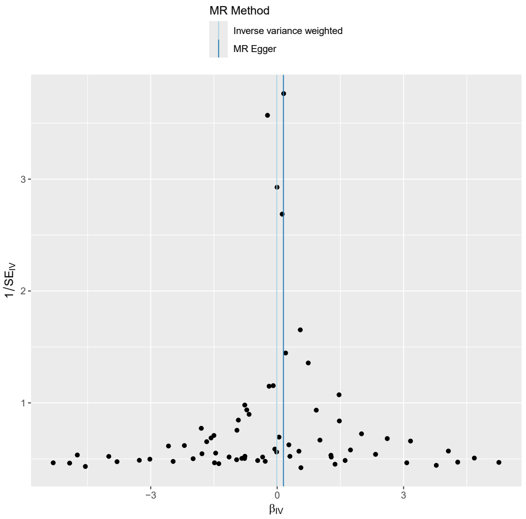

VITD

STROBE-MR checklist of recommended items to address in reports of Mendelian randomization studies[1 2]

Item No.	Section		Checklist item
1	**TITLE and ABSTRACT**		Indicate Mendelian randomization (MR) as the study's design in the title and/or the abstract if that is a main purpose of the study
	**INTRODUCTION**		
2	**Background**		Explain the scientific background and rationale for the reported study. What is the exposure? Is a potential causal relationship between exposure and outcome plausible? Justify why MR is a helpful method to address the study question
3	**Objectives**		State specific objectives clearly, including pre-specified causal hypotheses (if any). State that MR is a method that, under specific assumptions, intends to estimate causal effects
	**METHODS**		
4	**Study design and data sources**		Present key elements of the study design early in the article. Consider including a table listing sources of data for all phases of the study. For each data source contributing to the analysis, describe the following:
		a)	Setting: Describe the study design and the underlying population, if possible. Describe the setting, locations, and relevant dates, including periods of recruitment, exposure, follow-up, and data collection, when available.
		b)	Participants: Give the eligibility criteria, and the sources and methods of selection of participants. Report the sample size, and whether any power or sample size calculations were carried out prior to the main analysis
		c)	Describe measurement, quality control and selection of genetic variants
		d)	For each exposure, outcome, and other relevant variables, describe methods of assessment and diagnostic criteria for diseases
		e)	Provide details of ethics committee approval and participant informed consent, if relevant
5	**Assumptions**		Explicitly state the three core IV assumptions for the main analysis (relevance, independence and exclusion restriction) as well assumptions for any additional or sensitivity analysis
6	**Statistical methods: main analysis**		Describe statistical methods and statistics used
		a)	Describe how quantitative variables were handled in the analyses (i.e., scale, units, model)
		b)	Describe how genetic variants were handled in the analyses and, if applicable, how their weights were selected
		c)	Describe the MR estimator (e.g. two-stage least squares, Wald ratio) and related statistics. Detail the included covariates and, in case of two-sample MR, whether the same covariate set was used for adjustment in the two samples
		d)	Explain how missing data were addressed
		e)	If applicable, indicate how multiple testing was addressed

7	**Assessment of assumptions**		Describe any methods or prior knowledge used to assess the assumptions or justify their validity
8	**Sensitivity analyses and additional analyses**		Describe any sensitivity analyses or additional analyses performed (e.g. comparison of effect estimates from different approaches, independent replication, bias analytic techniques, validation of instruments, simulations)
9	**Software and pre-registration**		
		a)	Name statistical software and package(s), including version and settings used
		b)	State whether the study protocol and details were pre-registered (as well as when and where)
	**RESULTS**		
10	**Descriptive data**		
		a)	Report the numbers of individuals at each stage of included studies and reasons for exclusion. Consider use of a flow diagram
		b)	Report summary statistics for phenotypic exposure(s), outcome(s), and other relevant variables (e.g. means, SDs, proportions)
		c)	If the data sources include meta-analyses of previous studies, provide the assessments of heterogeneity across these studies
		d)	For two-sample MR:<br>i. Provide justification of the similarity of the genetic variant-exposure associations between the exposure and outcome samples<br>ii. Provide information on the number of individuals who overlap between the exposure and outcome studies
11	**Main results**		
		a)	Report the associations between genetic variant and exposure, and between genetic variant and outcome, preferably on an interpretable scale
		b)	Report MR estimates of the relationship between exposure and outcome, and the measures of uncertainty from the MR analysis, on an interpretable scale, such as odds ratio or relative risk per SD difference
		c)	If relevant, consider translating estimates of relative risk into absolute risk for a meaningful time period
		d)	Consider plots to visualize results (e.g. forest plot, scatterplot of associations between genetic variants and outcome versus between genetic variants and exposure)
12	**Assessment of assumptions**		
		a)	Report the assessment of the validity of the assumptions
		b)	Report any additional statistics (e.g., assessments of heterogeneity across genetic variants, such as I^2, Q statistic or E-value)
13	**Sensitivity analyses and additional analyses**		

		a)	Report any sensitivity analyses to assess the robustness of the main results to violations of the assumptions
		b)	Report results from other sensitivity analyses or additional analyses
		c)	Report any assessment of direction of causal relationship (e.g., bidirectional MR)
		d)	When relevant, report and compare with estimates from non-MR analyses
		e)	Consider additional plots to visualize results (e.g., leave-one-out analyses)
	**DISCUSSION**		
14	**Key results**		Summarize key results with reference to study objectives
15	**Limitations**		Discuss limitations of the study, taking into account the validity of the IV assumptions, other sources of potential bias, and imprecision. Discuss both direction and magnitude of any potential bias and any efforts to address them
16	**Interpretation**		
		a)	Meaning: Give a cautious overall interpretation of results in the context of their limitations and in comparison with other studies
		b)	Mechanism: Discuss underlying biological mechanisms that could drive a potential causal relationship between the investigated exposure and the outcome, and whether the gene-environment equivalence assumption is reasonable. Use causal language carefully, clarifying that IV estimates may provide causal effects only under certain assumptions
		c)	Clinical relevance: Discuss whether the results have clinical or public policy relevance, and to what extent they inform effect sizes of possible interventions
17	**Generalizability**		Discuss the generalizability of the study results (a) to other populations, (b) across other exposure periods/timings, and (c) across other levels of exposure
	**OTHER INFORMATION**		
18	**Funding**		Describe sources of funding and the role of funders in the present study and, if applicable, sources of funding for the databases and original study or studies on which the present study is based
19	**Data and data sharing**		Provide the data used to perform all analyses or report where and how the data can be accessed, and reference these sources in the article. Provide the statistical code needed to reproduce the results in the article, or report whether the code is publicly accessible and if so, where
20	**Conflicts of Interest**		All authors should declare all potential conflicts of interest

This checklist is copyrighted by the Equator Network under the Creative Commons Attribution 3.0 Unported (CC BY 3.0) license.

1. Skrivankova VW, Richmond RC, Woolf BAR, Yarmolinsky J, Davies NM, Swanson SA, et al. Strengthening the Reporting of Observational Studies in Epidemiology using Mendelian Randomization (STROBE-MR) Statement. JAMA. 2021;under review.

2. Skrivankova VW, Richmond RC, Woolf BAR, Davies NM, Swanson SA, VanderWeele TJ, et al. Strengthening the Reporting of Observational Studies in Epidemiology using Mendelian Randomisation (STROBE-MR): Explanation and Elaboration. BMJ. 2021;375:n2233.
